# Construction of a Library of Fatty Acid Esters of Hydroxy Fatty Acids

**DOI:** 10.3390/molecules30020286

**Published:** 2025-01-13

**Authors:** Olga G. Mountanea, Charikleia S. Batsika, Christiana Mantzourani, Christoforos G. Kokotos, George Kokotos

**Affiliations:** 1 Department of Chemistry, National and Kapodistrian University of Athens, 15771 Athens, Greece; olgamount@chem.uoa.gr (O.G.M.); cbatsika@chem.uoa.gr (C.S.B.); chrmantz@chem.uoa.gr (C.M.); ckokotos@chem.uoa.gr (C.G.K.); 2 Center of Excellence for Drug Design and Discovery, National and Kapodistrian University of Athens, 15771 Athens, Greece

**Keywords:** bioactive lipids, FAHFAs, hydroacylation, hydroxy fatty acids, photochemistry

## Abstract

Fatty Acid Esters of Hydroxy Fatty Acids (FAHFAs) have emerged as extraordinary bioactive lipids, exhibiting diverse bioactivities, from the enhancement of insulin secretion and the optimization of blood glucose absorption to anti-inflammatory effects. The intricate nature of FAHFAs’ structure reflects a synthetic challenge that requires the strategic introduction of ester bonds along the hydroxy fatty acid chain. Our research seeks to create an effective methodology for generating varied FAHFA derivatives. Our primary approach centers on a photochemical hydroacylation reaction, merging terminal alkenes, either *ω*-alkenoic acids or *ω*-alkenyl alcohols, with commercially available aldehydes. This transformative, environmentally friendly process, orchestrated by phenylglyoxylic acid as the photoinitiator, serves as the linchpin in establishing a practical and relatively simple method for constructing a library of racemic FAHFAs. The ketones produced by the photochemical reactions are easily converted to hydroxy derivatives, which are coupled with caproic, palmitic, or oleic acid, providing a large set of FAHFAs, which broaden our ability for future structure–activity relationship studies.

## 1. Introduction

**Figure 1:**
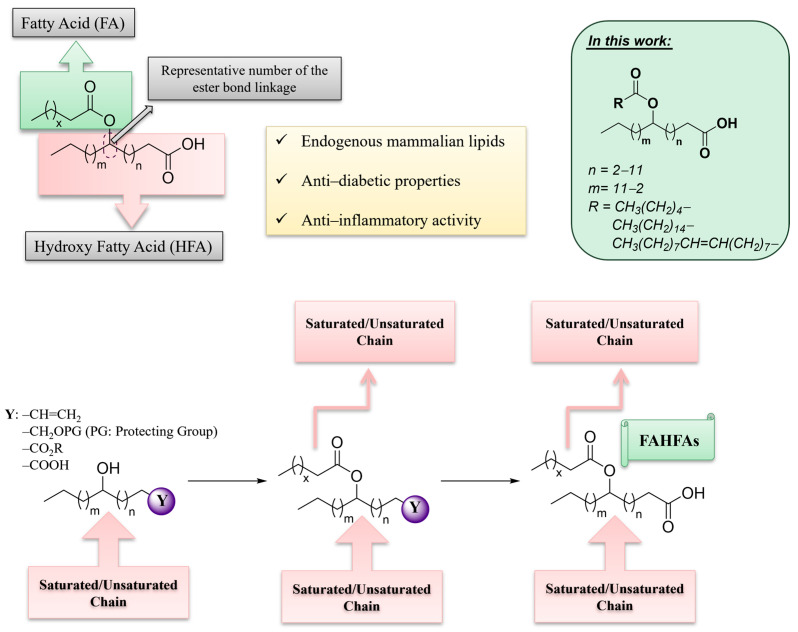
General structure of Fatty Acid Esters of Hydroxy Fatty Acids (FAHFAs) and schematic overview of the basic strategies for the synthesis of FAHFAs.

Among the diverse classes of bioactive lipids, Fatty Acid Esters of Hydroxy Fatty Acids (FAHFAs) constitute a unique class of endogenous lipids, exhibiting fascinating biological properties [1,2,3]. In their seminal work in 2014, Kahn and her colleagues, performing lipidomics analysis of adipose tissue from mice engineered to overexpress the glucose transporter GLUT4 (AG4OX mice), uncovered a novel category of mammalian lipids distinguished by a branched ester connection linking a fatty acid (FA) with a hydroxy fatty acid (HFA), referred to as FAHFAs (Figure 1) [4]. For convenience, abbreviations will be used throughout this work (e.g., CA for caproic acid, PA for palmitic acid, SA for stearic acid and OA for oleic acid, prefixed with “H” for hydroxy fatty acids [HFAs] and a unique number representing the location of the branch). FAHFAs belong to a family of estolides, which are fatty acid oligomers [1]. They are endogenously produced in insects [5] and mammals, such as mice [4,6] and humans [6,7]. The most studied family of FAHFAs is PAHSAs, which are synthesized *in vivo*. FAHFAs are naturally derived compounds identified in microalgae [8], breast milk [9], and various food sources, including grains, fruits and vegetables, oils, eggs, and meat [3,4,10,11], suggesting that FAHFAs may occur in other types of food and that mammals could potentially obtain FAHFAs through dietary intake.

FAHFAs can improve blood glucose uptake, enhance insulin secretion, and alleviate obesity-related inflammation in mammals, as well as reduce various pro-inflammatory responses [1]. More specifically, a correlation of FAHFA levels with insulin sensitivity was observed, and their presence was found to be diminished in the adipose tissue and serum of individuals with insulin resistance. Consequently, it is anticipated that these natural lipids could be potentially employed in the treatment of diabetes [4]. Kahn claimed that the administration of PAHSAs to mice lowers the body’s glycemia, improves glucose tolerance, and stimulates glucagon-like peptide-1 (GLP-1) and insulin secretion [4]. Additionally, within the scope of studying the bioactivities of FAHFAs, several groups aimed at exploring the potential anti-inflammatory effects of PAHSAs, during which they observed successful anti-inflammatory activity primarily attributed to the activation of G protein-coupled receptors and the downregulation of macrophage activation, as well as the reduction of pro-inflammatory cytokines [4,12,13,14,15]. Simultaneously, research on FAHFAs has exhibited promising outcomes in combatting ulcerative colitis [16], and these compounds are noted for their potential to fortify the immune system of infants, being transmitted through breast milk from mothers to their newborns during breastfeeding [9]. Moreover, FAHFAs are purported to function as activators of Nrf2, a transcription factor responsible for regulating the gene expression of antioxidants and enzymes. This action reinforces the idea that FAHFAs may also act as antioxidants, diminishing oxidative stress and amplifying the expression of cytoprotective antioxidant enzymes [17]. It is conceivable that various FAHFAs’ regio-isomers might not demonstrate identical properties or exhibit the same level of efficacy [4,18].

The significance of conducting a structure–activity relationship study, along with the absence of synthetic techniques at that time for generating chiral FAHFAs, prompted the creation of a synthetic method for producing the two distinct enantiomeric forms of 9-PAHSA, employing commercially available enantiopure epichlorohydrin as the starting material via a chiral pool approach [18]. A five-step process starting from commercially available (*R*)- or (*S*)-epichlorohydrin led to the synthesis of (*S*)- or (*R*)-9-PAHSA. Back in 2014, Kahn’s research team conducted the first synthesis of FAHFAs using an aldehyde as the starting material [4]. The same group published yet another synthetic methodology for the preparation of racemic FAHFAs, starting from the introduction of a tetrahydropyranyl group into commercially available *α,ω*-diols, mainly proposing 9-PAHSA synthesis in five steps [19]. In 2016, Balas and co-workers presented a study on the effective synthesis of FAHFAs, using commercially accessible terminal alkenes and alkynes, via a seven-step synthesis, which mainly led to 5-, 7-, 9- and 10-regio-isomers [20], while in 2018, Bielohuby and Tennagel’s group pursued an enantioselective approach for the synthesis of 5- and 9-PAHSAs using cross metathesis, in an attempt to investigate the characteristics of FAHFAs and explore the interactions of these compounds with different receptors [21]. In 2019, a different route for the asymmetric synthesis of FAHFAs, employing an enantioselective organocatalytic step resulting in the formation of chiral terminal epoxides, starting from monoprotected *α,ω*-diols and their subsequent ring opening by a Grignard reagent as the key-steps, was developed by our group [22]. Lastly, during 2019, Siegel conducted research for the synthesis of 9-PAHSA analogs, featuring varying numbers of carbons in the fatty acid chain [14]. These synthesized analogs underwent analysis for their anti-inflammatory properties, particularly in inhibiting the production of IL-6. The results indicated that among them, 9-(hexanoyloxy)octadecanoic acid (9-CAHSA) exhibited the most promising outcome, even surpassing the known anti-inflammatory capabilities of 9-PAHSA [14]. With an overview of all the strategies developed for the synthesis of FAHFAs (Figure 1) [23], it is observed that most of them target the synthesis of specific regio-isomers, mainly 9- and 5-, while a more general method leading to a wide variety of regio-isomers would be of practical value for studying the bioactivities of such lipids.

The diverse chemical composition within the FAHFAs superfamily (regarding the position of the ester group, the fatty- or hydroxy fatty-acid chain length, and the presence or absence of unsaturation) renders their synthesis particularly challenging. The objective of this current study was the development of a practical, high-yield, and few-synthetic-step method to generate a library of racemic FAHFAs with the ability to select the position of the ester bond along the HFA chain. Meanwhile, the previously reported study concerning the anti-inflammatory activity of FAHFA derivatives, notably the superior activity observed in 9-CAHSA [14], inspired us to expand our research. We envisioned that alteration in the FA chain, like the linkage of different fatty acids to the respective HFA, may affect the biological impact of FAHFAs. Consequently, we utilized three different FAs, caproic, palmitic, and oleic acid, contributing to the production of diverse regio-isomers of CAHSAs, PAHSAs, and OAHSAs. With these considerations in mind, we successfully developed a versatile and efficient method for synthesizing FAHFAs that accommodates the diverse chemical structures of the superfamily, while providing flexibility in the choice of starting materials to generate structurally diverse libraries. This modular approach, based on a photochemical hydroacylation, is both cost-effective and well suited for structure–activity relationship studies, paving the way for advanced research into the biological functions and therapeutic potential of FAHFAs.

## 2. Results and Discussion

Photochemistry utilizes light to enable complex chemical transformations, facilitating reactions previously considered challenging. Most examples employ metal-based catalysts, primarily Ru or Ir, having the advantage of adjusting their electronic characteristics through ligand coordination. However, the cost of metal-based catalysts along with their limited availability prompt scientists to explore alternatives, like small organic molecules. Known as photoorganocatalysis, this approach stands out for its environmentally friendly and cost-effective nature, thanks to moderate energy input and reaction conditions, offering a promising solution [24,25,26,27,28,29,30,31,32,33,34]. In 2020, Kokotos and co-workers developed a green, mild, metal-free photochemical protocol that is easy to operate, employing cheap household lamps as the irradiation source and phenylglyoxylic acid, a cheap, organic, and commercially available compound, as the photoinitiator for the hydroacylation of olefins without the need for any directing groups or additives [35].

Drawn from this particular light-driven approach, our group developed a photochemical method in 2021 for synthesizing a class of lipids with mostly unexplored biological properties. We employed the hydroacylation reaction to produce saturated oxo fatty acids (SOFAs), bearing the oxo functionality at various positions of the long aliphatic chain [36]. In this context, we chose to broaden our research scope by utilizing the previously established photochemical hydroacylation reaction to produce oxo fatty acids and various oxo derivatives. This process served as the fundamental step, enabling the generation of different families of FAHFAs.

**Scheme 1:**
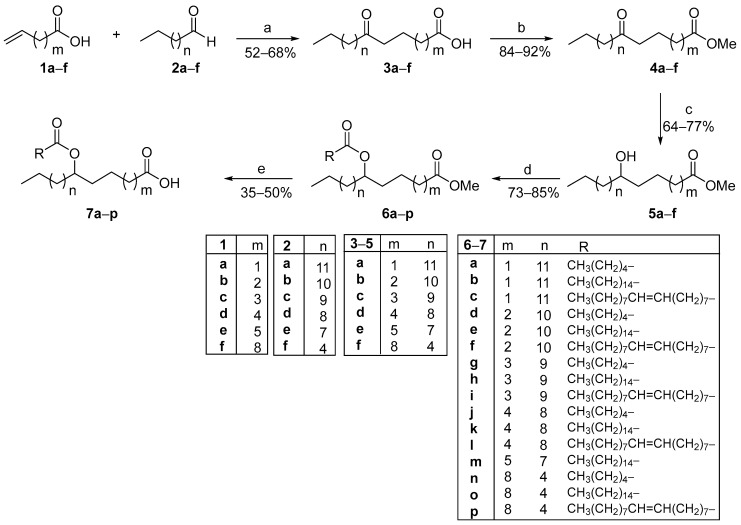
General synthetic route for the construction of FAHFAs from *ω*-alkenoic acids and aldehydes, using a photocatalytic hydroacylation protocol as the key step. (a) Phenylglyoxylic acid (20 mol%), H_2_O, hv (2 × 85 W CFL household bulbs); (b) CH_3_OH, conc. H_2_SO_4_; (c) NaBH_4_, CH_3_OH; (d) CH_3_(CH_2_)_4_COOH or CH_3_(CH_2_)_14_COOH or CH_3_(CH_2_)_7_CH=CH(CH_2_)_7_COOH, EDCI_·_HCl, Et_3_N, 4-dimethylaminopyridine, dry CH_2_Cl_2_; (e) LiOH_·_H_2_O, THF:H_2_O.

As depicted in Scheme 1, firstly, the desired OFAs were produced in a 52–68% yield from the photochemical hydroacylation reaction between commercially available terminal alkenoic acids **1a**–**f** and aliphatic aldehydes **2a**–**f**, utilizing phenylglyoxylic acid as the photoinitiator and water as the solvent under household lamp irradiation.

The synthesized OFAs **3a**–**f** were subsequently subjected to a Fischer esterification reaction in methanol and conc. H_2_SO_4_ to afford the corresponding methyl esters **4a**–**f** in excellent yields. In the next step, the reduction in the oxo functionality of **4a**–**f** by the employment of NaBH_4_ as the reducing agent resulted in secondary alcohols **5a**–**f**. Coupling reaction between alcohols **5a**–**f** and three different FAs (CA, PA and OA) was accomplished using EDCI_·_HCl as the coupling reagent, in the presence of Et_3_N and 4-dimethylaminopyridine (4-DMAP), leading to the formation of methyl esters **6a**–**p** in high yields. Finally, the desired FAHFAs **7a**–**p** were obtained after the basic hydrolysis of the methyl-ester group using LiOH_·_H_2_O. The partial hydrolysis of the second ester moiety occurred under the reaction conditions, though to a lesser extent, resulting in a slight reduction in the yields of the hydrolysis step.

**Scheme 2:**
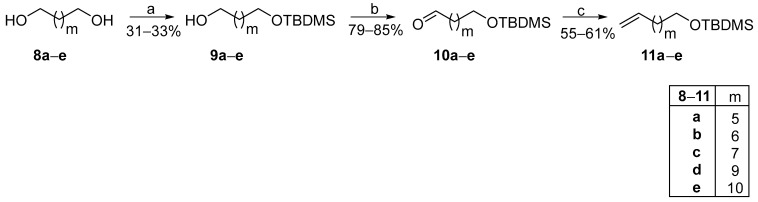
Synthetic pathway for the conversion of *α*,*ω*-commercially available diols to *ω*-alkenyl alcohols. (a) *tert*-Butyldimethylsilyl chloride, imidazole, 4-dimethylaminopyridine, dry *N*,*N*-dimethylformamide; (b) Pyridinium chlorochromate, dry CH_2_Cl_2_; (c) MePPh_3_Br, *n*-BuLi (1.6 M in hexanes), dry THF.

**Scheme 3:**
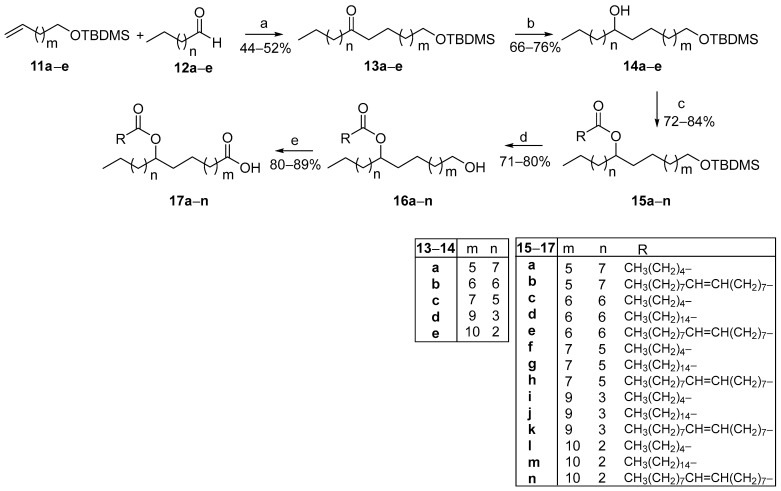
General synthetic route for the construction of FAHFAs from *ω*-alkenyl alcohols and aldehydes through photocatalytic hydroacylation. (a) Phenylglyoxylic acid (20 mol%), H_2_O, hv (2 × 85 W CFL household bulbs); (b) NaBH_4_, CH_3_OH; (c) CH_3_(CH_2_)_4_COOH or CH_3_(CH_2_)_14_COOH or CH_3_(CH_2_)_7_CH=CH(CH_2_)_7_COOH, EDCI_·_HCl, Et_3_N, 4-dimethylaminopyridine, dry CH_2_Cl_2_; (d) TBAF, dry THF; (e) Jones reagent, acetone.

The cost of *ω*-terminal alkenoic acids is exponentially becoming more expensive as their chain lengthens. Thus, in such cases, we envisaged the use of cheap commercially available *α,ω*-diols as starting materials, proposing a modified FAHFA synthetic route. As presented in Scheme 2, *α*,*ω*-diols **8a**–**e** were mono-protected with a *tert*-butyldimethylsilyl (TBDMS) protecting group and then, alcohols **9a**–**e** were oxidized to the corresponding aldehydes **10a**–**e**, using pyridinium chlorochromate (PCC) as the oxidant. The TBDMS protecting group was selected for its compatibility with our methodology and the specific requirements of the target molecules. Its stability ensured that other functional groups such as the oleic acid’s double bond remained unaffected. This would not have been feasible with the use of a benzyl protecting group, as its deprotection would also compromise the double bond. Although alternative protecting groups, including ester-based options, were evaluated, their deprotection via hydrolysis often led to partial hydrolysis, thereby reducing efficiency. Wittig olefination reaction between aldehydes **10a**–**e** and phosphonium ylide MePPH_3_Br produced TBDMS-protected alkenes **11a**–**e**, in good yields, which were employed in the photochemical process, along with commercially available aliphatic aldehydes **12a**–**e** to afford TBDMS-protected ketones **13a**–**e** (Scheme 3).

In a manner similar to Scheme 1, the reduction of the oxo group by NaBH_4_ led to the corresponding alcohols **14a**–**e**. Similarly, the coupling reaction between alcohols **14a**–**e** and caproic, palmitic, or oleic acid afforded **15a**–**n** in high yields. Deprotection of the TBDMS group by treatment with tetra-*n*-butylammonium fluoride (TBAF), followed by Jones oxidation led to the desired FAHFAs **17a**–**n**.

## 3. Materials and Methods

### 3.1. General Remarks

All commercially available products and solvents were purchased from Fluorochem (Fluorochem Ltd., Hadfield, Glossop, UK), Sigma-Aldrich (Sigma-Aldrich, Saint Louis, MO, USA), Fluka (Fluka Chemicals Ltd., Gillingham, Dorset, UK), Merck (Merck, Darmstadt, Germany), and Alfa Aesar (Alfa Aesar, Ward Hill, MA, USA). The chromatographic purification of products was accomplished using forced-flow chromatography on Merck^®^ (Merck, Darmstadt, Germany) Kieselgel 60 F254 230–400 mesh. Thin-layer chromatography (TLC) was performed on aluminum-backed silica plates (0.2 mm, 60 F254). The visualization of the developed chromatogram was performed by fluorescence quenching using phosphomolybdic acid. Melting points were measured on a Buchi 530 apparatus (Buchi, Flawil, Switzerland) and were uncorrected. High-resolution mass spectra were obtained on a Bruker Maxis Impact QTOF spectrometer (Bruker Daltonics, Bremen, Germany). ^1^H-NMR and ^13^C-NMR spectra were recorded on an Avance III HD Bruker 400 MHz (Bruker, Fällanden, Switzerland) (400 MHz and 100 MHz, respectively) or a Varian Mercury (Varian, Palo Alto, CA, USA) (200 MHz and 50 MHz, respectively) and are internally referenced to residual solvent signals. Data for ^1^H-NMR are reported as follows: chemical shift (*δ* ppm), multiplicity (s = singlet, t = triplet, quin = quintet, m = multiplet, br s = broad signal), coupling constant, integration, and assignment. Data for ^13^C-NMR are reported in terms of a chemical shift (*δ* ppm). The synthesis of **3a**–**f** and **13b** was carried out as described by Batsika et al. [36], and their spectroscopic data were in accordance with the literature. Additionally, compounds **4a**–**f** [37,38,39,40,41,42], **5a** [39], **5b** [43], **5c,d** [40], **5e** [42], **5f** [44], **9a**–**d** [45,46,47,48], **9e** [36], **10a**–**d** [49,50,51,52], **10e** [36], **11a,b** [53,54], **11c** [51], **11d** [55], and **11e** [36] were prepared by known procedures, and their spectroscopic data were identical with those in the literature.

### 3.2. General Procedure for the Photochemical Reaction of Aldehydes with Alkenes

In a glass vial equipped with a PTFE-coated stirring bar, the appropriate alkene (0.50 mmol), the aldehyde (1.50 mmol), and the phenylglyoxylic acid (15 mg, 0.10 mmol) were added using water (1 mL) as the reaction solvent. The vial was sealed with a screw cap and left stirring under household bulb irradiation (2 × 85 W household lamps) for 48 h. The reaction mixture was extracted with CH_2_Cl_2_ (3 × 3 mL). The combined organic layers were dried over Na_2_SO_4_, and the solvent was removed *in vacuo*. The desired oxo fatty acids **3a**–**f** were isolated by precipitation with hexane (3–5 mL) (cooled with an external ice bath). Ketones **13a**–**e** were purified by flash chromatography on silica gel eluting with a petroleum ether (bp 40–60 °C)/ethyl acetate 95/5 system.

1-((*tert*-Βutyldimethylsilyl)oxy)octadecan-9-one (**13a**). Colorless oil; Yield: 44%; ^1^H-NMR (400 MHz, CDCl_3_): *δ* 3.59 (t, *J* = 6.6 Hz, 2H, CH_2_OTBDMS), 2.37 (t, *J* = 7.4 Hz, 4H, 2 × COCH_2_), 1.59–1.44 (m, 6H, 3 × CH_2_), 1.31–1.23 (m, 20H, 10 × CH_2_), 0.91–0.84 (m, 12H, 4 × CH_3_), 0.04 (s, 6H, 2 × CH_3_); ^13^C-NMR (100 MHz, CDCl_3_): *δ* 211.6, 63.3, 42.8, 42.8, 32.8, 31.9, 29.4, 29.4, 29.4, 29.3, 29.3, 29.2, 26.0, 25.7, 23.9, 23.9, 22.6, 18.4, 14.1, −5.3; HRMS (ESI^+^): *m*/*z* calcd for C_24_H_50_NaO_2_Si^+^: 421.3472; [M + Na]^+^ found: 421.3477.

18-((*tert*-Butyldimethylsilyl)oxy)octadecan-8-one (**13c**). Colorless oil; Yield: 49%; ^1^H-NMR (200 MHz, CDCl_3_): *δ* 3.54 (t, *J* = 6.5 Hz, 2H, CH_2_OTBDMS), 2.33 (t, *J* = 7.4 Hz, 4H, 2 × COCH_2_), 1.57–1.41 (m, 6H, 3 × CH_2_), 1.28–1.16 (m, 20H, 10 × CH_2_), 0.86–0.78 (m, 12H, 4 × CH_3_), -0.01 (s, 6H, 2 × CH_3_); ^13^C-NMR (50 MHz, CDCl_3_): *δ* 211.3, 63.2, 42.7, 32.8, 31.6, 29.5, 29.3, 29.1, 29.0, 25.9, 25.7, 23.8, 22.5, 18.2, 14.0, −5.4; HRMS (ESI^+^): *m*/*z* calcd for C_24_H_50_NaO_2_Si^+^: 421.3472; [M + Na]^+^ found: 421.3472.

18-((*tert*-Butyldimethylsilyl)oxy)octadecan-6-one (**13d**). Colorless oil; Yield: 52%; ^1^H-NMR (400 MHz, CDCl_3_): *δ* 3.59 (t, *J* = 6.6 Hz, 2H, CH_2_OTBDMS), 2.37 (t, *J* = 7.4 Hz, 4H, 2 × COCH_2_), 1.57–1.47 (m, 6H, 3 × CH_2_), 1.32–1.24 (m, 20H, 10 × CH_2_), 0.91–0.86 (m, 12H, 4 × CH_3_), 0.04 (s, 6H, 2 × CH_3_); ^13^C-NMR (100 MHz, CDCl_3_): *δ* 211.7, 63.3, 42.8, 42.8, 32.9, 31.4, 29.6, 29.6, 29.5, 29.4, 29.4, 29.3, 26.0, 25.8, 23.9, 23.6, 22.4, 18.4, 13.9, −5.3; HRMS (ESI^+^): *m*/*z* calcd for C_24_H_50_NaO_2_Si^+^: 421.3472; [M + Na]^+^ found: 421.3478.

18-((*tert*-Butyldimethylsilyl)oxy)octadecan-5-one (**13e**). Colorless oil; Yield: 46%; ^1^H-NMR (200 MHz, CDCl_3_): *δ* 3.54 (t, *J* = 6.5 Hz, 2H, CH_2_OTBDMS), 2.33 (t, *J* = 7.2 Hz, 4H, 2 × COCH_2_), 1.60–1.40 (m, 6H, 3 × CH_2_), 1.32–1.16 (m, 20H, 10 × CH_2_), 0.93–0.77 (m, 12H, 4 × CH_3_), −0.01 (s, 6H, 2 × CH_3_); ^13^C-NMR (50 MHz, CDCl_3_): *δ* 211.2, 63.2, 42.7, 42.4, 32.8, 29.5, 29.5, 29.4, 29.4, 29.2, 25.9, 25.7, 23.8, 22.3, 18.2, 13.8, −5.4; HRMS (ESI^+^): *m*/*z* calcd for C_24_H_50_NaO_2_Si^+^: 421.3472; [M + Na]^+^ found: 421.3478.

### 3.3. General Procedure for the Synthesis of Alcohols via Oxo Functionality Reduction

TBDMS-protected ketone **13a**–**e** (1.00 mmol) in MeOH (5 mL) was added to a round-bottomed flask at 0 °C. Next, NaBH_4_ (189 mg, 5.00 mmol) was added under vigorous stirring. The solution was stirred for 1 h, and then methanol was removed under reduced pressure. The reaction mixture was washed with a saturated aqueous solution of NH_4_Cl (3 × 10 mL) and the combined organic layers were collected and dried with Na_2_SO_4_, and the solvent was removed under reduced pressure. The crude mixture was purified by flash chromatography (petroleum ether (bp 40–60 °C)/ethyl acetate, 70/30 for methyl esters, or 90/10 for TBDMS-protected compounds) to afford the desired alcohol.

1-((*tert*-Βutyldimethylsilyl)oxy)octadecan-9-ol (**14a**) [22]. Colorless oil; Yield: 74%; ^1^H-NMR (200 MHz, CDCl_3_): *δ* 3.64–3.53 (m, 3H, CH_2_OTBDMS and C*H*OH), 1.56–1.22 (m, 31H, 15 × CH_2_ and OH), 0.96–0.77 (m, 12H, 4 × CH_3_), 0.04 (s, 6H, 2 × CH_3_); ^13^C-NMR (50 MHz, CDCl_3_): *δ* 72.0, 63.3, 37.5, 32.9, 31.9, 29.7, 29.6, 29.6, 29.6 29.4, 29.3, 26.0, 25.8, 25.6, 22.7, 14.1, −5.3; HRMS (ESI^+^): *m*/*z* calcd for C_24_H_52_NaO_2_Si^+^: 423.3629; [M + Na]^+^ found: 423.3633.

18-((*tert*-Butyldimethylsilyl)oxy)octadecan-9-ol (**14b**) [20]. Colorless oil; Yield: 76%; ^1^H-NMR (400 MHz, CDCl_3_): *δ* 3.68–3.47 (m, 3H, CH_2_OTBDMS and C*H*OH), 1.53–1.39 (m, 7H, 3 × CH_2_ and OH), 1.33–1.23 (m, 24H, 12 × CH_2_), 0.90–0.86 (m, 12H, 4 × CH_3_), 0.04 (s, 6H, 2 × CH_3_); ^13^C-NMR (100 MHz, CDCl_3_): *δ* 72.0, 63.3, 37.5, 32.9, 31.9, 29.7, 29.7, 29.6, 29.6, 29.4, 29.3, 26.0, 25.8, 25.6, 22.7, 18.4, 14.1, −5.3; HRMS (ESI^+^): *m*/*z* calcd for C_24_H_52_NaO_2_Si^+^, 423.3629; [M + Na]^+^ found, 423.3630.

18-((*tert*-Butyldimethylsilyl)oxy)octadecan-8-ol (**14c**). Colorless oil; Yield: 71%; ^1^H-NMR (200 MHz, CDCl_3_): *δ* 3.62–3.52 (m, 3H, CH_2_OTBDMS and C*H*OH), 1.56–1.39 (m, 7H, 3 × CH_2_ and OH), 1.35–1.21 (m, 24H, 12 × CH_2_), 0.90–0.82 (m, 12H, 4 × CH_3_), 0.03 (s, 6H, 2 × CH_3_); ^13^C-NMR (50 MHz, CDCl_3_): *δ* 72.0, 63.3, 37.5, 32.8, 31.8, 29.7, 29.6, 29.4, 29.3, 25.9, 25.8, 25.6, 22.6, 18.3, 14.1, −5.3; HRMS (ESI^+^): *m*/*z* calcd for C_24_H_52_NaO_2_Si^+^: 423.3629; [M + Na]^+^ found: 423.3632.

18-((*tert*-Butyldimethylsilyl)oxy)octadecan-6-ol (**14d**). Colorless oil; Yield: 66%; ^1^H-NMR (400 MHz, CDCl_3_): *δ* 3.62–3.55 (m, 3H, CH_2_OTBDMS and C*H*OH), 1.52–1.40 (m, 7H, 3 × CH_2_ and OH), 1.36–1.21 (m, 24H, 12 × CH_2_), 0.91–0.86 (m, 12H, 4 × CH_3_), 0.04 (s, 6H, 2 × CH_3_); ^13^C-NMR (100 MHz, CDCl_3_): *δ* 72.0, 63.3, 37.5, 37.5, 32.9, 31.9, 29.7, 29.6, 29.6, 29.4, 26.0, 25.8, 25.7, 25.3, 22.6, 18.4, 14.0, −5.3; HRMS (ESI^+^): *m*/*z* calcd for C_24_H_52_NaO_2_Si^+^: 423.3629; [M + Na]^+^ found: 423.3646.

18-((*tert*-Butyldimethylsilyl)oxy)octadecan-5-ol (**14e**). Colorless oil; Yield: 70%; ^1^H-NMR (400 MHz, CDCl_3_): *δ* 3.63–3.50 (m, 3H, CH_2_OTBDMS and C*H*OH), 1.58–1.16 (m, 31H, 15 × CH_2_ and OH), 0.98–0.76 (m, 12H, 4 × CH_3_), 0.03 (s, 6H, 2 × CH_3_); ^13^C-NMR (100 MHz, CDCl_3_): *δ* 71.9, 63.3, 37.5, 37.2, 32.9, 29.7, 29.6, 29.4, 27.8, 26.0, 25.8, 25.7, 22.8, 18.4, 14.1, −5.3; HRMS (ESI^+^): *m*/*z* calcd for C_24_H_52_NaO_2_Si^+^: 423.3629; [M + Na]^+^ found: 423.3624.

### 3.4. General Procedure for the Coupling Reaction Between Alcohols and FAs

To a solution of the appropriate fatty acid (caproic or palmitic or oleic acid) (4.00 mmol) in dry CH_2_Cl_2_ (10 mL) at 0 °C, *N*-(3-dimethylaminopropyl)-*N*’-ethylcarbodiimide hydrochloride (767 mg, 4.00 mmol), triethylamine (0.56 mL, 4.00 mmol), and 4-dimethylaminopyridine (24 mg, 0.20 mmol) were added consecutively. The reaction mixture was left stirring at 0 °C for 10 min, and then a solution of the secondary alcohol (1.00 mmol) in dry CH_2_Cl_2_ (10 mL) was added. After 16 h, the reaction mixture was extracted with a saturated aqueous solution of NH_4_Cl (3 × 10 mL), and the combined organic layers were collected and dried with Na_2_SO_4_. The solvent was then removed under reduced pressure. The crude mixture was purified by flash silica chromatography (petroleum ether (bp 40–60 °C)/ethyl acetate, 90/10–80/20) to afford the desired ester as a colorless oil.

Methyl 5-(hexanoyloxy)octadecanoate (**6a**). Colorless oil; Yield: 77%; ^1^H-NMR (400 MHz, CDCl_3_): *δ* 4.96–4.81 (m, 1H, COOCH), 3.66 (s, 3H, OCH_3_), 2.40–2.21 (m, 4H, 2 × COCH_2_), 1.69–1.50 (m, 8H, 4 × CH_2_), 1.39–1.21 (m, 26H, 13 × CH_2_), 1.01–0.80 (m, 6H, 2 × CH_3_); ^13^C-NMR (100 MHz, CDCl_3_): *δ* 173.8, 173.6, 73.4, 51.5, 34.6, 34.0, 33.8, 33.5, 31.9, 31.3, 29.7, 29.6, 29.6, 29.5, 29.5, 29.5, 29.3, 25.3, 24.8, 22.7, 22.3, 20.7, 14.1, 13.9; HRMS (ESI^+^): *m*/*z* calcd for C_25_H_48_NaO_4_^+^: 435.3445; [M+Na]^+^ found: 435.3445.

Methyl 5-(palmitoyloxy)octadecanoate (**6b**). Colorless oil; Yield: 75%; ^1^H-NMR (400 MHz, CDCl_3_): *δ* 4.93–4.83 (m, 1H, COOCH), 3.66 (s, 3H, OCH_3_), 2.36–2.22 (m, 4H, 2 × COCH_2_), 1.70–1.48 (m, 8H, 4 × CH_2_), 1.34–1.19 (m, 46H, 23 × CH_2_), 0.87 (t, *J* = 6.8 Hz, 6H, 2 × CH_3_); ^13^C-NMR (100 MHz, CDCl_3_): *δ* 173.8, 173.6, 73.4, 51.5, 34.7, 34.0, 33.8, 33.5, 31.9, 29.7, 29.6, 29.6, 29.6, 29.6, 29.5, 29.5, 29.3, 29.3, 29.2, 25.3, 25.1, 22.7, 20.7, 14.1; HRMS (ESI^+^): *m*/*z* calcd for C_35_H_68_NaO_4_^+^: 575.5010; [M + Na]^+^ found: 575.5008.

1-Methoxy-1-oxooctadecan-5-yl oleate (**6c**). Colorless oil; Yield: 74%; ^1^H-NMR (400 MHz, CDCl_3_): *δ* 5.44–5.31 (m, 2H, 2 × =CH), 4.94–4.83 (m, 1H, COOCH), 3.66 (s, 3H, OCH_3_), 2.35–2.24 (m, 4H, 2 × COCH_2_), 2.07–1.94 (m, 4H, 2 × =CHC*H*_2_), 1.64–1.51 (m, 8H, 4 × CH_2_), 1.38–1.19 (m, 42H, 21 × CH_2_), 0.88 (t, *J* = 6.6 Hz, 6H, 2 × CH_3_); ^13^C-NMR (100 MHz, CDCl_3_): *δ* 173.8, 173.6, 130.0, 129.7, 73.4, 51.5, 34.6, 34.0, 33.8, 33.5, 31.9, 31.9, 29.8, 29.7, 29.7, 29.7, 29.6, 29.6, 29.5, 29.5, 29.5, 29.3, 29.3, 29.2, 29.2, 29.1, 27.2, 27.2, 25.3, 25.1, 22.7, 20.7, 14.1; HRMS (ESI^+^): *m*/*z* calcd for C_37_H_70_NaO_4_^+^: 601.5166; [M + Na]^+^ found: 601.5161.

Methyl 6-(hexanoyloxy)octadecanoate (**6d**). Colorless oil; Yield: 74%; ^1^H-NMR (400 MHz, CDCl_3_): *δ* 4.96–4.82 (m, 1H, COOCH), 3.68 (s, 3H, OCH_3_), 2.42–2.22 (m, 4H, 2 × COCH_2_), 1.68–1.61 (m, 4H, 2 × CH_2_), 1.59–1.48 (m, 4H, 2 × CH_2_), 1.42–1.24 (m, 26H, 13 × CH_2_), 0.96–0.86 (m, 6H, 2 × CH_3_); ^13^C-NMR (100 MHz, CDCl_3_): *δ* 174.0, 173.7, 73.8, 51.5, 34.7, 34.2, 34.0, 33.8, 31.9, 31.4, 29.7, 29.7, 29.6, 29. 6, 29.5, 29.4, 25.3, 24.9, 24.9, 22.7, 22.4, 14.1, 13.9; HRMS (ESI^+^): *m*/*z* calcd for C_25_H_48_NaO_4_^+^: 435.3445; [M + Na]^+^ found: 435.3444.

Methyl 6-(palmitoyloxy)octadecanoate (**6e**). Low melting point solid; Yield: 79%; ^1^H-NMR (400 MHz, CDCl_3_): *δ* 4.91–4.80 (m, 1H, COOCH), 3.65 (s, 3H, OCH_3_), 2.33–2.23 (m, 4H, 2 × COCH_2_), 1.65–1.57 (m, 4H, 2 × CH_2_), 1.57–1.46 (m, 4H, 2 × CH_2_), 1.35–1.21 (m, 46H, 23 × CH_2_), 0.87 (t, *J* = 6.7 Hz, 6H, 2 × CH_3_); ^13^C-NMR (100 MHz, CDCl_3_): *δ* 173.9, 173.6, 73.7, 51.4, 34.7, 34.1, 33.9, 33.8, 31.9, 29.7, 29.6, 29.6, 29.6, 29.5, 29.5, 29.3, 29.3, 29.2, 25.3, 25.1, 24.9, 24.8, 22.7, 14.1; HRMS (ESI^+^): *m*/*z* calcd for C_35_H_68_NaO_4_^+^: 575.5010; [M + Na]^+^ found: 575.5011.

1-Methoxy-1-oxooctadecan-6-yl oleate (**6f**). Colorless oil; Yield: 81%; ^1^H-NMR (400 MHz, CDCl_3_): *δ* 5.46–5.27 (m, 2H, 2 × =CH), 4.96–4.77 (m, 1H, COOCH), 3.66 (s, 3H, OCH_3_), 2.37–2.18 (m, 4H, 2 × COCH_2_), 2.14–1.88 (m, 4H, 2 × =CHC*H*_2_), 1.65–1.58 (m, 4H, 2 × CH_2_), 1.55–1.46 (m, 4H, 2 × CH_2_), 1.36–1.23 (m, 42H, 21 × CH_2_), 0.88 (t, *J* = 6.8 Hz, 6H, 2 × CH_3_); ^13^C-NMR (100 MHz, CDCl_3_): *δ* 174.0, 173.6, 130.0, 129.7, 73.7, 51.4, 34.7, 34.1, 33.9, 33.8, 31.9, 31.9, 29.8, 29.7, 29.7, 29.6, 29.6, 29.5, 29.5, 29.3, 29.3, 29.3, 29.2, 29.2, 29.1, 27.2, 27.2, 25.3, 25.1, 24.9, 24.8, 22.7, 14.1; HRMS (ESI^+^): *m*/*z* calcd for C_37_H_70_NaO_4_^+^: 601.5166; [M + Na]^+^ found: 601.5161.

Methyl 7-(hexanoyloxy)octadecanoate (**6g**). Colorless oil; Yield: 76%; ^1^H-NMR (400 MHz, CDCl_3_): *δ* 4.90–4.80 (m, 1H, COOCH), 3.65 (s, 3H, OCH_3_), 2.33–2.23 (m, 4H, 2 × COCH_2_), 1.65–1.57 (m, 4H, 2 × CH_2_), 1.55–1.45 (m, 4H, 2 × CH_2_), 1.35–1.20 (m, 26H, 13 × CH_2_), 0.94–0.84 (m, 6H, 2 × CH_3_); ^13^C-NMR (100 MHz, CDCl_3_): *δ* 174.1, 173.7, 73.8, 51.4, 34.6, 34.1, 33.9, 31.9, 31.3, 29.6, 29.6, 29.5, 29.5, 29.5, 29.3, 29.0, 25.3, 25.0, 24.8, 24.8, 22.7, 22.3, 14.1, 13.9; HRMS (ESI^+^): *m*/*z* calcd for C_25_H_48_NaO_4_^+^: 435.3445; [M + Na]^+^ found: 435.3445.

Methyl 7-(palmitoyloxy)octadecanoate (**6h**). Off white low melting point solid; Yield: 77%; ^1^H-NMR (200 MHz, CDCl_3_): *δ* 4.91–4.79 (m, 1H, COOCH), 3.65 (s, 3H, OCH_3_), 2.33–2.21 (m, 4H, 2 × COCH_2_), 1.69–1.46 (m, 8H, 4 × CH_2_), 1.46–1.05 (m, 46H, 23 × CH_2_), 0.86 (t, *J* = 6.4 Hz, 6H, 2 × CH_3_); ^13^C-NMR (50 MHz, CDCl_3_): *δ* 174.1, 173.7, 73.8, 51.4, 34.7, 34.1, 33.9, 31.9, 29.7, 29.6, 29.6, 29.6, 29.5, 29.5, 29.3, 29.3, 29.2, 29.0, 25.3, 25.2, 25.0, 24.8, 22.7, 14.1; HRMS (ESI^+^): *m*/*z* calcd for C_35_H_68_NaO_4_^+^: 575.5010; [M + Na]^+^ found: 575.5020.

1-Methoxy-1-oxooctadecan-7-yl oleate (**6i**). Colorless oil; Yield: 81%; ^1^H-NMR (400 MHz, CDCl_3_): *δ* 5.43–5.26 (m, 2H, 2 × =CH), 4.89–4.81 (m, 1H, COOCH), 3.65 (s, 3H, OCH_3_), 2.31–2.24 (m, 4H, 2 × COCH_2_), 2.11–1.88 (m, 4H, 2 × =CHC*H*_2_), 1.64–1.56 (m, 4H, 2 × CH_2_), 1.54–1.45 (m, 4H, 2 × CH_2_), 1.36–1.20 (m, 42H, 21 × CH_2_), 0.87 (t, *J* = 6.8 Hz, 6H, 2 × CH_3_); ^13^C-NMR (100 MHz, CDCl_3_): *δ* 174.1, 173.6, 129.9, 129.7, 73.8, 51.4, 34.7, 34.1, 33.9, 31.9, 31.9, 29.7, 29.7, 29.6, 29.6, 29.6, 29.5, 29.5, 29.3, 29.3, 29.2, 29.1, 29.1, 29.0, 27.2, 27.1, 25.3, 25.1, 25.0, 24.8, 22.7, 14.1; HRMS (ESI^+^): *m*/*z* calcd for C_37_H_70_NaO_4_^+^: 601.5166; [M + Na]^+^ found: 601.5165.

Methyl 8-(hexanoyloxy)octadecanoate (**6j**). Colorless oil; Yield: 82%; ^1^H-NMR (400 MHz, CDCl_3_): *δ* 4.92–4.79 (m, 1H, COOCH), 3.65 (s, 3H, OCH_3_), 2.35–2.21 (m, 4H, 2 × COCH_2_), 1.65–1.58 (m, 4H, 2 × CH_2_), 1.54–1.46 (m, 4H, 2 × CH_2_), 1.36–1.21 (m, 26H, 13 × CH_2_), 0.96–0.83 (m, 6H, 2 × CH_3_); ^13^C-NMR (100 MHz, CDCl_3_): *δ* 174.2, 173.6, 73.9, 51.4, 34.7, 34.1, 34.1, 34.0, 31.9, 31.3, 29.6, 29.5, 29.5, 29.3, 29.1, 29.0, 25.3, 25.1, 24.8, 22.7, 22.3, 14.1, 13.9; HRMS (ESI^+^): *m*/*z* calcd for C_25_H_48_NaO_4_^+^: 435.3445; [M + Na]^+^ found: 435.3457.

Methyl 8-(palmitoyloxy)octadecanoate (**6k**). Colorless oil; Yield: 85%; ^1^H-NMR (400 MHz, CDCl_3_): *δ* 4.92–4.79 (m, 1H, COOCH), 3.66 (s, 3H, OCH_3_), 2.32–2.23 (m, 4H, 2 × COCH_2_), 1.65–1.57 (m, 4H, 2 × CH_2_), 1.55–1.45 (m, 4H, 2 × CH_2_), 1.35–1.21 (m, 46H, 23 × CH_2_), 0.87 (t, *J* = 6.7 Hz, 6H, 2 × CH_3_); ^13^C-NMR (100 MHz, CDCl_3_): *δ* 174.2, 173.7, 73.9, 51.4, 34.7, 34.2, 34.1, 34.0, 31.9, 31.9, 29.7, 29.6, 29.6, 29.6, 29.6, 29.5, 29.5, 29.3, 29.3, 29.3, 29.2, 29.1, 29.0, 25.3, 25.2, 25.1, 24.9, 22.7, 14.1; HRMS (ESI^+^): *m*/*z* calcd for C_35_H_68_NaO_4_^+^: 575.5010; [M + Na]^+^ found: 575.5009.

1-Methoxy-1-oxooctadecan-8-yl oleate (**6l**). Colorless oil; Yield: 84%; ^1^H-NMR (400 MHz, CDCl_3_): *δ* 5.45–5.26 (m, 2H, 2 × =CH), 4.93–4.79 (m, 1H, COOCH), 3.65 (s, 3H, OCH_3_), 2.34–2.22 (m, 4H, 2 × COCH_2_), 2.10–1.88 (m, 4H, 2 × =CHC*H*_2_), 1.67–1.57 (m, 4H, 2 × CH_2_), 1.54–1.43 (m, 4H, 2 × CH_2_), 1.39–1.11 (m, 42H, 21 × CH_2_), 0.87 (t, *J* = 6.7 Hz, 6H, 2 × CH_3_); ^13^C-NMR (100 MHz, CDCl_3_): *δ* 174.2, 173.6, 130.0, 129.7, 73.9, 51.4, 34.7, 34.1, 34.07, 34.0, 31.9, 29.7, 29.7, 29.6, 29.6, 29.6, 29.5, 29.5, 29.3, 29.2, 29.1, 29.1, 29.0, 27.2, 27.2, 25.3, 25.1, 24.8, 22.7, 14.1; HRMS (ESI^+^): *m*/*z* calcd for C_37_H_70_NaO_4_^+^: 601.5166; [M + Na]^+^ found: 601.5162.

Methyl 9-(palmitoyloxy)octadecanoate (**6m**) [42]. Colorless oil; Yield: 73%; ^1^H-NMR (400 MHz, CDCl_3_): *δ* 4.96–4.75 (m, 1H, COOCH), 3.65 (s, 3H, OCH_3_), 2.34–2.22 (m, 4H, 2 × COCH_2_), 1.64–1.57 (m, 4H, 2 × CH_2_), 1.57–1.45 (m, 4H, 2 × CH_2_), 1.35–1.22 (m, 46H, 23 × CH_2_), 0.87 (t, *J* = 6.7 Hz, 6H, 2 × CH_3_); ^13^C-NMR (100 MHz, CDCl_3_): *δ* 174.2, 173.7, 74.0, 51.4, 34.7, 34.2, 34.1, 34.1, 31.9, 31.9, 29.7, 29.6, 29.6, 29.5, 29.5, 29.5, 29.3, 29.3, 29.3, 29.2, 29.1, 29.0, 25.3, 25.2, 25.2, 24.9, 22.7, 22.7, 14.1; HRMS (ESI^+^): *m*/*z* calcd for C_35_H_68_NaO_4_^+^: 575.5010; [M + Na]^+^ found: 575.5010.

Methyl 12-(hexanoyloxy)octadecanoate (**6n**). Colorless oil; Yield: 75%; ^1^H-NMR (400 MHz, CDCl_3_): *δ* 4.90–4.80 (m, 1H, COOCH), 3.64 (s, 3H, OCH_3_), 2.33–2.22 (m, 4H, 2 × COCH_2_), 1.67–1.54 (m, 4H, 2 × CH_2_), 1.54–1.42 (m, 4H, 2 × CH_2_), 1.40–1.15 (m, 26H, 13 × CH_2_), 0.91–0.82 (m, 6H, 2 × CH_3_); ^13^C-NMR (100 MHz, CDCl_3_): *δ* 174.2, 173.6, 74.0, 51.3, 34.6, 34.1, 34.0, 31.7, 31.3, 29.5, 29.5, 29.4, 29.4, 29.2, 29.1, 29.1, 25.3, 25.2, 24.9, 24.8, 22.5, 22.3, 14.0, 13.8; HRMS (ESI^+^): *m*/*z* calcd for C_25_H_48_NaO_4_^+^: 435.3445; [M + Na]^+^ found: 435.3446.

Methyl 12-(palmitoyloxy)octadecanoate (**6o**) [56]. Colorless oil; Yield: 77%; ^1^H-NMR (400 MHz, CDCl_3_): *δ* 4.91–4.81 (m, 1H, COOCH), 3.65 (s, 3H, OCH_3_), 2.35–2.23 (m, 4H, 2 × COCH_2_), 1.64–1.56 (m, 4H, 2 × CH_2_), 1.53–1.45 (m, 4H, 2 × CH_2_), 1.38–1.21 (m, 46H, 23 × CH_2_), 0.86 (t, *J* = 6.2 Hz, 6H, 2 × CH_3_); ^13^C-NMR (100 MHz, CDCl_3_): *δ* 174.2, 173.6, 74.0, 51.3, 34.7, 34.1, 34.1, 31.9, 31.7, 29.7, 29.7, 29.6, 29.6, 29.5, 29.5, 29.5, 29.4, 29.3, 29.3, 29.2, 29.2, 29.1, 25.3, 25.3, 25.2, 24.9, 22.7, 22.5, 14.1, 14.0; HRMS (ESI^+^): *m*/*z* calcd for C_35_H_68_NaO_4_^+^: 575.5010; [M + Na]^+^ found: 575.5024.

18-Methoxy-18-oxooctadecan-7-yl oleate (**6p**). Colorless oil; Yield: 76%; ^1^H-NMR (400 MHz, CDCl_3_): *δ* 5.40–5.27 (m, 2H, 2 × =CH), 4.90–4.81 (m, 1H, COOCH), 3.64 (s, 3H, OCH_3_), 2.31–2.22 (m, 4H, 2 × COCH_2_), 2.06–1.90 (m, 4H, 2 × =CHC*H*_2_), 1.64–1.56 (m, 4H, 2 × CH_2_), 1.54–1.43 (m, 4H, 2 × CH_2_), 1.37–1.17 (m, 42H, 21 × CH_2_), 0.86 (t, *J* = 6.1 Hz, 6H, 2 × CH_3_); ^13^C-NMR (100 MHz, CDCl_3_): *δ* 174.2, 173.6, 129.9, 129.7, 74.0, 51.3, 34.7, 34.1, 34.1, 31.9, 31.7, 29.7, 29.7, 29.6, 29.6, 29.5, 29.5, 29.5, 29.4, 29.3, 29.2, 29.2, 29.1, 29.1, 27.2, 27.1, 25.3, 25.2, 25.1, 24.9, 22.6, 22.5, 14.0, 14.0; HRMS (ESI^+^): *m*/*z* calcd for C_37_H_70_NaO_4_^+^: 601.5166; [M + Na]^+^ found: 601.5164.

1-((*tert*-Βutyldimethylsilyl)oxy)octadecan-9-yl hexanoate (**15a**). Colorless oil; Yield: 77%; ^1^H-NMR (400 MHz, CDCl_3_): *δ* 4.90–4.83 (m, 1H, COOCH), 3.59 (t, *J* = 6.6 Hz, 2H, CH_2_OTBDMS), 2.27 (t, *J* = 7.5 Hz, 2H, CH_2_COO),1.64–159 (m, 2H, CH_2_), 1.53–1.46 (m, 6H, 3 × CH_2_), 1.32–1.23 (m, 28H, 14 × CH_2_), 0.91–0.85 (m, 15H, 5 × CH_3_), 0.04 (s, 6H, 2 × CH_3_); ^13^C-NMR (100 MHz, CDCl_3_): *δ* 173.7, 74.1, 63.3, 34.7, 34.2, 32.9, 31.9, 31.4, 29.5, 29.5, 29.5, 29.4, 29.3, 26.0, 25.8, 25.3, 24.9, 22.7, 22.3, 18.4, 14.1, 13.9, −5.3; HRMS (ESI^+^): *m*/*z* calcd for C_30_H_62_NaO_3_Si^+^: 521.4360; [M + Na]^+^ found: 521.4364.

1-((*tert*-Butyldimethylsilyl)oxy)octadecan-9-yl oleate (**15b**) [22]. Colorless oil; Yield: 84%; ^1^H-NMR (400 MHz, CDCl_3_): *δ* 5.39–5.30 (m, 2H, 2 × =CH), 4.90–4.82 (m, 1H, COOCH), 3.59 (t, *J* = 7.5 Hz, 2H, CH_2_OTBDMS), 2.27 (t, *J* = 7.5 Hz, 2H, CH_2_COO), 2.04–1.93 (m, 4H, 2 × =CHC*H*_2_), 1.66–1.55 (m, 4H, 2 × CH_2_), 1.53–1.47 (m, 6H, 3 × CH_2_), 1.34–1.25 (m, 42H, 21 × CH_2_), 0.90–0.85 (m, 15H, 5 × CH_3_), 0.04 (s, 6H, 2 × CH_3_); ^13^C-NMR (100 MHz, CDCl_3_): *δ* 173.6, 130.0, 129.7, 74.1, 63.3, 34.7, 34.2, 32.9, 31.9, 29.8, 29.7, 29.7, 29.6, 29.5, 29.5, 29.4, 29.3, 29.3, 29.2, 29.2, 29.1, 27.2, 27.2, 26.0, 25.8, 25.3, 25.2, 22.7, 18.4, 14.1, −5.3; HRMS (ESI^+^): *m*/*z* calcd for C_42_H_84_NaO_3_Si^+^: 687.6082; [M + Na]^+^ found: 687.6095.

18-((*tert*-Butyldimethylsilyl)oxy)octadecan-9-yl hexanoate (**15c**). Colorless oil; Yield: 79%; ^1^H-NMR (400 MHz, CDCl_3_): *δ* 4.94–4.76 (m, 1H, COOCH), 3.59 (t, *J* = 6.6 Hz, 2H, CH_2_OTBDMS), 2.27 (t, *J* = 7.5 Hz, 2H, CH_2_COO), 1.66–1.47 (m, 8H, 4 × CH_2_), 1.33–1.23 (m, 28H, 14 × CH_2_), 0.94–0.82 (m, 15H, 5 × CH_3_), 0.04 (s, 6H, 2 × CH_3_); ^13^C-NMR (100 MHz, CDCl_3_): *δ* 173.7, 74.1, 63.3, 34.7, 34.2, 32.9, 31.9, 31.3, 29.7, 29.5, 29.5, 29.5, 29.4, 29.2, 26.0, 25.8, 25.3, 24.9, 22.6, 22.3, 18.4, 14.1, 13.9, −5.3; HRMS (ESI^+^): *m*/*z* calcd for C_30_H_62_NaO_3_Si^+^: 521.4360; [M + Na]^+^ found: 521.4371.

18-((*tert*-Butyldimethylsilyl)oxy)octadecan-9-yl palmitate (**15d**). Colorless oil; Yield: 84%; ^1^H-NMR (400 MHz, CDCl_3_): *δ* 4.94–4.77 (m, 1H, COOCH), 3.59 (t, *J* = 6.6 Hz, 2H, CH_2_OTBDMS), 2.27 (t, *J* = 7.4 Hz, 2H, CH_2_COO), 1.65–1.59 (m, 2H, CH_2_), 1.54–1.44 (m, 6H, 3 × CH_2_), 1.35–1.21 (m, 48H, 24 × CH_2_), 0.97–0.79 (m, 15H, 5 × CH_3_), 0.05 (s, 6H, 2 × CH_3_); ^13^C-NMR (100 MHz, CDCl_3_): *δ* 173.7, 74.1, 63.3, 34.8, 34.2, 32.9, 31.9, 31.9, 29.7, 29.7, 29.6, 29.6, 29.5, 29.5, 29.5, 29.4, 29.4, 29.3, 29.2, 29.2, 26.0, 25.8, 25.3, 25.2, 22.7, 22.7, 18.4, 14.1, −5.3; HRMS (ESI^+^): *m*/*z* calcd for C_40_H_82_NaO_3_Si^+^: 661.5925; [M + Na]^+^ found: 661.5921.

18-((*tert*-Butyldimethylsilyl)oxy)octadecan-9-yl oleate (**15e**) [20]. Colorless oil; Yield: 83%; ^1^H-NMR (400 MHz, CDCl_3_): *δ* 5.49–5.23 (m, 2H, 2 × =CH), 4.93–4.79 (m, 1H, COOCH), 3.59 (t, *J* = 6.6 Hz, 2H, CH_2_OTBDMS), 2.27 (t, *J* = 7.5 Hz, 2H, CH_2_COO), 2.12–1.88 (m, 4H, 2 × =CHC*H*_2_), 1.68–1.55 (m, 4H, 2 × CH_2_), 1.53–1.45 (m, 6H, 3 × CH_2_), 1.35–1.23 (m, 42H, 21 × CH_2_), 0.97–0.80 (m, 15H, 5 × CH_3_), 0.04 (s, 6H, 2 × CH_3_); ^13^C-NMR (100 MHz, CDCl_3_): *δ* 173.7, 130.0, 129.7, 74.1, 63.3, 34.7, 34.2, 32.9, 31.9, 31.9, 29.8, 29.7, 29.7, 29.6, 29.5, 29.5, 29.5, 29.5, 29.4, 29.3, 29.3, 29.2, 29.2, 29.2, 29.1, 27.2, 27.2, 26.0, 25.8, 25.3, 25.2, 22.7, 22.7, 18.4, 14.1, 14.1, −5.3; HRMS (ESI^+^): *m*/*z* calcd for C_42_H_84_NaO_3_Si^+^: 687.6082; [M + Na]^+^ found: 687.6080.

18-((*tert*-Butyldimethylsilyl)oxy)octadecan-8-yl hexanoate (**15f**). Colorless oil; Yield: 79%; ^1^H-NMR (400 MHz, CDCl_3_): *δ* 4.95–4.78 (m, 1H, COOCH), 3.59 (t, *J* = 6.6 Hz, 2H, CH_2_OTBDMS), 2.28 (t, *J* = 7.5 Hz, 2H, CH_2_COO), 1.67–1.60 (m, 2H, CH_2_), 1.55–1.45 (m, 6H, 3 × CH_2_), 1.34–1.23 (m, 28H, 14 × CH_2_), 0.93–0.83 (m, 15H, 5 × CH_3_), 0.04 (s, 6H, 2 × CH_3_); ^13^C-NMR (100 MHz, CDCl_3_): *δ* 173.7, 74.1, 63.3, 34.7, 34.2, 34.1, 32.9, 31.7, 31.4, 29.6, 29.6, 29.6, 29.6, 29.5, 29.4, 26.0, 25.8, 25.3, 25.0, 24.9, 22.5, 22.3, 18.4, 14.0, 13.9, −5.3; HRMS (ESI^+^): *m*/*z* calcd for C_30_H_62_NaO_3_Si^+^: 521.4360; [M + Na]^+^ found: 521.4374.

18-((*tert*-Butyldimethylsilyl)oxy)octadecan-8-yl palmitate (**15g**). Colorless oil; Yield: 74%; ^1^H-NMR (400 MHz, CDCl_3_): *δ* 4.91–4.79 (m, 1H, COOCH), 3.59 (t, *J* = 6.6 Hz, 2H, CH_2_OTBDMS), 2.27 (t, *J* = 7.5 Hz, 2H, CH_2_COO), 1.65–1.59 (m, 2H, CH_2_), 1.55–1.45 (m, 6H, 3 × CH_2_), 1.32–1.22 (m, 48H, 24 × CH_2_), 0.92–0.85 (m, 15H, 5 × CH_3_), 0.04 (s, 6H, 2 × CH_3_); ^13^C-NMR (100 MHz, CDCl_3_): *δ* 173.7, 74.1, 63.3, 34.8, 34.2, 32.9, 31.9, 31.8, 29.7, 29.7, 29.6, 29.5, 29.5, 29.4, 29.4, 29.3, 29.2, 29.2, 26.0, 25.8, 25.3, 25.2, 22.7, 22.6, 14.1, 14.1, −5.3; HRMS (ESI^+^): *m*/*z* calcd for C_40_H_82_NaO_3_Si^+^: 661.5925; [M + Na]^+^ found: 661.5958.

18-((*tert*-Butyldimethylsilyl)oxy)octadecan-8-yl oleate (**15h**). Colorless oil; Yield: 82%; ^1^H-NMR (400 MHz, CDCl_3_): *δ* 5.39–5.28 (m, 2H, 2 × =CH), 4.90–4.83 (m, 1H, COOCH), 3.59 (t, *J* = 6.5 Hz, 2H, CH_2_OTBDMS), 2.27 (t, *J* = 7.4 Hz, 2H, CH_2_COO), 2.06–1.94 (m, 4H, 2 × =CHC*H*_2_), 1.67–1.56 (m, 4H, 2 × CH_2_), 1.53–1.47 (m, 6H, 3 × CH_2_), 1.33–1.24 (m, 42H, 21 × CH_2_), 0.90–0.85 (m, 15H, 5 × CH_3_), 0.04 (s, 6H, 2 × CH_3_); ^13^C-NMR (100 MHz, CDCl_3_): *δ* 173.7, 130.0, 129.8, 74.1, 63.3, 34.7, 34.2, 32.9, 31.9, 31.8, 29.8, 29.7, 29.7, 29.6, 29.6, 29.5, 29.50, 29.4, 29.3, 29.2, 29.2, 29.1, 27.2, 27.2, 26.0, 25.8, 25.3, 25.2, 22.7, 22.6, 18.4, 14.1, 14.1, −5.3; HRMS (ESI^+^): *m*/*z* calcd for C_42_H_84_NaO_3_Si^+^: 687.6082; [M + Na]^+^ found: 687.6109.

18-((*tert*-Butyldimethylsilyl)oxy)octadecan-6-yl hexanoate (**15i**). Colorless oil; Yield: 72%; ^1^H-NMR (400 MHz, CDCl_3_): *δ* 4.90–4.83 (m, 1H, COOCH), 3.59 (t, *J* = 6.6 Hz, 2H, CH_2_OTBDMS), 2.27 (t, *J* = 7.5 Hz, 2H, CH_2_COO), 1.66–1.58 (m, 2H, CH_2_), 1.55–1.45 (m, 6H, 3 × CH_2_), 1.34–1.22 (m, 28H, 14 × CH_2_), 0.92–0.84 (m, 15H, 5 × CH_3_), 0.04 (s, 6H, 2 × CH_3_); ^13^C-NMR (100 MHz, CDCl_3_): *δ* 173.7, 74.1, 63.3, 34.7, 34.2, 34.1, 32.9, 31.7, 31.4, 29.6, 29.6, 29.6, 29.6, 29.5, 29.4, 26.0, 25.8, 25.3, 25.0, 24.9, 22.5, 22.3, 18.4, 14.0, 13.9, −5.3; HRMS (ESI^+^): *m*/*z* calcd for C_30_H_62_NaO_3_Si^+^: 521.4360; [M + Na]^+^ found: 521.4374.

18-((*tert*-Butyldimethylsilyl)oxy)octadecan-6-yl palmitate (**15j**). Colorless oil; Yield: 76%; ^1^H-NMR (400 MHz, CDCl_3_): *δ* 4.91–4.81 (m, 1H, COOCH), 3.59 (t, *J* = 6.6 Hz, 2H, CH_2_OTBDMS), 2.27 (t, *J* = 7.5 Hz, 2H, CH_2_COO), 1.66–1.58 (m, 2H, CH_2_), 1.55–1.45 (m, 6H, 3 × CH_2_), 1.34–1.19 (m, 48H, 24 × CH_2_), 0.92–0.84 (m, 15H, 5 × CH_3_), 0.04 (s, 6H, 2 × CH_3_); ^13^C-NMR (100 MHz, CDCl_3_): *δ* 173.7, 74.0, 63.3, 34.7, 34.2, 34.1, 32.9, 31.9, 31.7, 29.7, 29.7, 29.6, 29.6, 29.6, 29.6, 29.5, 29.5, 29.4, 29.3, 29.2, 26.0, 25.8, 25.3, 25.2, 25.0, 22.7, 22.5, 18.4, 14.1, 14.0, −5.3; HRMS (ESI^+^): *m*/*z* calcd for C_40_H_82_NaO_3_Si^+^: 661.5925; [M + Na]^+^ found: 661.5949.

18-((*tert*-Butyldimethylsilyl)oxy)octadecan-6-yl oleate (**15k**). Colorless oil; Yield: 81%; ^1^H-NMR (400 MHz, CDCl_3_): *δ* 5.47–5.25 (m, 2H, 2 × =CH), 4.92–4.83 (m, 1H, COOCH), 3.59 (t, *J* = 6.6 Hz, 2H, CH_2_OTBDMS), 2.27 (t, *J* = 7.5 Hz, 2H, CH_2_COO), 2.11–1.89 (m, 4H, 2 × =CHC*H*_2_), 1.65–1.59 (m, 2H, CH_2_), 1.55–1.46 (m, 6H, 3 × CH_2_), 1.37–1.22 (m, 44H, 22 × CH_2_), 0.92–0.83 (m, 15H, 5 × CH_3_), 0.04 (s, 6H, 2 × CH_3_); ^13^C-NMR (100 MHz, CDCl_3_): *δ* 173.7, 130.0, 129.7, 74.1, 63.3, 34.7, 34.2, 34.1, 32.9, 31.9, 31.7, 29.8, 29.7, 29.6, 29.6, 29.6, 29.6, 29.6, 29.5, 29.5, 29.3, 29.2, 29.2, 29.1, 27.2, 27.2, 26.0, 25.8, 25.3, 25.2, 25.0, 22.7, 22.6, 22.5, 18.4, 14.1, 14.0, −5.3; HRMS (ESI^+^): *m*/*z* calcd for C_42_H_84_NaO_3_Si^+^: 687.6082; [M + Na]^+^ found: 687.6099.

18-((*tert*-Βutyldimethylsilyl)oxy)octadecan-5-yl hexanoate (**15l**). Colorless oil; Yield: 80%; ^1^H-NMR (400 MHz, CDCl_3_): *δ* 4.90–4.83 (m, 1H, COOCH), 3.59 (t, *J* = 6.6 Hz, 2H, CH_2_OTBDMS), 2.27 (t, *J* = 7.5 Hz, 2H, CH_2_COO), 1.66–1.59 (m, 2H, CH_2_), 1.56–1.45 (m, 6H, 3 × CH_2_), 1.36–1.23 (m, 28H, 14 × CH_2_), 0.94–0.85 (m, 15H, 5 × CH_3_), 0.04 (s, 6H, 2 × CH_3_); ^13^C-NMR (100 MHz, CDCl_3_): *δ* 173.7, 74.1, 63.3, 34.7, 34.2, 33.9, 32.9, 31.4, 29.6, 29.6, 29.6, 29.6, 29.5, 27.5, 26.0, 25.8, 25.3, 24.9, 22.6, 22.3, 18.4, 14.0, 13.9, −5.3; HRMS (ESI^+^): *m*/*z* calcd for C_30_H_62_NaO_3_Si^+^: 521.4360; [M + Na]^+^ found: 521.4363.

18-((*tert*-Butyldimethylsilyl)oxy)octadecan-5-yl palmitate (**15m**). Colorless oil; Yield: 80%; ^1^H-NMR (400 MHz, CDCl_3_): *δ* 4.94–4.81 (m, 1H, COOCH), 3.59 (t, *J* = 6.6 Hz, 2H, CH_2_OTBDMS), 2.27 (t, *J* = 7.5 Hz, 2H, CH_2_COO), 1.64–1.59 (m, 2H, CH_2_), 1.55–1.46 (m, 6H, 3 × CH_2_), 1.37–1.21 (m, 48H, 24 × CH_2_), 0.98–0.77 (m, 15H, 5 × CH_3_), 0.04 (s, 6H, 2 × CH_3_); ^13^C-NMR (100 MHz, CDCl_3_): *δ* 173.7, 74.0, 63.3, 34.7, 34.2, 33.9, 32.9, 31.9, 29.7, 29.7, 29.7, 29.6, 29.6, 29.6, 29.5, 29.5, 29.4, 29.3, 29.2, 27.5, 26.0, 25.8, 25.3, 25.2, 22.7, 22.6, 18.4, 14.1, 14.0, −5.3; HRMS (ESI^+^): *m*/*z* calcd for C_40_H_82_NaO_3_Si^+^: 661.5925; [M + Na]^+^ found: 661.5939.

18-((*tert*-Butyldimethylsilyl)oxy)octadecan-5-yl oleate (**15n**). Colorless oil; Yield: 82%; ^1^H-NMR (400 MHz, CDCl_3_): *δ* 5.40–5.29 (m, 2H, 2 × =CH), 4.91–4.83 (m, 1H, COOCH), 3.59 (t, *J* = 6.6 Hz, 2H, CH_2_OTBDMS), 2.27 (t, *J* = 7.4 Hz, 2H, CH_2_COO), 2.08–1.92 (m, 4H, 2 × =CHC*H*_2_), 1.64–1.48 (m, 8H, 4 × CH_2_), 1.35–1.22 (m, 44H, 22 × CH_2_), 0.92–0.84 (m, 15H, 5 × CH_3_), 0.04 (s, 6H, 2 × CH_3_); ^13^C-NMR (100 MHz, CDCl_3_): *δ* 173.6, 130.0, 129.7, 74.1, 63.3, 34.7, 34.2, 33.9, 32.9, 31.9, 29.8, 29.7, 29.7, 29.6, 29.6, 29.6, 29.5, 29.5, 29.3, 29.3, 29.2, 29.2, 29.1, 29.0, 27.5, 27.2, 27.2, 26.0, 25.8, 25.3, 25.2, 22.7, 22.6, 18.4, 14.1, 14.0, −5.3; HRMS (ESI^+^): *m*/*z* calcd for C_42_H_84_NaO_3_Si^+^: 687.6082; [M + Na]^+^ found: 687.6099.

### 3.5. General Procedure for the Synthesis of FAHFAs Through the Saponification of Esters

To a round-bottomed flask containing methyl esters **6a**–**p** (1.00 mmol) diluted in a mixture of THF and H_2_O (3:1, 10 mL), LiOH_·_H_2_O (210 mg, 5.00 mmol) was added, and the reaction mixture was left stirring at room temperature for 16 h. The reaction was then quenched with H_2_O, followed by the addition of HCl 1 N (10 mL) to pH 1. Then, the mixture was extracted with EtOAc (3 × 10 mL), washed with brine (1 × 30 mL), dried over Na_2_SO_4_, filtered, and concentrated under reduced pressure. Flash chromatography on silica gel of the crude mixture (petroleum ether (bp 40–60 °C)/ethyl acetate, 60/40) resulted in the desired FAHFA.

5-(Hexanoyloxy)octadecanoic acid (**7a**). White solid; Yield: 35%; m. p.: 39–40 °C; ^1^H-NMR (400 MHz, CDCl_3_): *δ* 4.97–4.82 (m, 1H, COOCH), 2.36 (t, *J* = 7.5 Hz, 2H, COCH_2_), 2.28 (t, *J* = 7.5 Hz, 2H, COCH_2_), 1.68–1.49 (m, 8H, 4 × CH_2_), 1.39–1.23 (m, 26H, 13 × CH_2_), 1.01–0.81 (m, 6H, 2 × CH_3_); ^13^C-NMR (100 MHz, CDCl_3_): *δ* 178.2, 173.7, 73.4, 34.6, 34.1, 33.5, 33.4, 31.9, 31.3, 29.7, 29.7, 29.6, 29.6, 29.5, 29.5, 29.4, 25.3, 24.8, 22.7, 22.3, 20.5, 14.1, 13.9; HRMS (ESI^+^): *m*/*z* calcd for C_24_H_46_NaO_4_^+^: 421.3288; [M + Na]^+^ found: 421.3287.

5-(Palmitoyloxy)octadecanoic acid (**7b**) [21]. White solid; Yield: 42%; m. p.: 39–40 °C, (lit. m.p.: 39–40 °C); ^1^H-NMR (400 MHz, CDCl_3_): *δ* 4.97–4.81 (m, 1H, COOCH), 2.36 (t, *J* = 7.5 Hz, 2H, COCH_2_), 2.28 (t, *J* = 7.5 Hz, 2H, COCH_2_), 1.72–1.45 (m, 8H, 4 × CH_2_), 1.36–1.19 (m, 46H, 23 × CH_2_), 0.88 (t, *J* = 6.7 Hz, 6H, 2 × CH_3_); ^13^C-NMR (100 MHz, CDCl_3_): *δ* 179.2, 173.7, 73.4, 34.7, 34.1, 33.7, 33.4, 31.9, 29.7, 29.7, 29.7, 29.6, 29.6, 29.5, 29.5, 29.4, 29.4, 29.3, 29.2, 29.2, 29.1, 25.3, 25.1, 24.7, 22.7, 20.4, 14.1; HRMS (ESI^+^): *m*/*z* calcd for C_34_H_66_NaO_4_^+^: 561.4853; [M + Na]^+^ found: 561.4853.

5-(Oleoyloxy)octadecanoic acid (**7c**). Colorless oil; Yield: 41%; ^1^H-NMR (400 MHz, CDCl_3_): *δ* 5.46–5.23 (m, 2H, 2 × =CH), 4.97–4.81 (m, 1H, COOCH), 2.36 (t, *J* = 7.5 Hz, 2H, COCH_2_), 2.28 (t, *J* = 7.5 Hz, 2H, COCH_2_), 2.12–1.89 (m, 4H, 2 × =CHC*H*_2_), 1.70–1.48 (m, 8H, 4 × CH_2_), 1.40–1.09 (m, 42H, 21 × CH_2_), 0.88 (t, *J* = 6.7 Hz, 6H, 2 × CH_3_); ^13^C-NMR (100 MHz, CDCl_3_): *δ* 178.4, 173.7, 130.0, 129.8, 73.4, 34.7, 34.1, 33.4, 31.9, 31.9, 29.8, 29.7, 29.7, 29.7, 29.6, 29.5, 29.5, 29.5, 29.4, 29.3, 29.3, 29.2, 29.2, 29.1, 27.2, 27.2, 25.3, 25.1, 22.7, 20.5, 14.1; HRMS (ESI^+^): *m*/*z* calcd for C_36_H_68_NaO_4_^+^: 587.5010; [M + Na]^+^ found: 587.5008.

6-(Hexanoyloxy)octadecanoic acid (**7d**). Colorless oil; Yield: 39%; ^1^H-NMR (400 MHz, CDCl_3_): *δ* 4.92–4.83 (m, 1H, COOCH), 2.34 (t, *J* = 7.5 Hz, 2H, COCH_2_), 2.28 (t, *J* = 7.5 Hz, 2H, COCH_2_), 1.72–1.58 (m, 4H, 2 × CH_2_), 1.58–1.45 (m, 4H, 2 × CH_2_), 1.40–1.21 (m, 26H, 13 × CH_2_), 0.94–0.84 (m, 6H, 2 × CH_3_); ^13^C-NMR (100 MHz, CDCl_3_): *δ* 178.8, 173.7, 73.7, 34.7, 34.1, 33.8, 33.7, 31.9, 31.3, 29.7, 29.6, 29.6, 29.5, 29.5, 29.3, 25.3, 24.8, 24.5, 22.7, 22.3, 14.1, 13.9; HRMS (ESI^+^): *m*/*z* calcd for C_24_H_46_NaO_4_^+^: 421.3288; [M + Na]^+^ found: 421.3286.

6-(Palmitoyloxy)octadecanoic acid (**7e**). White solid; Yield: 46%; m. p.: 38–40 °C; ^1^H-NMR (400 MHz, CDCl_3_): *δ* 4.94–4.80 (m, 1H, COOCH), 2.34 (t, *J* = 7.5 Hz, 2H, COCH_2_), 2.27 (t, *J* = 7.5 Hz, 2H, COCH_2_), 1.72–1.58 (m, 4H, 2 × CH_2_), 1.58–1.43 (m, 4H, 2 × CH_2_), 1.39–1.20 (m, 46H, 23 × CH_2_), 0.88 (t, *J* = 6.8 Hz, 6H, 2 × CH_3_); ^13^C-NMR (100 MHz, CDCl_3_): *δ* 179.4, 173.7, 73.7, 34.7, 34.1, 33.8, 33.8, 31.9, 29.7, 29.7, 29.7, 29.6, 29.6, 29.5, 29.5, 29.4, 29.3, 29.2, 29.2, 25.3, 25.2, 24.8, 24.5, 22.7, 14.1; HRMS (ESI^+^): *m*/*z* calcd for C_34_H_66_NaO_4_^+^: 561.4853; [M + Na]^+^ found: 561.4851.

6-(Oleoyloxy)octadecanoic acid (**7f**). Colorless oil; Yield: 50%; ^1^H-NMR (400 MHz, CDCl_3_): *δ* 5.53–5.20 (m, 2H, 2 × =CH), 4.94–4.81 (m, 1H, COOCH), 2.34 (t, *J* = 7.5 Hz, 2H, COCH_2_), 2.27 (t, *J* = 7.5 Hz, 2H, COCH_2_), 2.12–1.89 (m, 4H, 2 × =CHC*H*_2_), 1.67–1.58 (m, 4H, 2 × CH_2_), 1.57–1.48 (m, 4H, 2 × CH_2_), 1.37–1.22 (m, 42H, 21 × CH_2_), 0.88 (t, *J* = 6.5 Hz, 6H, 2 × CH_3_); ^13^C-NMR (100 MHz, CDCl_3_): *δ* 179.2, 173.7, 130.0, 129.7, 73.7, 34.7, 34.1, 33.8, 33.8, 31.9, 31.9, 29.8, 29.7, 29.7, 29.6, 29.6, 29.5, 29.5, 29.4, 29.3, 29.3, 29.2, 29.2, 29.1, 27.2, 27.2, 25.3, 25.1, 24.8, 24.5, 22.7, 14.1; HRMS (ESI^+^): *m*/*z* calcd for C_36_H_68_NaO_4_^+^: 587.5010; [M + Na]^+^ found: 587.5010.

7-(Hexanoyloxy)octadecanoic acid (**7g**). Off white low-melting point solid; Yield: 38%; ^1^H-NMR (400 MHz, CDCl_3_): *δ* 4.92–4.81 (m, 1H, COOCH), 2.34 (t, *J* = 7.5 Hz, 2H, COCH_2_), 2.27 (t, *J* = 7.5 Hz, 2H, COCH_2_), 1.67–1.56 (m, 4H, 2 × CH_2_), 1.56–1.45 (m, 4H, 2 × CH_2_), 1.39–1.18 (m, 26H, 13 × CH_2_), 0.92–0.84 (m, 6H, 2 × CH_3_); ^13^C-NMR (100 MHz, CDCl_3_): *δ* 179.4, 173.8, 73.9, 34.7, 34.2, 33.9, 33.9, 31.9, 31.3, 29.6, 29.6, 29.6, 29.5, 29.5, 29.3, 28.9, 25.3, 25.0, 24.8, 24.5, 22.7, 22.3, 14.1, 13.9; HRMS (ESI^−^): *m*/*z* calcd for C_24_H_45_O_4_^−^: 397.3323; [M − H]^−^ found: 397.3324.

7-(Palmitoyloxy)octadecanoic acid (**7h**) [20]. White solid; Yield: 48%; m. p.: 39–40 °C; ^1^H-NMR (400 MHz, CDCl_3_): *δ* 4.92–4.80 (m, 1H, COOCH), 2.33 (t, *J* = 7.5 Hz, 2H, COCH_2_), 2.27 (t, *J* = 7.5 Hz, 2H, COCH_2_), 1.66–1.57 (m, 4H, 2 × CH_2_), 1.55–1.44 (m, 4H, 2 × CH_2_), 1.40–1.17 (m, 46H, 23 × CH_2_), 0.87 (t, *J* = 6.9 Hz, 6H, 2 × CH_3_); ^13^C-NMR (100 MHz, CDCl_3_): *δ* 179.8, 173.8, 73.9, 34.7, 34.2, 33.9, 33.9, 31.9, 29.7, 29.6, 29.6, 29.6, 29.6, 29.5, 29.5, 29.5, 29.3, 29.3, 29.3, 29.2, 28.9, 25.3, 25.2, 25.0, 24.5, 22.7, 14.1; HRMS (ESI^+^): *m*/*z* calcd for C_34_H_66_NaO_4_^+^: 561.4853; [M + Na]^+^ found: 561.4870.

7-(Oleoyloxy)octadecanoic acid (**7i**) [20]. Colorless oil; Yield: 49%; ^1^H-NMR (400 MHz, CDCl_3_): *δ* 5.48–5.19 (m, 2H, 2 × =CH), 4.95–4.77 (m, 1H, COOCH), 2.33 (t, *J* = 7.5 Hz, 2H, COCH_2_), 2.27 (t, *J* = 7.5 Hz, 2H, COCH_2_), 2.12–1.89 (m, 4H, 2 × =CHC*H*_2_), 1.68–1.57 (m, 4H, 2 × CH_2_), 1.55–1.44 (m, 4H, 2 × CH_2_), 1.41–1.13 (m, 42H, 21 × CH_2_), 0.87 (t, *J* = 6.7 Hz, 6H, 2 × CH_3_); ^13^C-NMR (100 MHz, CDCl_3_): *δ* 179.7, 173.7, 130.0, 129.7, 73.9, 34.7, 34.1, 33.9, 33.9, 31.9, 29.8, 29.7, 29.6, 29.6, 29.6, 29.5, 29.5, 29.3, 29.3, 29.2, 29.2, 29.1, 28.9, 27.2, 27.2, 25.3, 25.1, 25.0, 24.5, 22.7, 14.1; HRMS (ESI^+^): *m*/*z* calcd for C_36_H_68_NaO_4_^+^: 587.5010; [M + Na]^+^ found: 587.5011.

8-(Hexanoyloxy)octadecanoic acid (**7j**). White solid; Yield: 42%; m. p.: 35–37 °C; ^1^H-NMR (400 MHz, CDCl_3_): *δ* 4.91–4.81 (m, 1H, COOCH), 2.33 (t, *J* = 7.5 Hz, 2H, COCH_2_), 2.27 (t, *J* = 7.5 Hz, 2H, COCH_2_), 1.67–1.57 (m, 4H, 2 × CH_2_), 1.55–1.44 (m, 4H, 2 × CH_2_), 1.41–1.16 (m, 26H, 13 × CH_2_), 0.98–0.79 (m, 6H, 2 × CH_3_); ^13^C-NMR (100 MHz, CDCl_3_): *δ* 179.8, 173.8, 74.0, 34.7, 34.1, 34.1, 34.0, 31.9, 31.3, 29.6, 29.5, 29.5, 29.5, 29.3, 29.1, 28.9, 25.3, 25.1, 24.8, 24.6, 22.7, 22.3, 14.1, 13.9; HRMS (ESI^+^): *m*/*z* calcd for C_24_H_46_NaO_4_^+^: 421.3288; [M + Na]^+^ found: 421.3289.

8-(Palmitoyloxy)octadecanoic acid (**7k**). White solid; Yield: 39%; m. p.: 39–40 °C; ^1^H-NMR (400 MHz, CDCl_3_): *δ* 4.93–4.77 (m, 1H, COOCH), 2.33 (t, *J* = 7.5 Hz, 2H, COCH_2_), 2.27 (t, *J* = 7.5 Hz, 2H, COCH_2_), 1.67–1.57 (m, 4H, 2 × CH_2_), 1.55–1.44 (m, 4H, 2 × CH_2_), 1.37–1.19 (m, 46H, 23 × CH_2_), 0.88 (t, *J* = 6.6 Hz, 6H, 2 × CH_3_); ^13^C-NMR (100 MHz, CDCl_3_): *δ* 179.7, 173.7, 74.0, 34.7, 34.2, 34.1, 34.0, 31.9, 31.9, 29.7, 29.7, 29.6, 29.6, 29.6, 29.5, 29.5, 29.5, 29.3, 29.3, 29.3, 29.2, 29.1, 28.9, 25.3, 25.2, 25.1, 24.6, 22.7, 14.1; HRMS (ESI^+^): *m*/*z* calcd for C_34_H_66_NaO_4_^+^: 561.4853; [M + Na]^+^ found: 561.4854.

8-(Oleoyloxy)octadecanoic acid (**7l**). Colorless oil; Yield: 43%; ^1^H-NMR (400 MHz, CDCl_3_): *δ* 5.44–5.30 (m, 2H, 2 × =CH), 4.91–4.81 (m, 1H, COOCH), 2.33 (t, *J* = 7.5 Hz, 2H, COCH_2_), 2.27 (t, *J* = 7.5 Hz, 2H, COCH_2_), 2.11–1.88 (m, 4H, 2 × =CHC*H*_2_), 1.68–1.56 (m, 4H, 2 × CH_2_), 1.55–1.45 (m, 4H, 2 × CH_2_), 1.42–1.18 (m, 42H, 21 × CH_2_), 0.88 (t, *J* = 6.5 Hz, 6H, 2 × CH_3_); ^13^C-NMR (100 MHz, CDCl_3_): *δ* 179.5, 173.7, 130.0, 129.7, 74.0, 34.7, 34.2, 34.1, 33.9, 31.9, 29.8, 29.7, 29.6, 29.6, 29.5, 29.5, 29.3, 29.3, 29.2, 29.2, 29.1, 29.1, 28.9, 27.2, 27.2, 25.3, 25.2, 25.1, 24.6, 22.7, 14.1; HRMS (ESI^+^): *m*/*z* calcd for C_36_H_68_NaO_4_^+^: 587.5010; [M + Na]^+^ found: 587.5016.

9-(Palmitoyloxy)octadecanoic acid (**7m**) [42]. Colorless oil; Yield: 45%; ^1^H-NMR (400 MHz, CDCl_3_): *δ* 4.93–4.80 (m, 1H, COOCH), 2.34 (t, *J* = 7.5 Hz, 2H, COCH_2_), 2.27 (t, *J* = 7.5 Hz, 2H, COCH_2_), 1.67–1.58 (m, 4H, 2 × CH_2_), 1.58–1.45 (m, 4H, 2 × CH_2_), 1.34–1.23 (m, 46H, 23 × CH_2_), 0.88 (t, *J* = 6.7 Hz, 6H, 2 × CH_3_); ^13^C-NMR (100 MHz, CDCl_3_): *δ* 179.4, 173.8, 74.0, 34.8, 34.2, 34.1, 33.9, 31.9, 31.9, 29.7, 29.7, 29.6, 29.6, 29.5, 29.5, 29.4, 29.3, 29.2, 29.1, 29.0, 25.3, 25.2, 25.2, 24.6, 22.7, 14.1; HRMS (ESI^+^): *m*/*z* calcd for C_34_H_66_NaO_4_^+^: 561.4853; [M + Na]^+^ found: 561.4852.

12-(Hexanoyloxy)octadecanoic acid (**7n**). Colorless oil; Yield: 37%; ^1^H-NMR (400 MHz, CDCl_3_): *δ* 4.92–4.80 (m, 1H, COOCH), 2.33 (t, *J* = 7.5 Hz, 2H, COCH_2_), 2.27 (t, *J* = 7.5 Hz, 2H, COCH_2_), 1.66–1.58 (m, 4H, 2 × CH_2_), 1.55–1.45 (m, 4H, 2 × CH_2_), 1.34–1.22 (m, 26H, 13 × CH_2_), 0.91–0.85 (m, 6H, 2 × CH_3_); ^13^C-NMR (100 MHz, CDCl_3_): *δ* 179.9, 173.8, 74.1, 34.7, 34.1, 34.1, 31.7, 31.3, 29.5, 29.5, 29.4, 29.4, 29.2, 29.2, 29.0, 25.3, 25.3, 24.8, 24.7, 22.5, 22.3, 14.0, 13.9; HRMS (ESI^+^): *m*/*z* calcd for C_24_H_46_NaO_4_^+^: 421.3288; [M + Na]^+^ found: 421.3289.

12-(Palmitoyloxy)octadecanoic acid (**7o**) [56]. White solid; Yield: 40%; m. p.: 39–40 °C; ^1^H-NMR (400 MHz, CDCl_3_): *δ* 4.94–4.80 (m, 1H, COOCH), 2.34 (t, *J* = 7.5 Hz, 2H, COCH_2_), 2.27 (t, *J* = 7.5 Hz, 2H, COCH_2_), 1.66–1.57 (m, 4H, 2 × CH_2_), 1.55–1.46 (m, 4H, 2 × CH_2_), 1.38–1.18 (m, 46H, 23 × CH_2_), 0.87 (t, *J* = 6.2 Hz, 6H, 2 × CH_3_); ^13^C-NMR (100 MHz, CDCl_3_): *δ* 179.7, 173.8, 74.1, 34.8, 34.2, 34.0, 31.9, 31.7, 29.7, 29.7, 29.6, 29.6, 29.5, 29.5, 29.5, 29.4, 29.3, 29.3, 29.2, 29.2, 29.0, 25.3, 25.3, 25.2, 24.7, 22.7, 22.6, 14.1, 14.0; HRMS (ESI^+^): *m*/*z* calcd for C_34_H_66_NaO_4_^+^: 561.4853; [M + Na]^+^ found: 561.4846.

12-(Oleoyloxy)octadecanoic acid (**7p**). Colorless oil; Yield: 36%; ^1^H-NMR (400 MHz, CDCl_3_): *δ* 5.47–5.25 (m, 2H, 2 × =CH), 4.92–4.81 (m, 1H, COOCH), 2.34 (t, *J* = 7.5 Hz, 2H, COCH_2_), 2.27 (t, *J* = 7.5 Hz, 2H, COCH_2_), 2.13–1.88 (m, 4H, 2 × =CHC*H*_2_), 1.67–1.58 (m, 4H, 2 × CH_2_), 1.56–1.45 (m, 4H, 2 × CH_2_), 1.37–1.21 (m, 42H, 21 × CH_2_), 0.88 (t, *J* = 6.1 Hz, 6H, 2 × CH_3_); ^13^C-NMR (100 MHz, CDCl_3_): *δ* 179.5, 173.7, 130.0, 129.8, 74.1, 34.7, 34.2, 34.0, 31.9, 31.8, 29.8, 29.7, 29.7, 29.7, 29.6, 29.5, 29.5, 29.5, 29.4, 29.3, 29.3, 29.3, 29.2, 29.2, 29.1, 29.0, 27.2, 27.2, 25.3, 25.3, 25.2, 24.7, 22.7, 22.6, 14.1, 14.0; HRMS (ESI^+^): *m*/*z* calcd for C_36_H_68_NaO_4_^+^: 587.5010; [M + Na]^+^ found: 587.5010.

### 3.6. General Procedure for the Deprotection of Tert-Butyldimethylsilyl (TBDMS) Group

The appropriate protected alcohol **15a**–**n** (1.00 mmol) was added to a flame-dried flask in a dry THF (3 mL) and a solution of tetra-n-butylammonium fluoride (1 mL, 1.00 mmol, 1 M in THF) was added dropwise. The reaction mixture was left stirring for 1 h at room temperature, and then the solvent was removed under reduced pressure. The desired product was isolated by flash chromatography (petroleum ether (bp 40–60 °C)/ethyl acetate, 80/20–70/30) on silica gel.

1-Hydroxyoctadecan-9-yl hexanoate (**16a**). Colorless oil; Yield: 76%; ^1^H-NMR (400 MHz, CDCl_3_): *δ* 4.91–4.81 (m, 1H, COOCH), 3.63 (t, *J* = 6.6 Hz, 2H, CH_2_OH), 2.27 (t, *J* = 7.5 Hz, 2H, CH_2_COO), 1.67–1.42 (m, 9H, 4 × CH_2_ and OH), 1.37–1.22 (m, 28H, 14 × CH_2_), 0.93–0.83 (m, 6H, 2 × CH_3_); ^13^C-NMR (100 MHz, CDCl_3_): *δ* 173.7, 74.0, 63.0, 34.7, 34.2, 34.1, 32.8, 31.9, 31.3, 29.5, 29.4, 29.4, 29.3, 25.7, 25.3, 25.3, 24.8, 22.7, 22.3, 14.1, 13.9; HRMS (ESI^+^): *m*/*z* calcd for C_24_H_48_NaO_3_^+^: 407.3496; [M + Na]^+^ found: 407.3480.

1-Hydroxyoctadecan-9-yl oleate (**16b**) [22]. Colorless oil; Yield: 74%; ^1^H-NMR (400 MHz, CDCl_3_): *δ* 5.43–5.26 (m, 2H, 2 × =CH), 4.93–4.79 (m, 1H, COOCH), 3.63 (t, *J* = 6.6 Hz, 2H, CH_2_OH), 2.27 (t, *J* = 7.5 Hz, 2H, CH_2_COO), 2.10–1.88 (m, 4H, 2 × =CHC*H*_2_), 1.64–1.47 (m, 8H, 4 × CH_2_), 1.43–1.20 (m, 45H, 22 × CH_2_ and OH), 0.88 (t, *J* = 6.6 Hz, 6H, 2 × CH_3_); ^13^C-NMR (100 MHz, CDCl_3_): *δ* 173.7, 130.0, 129.7, 74.0, 63.0, 34.7, 34.2, 34.1, 32.8, 31.9, 29.8, 29.7, 29.6, 29.6, 29.5, 29.5, 29.5, 29.4, 29.4, 29.3, 29.3, 29.3, 29.2, 29.2, 29.1, 27.2, 27.2, 25.7, 25.3, 25.3, 25.2, 22.7, 14.1; HRMS (ESI^+^): *m*/*z* calcd for C_36_H_70_NaO_3_^+^: 573.5217; [M + Na]^+^ found: 573.5202.

18-Hydroxyoctadecan-9-yl hexanoate (**16c**). Colorless oil; Yield: 79%; ^1^H-NMR (400 MHz, CDCl_3_): *δ* 4.96–4.76 (m, 1H, COOCH), 3.63 (t, *J* = 6.6 Hz, 2H, CH_2_OH), 2.27 (t, *J* = 7.4 Hz, 2H, CH_2_COO), 1.66–1.45 (m, 9H, 4 × CH_2_ and OH), 1.39–1.19 (m, 28H, 14 × CH_2_), 0.98–0.78 (m, 6H, 2 × CH_3_); ^13^C-NMR (100 MHz, CDCl_3_): *δ* 173.7, 74.1, 63.1, 34.7, 34.2, 34.1, 32.8, 31.9, 31.4, 29.7, 29.5, 29.5, 29.5, 29.4, 29.4, 29.2, 25.7, 25.3, 25.3, 24.9, 22.6, 22.3, 14.1, 13.9; HRMS (ESI^+^): *m*/*z* calcd for C_24_H_48_NaO_3_^+^: 407.3496; [M + Na]^+^ found: 407.3509.

18-Hydroxyoctadecan-9-yl palmitate (**16d**). Colorless oil; Yield: 75%; ^1^H-NMR (400 MHz, CDCl_3_): *δ* 4.92–4.79 (m, 1H, COOCH), 3.63 (t, *J* = 6.6 Hz, 2H, CH_2_OH), 2.27 (t, *J* = 7.5 Hz, 2H, CH_2_COO), 1.65–1.46 (m, 11H, 5 × CH_2_ and OH), 1.35–1.22 (m, 46H, 23 × CH_2_), 0.87 (t, *J* = 6.5 Hz, 6H, 2 × CH_3_); ^13^C-NMR (100 MHz, CDCl_3_): *δ* 173.7, 74.0, 63.0, 34.7, 34.2, 34.1, 32.8, 31.9, 31.9, 29.7, 29.7, 29.6, 29.6, 29.5, 29.5, 29.5, 29.4, 29.4, 29.3, 29.2, 29.2, 25.7, 25.3, 25.3, 25.2, 22.7, 22.6, 14.1, 14.1; HRMS (ESI^+^): *m*/*z* calcd for C_34_H_68_NaO_3_^+^: 547.5061; [M + Na]^+^ found: 547.5070.

18-Hydroxyoctadecan-9-yl oleate (**16e**) [20]. Colorless oil; Yield: 73%; ^1^H-NMR (400 MHz, CDCl_3_): *δ* 5.51–5.21 (m, 2H, 2 × =CH), 4.94–4.79 (m, 1H, COOCH), 3.63 (t, *J* = 6.6 Hz, 2H, CH_2_OH), 2.27 (t, *J* = 7.5 Hz, 2H, CH_2_COO), 2.11–1.89 (m, 4H, 2 × =CHC*H*_2_), 1.66–1.45 (m, 10H, 5 × CH_2_), 1.40–1.17 (m, 43H, 21 × CH_2_ and OH), 0.88 (t, *J* = 6.3 Hz, 6H, 2 × CH_3_); ^13^C-NMR (100 MHz, CDCl_3_): *δ* 173.7, 130.0, 129.7, 74.1, 63.0, 34.7, 34.1, 32.8, 31.9, 31.8, 29.8, 29.7, 29.6, 29.6, 29.5, 29.5, 29.5, 29.5, 29.5, 29.4, 29.4, 29.3, 29.3, 29.2, 29.2, 29.2, 29.1, 27.2, 27.2, 25.7, 25.3, 25.3, 25.2, 22.7, 22.6, 14.1, 14.1; HRMS (ESI^+^): *m*/*z* calcd for C_36_H_70_NaO_3_^+^: 573.5217; [M + Na]^+^ found: 573.5229.

18-Hydroxyoctadecan-8-yl hexanoate (**16f**). Colorless oil; Yield: 78%; ^1^H-NMR (400 MHz, CDCl_3_): *δ* 4.92–4.80 (m, 1H, COOCH), 3.63 (t, *J* = 6.6 Hz, 2H, CH_2_OH), 2.27 (t, *J* = 7.5 Hz, 2H, CH_2_COO), 1.66–1.47 (m, 9H, 4 × CH_2_ and OH), 1.35–1.24 (m, 28H, 14 × CH_2_), 0.93–0.84 (m, 6H, 2 × CH_3_); ^13^C-NMR (100 MHz, CDCl_3_): *δ* 173.7, 74.1, 63.1, 34.7, 34.2, 32.8, 31.8, 31.3, 29.5, 29.5, 29.4, 29.4, 29.2, 25.7, 25.3, 25.3, 24.8, 22.6, 22.3, 14.0, 13.9; HRMS (ESI^+^): *m*/*z* calcd for C_24_H_48_NaO_3_^+^: 407.3496; [M + Na]^+^ found: 407.3497.

18-Hydroxyoctadecan-8-yl palmitate (**16g**). Colorless oil; Yield: 75%; ^1^H-NMR (400 MHz, CDCl_3_): *δ* 4.93–4.80 (m, 1H, COOCH), 3.63 (t, *J* = 6.6 Hz, 2H, OCH_2_), 2.27 (t, *J* = 7.4 Hz, 2H, COCH_2_), 1.68–1.44 (m, 10H, 5 × CH_2_), 1.36–1.23 (m, 47H, 23 × CH_2_ and OH), 0.88 (t, *J* = 6.6 Hz, 6H, 2 × CH_3_); ^13^C-NMR (100 MHz, CDCl_3_): *δ* 173.7, 74.1, 63.1, 34.8, 34.2, 32.8, 31.9, 31.8, 29.7, 29.6, 29.6, 29.5, 29.5, 29.5, 29.4, 29.3, 29.3, 29.2, 25.7, 25.3, 25.2, 22.7, 22.6, 14.1, 14.0; HRMS (ESI^+^): *m*/*z* calcd for C_34_H_68_NaO_3_^+^: 547.5061; [M + Na]^+^ found: 547.5065.

18-Hydroxyoctadecan-8-yl oleate (**16h**). Colorless oil; Yield: 79%; ^1^H-NMR (400 MHz, CDCl_3_): *δ* 5.45–5.25 (m, 2H, 2 × =CH), 4.92–4.80 (m, 1H, COOCH), 3.63 (t, *J* = 6.5 Hz, 2H, OCH_2_), 2.27 (t, *J* = 7.4 Hz, 2H, CH_2_COO), 2.05–1.95 (m, 4H, 2 × =CHC*H*_2_), 1.65–1.46 (m, 11H, 5 × CH_2_ and OH), 1.36–1.24 (m, 42H, 21 × CH_2_), 0.88 (t, *J* = 6.2 Hz, 6H, 2 × CH_3_); ^13^C-NMR (100 MHz, CDCl_3_): *δ* 173.7, 130.0, 129.7, 74.1, 63.0, 34.7, 34.2, 32.8, 31.9, 31.8, 29.8, 29.7, 29.6, 29.6, 29.5, 29.5, 29.5, 29.4, 29.3, 29.3, 29.2, 29.2, 29.1, 27.2, 27.2, 25.7, 25.3, 25.2, 22.7, 22.6, 14.1, 14.1; HRMS (ESI^+^): *m*/*z* calcd for C_36_H_70_NaO_3_^+^: 573.5217; [M + Na]^+^ found: 573.5220.

18-Hydroxyoctadecan-6-yl hexanoate (**16i**). Colorless oil; Yield: 71%; ^1^H-NMR (400 MHz, CDCl_3_): *δ* 4.91–4.81 (m, 1H, COOCH), 3.63 (t, *J* = 6.6 Hz, 2H, CH_2_OH), 2.27 (t, *J* = 7.5 Hz, 2H, CH_2_COO), 1.71–1.40 (m, 9H, 4 × CH_2_ and OH), 1.37–1.22 (m, 28H, 14 × CH_2_), 0.94–0.83 (m, 6H, 2 × CH_3_); ^13^C-NMR (100 MHz, CDCl_3_): *δ* 173.7, 74.1, 63.1, 34.7, 34.1, 34.1, 32.8, 31.7, 31.3, 29.6, 29.5, 29.5, 29.5, 29.4, 25.7, 25.3, 25.0, 24.8, 22.5, 22.3, 14.0, 13.9; HRMS (ESI^+^): *m*/*z* calcd for C_24_H_48_NaO_3_^+^: 407.3496; [M + Na]^+^ found: 407.3500.

18-Hydroxyoctadecan-6-yl palmitate (**16j**). Colorless oil; Yield: 71%; ^1^H-NMR (400 MHz, CDCl_3_): *δ* 4.92–4.80 (m, 1H, COOCH), 3.62 (t, *J* = 6.6 Hz, 2H, OCH_2_), 2.26 (t, *J* = 7.4 Hz, 2H, COCH_2_), 1.62–1.46 (m, 11H, 5 × CH_2_ and OH), 1.34–1.23 (m, 46H, 23 × CH_2_), 0.87 (t, *J* = 6.5 Hz, 6H, 2 × CH_3_); ^13^C-NMR (100 MHz, CDCl_3_): *δ* 173.7, 74.1, 63.0, 34.7, 34.1, 34.1, 32.8, 31.9, 31.7, 29.7, 29.6, 29.6, 29.6, 29.5, 29.5, 29.5, 29.5, 29.4, 29.3, 29.3, 29.2, 25.7, 25.3, 25.2, 25.0, 22.7, 22.5, 14.1, 14.0; HRMS (ESI^+^): *m*/*z* calcd for C_34_H_68_NaO_3_^+^: 547.5061; [M + Na]^+^ found: 547.5065.

18-Hydroxyoctadecan-6-yl oleate (**16k**). Colorless oil; Yield: 76%; ^1^H-NMR (400 MHz, CDCl_3_): *δ* 5.45–5.23 (m, 2H, 2 × =CH), 4.91–4.81 (m, 1H, COOCH), 3.63 (t, *J* = 6.6 Hz, 2H, OCH_2_), 2.27 (t, *J* = 7.5 Hz, 2H, CH_2_COO), 2.10–1.89 (m, 4H, 2 × =CHC*H*_2_), 1.71–1.42 (m, 11H, 5 × CH_2_ and OH), 1.35–1.23 (m, 42H, 21 × CH_2_), 0.87 (t, *J* = 6.3 Hz, 6H, 2 × CH_3_); ^13^C-NMR (100 MHz, CDCl_3_): *δ* 173.7, 130.0, 129.7, 74.1, 63.0, 34.7, 34.1, 34.1, 32.8, 31.9, 31.7, 29.8, 29.7, 29.6, 29.6, 29.5, 29.5, 29.5, 29.4, 29.3, 29.2, 29.1, 29.1, 27.2, 27.2, 25.7, 25.3, 25.2, 25.0, 22.7, 22.5, 14.1, 14.0; HRMS (ESI^+^): *m*/*z* calcd for C_36_H_70_NaO_3_^+^: 573.5217; [M + Na]^+^ found: 573.5221.

18-Hydroxyoctadecan-5-yl hexanoate (**16l**). Colorless oil; Yield: 78%; ^1^H-NMR (400 MHz, CDCl_3_): *δ* 4.95–4.79 (m, 1H, COOCH), 3.63 (t, *J* = 6.6 Hz, 2H, CH_2_OH), 2.27 (t, *J* = 7.5 Hz, 2H, CH_2_COO), 1.66–1.48 (m, 9H, 4 × CH_2_ and OH), 1.43–1.17 (m, 28H, 14 × CH_2_), 0.99–0.80 (m, 6H, 2 × CH_3_); ^13^C-NMR (100 MHz, CDCl_3_): *δ* 173.7, 74.1, 63.1, 34.7, 34.2, 33.8, 32.8, 31.3, 29.6, 29.6, 29.6, 29.5, 29.5, 29.4, 27.5, 25.7, 25.3, 24.8, 22.6, 22.3, 14.0, 13.9; HRMS (ESI^+^): *m*/*z* calcd for C_24_H_48_NaO_3_^+^: 407.3496; [M + Na]^+^ found: 407.3496.

18-Hydroxyoctadecan-5-yl palmitate (**16m**). Colorless oil; Yield: 80%; ^1^H-NMR (400 MHz, CDCl_3_): *δ* 4.91–4.82 (m, 1H, COOCH), 3.63 (t, *J* = 6.6 Hz, 2H, CH_2_OH), 2.27 (t, *J* = 7.4 Hz, 2H, CH_2_COO), 1.65–1.43 (m, 9H, 4 × CH_2_ and OH), 1.38–1.16 (m, 48H, 24 × CH_2_), 0.87 (t, *J* = 5.9 Hz, 6H, 2 × CH_3_); ^13^C-NMR (100 MHz, CDCl_3_): *δ* 173.7, 74.0, 63.1, 34.7, 34.2, 33.9, 32.8, 31.9, 29.7, 29.7, 29.6, 29.6, 29.6, 29.6, 29.5, 29.5, 29.4, 29.3, 29.3, 29.2, 27.5, 25.7, 25.3, 25.2, 22.7, 22.6, 14.1, 14.0; HRMS (ESI^+^): *m*/*z* calcd for C_34_H_68_NaO_3_^+^: 547.5061; [M + Na]^+^ found: 547.5064.

18-Hydroxyoctadecan-5-yl oleate (**16n**). Colorless oil; Yield: 79%; ^1^H-NMR (400 MHz, CDCl_3_): *δ* 5.47–5.25 (m, 2H, 2 × =CH), 4.94–4.80 (m, 1H, COOCH), 3.63 (t, *J* = 6.6 Hz, 2H, CH_2_OH), 2.27 (t, *J* = 7.5 Hz, 2H, CH_2_COO), 2.11–1.90 (m, 4H, 2 × =CHC*H*_2_), 1.65–1.48 (m, 9H, 4 × CH_2_ and OH), 1.38–1.21 (m, 44H, 22 × CH_2_), 0.88 (t, *J* = 6.8 Hz, 6H, 2 × CH_3_); ^13^C-NMR (100 MHz, CDCl_3_): *δ* 173.7, 130.0, 129.8, 74.1, 63.1, 34.7, 34.2, 33.9, 32.8, 31.9, 29.8, 29.7, 29.6, 29.6, 29.6, 29.6, 29.6, 29.5, 29.5, 29.4, 29.3, 29.2, 29.2, 29.1, 27.5, 27.2, 27.2, 25.7, 25.3, 25.2, 22.7, 22.6, 14.1, 14.0; HRMS (ESI^+^): *m*/*z* calcd for C_36_H_70_NaO_3_^+^: 573.5217; [M + Na]^+^ found: 573.5215.

### 3.7. General Procedure for the Oxidation of Alcohols ***16a–n*** to FAHFAs

To a round-bottomed flask containing the appropriate alcohol **16a**–**n** (1.00 mmol), acetone (10 mL) was added, and the mixture was left stirring at 0 °C until full dissolution. Jones reagent (2 M, 1.5 mL, 3.00 mmol) was added dropwise at 0 °C and the reaction mixture was left stirring for 1 h at this temperature. Then, the reaction mixture was quenched with a saturated aqueous solution of NaHSO_3_ (10 mL) and extracted with Et_2_O (3 × 20 mL). The combined organic layers were washed with brine (1 × 50 mL), dried over Na_2_SO_4,_ and,] then evaporated to give a crude mixture, which was purified by flash column chromatography on silica gel (petroleum ether (bp 40–60 °C)/ethyl acetate, 60/40) to afford the desired FAHFAs.

9-(Hexanoyloxy)octadecanoic acid (**17a**) [14]. Colorless oil; Yield: 84%; ^1^H-NMR (400 MHz, CDCl_3_): *δ* 4.90–4.83 (m, 1H, COOCH), 2.34 (t, *J* = 7.5 Hz, 2H, COCH_2_), 2.28 (t, *J* = 7.5 Hz, 2H, COCH_2_), 1.66–1.59 (m, 4H, 2 × CH_2_), 1.54–1.46 (m, 4H, 2 × CH_2_), 1.35–1.23 (m, 26H, 13 × CH_2_), 0.93–0.84 (m, 6H, 2 × CH_3_); ^13^C-NMR (100 MHz, CDCl_3_): *δ* 179.3, 173.8, 74.0, 34.7, 34.2, 34.1, 33.9, 31.9, 31.3, 29.5, 29.5, 29.3, 29.1, 29.0, 25.3, 25.2, 24.8, 24.6, 22.7, 22.3, 14.1, 13.9; HRMS (ESI^+^): *m*/*z* calcd for C_24_H_46_NaO_4_^+^: 421.3288; [M + Na]^+^ found: 421.3286.

9-(Oleoyloxy)octadecanoic acid (**17b**) [57]. Colorless oil; Yield: 82%; ^1^H-NMR (400 MHz, CDCl_3_): *δ* 5.50–5.22 (m, 2H, 2 × =CH), 4.94–4.78 (m, 1H, COOCH), 2.34 (t, *J* = 7.5 Hz, 2H, CH_2_COO), 2.27 (t, *J* = 7.5 Hz, 2H, CH_2_COO), 2.12–1.89 (m, 4H, 2 × =CHC*H*_2_), 1.66–1.59 (m, 4H, 2 × CH_2_), 1.55–1.46 (m, 4H, 2 × CH_2_), 1.36–1.22 (m, 42H, 21 × CH_2_), 0.88 (t, *J* = 6.7 Hz, 6H, 2 × CH_3_); ^13^C-NMR (100 MHz, CDCl_3_): *δ* 178.6, 173.7, 130.0, 129.8, 74.0, 34.7, 34.2, 34.1, 33.8, 31.9, 31.9, 29.8, 29.7, 29.7, 29.6, 29.6, 29.5, 29.5, 29.5, 29.4, 29.3, 29.2, 29.2, 29.1, 29.1, 29.1, 29.0, 27.2, 27.2, 25.3, 25.3, 25.2, 24.6, 22.7, 14.1; HRMS (ESI^+^): *m*/*z* calcd for C_36_H_68_NaO_4_^+^: 587.5010; [M + Na]^+^ found: 587.5010.

10-(Hexanoyloxy)octadecanoic acid (**17c**). Colorless oil; Yield: 84%; ^1^H-NMR (400 MHz, CDCl_3_): *δ* 4.95–4.77 (m, 1H, COOCH), 2.34 (t, *J* = 7.5 Hz, 2H, COCH_2_), 2.28 (t, *J* = 7.5 Hz, 2H, COCH_2_), 1.67–1.58 (m, 4H, 2 × CH_2_), 1.58–1.39 (m, 4H, 2 × CH_2_), 1.33–1.23 (m, 26H, 13 × CH_2_), 0.93–0.82 (m, 6H, 2 × CH_3_); ^13^C-NMR (100 MHz, CDCl_3_): *δ* 179.0, 173.8, 74.1, 34.7, 34.1, 33.9, 31.9, 31.9, 31.3, 29.7, 29.7, 29.6, 29.5, 29.5, 29.4, 29.4, 29.3, 29.2, 29.1, 29.1, 29.0, 25.3, 25.3, 24.8, 24.7, 24.6, 22.7, 22.7, 22.3, 14.1, 13.9; HRMS (ESI^+^): *m*/*z* calcd for C_24_H_46_NaO_4_^+^: 421.3288; [M + Na]^+^ found: 421.3286.

10-(Palmitoyloxy)octadecanoic acid (**17d**) [58]. White solid; Yield: 86%; m.p.: 36–37 °C; ^1^H-NMR (400 MHz, CDCl_3_): *δ* 4.94–4.78 (m, 1H, COOCH), 2.34 (t, *J* = 7.5 Hz, 2H, COCH_2_), 2.27 (t, *J* = 7.5 Hz, 2H, COCH_2_), 1.67–1.57 (m, 4H, 2 × CH_2_), 1.56–1.42 (m, 4H, 2 × CH_2_), 1.37–1.22 (m, 46H, 23 × CH_2_), 0.88 (t, *J* = 6.6 Hz, 6H, 2 × CH_3_); ^13^C-NMR (100 MHz, CDCl_3_): *δ* 179.0, 173.8, 74.1, 34.8, 34.2, 33.9, 31.9, 31.9, 29.7, 29.7, 29.7, 29.6, 29.5, 29.5, 29.4, 29.4, 29.3, 29.3, 29.2, 29.2, 29.1, 29.0, 25.3, 25.3, 25.2, 24.7, 22.7, 22.7, 14.1, 14.1; HRMS (ESI^+^): *m*/*z* calcd for C_34_H_66_NaO_4_^+^: 561.4853; [M + Na]^+^ found: 561.4858.

10-(Oleoyloxy)octadecanoic acid (**17e**) [20]. Colorless oil; Yield: 83%;^1^H-NMR (400 MHz, CDCl_3_): *δ* 5.49–5.25 (m, 2H, 2 × =CH), 4.93–4.78 (m, 1H, COOCH), 2.34 (t, *J* = 7.5 Hz, 2H, CH_2_COO), 2.27 (t, *J* = 7.5 Hz, 2H, CH_2_COO), 2.17–1.82 (m, 4H, 2 × =CHC*H*_2_), 1.67–1.57 (m, 4H, 2 × CH_2_), 1.55–1.44 (m, 4H, 2 × CH_2_), 1.36–1.22 (m, 42H, 21 × CH_2_), 0.88 (t, *J* = 6.5 Hz, 6H, 2 × CH_3_); ^13^C-NMR (100 MHz, CDCl_3_): *δ* 178.8, 173.7, 130.0, 129.8, 74.1, 34.7, 34.2, 33.8, 31.9, 31.9, 29.8, 29.7, 29.7, 29.5, 29.5, 29.5, 29.4, 29.3, 29.3, 29.3, 29.2, 29.2, 29.2, 29.1, 29.0, 27.2, 27.2, 25.3, 25.3, 25.2, 24.7, 22.7, 22.7, 14.1; HRMS (ESI^+^): *m*/*z* calcd for C_36_H_68_NaO_4_^+^: 587.5010; [M + Na]^+^ found: 587.5010.

11-(Hexanoyloxy)octadecanoic acid (**17f**). Colorless oil; Yield: 80%; ^1^H-NMR (400 MHz, CDCl_3_): *δ* 4.93–480 (m, 1H, COOCH), 2.34 (t, *J* = 7.5 Hz, 2H, COCH_2_), 2.28 (t, *J* = 7.5 Hz, 2H, COCH_2_), 1.66–1.59 (m, 4H, 2 × CH_2_), 1.54–1.46 (m, 4H, 2 × CH_2_), 1.36–1.24 (m, 26H, 13 × CH_2_), 0.93–0.84 (m, 6H, 2 × CH_3_); ^13^C-NMR (100 MHz, CDCl_3_): *δ* 179.2, 173.8, 74.1, 34.7, 34.2, 33.9, 31.8, 31.3, 29.5, 29.5, 29.4, 29.3, 29.2, 29.2, 29.0, 25.3, 25.3, 24.9, 24.7, 22.6, 22.3, 14.1, 13.9; HRMS (ESI^+^): *m*/*z* calcd for C_24_H_46_NaO_4_^+^: 421.3288; [M + Na]^+^ found: 421.3288.

11-(Palmitoyloxy)octadecanoic acid (**17g**). Colorless oil; Yield: 85%; ^1^H-NMR (400 MHz, CDCl_3_): *δ* 4.92–4.81 (m, 1H, COOCH), 2.34 (t, *J* = 7.5 Hz, 2H, COCH_2_), 2.27 (t, *J* = 7.5 Hz, 2H, COCH_2_), 1.65–1.59 (m, 4H, 2 × CH_2_), 1.53–1.47 (m, 4H, 2 × CH_2_), 1.34–1.23 (m, 46H, 23 × CH_2_), 0.88 (t, *J* = 6.6 Hz, 6H, 2 × CH_3_); ^13^C-NMR (100 MHz, CDCl_3_): *δ* 179.2, 173.8, 74.1, 34.8, 34.2, 33.9, 31.9, 31.8, 29.7, 29.7, 29.6, 29.5, 29.4, 29.4, 29.3, 29.3, 29.2, 29.0, 25.3, 25.2, 24.7, 22.7, 22.6, 14.1, 14.1; HRMS (ESI^+^): *m*/*z* calcd for C_34_H_66_NaO_4_^+^: 561.4853; [M + Na]^+^ found: 561.4863.

11-(Oleoyloxy)octadecanoic acid (**17h**). Colorless oil; Yield: 86%; ^1^H-NMR (400 MHz, CDCl_3_): *δ* 5.47–5.23 (m, 2H, 2 × =CH), 4.92–4.80 (m, 1H, COOCH), 2.34 (t, *J* = 7.5 Hz, 2H, CH_2_COO), 2.27 (t, *J* = 7.5 Hz, 2H, CH_2_COO), 2.14–1.87 (m, 4H, 2 × =CHC*H*_2_), 1.67–1.58 (m, 4H, 2 × CH_2_), 1.56–1.46 (m, 4H, 2 × CH_2_), 1.37–1.22 (m, 42H, 21 × CH_2_), 0.88 (t, *J* = 6.4 Hz, 6H, 2 × CH_3_); ^13^C-NMR (100 MHz, CDCl_3_): *δ* 179.0, 173.7, 130.0, 129.8, 74.1, 34.7, 34.2, 33.9, 31.9, 31.8, 29.8, 29.7, 29.7, 29.5, 29.5, 29.5, 29.4, 29.3, 29.3, 29.2, 29.2, 29.2, 29.1, 29.0, 27.2, 27.2, 25.3, 25.2, 24.7, 22.7, 22.6, 14.1, 14.1; HRMS (ESI^+^): *m*/*z* calcd for C_36_H_68_NaO_4_^+^: 587.5010; [M + Na]^+^ found: 587.5009.

13-(Hexanoyloxy)octadecanoic acid (**17i**). Colorless oil; Yield: 82%; ^1^H-NMR (400 MHz, CDCl_3_): *δ* 4.93–4.80 (m, 1H, COOCH), 2.34 (t, *J* = 7.1 Hz, 2H, COCH_2_), 2.27 (t, *J* = 7.1 Hz, 2H, COCH_2_), 1.68–1.57 (m, 4H, 2 × CH_2_), 1.55–1.44 (m, 4H, 2 × CH_2_), 1.38–1.19 (m, 26H, 13 × CH_2_), 0.94–0.81 (m, 6H, 2 × CH_3_); ^13^C-NMR (100 MHz, CDCl_3_): *δ* 179.7, 173.8, 74.1, 34.7, 34.1, 34.1, 34.0, 31.7, 31.3, 29.5, 29.4, 29.2, 29.0, 25.3, 25.0, 24.8, 24.7, 22.5, 22.3, 14.0, 13.9; HRMS (ESI^+^): *m*/*z* calcd for C_24_H_46_NaO_4_^+^: 421.3288; [M + Na]^+^ found: 421.3287.

13-(Palmitoyloxy)octadecanoic acid (**17j**). White solid; Yield: 89%; m.p.: 39–41 °C; ^1^H-NMR (400 MHz, CDCl_3_): *δ* 4.91–4.82 (m, 1H, COOCH), 2.34 (t, *J* = 7.5 Hz, 2H, COCH_2_), 2.27 (t, *J* = 7.5 Hz, 2H, COCH_2_), 1.67–1.59 (m, 4H, 2 × CH_2_), 1.54–1.47 (m, 4H, 2 × CH_2_), 1.35–1.23 (m, 46H, 23 × CH_2_), 0.88 (t, *J* = 6.5 Hz, 6H, 2 × CH_3_); ^13^C-NMR (100 MHz, CDCl_3_): *δ* 179.3, 173.8, 74.1, 34.8, 34.2, 34.1, 33.9, 31.9, 31.7, 29.7, 29.7, 29.6, 29.5, 29.4, 29.4, 29.3, 29.2, 29.2, 29.0, 25.3, 25.2, 25.0, 24.7, 22.7, 22.5, 14.1, 14.0; HRMS (ESI^+^): *m*/*z* calcd for C_34_H_66_NaO_4_^+^: 561.4853; [M + Na]^+^ found: 561.4879.

13-(Oleoyloxy)octadecanoic acid (**17k**). Colorless oil; Yield: 87%; ^1^H-NMR (400 MHz, CDCl_3_): *δ* 5.42–5.26 (m, 2H, 2 × =CH), 4.90–4.83 (m, 1H, COOCH), 2.34 (t, *J* = 7.5 Hz, 2H, CH_2_COO), 2.27 (t, *J* = 7.5 Hz, 2H, CH_2_COO), 2.10–1.87 (m, 4H, 2 × =CHC*H*_2_), 1.66–1.59 (m, 4H, 2 × CH_2_), 1.53–1.46 (m, 4H, 2 × CH_2_), 1.34–1.24 (m, 42H, 21 × CH_2_), 0.88 (t, *J* = 6.3 Hz, 6H, 2 × CH_3_); ^13^C-NMR (100 MHz, CDCl_3_): *δ* 179.5, 173.7, 130.0, 129.7, 74.1, 34.7, 34.2, 34.1, 34.0, 31.9, 31.7, 29.8, 29.7, 29.6, 29.6, 29.5, 29.4, 29.3, 29.3, 29.2, 29.2, 29.2, 29.1, 29.0, 29.0, 27.2, 27.2, 25.3, 25.2, 25.0, 24.7, 22.7, 22.5, 14.1, 14.0; HRMS (ESI^+^): *m*/*z* calcd for C_36_H_68_NaO_4_^+^: 587.5010; [M + Na]^+^ found: 587.5010.

14-(Hexanoyloxy)octadecanoic acid (**17l**). Colorless oil; Yield: 81%; ^1^H-NMR (400 MHz, CDCl_3_): *δ* 4.95–4.80 (m, 1H, COOCH), 2.34 (t, *J* = 7.5 Hz, 2H, COCH_2_), 2.28 (t, *J* = 7.5 Hz, 2H, COCH_2_), 1.67–1.58 (m, 4H, 2 × CH_2_), 1.55–1.46 (m, 4H, 2 × CH_2_), 1.38–1.21 (m, 26H, 13 × CH_2_), 0.94–0.83 (m, 6H, 2 × CH_3_); ^13^C-NMR (100 MHz, CDCl_3_): *δ* 179.5, 173.8, 74.1, 34.7, 34.2, 34.0, 33.8, 31.3, 29.6, 29.5, 29.5, 29.4, 29.2, 29.0, 27.5, 25.3, 24.8, 24.7, 22.6, 22.3, 14.0, 13.9; HRMS (ESI^+^): *m*/*z* calcd for C_24_H_46_NaO_4_^+^: 421.3288; [M + Na]^+^ found: 421.3288.

14-(Palmitoyloxy)octadecanoic acid (**17m**). White solid; Yield: 80%; m. p.: 41–42 °C; ^1^H-NMR (400 MHz, CDCl_3_): *δ* 4.94–4.80 (m, 1H, COOCH), 2.34 (t, *J* = 7.5 Hz, 2H, COCH_2_), 2.28 (t, *J* = 7.5 Hz, 2H, COCH_2_), 1.67–1.58 (m, 4H, 2 × CH_2_), 1.54–1.47 (m, 4H, 2 × CH_2_), 1.39–1.20 (m, 46H, 23 × CH_2_), 0.88 (t, *J* = 6.5 Hz, 6H, 2 × CH_3_); ^13^C-NMR (100 MHz, CDCl_3_): *δ* 179.1, 173.8, 74.1, 34.8, 34.2, 33.9, 33.9, 31.9, 29.7, 29.7, 29.6, 29.6, 29.6, 29.5, 29.5, 29.4, 29.4, 29.3, 29.2, 29.2, 29.1, 27.5, 25.3, 25.2, 24.7, 22.7, 22.6, 14.1, 14.0; HRMS (ESI^+^): *m*/*z* calcd for C_34_H_66_NaO_4_^+^: 561.4853; [M+Na]^+^ found: 561.4853.

14-(Oleoyloxy)octadecanoic acid (**17n**). Colorless oil; Yield: 85%; ^1^H-NMR (400 MHz, CDCl_3_): *δ* 5.49–5.23 (m, 2H, 2 × =CH), 4.95–4.79 (m, 1H, COOCH), 2.34 (t, *J* = 7.5 Hz, 2H, CH_2_COO), 2.28 (t, *J* = 7.5 Hz, 2H, CH_2_COO), 2.17–1.81 (m, 4H, 2 × =CHC*H*_2_), 1.67–1.58 (m, 4H, 2 × CH_2_), 1.56–1.46 (m, 4H, 2 × CH_2_), 1.37–1.16 (m, 42H, 21 × CH_2_), 0.88 (t, *J* = 5.8 Hz, 6H, 2 × CH_3_); ^13^C-NMR (100 MHz, CDCl_3_): *δ* 179.2, 173.8, 130.0, 129.7, 74.1, 34.7, 34.2, 33.9, 33.8, 31.9, 29.8, 29.7, 29.7, 29.6, 29.5, 29.5, 29.5, 29.4, 29.3, 29.2, 29.2, 29.2, 29.1, 29.1, 27.5, 27.2, 27.2, 25.3, 25.2, 24.7, 22.7, 22.6, 14.1, 14.0; HRMS (ESI^+^): *m*/*z* calcd for C_36_H_68_NaO_4_^+^: 587.5010; [M + Na]^+^ found: 587.5007.

## 4. Conclusions

In summary, we have developed a compact and convenient strategy for synthesizing endogenous lipids, FAHFAs, which are notable for their potent antidiabetic and anti-inflammatory effects. Our innovative approach harnesses the power of a highly efficient photo-induced hydroacylation reaction between terminal alkenes, either *ω*-alkenoic acids or *ω*-alkenyl alcohols, and commercially available aldehydes, utilizing phenylglyoxylic acid as the photoinitiator under household lamp irradiation as the key process. This environmentally friendly reaction not only enables the production of a rich array of oxo fatty acids and other oxo-functionalized compounds but also lays the foundation for the facile synthesis of FAHFAs. By thoughtfully selecting suitable olefins and aldehydes, we successfully introduced the ester linkage at various positions along the aliphatic chain, leading to the formation of a spectrum of regio-isomers and adding an extra layer of versatility to our methodology. Building upon this foundation, subsequent coupling reactions with diverse fatty acids (FAs) allowed us to synthesize a broad array of FAHFA families. This not onlypaves the way for an in-depth exploration of FAHFA structural variations, but also positions our work at the forefront of understanding their diverse biological activities.

## Data Availability

All data supporting this study are included in the article and Supplementary Materials.

## Supplementary Materials

The following supporting information can be downloaded at https://www.mdpi.com/article/10.3390/molecules30020286/s1, Scheme S1: Graphical representation of the structures of all final FAHFA compounds; Scheme S2: The photochemical hydroacylation reaction of aldehydes with alkenes to oxo fatty acids (products with yields); Scheme S3: The synthesis of oxo methyl esters from oxo fatty acids (products with yields); Scheme S4: The synthesis of alcohols **5a**–**f** via oxo functionality reduction (products with yields); Scheme S5: The coupling reaction between alcohols and FAs in the first synthetic route (products with yields); Scheme S6: The synthesis of FAHFAs through saponification of esters (products with yields); Scheme S7: Synthetic pathway for the conversion of *α,ω*-commercially available diols to *ω*-alkenyl alcohols (products with yields); Scheme S8: The photochemical hydroacylation reaction of *ω*-alkenyl alcohols and aldehydes (products with yields); Scheme S9: The synthesis of alcohols **14a**–**e** via oxo functionality reduction (products with yields); Scheme S10: The coupling reaction between alcohols and FAs in the second synthetic route (products with yields); Scheme S11: The deprotection of *tert*-butyldimethylsilyl (TBDMS) group (products with yields); Scheme S12: The oxidation of alcohols **16a**–**n** to FAHFAs (products with yields); ^1^H NMR and ^13^C NMR spectra of the compounds synthesized.
