# Supplementary material for: Construction of a Library of Fatty Acid Esters of Hydroxy Fatty Acids
Source: Molecules. 2025 Jan 13;30(2):286. doi: 10.3390/molecules30020286 (PMC11767521; doi:10.3390/molecules30020286)
Supplement: Supplementary file 1 [file molecules-30-00286-s001.zip › molecules-3368098-supplementary.pdf]

Construction of a Library of Fatty Acid Esters of Hydroxy Fatty Acids

Olga G. Mountanea[1,2], **Charikleia S. Batsika**[1,2], **Christiana Mantzourani**[1,2], **Christoforos G. Kokotos**[1,2] **and George Kokotos**[1,2*]

[1] *Department of Chemistry, National and Kapodistrian University of Athens, Panepistimiopolis, Athens 15771, Greece*

[2] *Center of Excellence for Drug Design and Discovery, National and Kapodistrian University of Athens, Panepistimiopolis, Athens 15771, Greece*

SUPPLEMENTARY MATERIALS

	**Page**
**Schematic Overview of the Products Synthesized and the Experimental Steps with Yields Included**	**S2**
**NMR Spectra**	**S8**

Schematic Overview of the Products Synthesized and the Experimental Steps with Yields Included

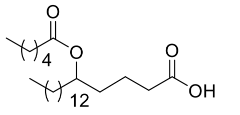
5-CAHSA
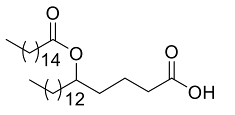
5-PAHSA
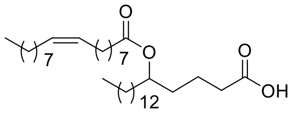
5-OAHSA

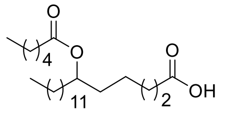
6-CAHSA
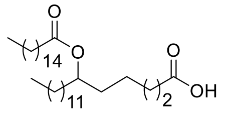
6-PAHSA
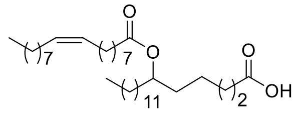
6-OAHSA

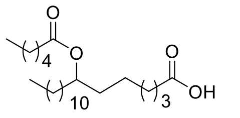
7-CAHSA
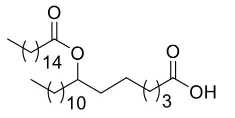
7-PAHSA
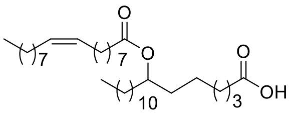
7-OAHSA

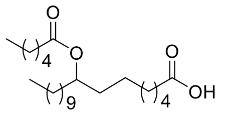
8-CAHSA
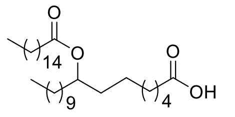
8-PAHSA
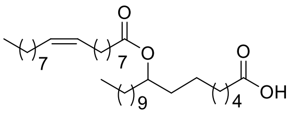
8-OAHSA

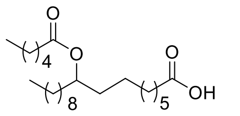
9-CAHSA
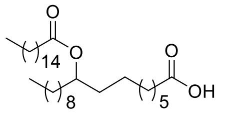
9-PAHSA
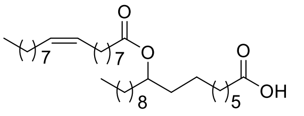
9-OAHSA

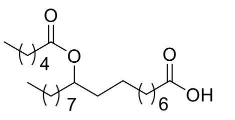
10-CAHSA
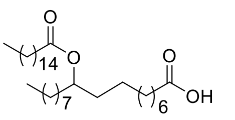
10-PAHSA
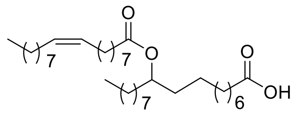
10-OAHSA

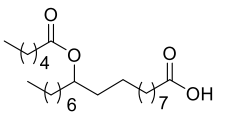
11-CAHSA
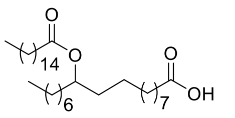
11-PAHSA
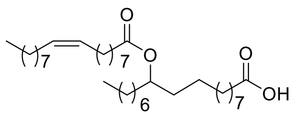
11-OAHSA

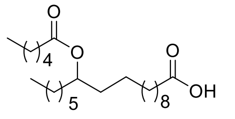
12-CAHSA
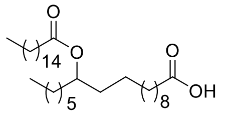
12-PAHSA
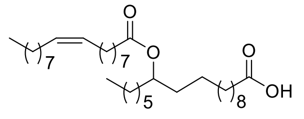
12-OAHSA

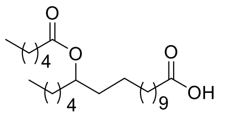
13-CAHSA
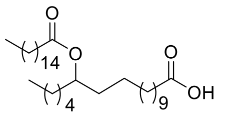
13-PAHSA
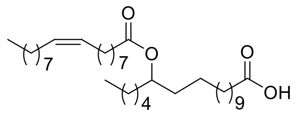
13-OAHSA

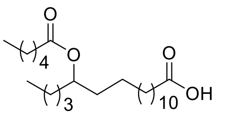
14-CAHSA
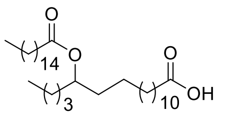
14-PAHSA
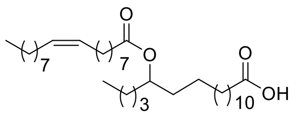
14-OAHSA

Scheme S1. Graphical representation of the structures of all final FAHFA compounds.

Experimental Steps with Yields Included

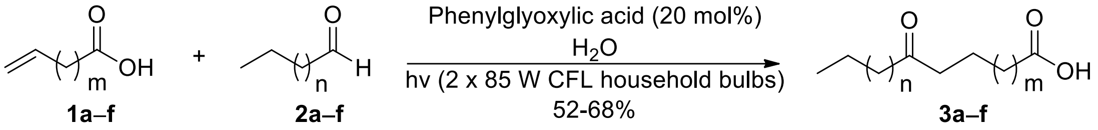

1	m
a	1
b	2
c	3
d	4
e	5
f	8

2	n
a	11
b	10
c	9
d	8
e	7
f	4

3	m	n	Yield (%)
a	1	11	53
b	2	10	63
c	3	9	52
d	4	8	54
e	5	7	67
f	8	4	68

Scheme S2. The photochemical hydroacylation reaction of aldehydes with alkenes to oxo fatty acids (products with yields).

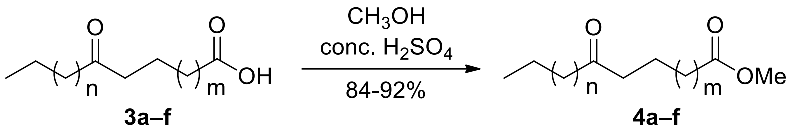

4	m	n	Yield (%)
a	1	11	89
b	2	10	86
c	3	9	92
d	4	8	84
e	5	7	88
f	8	4	85

Scheme S3. The synthesis of oxo methyl esters from oxo fatty acids (products with yields).

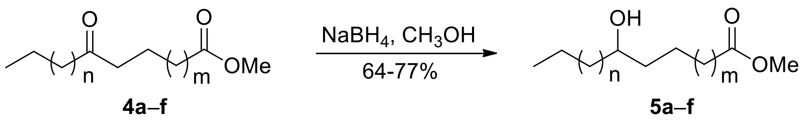

5	m	n	Yield (%)
a	1	11	64
b	2	10	76
c	3	9	72
d	4	8	69
e	5	7	67
f	8	4	77

Scheme S4. The synthesis of alcohols **5a–f** via oxo functionality reduction (products with yields).

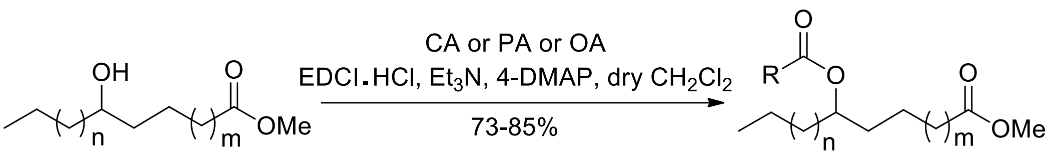

6	m	n	R	Yield (%)
a	1	11	$CH_3(CH_2)_4$-	77
b	1	11	$CH_3(CH_2)_{14}$-	75
c	1	11	$CH_3(CH_2)_7CH{=}CH(CH_2)_7$-	74
d	2	10	$CH_3(CH_2)_4$-	74
e	2	10	$CH_3(CH_2)_{14}$-	79
f	2	10	$CH_3(CH_2)_7CH{=}CH(CH_2)_7$-	81
g	3	9	$CH_3(CH_2)_4$-	76
h	3	9	$CH_3(CH_2)_{14}$-	77
i	3	9	$CH_3(CH_2)_7CH{=}CH(CH_2)_7$-	81
j	4	8	$CH_3(CH_2)_4$-	82
k	4	8	$CH_3(CH_2)_{14}$-	85
l	4	8	$CH_3(CH_2)_7CH{=}CH(CH_2)_7$-	84
m	5	7	$CH_3(CH_2)_{14}$-	73
n	8	4	$CH_3(CH_2)_4$-	75
o	8	4	$CH_3(CH_2)_{14}$-	77
p	8	4	$CH_3(CH_2)_7CH{=}CH(CH_2)_7$-	76

Scheme S5. The coupling reaction between alcohols and FAs in the first synthetic route (products with yields).

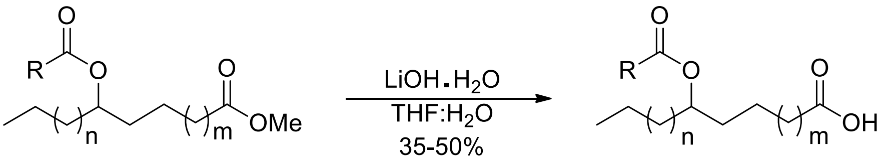

7	m	n	R	Yield (%)
a	1	11	$CH_3(CH_2)_4$-	35
b	1	11	$CH_3(CH_2)_{14}$-	42
c	1	11	$CH_3(CH_2)_7CH{=}CH(CH_2)_7$-	41
d	2	10	$CH_3(CH_2)_4$-	39
e	2	10	$CH_3(CH_2)_{14}$-	46
f	2	10	$CH_3(CH_2)_7CH{=}CH(CH_2)_7$-	50
g	3	9	$CH_3(CH_2)_4$-	38
h	3	9	$CH_3(CH_2)_{14}$-	48
i	3	9	$CH_3(CH_2)_7CH{=}CH(CH_2)_7$-	49
j	4	8	$CH_3(CH_2)_4$-	42
k	4	8	$CH_3(CH_2)_{14}$-	39
l	4	8	$CH_3(CH_2)_7CH{=}CH(CH_2)_7$-	43
m	5	7	$CH_3(CH_2)_{14}$-	45
n	8	4	$CH_3(CH_2)_4$-	37
o	8	4	$CH_3(CH_2)_{14}$-	40
p	8	4	$CH_3(CH_2)_7CH{=}CH(CH_2)_7$-	36

Scheme S6. The synthesis of FAHFAs through saponification of esters (products with yields).

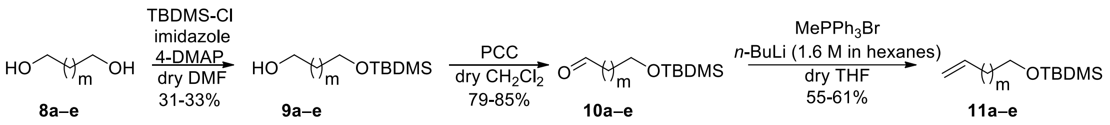

		Yield (%)		
**8–11**	m	**9**	**10**	**11**
**a**	5	32	85	55
**b**	6	33	85	58
**c**	7	31	83	61
**d**	9	32	81	59
**e**	10	31	79	56

Scheme S7. Synthetic pathway for the conversion of *α*,*ω*-commercially available diols to *ω*-alkenyl alcohols (products with yields).

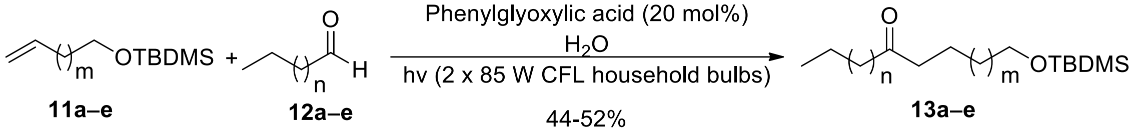

13	m	n	Yield (%)
**a**	5	7	44
**b**	6	6	52
**c**	7	5	49
**d**	9	3	52
**e**	10	2	46

Scheme S8. The photochemical hydroacylation reaction of *ω*-alkenyl alcohols and aldehydes (products with yields).

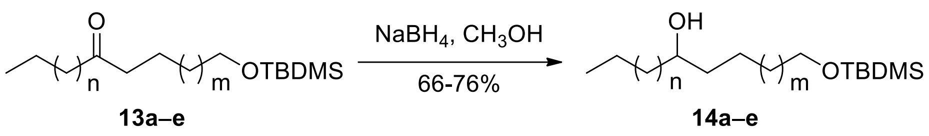

14	m	n	Yield (%)
**a**	5	7	74
**b**	6	6	76
**c**	7	5	71
**d**	9	3	66
**e**	10	2	70

Scheme S9. The synthesis of alcohols **14a–e** via oxo functionality reduction (products with yields).

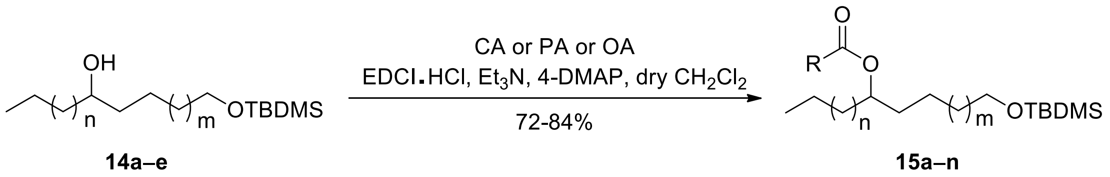

15	m	n	R	Yield (%)
**a**	5	7	$CH_3(CH_2)_4$-	77
**b**	5	7	$CH_3(CH_2)_7CH{=}CH(CH_2)_7$-	84
**c**	6	6	$CH_3(CH_2)_4$-	79
**d**	6	6	$CH_3(CH_2)_{14}$-	84
**e**	6	6	$CH_3(CH_2)_7CH{=}CH(CH_2)_7$-	83
**f**	7	5	$CH_3(CH_2)_4$-	79
**g**	7	5	$CH_3(CH_2)_{14}$-	74
**h**	7	5	$CH_3(CH_2)_7CH{=}CH(CH_2)_7$-	82
**i**	9	3	$CH_3(CH_2)_4$-	72
**j**	9	3	$CH_3(CH_2)_{14}$-	76
**k**	9	3	$CH_3(CH_2)_7CH{=}CH(CH_2)_7$-	81
**l**	10	2	$CH_3(CH_2)_4$-	80
**m**	10	2	$CH_3(CH_2)_{14}$-	80
**n**	10	2	$CH_3(CH_2)_7CH{=}CH(CH_2)_7$-	82

Scheme S10. The coupling reaction between alcohols and FAs in the second synthetic route (products with yields).

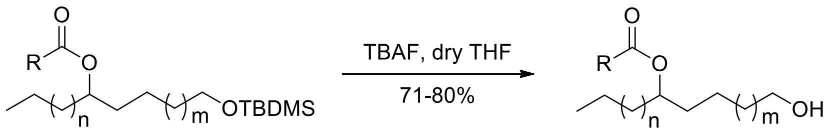

15a–n **16a–n**

16	m	n	R	Yield (%)
**a**	5	7	$CH_3(CH_2)_4$-	76
**b**	5	7	$CH_3(CH_2)_7CH{=}CH(CH_2)_7$-	74
**c**	6	6	$CH_3(CH_2)_4$-	79
**d**	6	6	$CH_3(CH_2)_{14}$-	75
**e**	6	6	$CH_3(CH_2)_7CH{=}CH(CH_2)_7$-	73
**f**	7	5	$CH_3(CH_2)_4$-	78
**g**	7	5	$CH_3(CH_2)_{14}$-	75
**h**	7	5	$CH_3(CH_2)_7CH{=}CH(CH_2)_7$-	79
**i**	9	3	$CH_3(CH_2)_4$-	71
**j**	9	3	$CH_3(CH_2)_{14}$-	71
**k**	9	3	$CH_3(CH_2)_7CH{=}CH(CH_2)_7$-	76
**l**	10	2	$CH_3(CH_2)_4$-	78
**m**	10	2	$CH_3(CH_2)_{14}$-	80
**n**	10	2	$CH_3(CH_2)_7CH{=}CH(CH_2)_7$-	79

Scheme S11. The deprotection of *tert*-butyldimethylsilyl (TBDMS) group (products with yields).

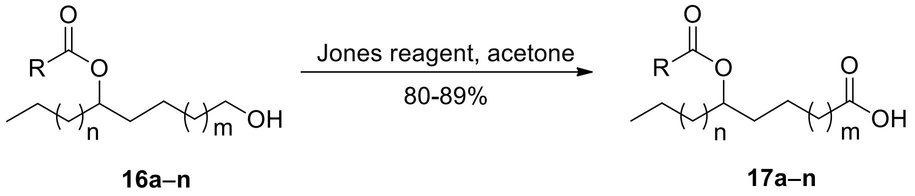

17	m	n	R	Yield (%)
**a**	5	7	$CH_3(CH_2)_4$-	84
**b**	5	7	$CH_3(CH_2)_7CH=CH(CH_2)_7$-	82
**c**	6	6	$CH_3(CH_2)_4$-	84
**d**	6	6	$CH_3(CH_2)_{14}$-	86
**e**	6	6	$CH_3(CH_2)_7CH=CH(CH_2)_7$-	83
**f**	7	5	$CH_3(CH_2)_4$-	80
**g**	7	5	$CH_3(CH_2)_{14}$-	85
**h**	7	5	$CH_3(CH_2)_7CH=CH(CH_2)_7$-	86
**i**	9	3	$CH_3(CH_2)_4$-	82
**j**	9	3	$CH_3(CH_2)_{14}$-	89
**k**	9	3	$CH_3(CH_2)_7CH=CH(CH_2)_7$-	87
**l**	10	2	$CH_3(CH_2)_4$-	81
**m**	10	2	$CH_3(CH_2)_{14}$-	80
**n**	10	2	$CH_3(CH_2)_7CH=CH(CH_2)_7$-	85

Scheme S12. The oxidation of alcohols **16a-n** to FAHFAs (products with yields).

NMR Spectra

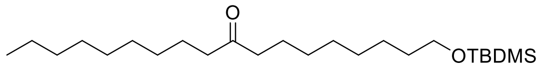

(13a)

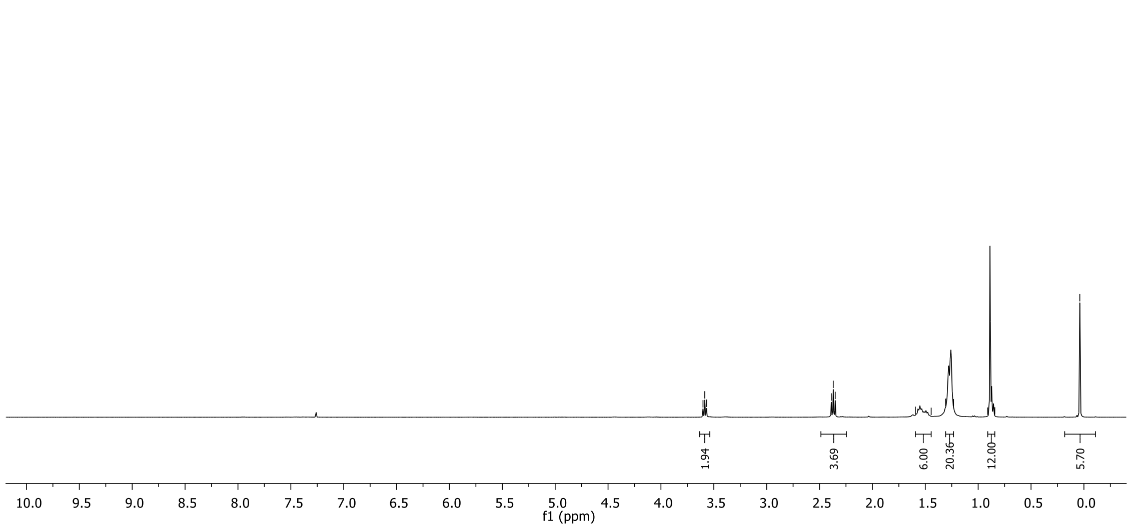

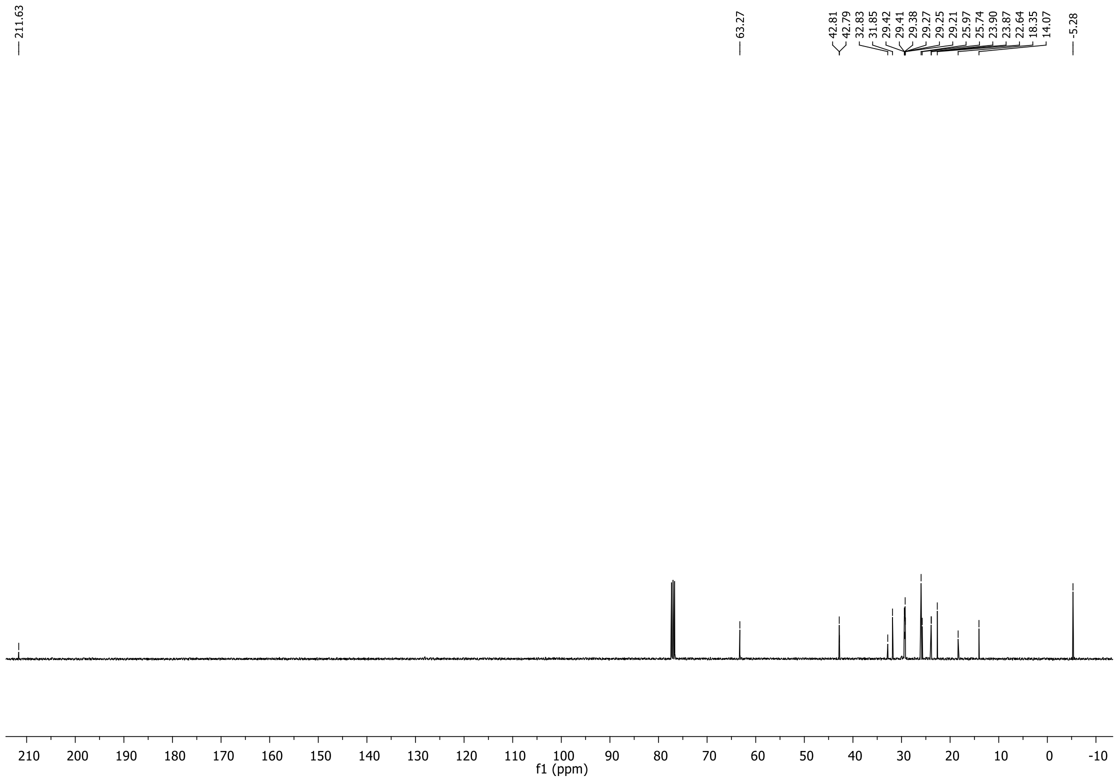

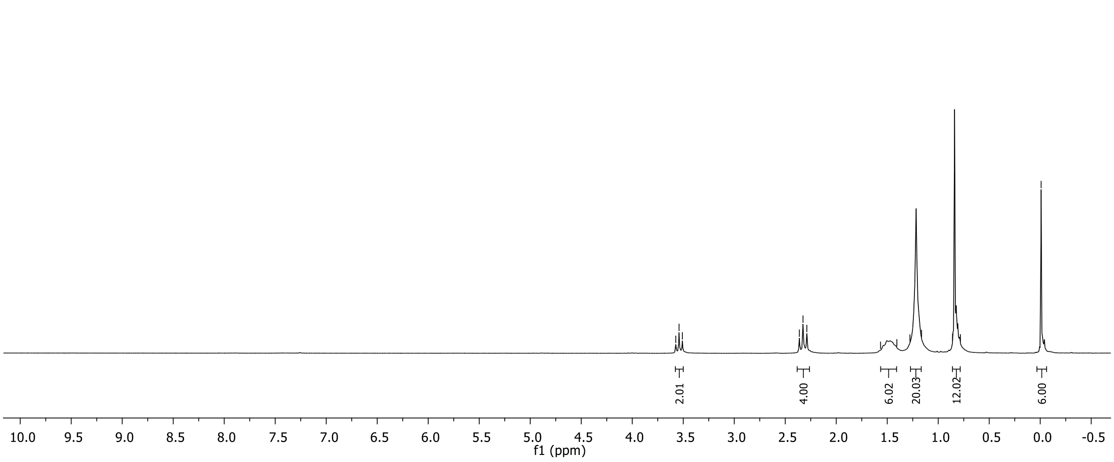
3.57
3.54
3.51
2.36
2.33
2.29
1.57
1.41
1.28
1.16
0.86
0.78
-0.01
2.01
4.00
6.02
20.03
12.02
6.00
10.0 9.5 9.0 8.5 8.0 7.5 7.0 6.5 6.0 5.5 5.0 4.5 4.0 3.5 3.0 2.5 2.0 1.5 1.0 0.5 0.0 -0.5
f1 (ppm)

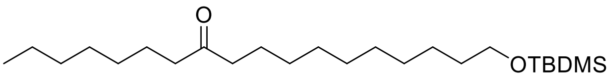
O
OTBDMS

(13c)

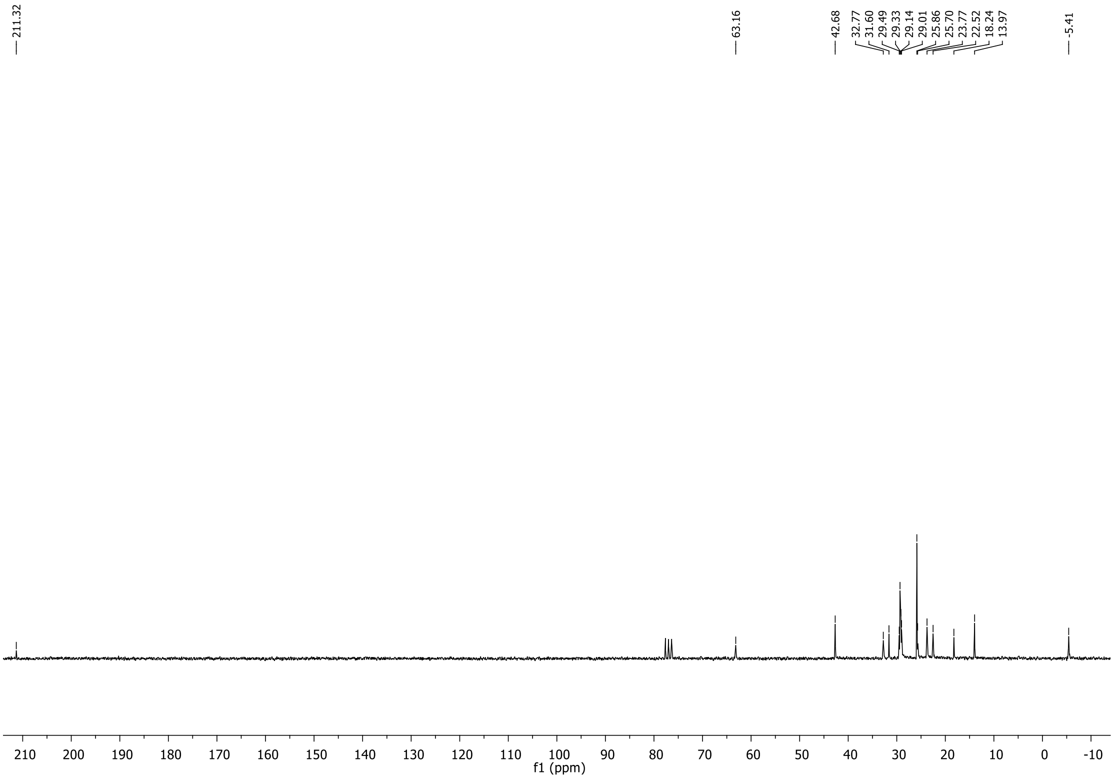
211.32
63.16
42.68
32.77
31.60
29.49
29.33
29.14
29.01
25.86
25.70
23.77
22.52
18.24
13.97
-5.41
210 200 190 180 170 160 150 140 130 120 110 100 90 80 70 60 50 40 30 20 10 0 -10
f1 (ppm)

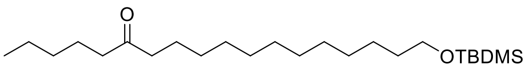

(13d)

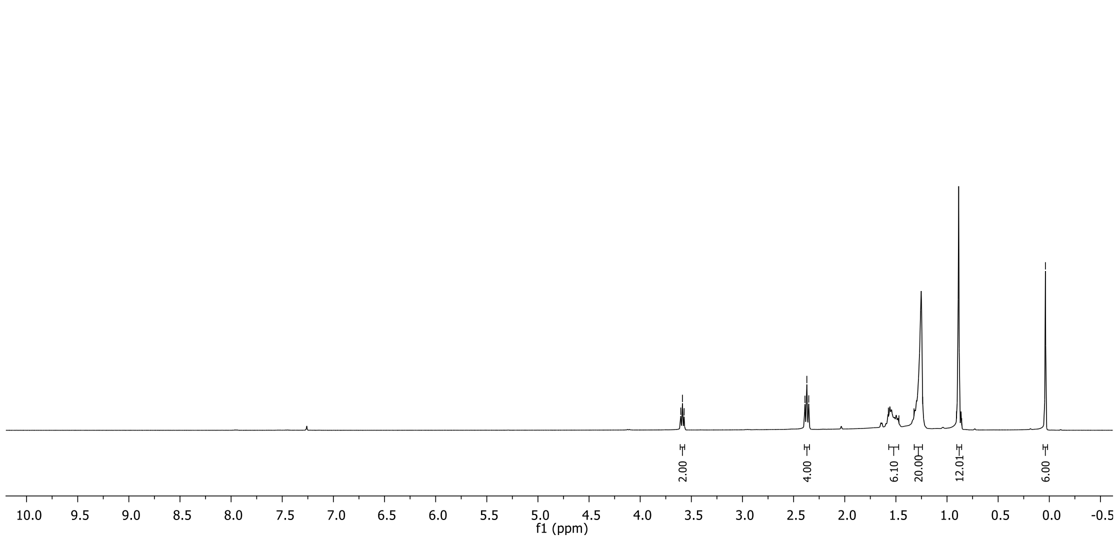

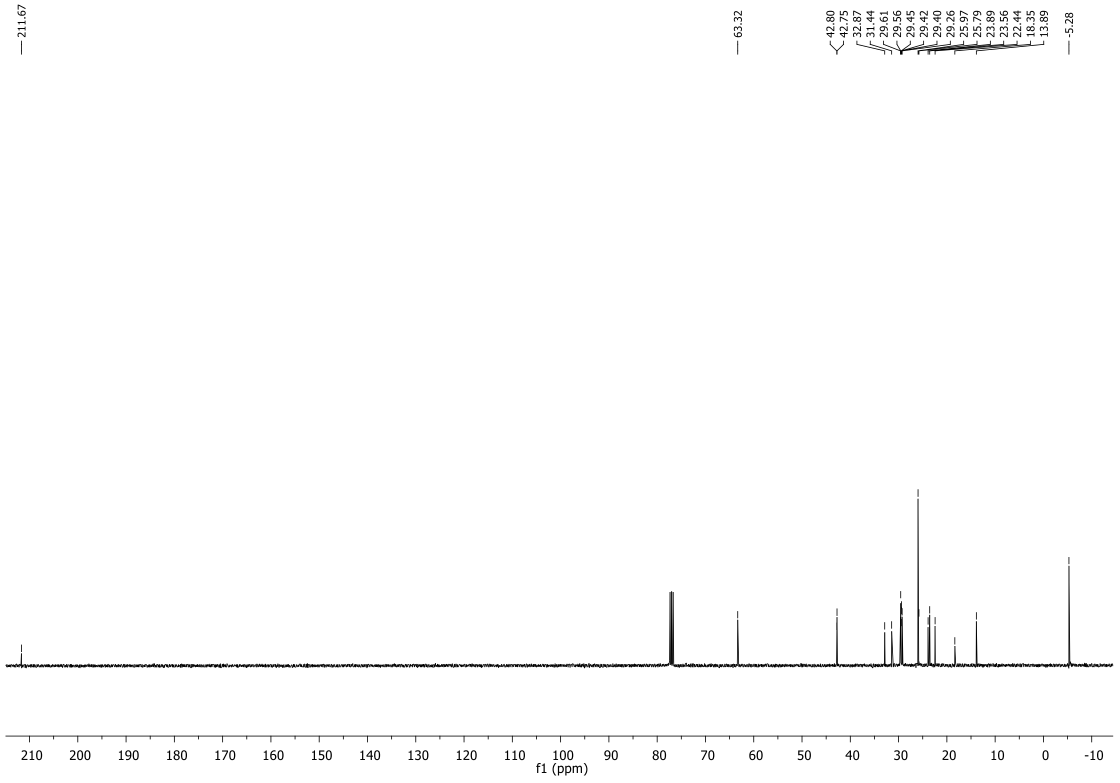

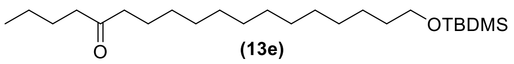
O
OTBDMS

(13e)

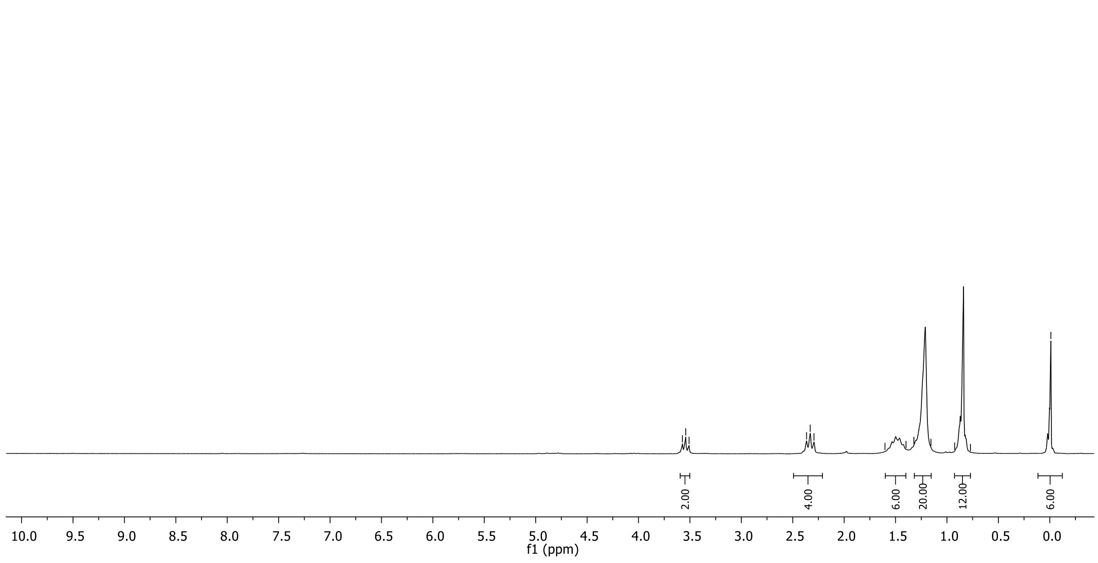
3.57
3.54
3.51
2.37
2.33
2.29
1.60
1.40
1.32
1.16
0.93
0.77
-0.01
2.00
4.00
6.00
20.00
12.00
6.00
10.0 9.5 9.0 8.5 8.0 7.5 7.0 6.5 6.0 5.5 5.0 4.5 4.0 3.5 3.0 2.5 2.0 1.5 1.0 0.5 0.0
f1 (ppm)

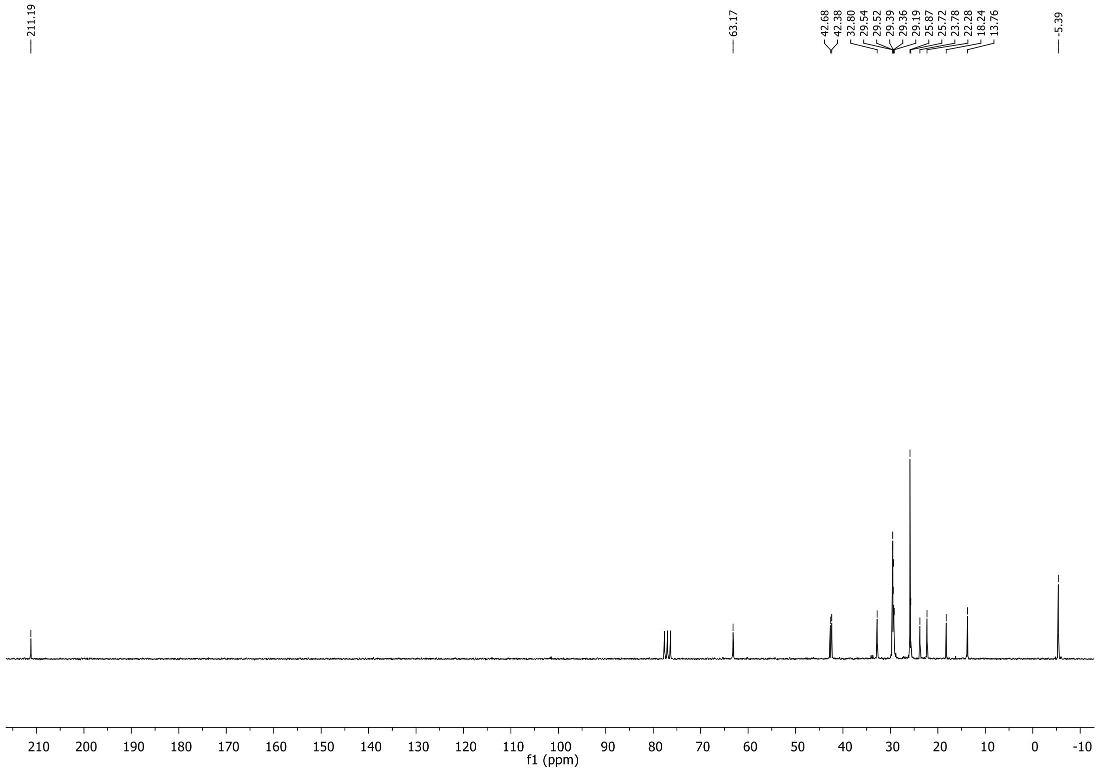
211.19
63.17
42.68
42.38
32.80
29.54
29.52
29.39
29.36
29.19
25.87
25.72
23.78
22.28
18.24
13.76
-5.39
210 200 190 180 170 160 150 140 130 120 110 100 90 80 70 60 50 40 30 20 10 0 -10
f1 (ppm)

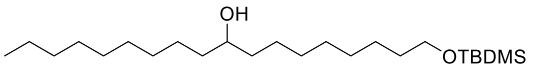
OH
OTBDMS

(14a)

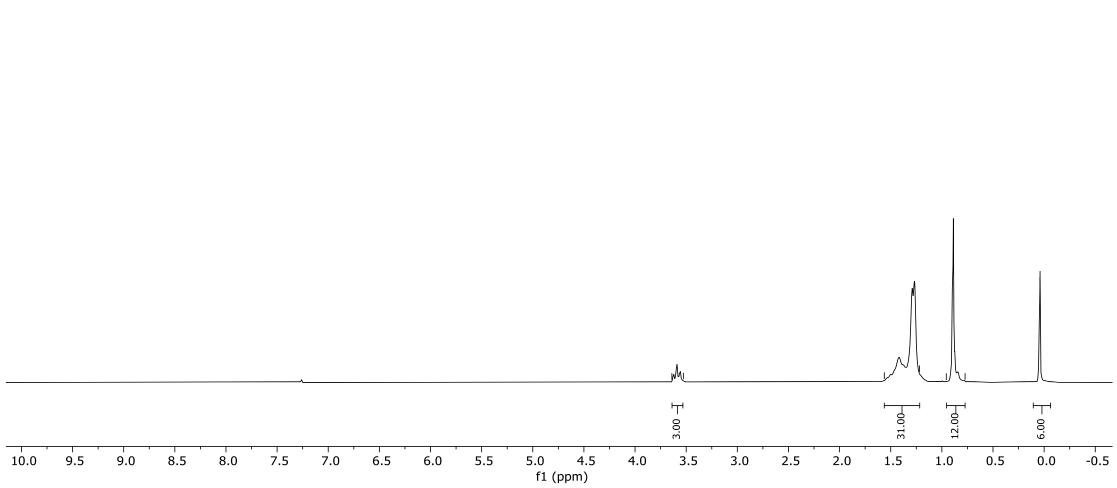
3.64
3.53
1.56
1.22
0.96
0.77
0.04
3.00
31.00
12.00
6.00
10.0 9.5 9.0 8.5 8.0 7.5 7.0 6.5 6.0 5.5 5.0 4.5 4.0 3.5 3.0 2.5 2.0 1.5 1.0 0.5 0.0 -0.5
f1 (ppm)

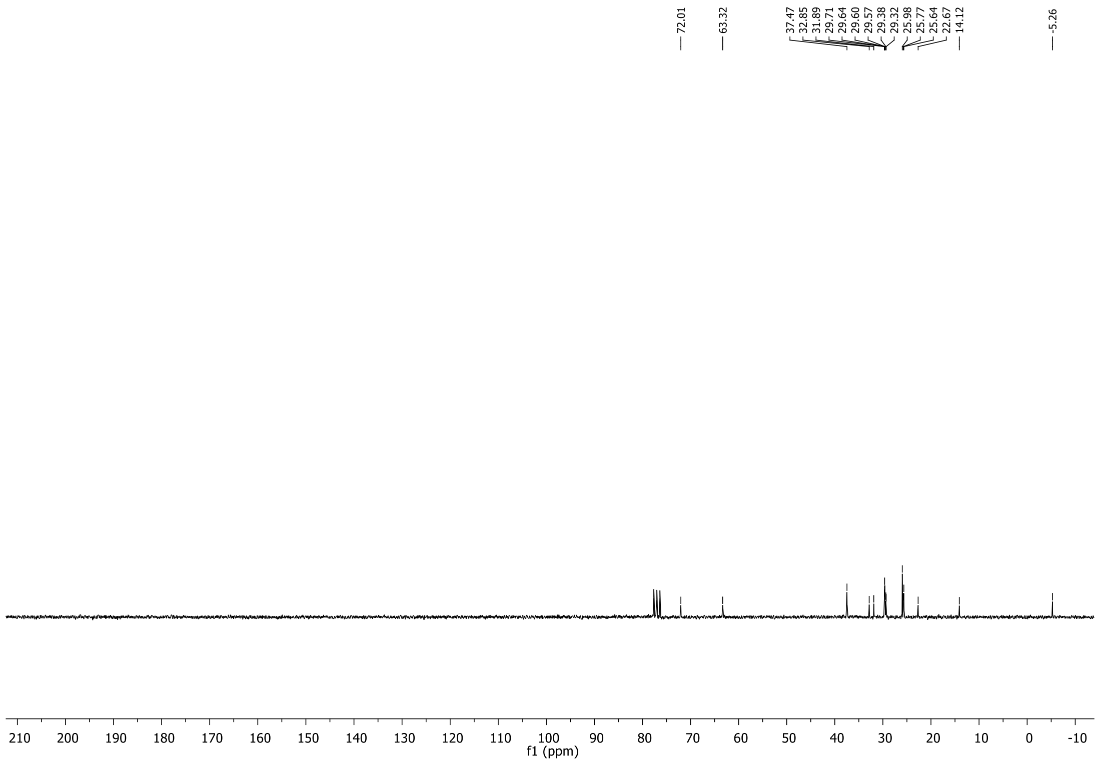
72.01
63.32
37.47
32.85
31.89
29.71
29.64
29.60
29.57
29.38
29.32
25.98
25.77
25.64
22.67
14.12
-5.26
210 200 190 180 170 160 150 140 130 120 110 100 90 80 70 60 50 40 30 20 10 0 -10
f1 (ppm)

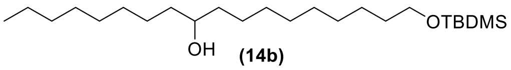
OTBDMS
OH

(14b)

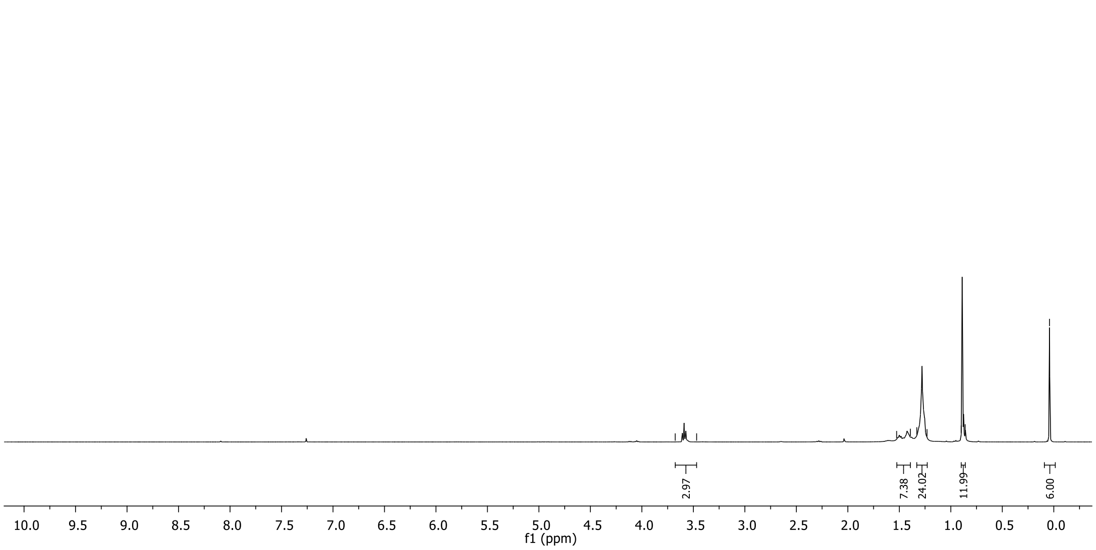
3.68
3.47
1.53
1.39
1.33
1.23
0.90
0.86
0.04
2.97
7.38
24.02
11.99
6.00
10.0 9.5 9.0 8.5 8.0 7.5 7.0 6.5 6.0 5.5 5.0 4.5 4.0 3.5 3.0 2.5 2.0 1.5 1.0 0.5 0.0
f1 (ppm)

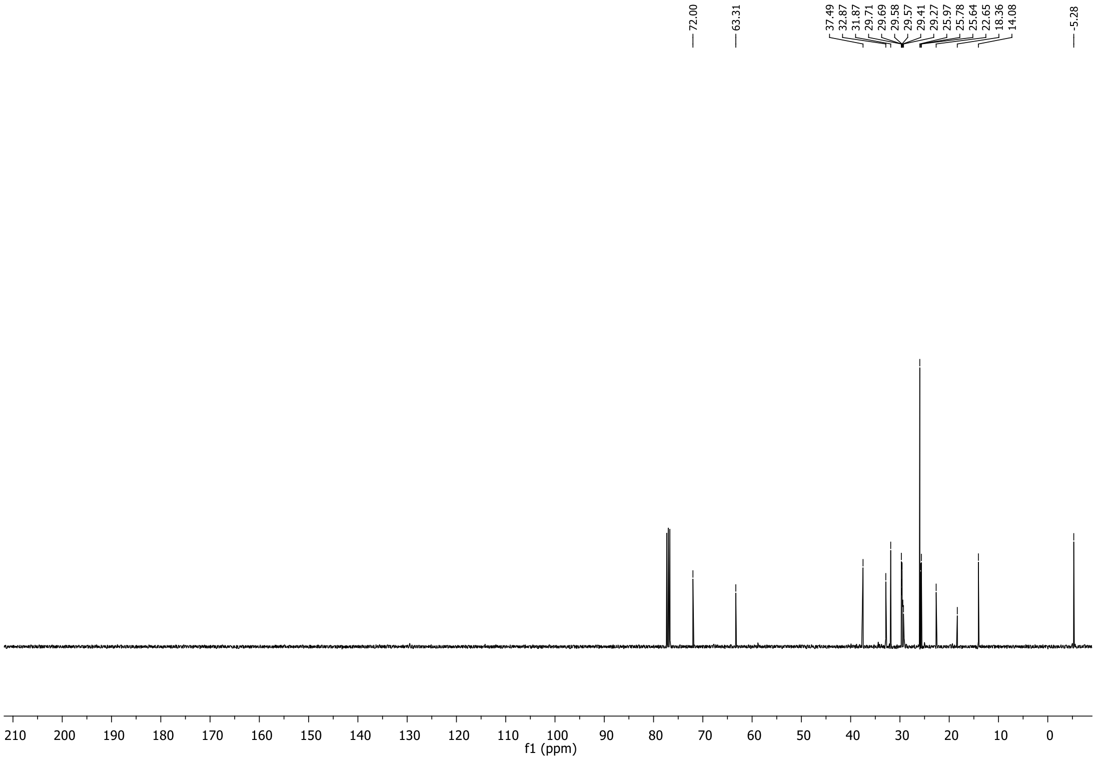
72.00
63.31
37.49
32.87
31.87
29.71
29.69
29.58
29.57
29.41
29.27
25.97
25.78
25.64
22.65
18.36
14.08
-5.28
210 200 190 180 170 160 150 140 130 120 110 100 90 80 70 60 50 40 30 20 10 0
f1 (ppm)

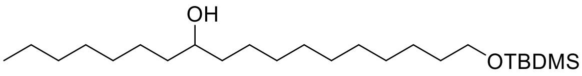
OH
OTBDMS

(14c)

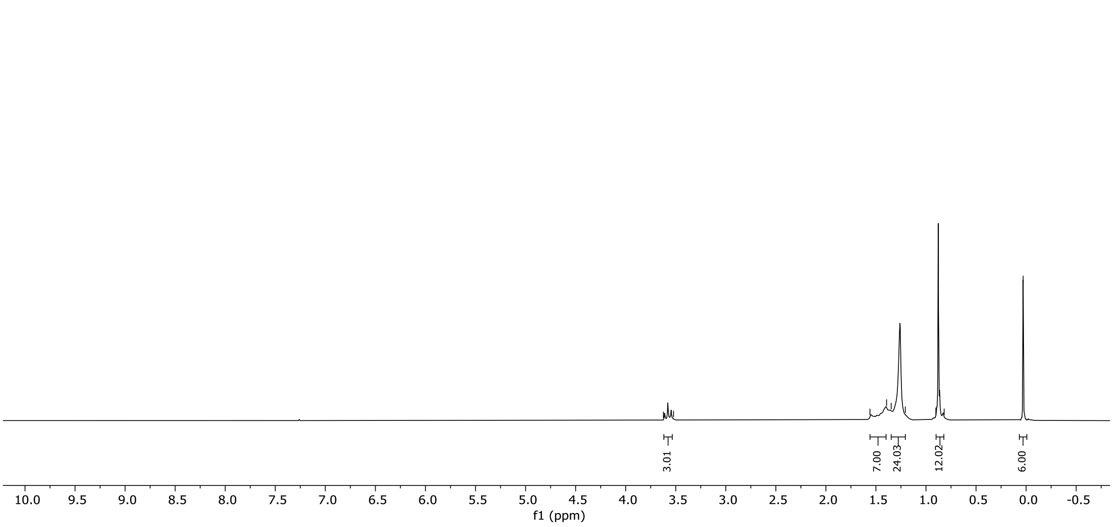
3.62
3.52
1.56
1.39
1.35
1.21
0.90
0.82
0.03
3.01
7.00
24.03
12.02
6.00
10.0 9.5 9.0 8.5 8.0 7.5 7.0 6.5 6.0 5.5 5.0 4.5 4.0 3.5 3.0 2.5 2.0 1.5 1.0 0.5 0.0 -0.5
f1 (ppm)

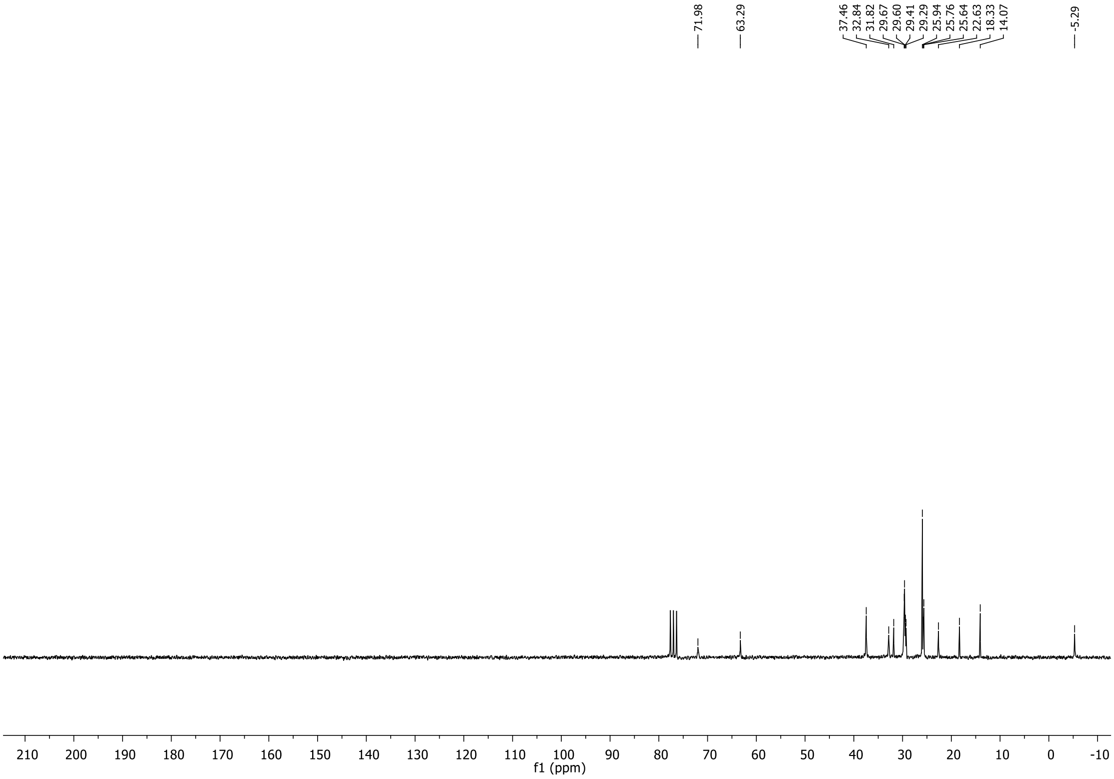
71.98
63.29
37.46
32.84
31.82
29.67
29.60
29.41
29.29
25.94
25.76
25.64
22.63
18.33
14.07
-5.29
210 200 190 180 170 160 150 140 130 120 110 100 90 80 70 60 50 40 30 20 10 0 -10
f1 (ppm)

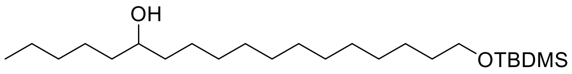
OH
OTBDMS

(14d)

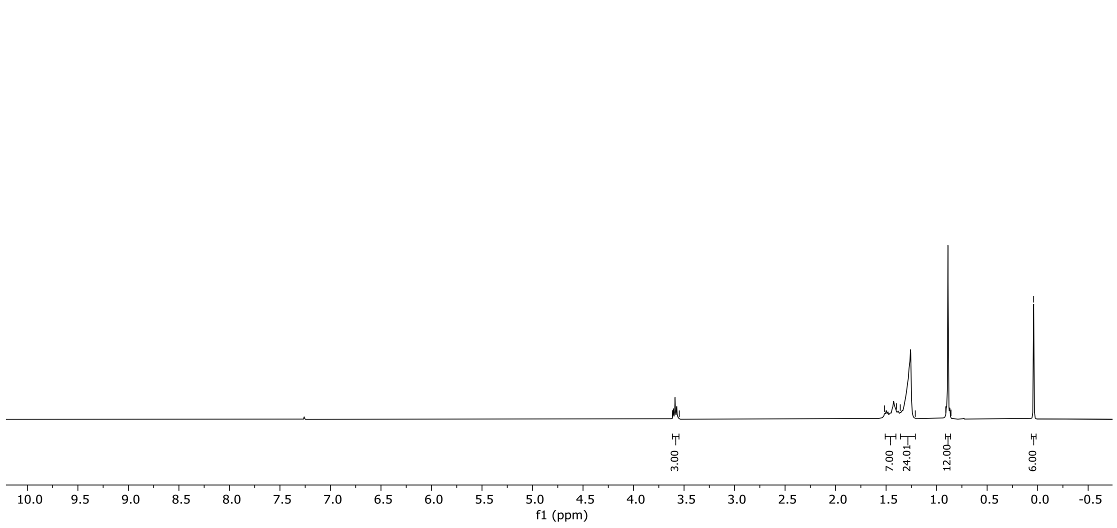
3.62
3.55
1.52
1.40
1.36
1.21
0.91
0.86
0.04
3.00
7.00
24.01
12.00
6.00
f1 (ppm)

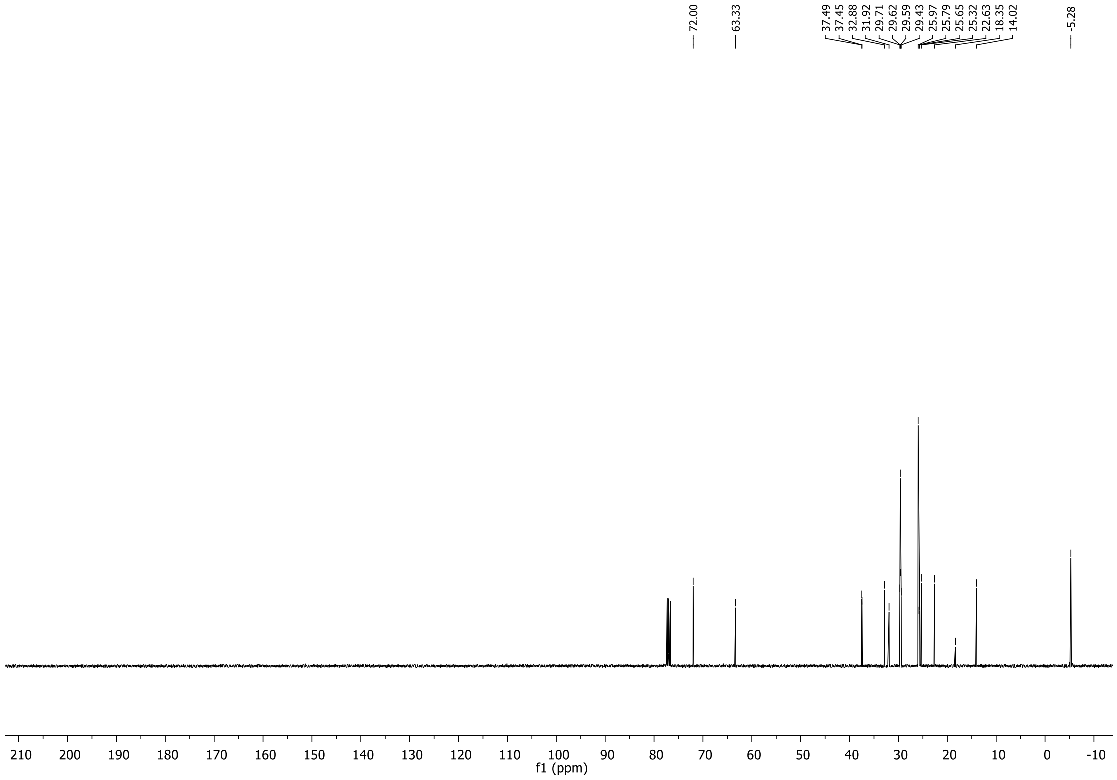
72.00
63.33
37.49
37.45
32.88
31.92
29.71
29.62
29.59
29.43
25.97
25.79
25.65
25.32
22.63
18.35
14.02
-5.28
f1 (ppm)

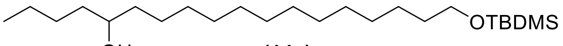
OH
OTBDMS

(14e)

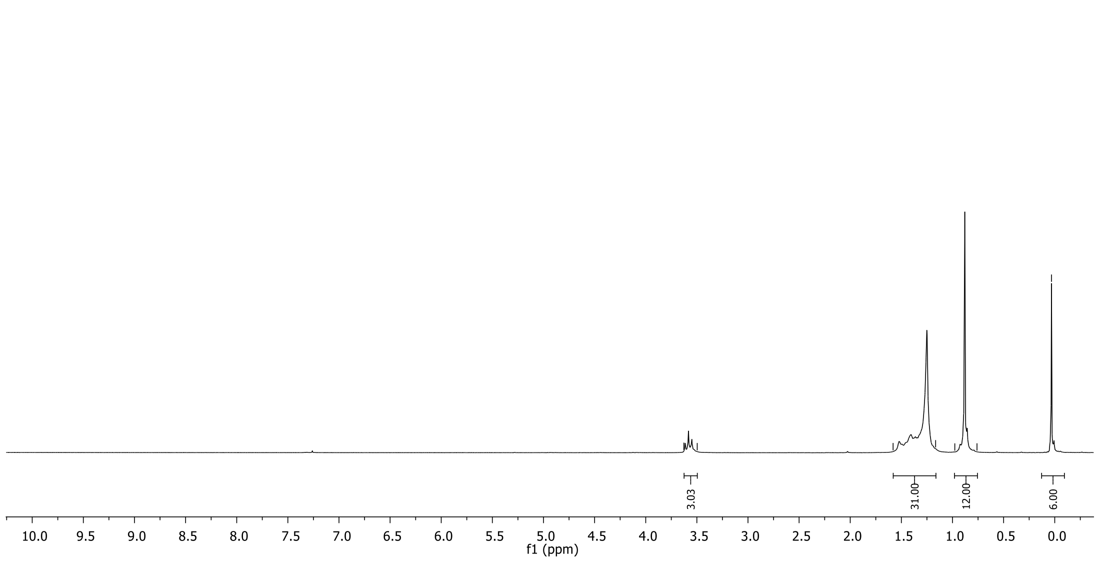
3.63
3.50
1.58
1.16
0.98
0.76
0.03
3.03
31.00
12.00
6.00
10.0 9.5 9.0 8.5 8.0 7.5 7.0 6.5 6.0 5.5 5.0 4.5 4.0 3.5 3.0 2.5 2.0 1.5 1.0 0.5 0.0
f1 (ppm)

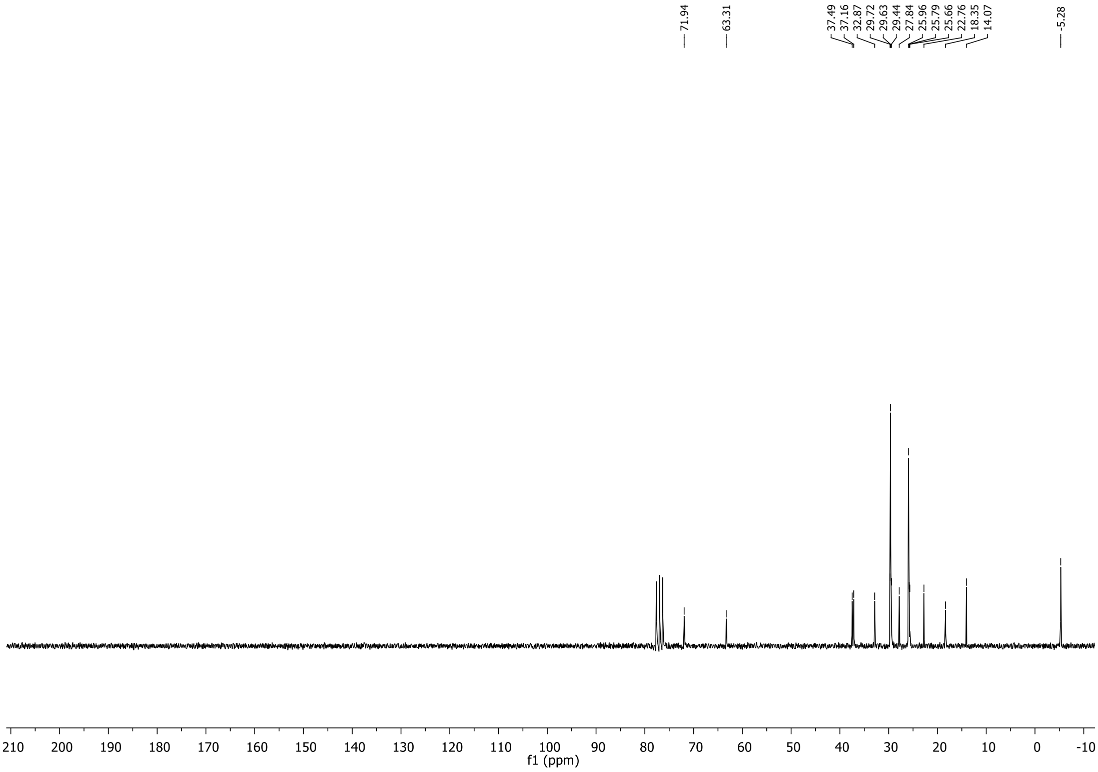
71.94
63.31
37.49
37.16
32.87
29.72
29.63
29.44
27.84
25.96
25.79
25.66
22.76
18.35
14.07
-5.28
210 200 190 180 170 160 150 140 130 120 110 100 90 80 70 60 50 40 30 20 10 0 -10
f1 (ppm)

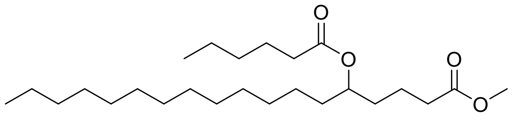
O
O
O
O

(6a)

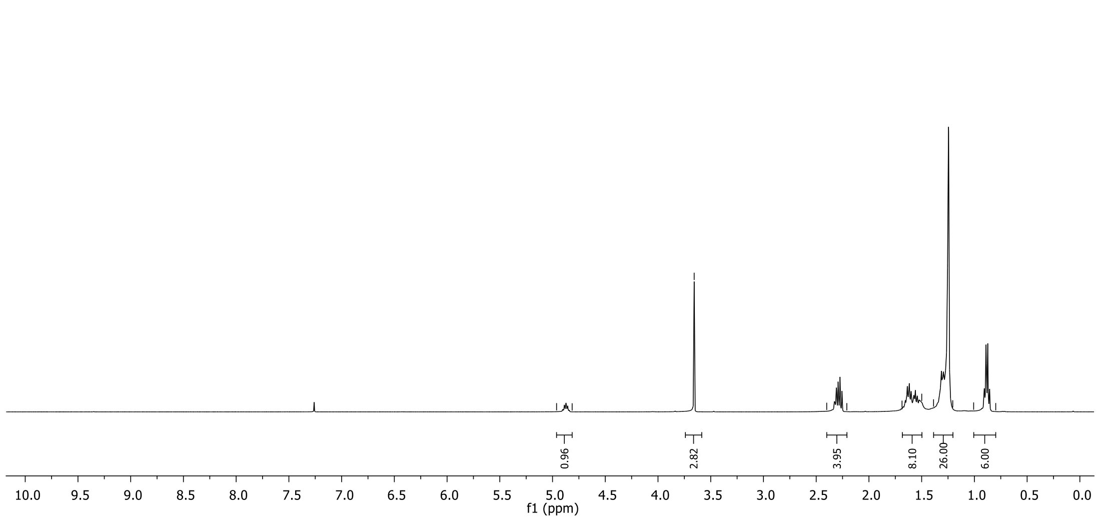
4.96
4.81
3.66
2.40
2.21
1.69
1.50
1.39
1.21
1.01
0.80
0.96
2.82
3.95
8.10
26.00
6.00
10.0 9.5 9.0 8.5 8.0 7.5 7.0 6.5 6.0 5.5 5.0 4.5 4.0 3.5 3.0 2.5 2.0 1.5 1.0 0.5 0.0
f1 (ppm)

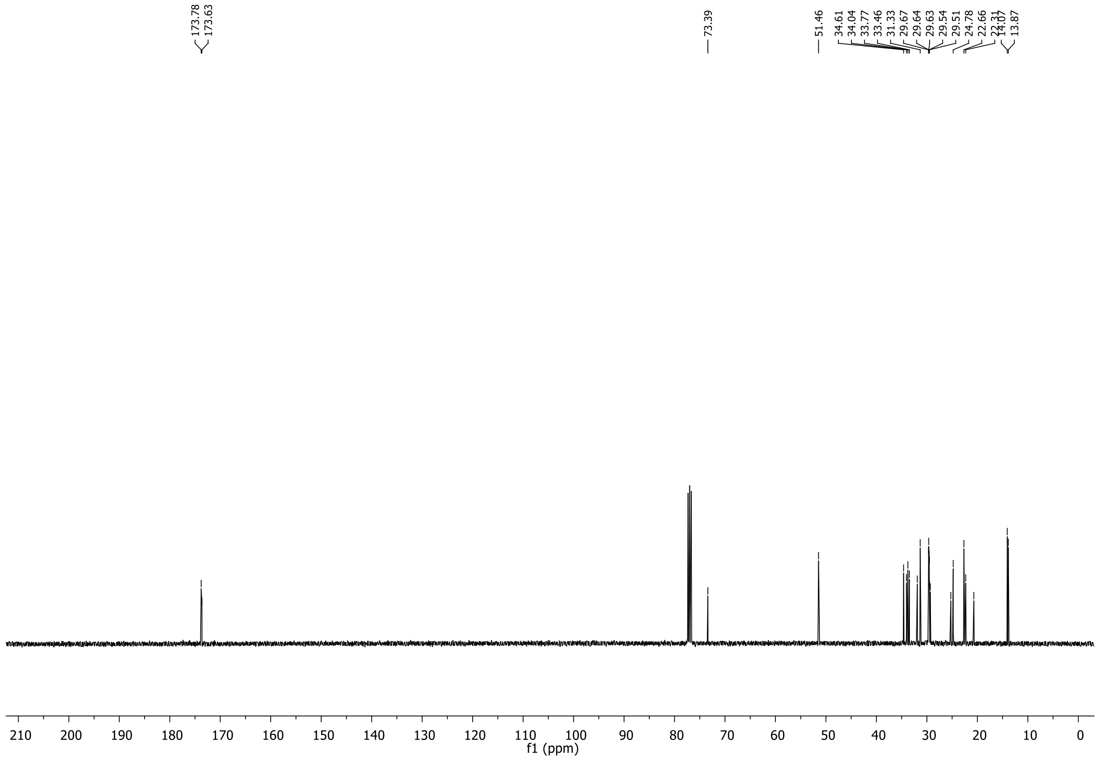
173.78
173.63
73.39
51.46
34.61
34.04
33.77
33.46
31.33
29.67
29.64
29.63
29.54
29.51
24.78
22.66
22.31
14.07
13.87
210 200 190 180 170 160 150 140 130 120 110 100 90 80 70 60 50 40 30 20 10 0
f1 (ppm)

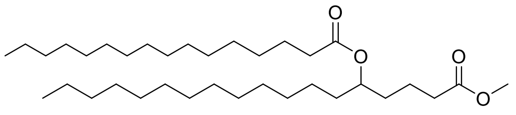

(6b)

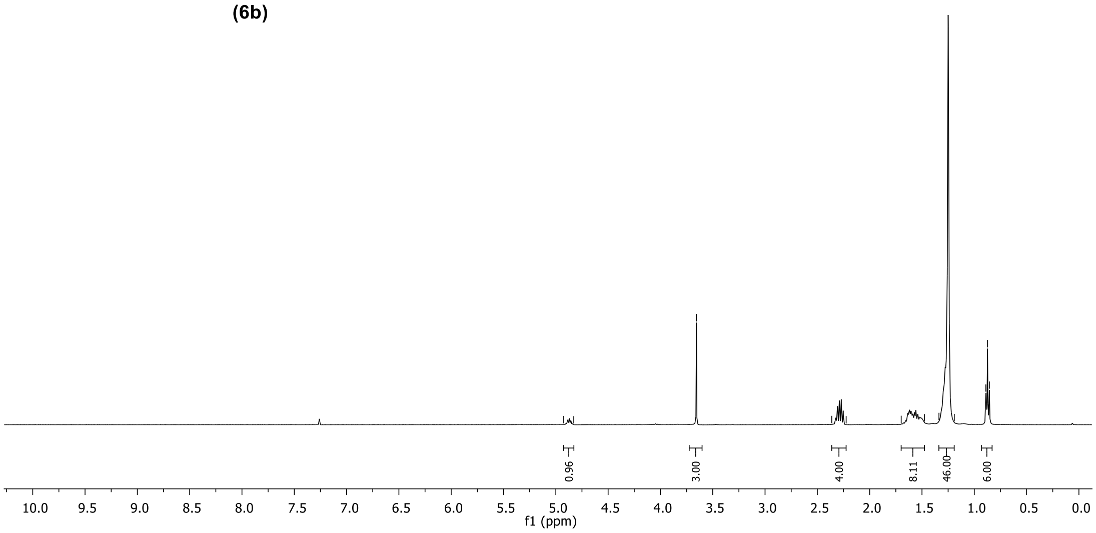
4.93
4.83
3.66
2.36
2.22
1.70
1.48
1.34
1.19
0.89
0.87
0.86
0.96
3.00
4.00
8.11
46.00
6.00
10.0 9.5 9.0 8.5 8.0 7.5 7.0 6.5 6.0 5.5 5.0 4.5 4.0 3.5 3.0 2.5 2.0 1.5 1.0 0.5 0.0
f1 (ppm)

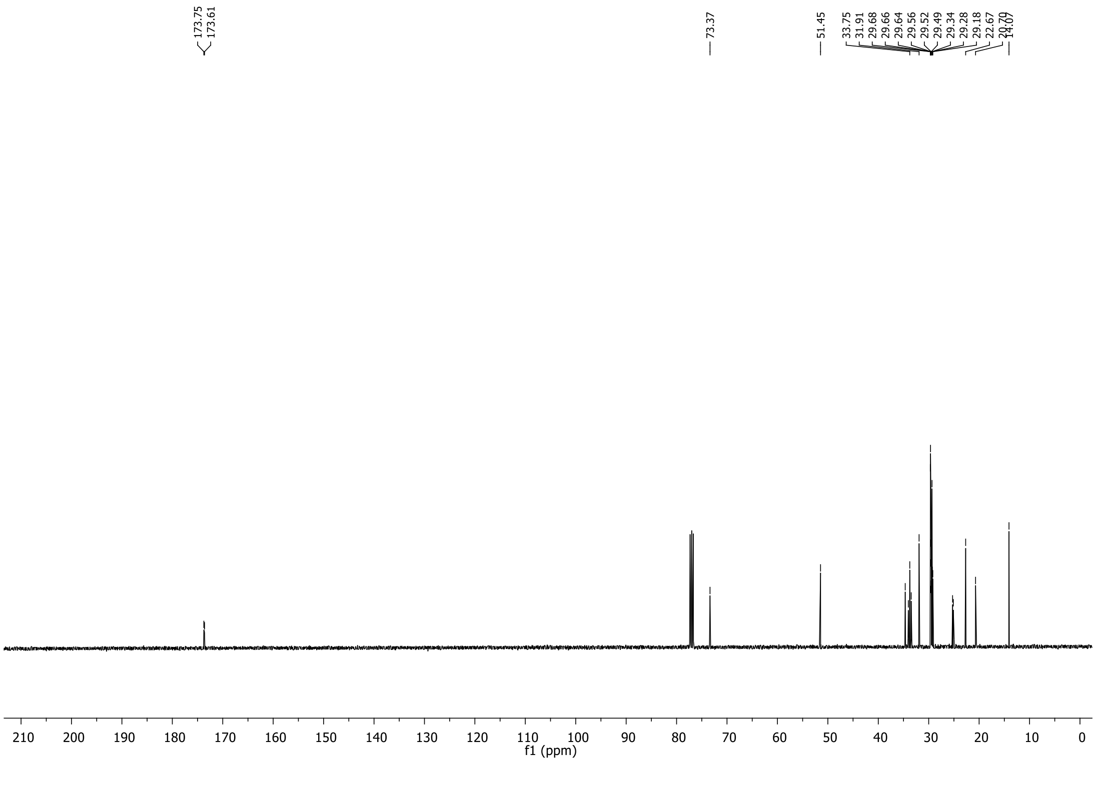
173.75
173.61
73.37
51.45
33.75
31.91
29.68
29.66
29.64
29.56
29.52
29.49
29.34
29.28
29.18
22.67
14.07
210 200 190 180 170 160 150 140 130 120 110 100 90 80 70 60 50 40 30 20 10 0
f1 (ppm)

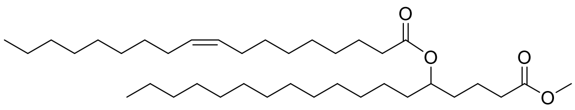

(6c)

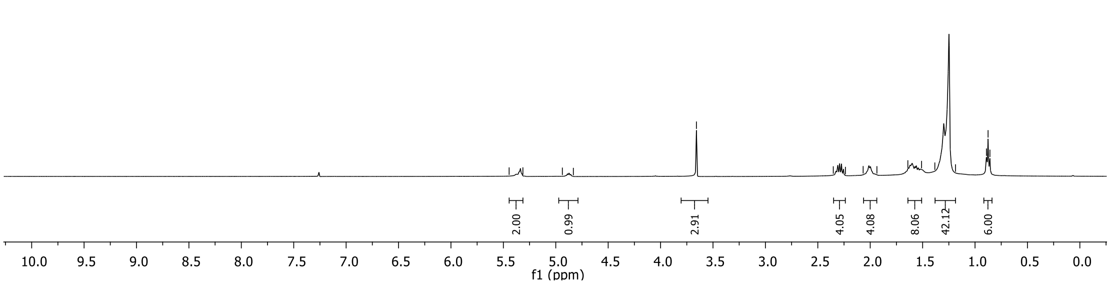
5.44
5.31
4.94
4.83
3.66
2.35
2.24
2.07
1.94
1.64
1.51
1.38
1.19
0.89
0.88
0.86
2.00
0.99
2.91
4.05
4.08
8.06
42.12
6.00
10.0 9.5 9.0 8.5 8.0 7.5 7.0 6.5 6.0 5.5 5.0 4.5 4.0 3.5 3.0 2.5 2.0 1.5 1.0 0.5 0.0
f1 (ppm)

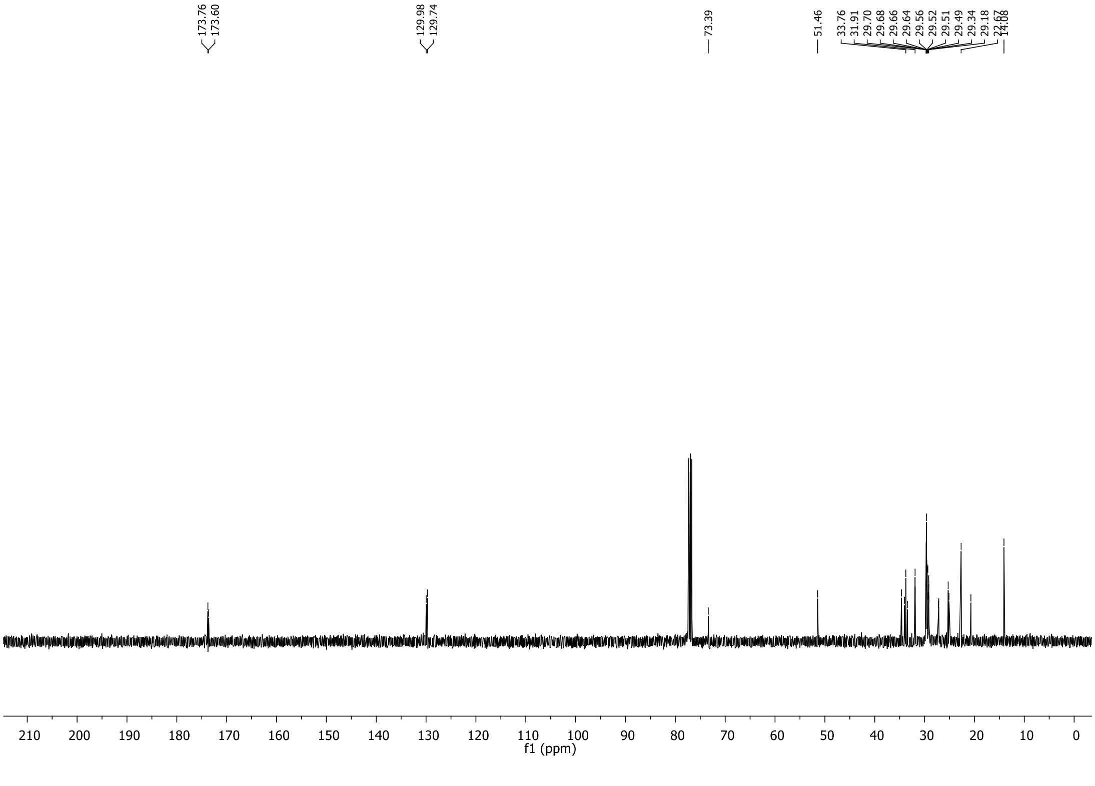
173.76
173.60
129.98
129.74
73.39
51.46
33.76
31.91
29.70
29.68
29.66
29.64
29.56
29.52
29.51
29.49
29.34
29.18
22.67
14.08
210 200 190 180 170 160 150 140 130 120 110 100 90 80 70 60 50 40 30 20 10 0
f1 (ppm)

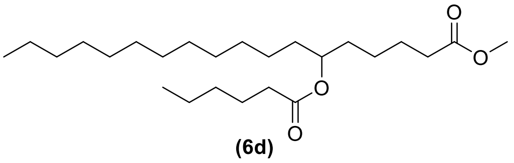
O
O
O
O

(6d)

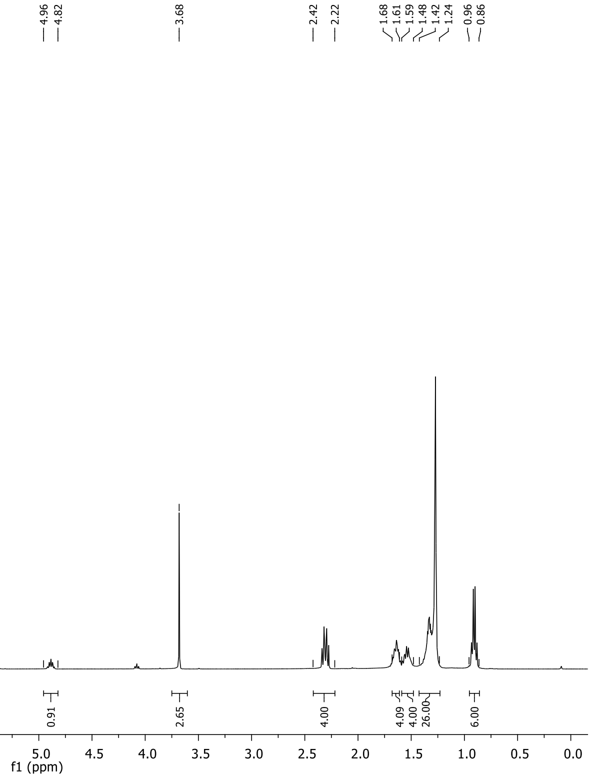
4.96
4.82
3.68
2.42
2.22
1.68
1.61
1.59
1.48
1.42
1.24
0.96
0.86
0.91
2.65
4.00
4.09
4.00
26.00
6.00
f1 (ppm)

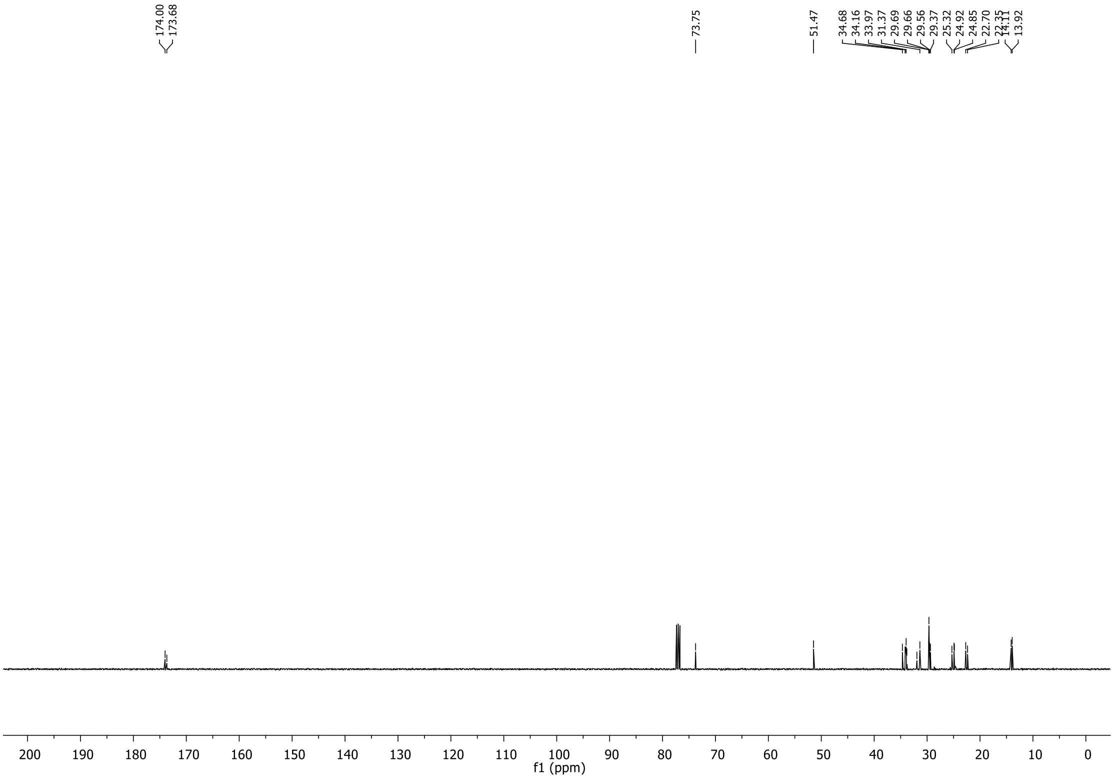
174.00
173.68
73.75
51.47
34.68
34.16
33.97
31.37
29.69
29.66
29.56
29.37
25.32
24.92
24.85
22.70
22.35
14.11
13.92
f1 (ppm)

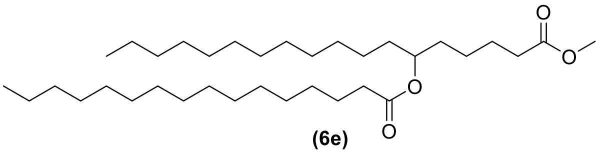

(6e)

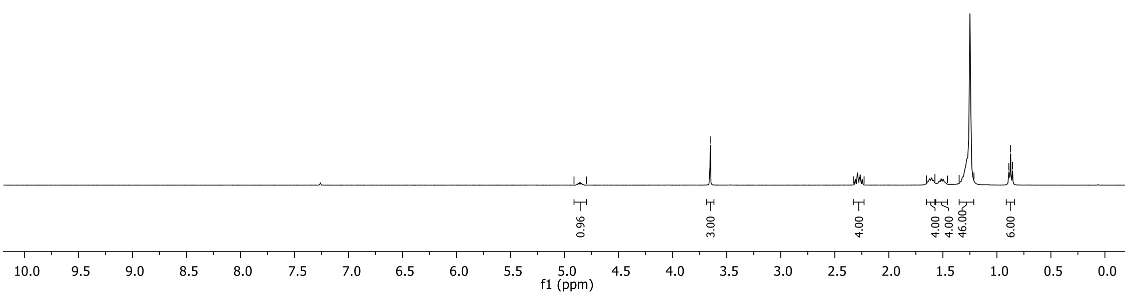
4.91
4.80
3.65
2.33
2.23
1.65
1.57
1.46
1.35
1.21
0.89
0.87
0.86
0.96
3.00
4.00
4.00
4.00
46.00
6.00
10.0 9.5 9.0 8.5 8.0 7.5 7.0 6.5 6.0 5.5 5.0 4.5 4.0 3.5 3.0 2.5 2.0 1.5 1.0 0.5 0.0
f1 (ppm)

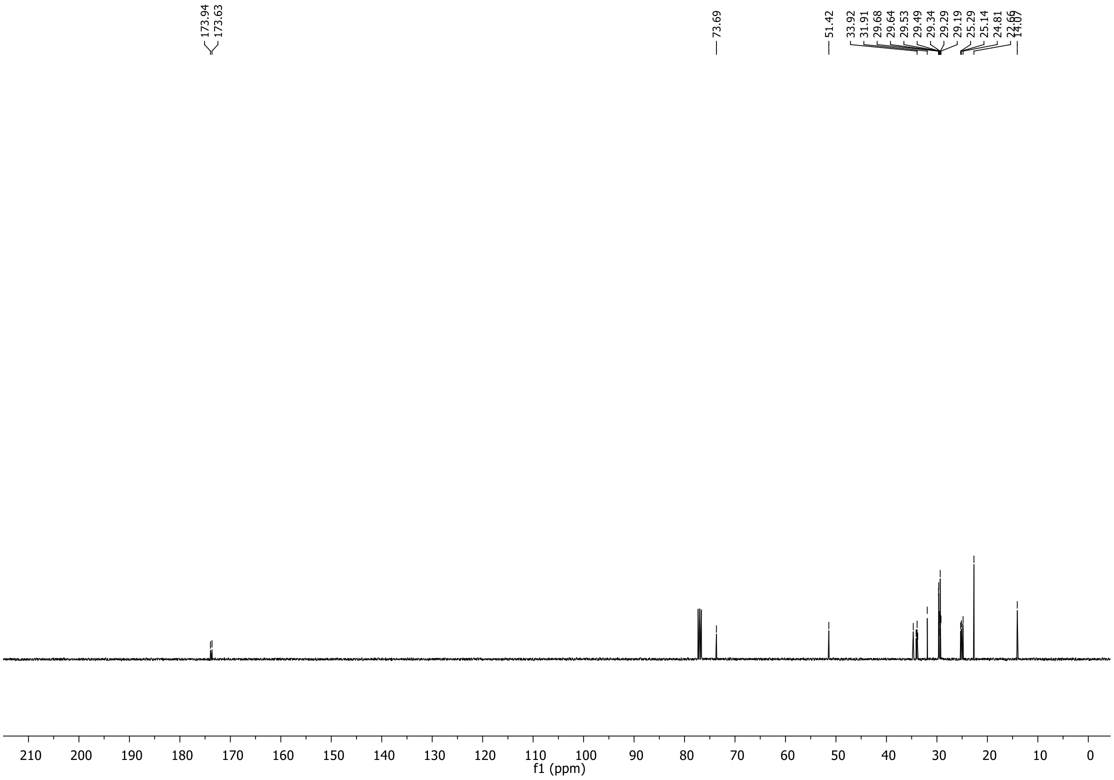
173.94
173.63
73.69
51.42
33.92
31.91
29.68
29.64
29.53
29.49
29.34
29.29
29.19
25.29
25.14
24.81
210 200 190 180 170 160 150 140 130 120 110 100 90 80 70 60 50 40 30 20 10 0
f1 (ppm)

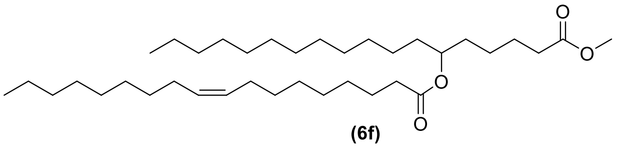
O
O
O
O

(6f)

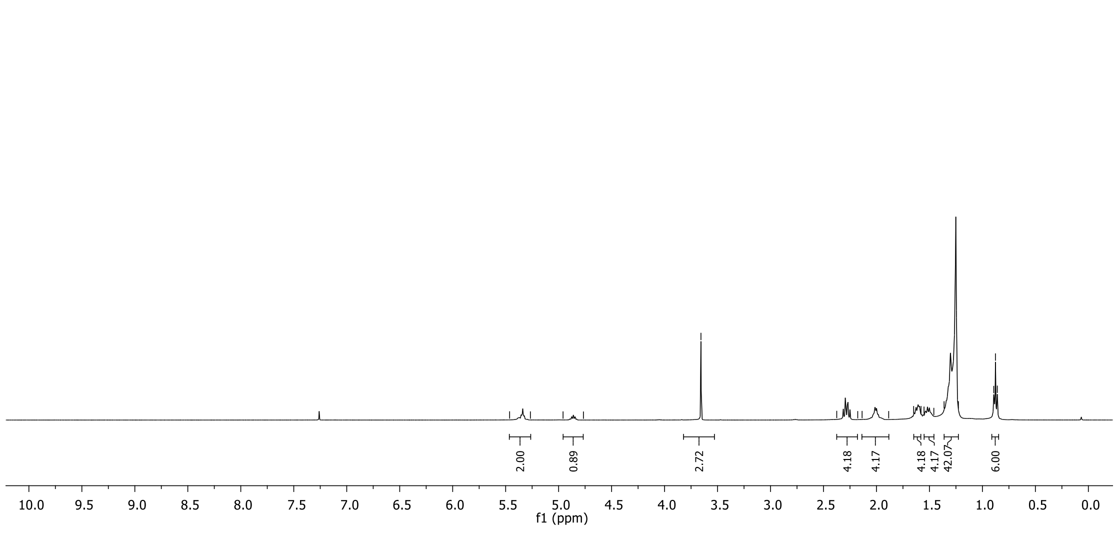
5.46
5.27
4.96
4.77
3.66
2.37
2.18
2.14
1.88
1.65
1.58
1.55
1.46
1.36
1.23
0.89
0.88
0.86
2.00
0.89
2.72
4.18
4.17
4.18
4.17
42.07
6.00
f1 (ppm)

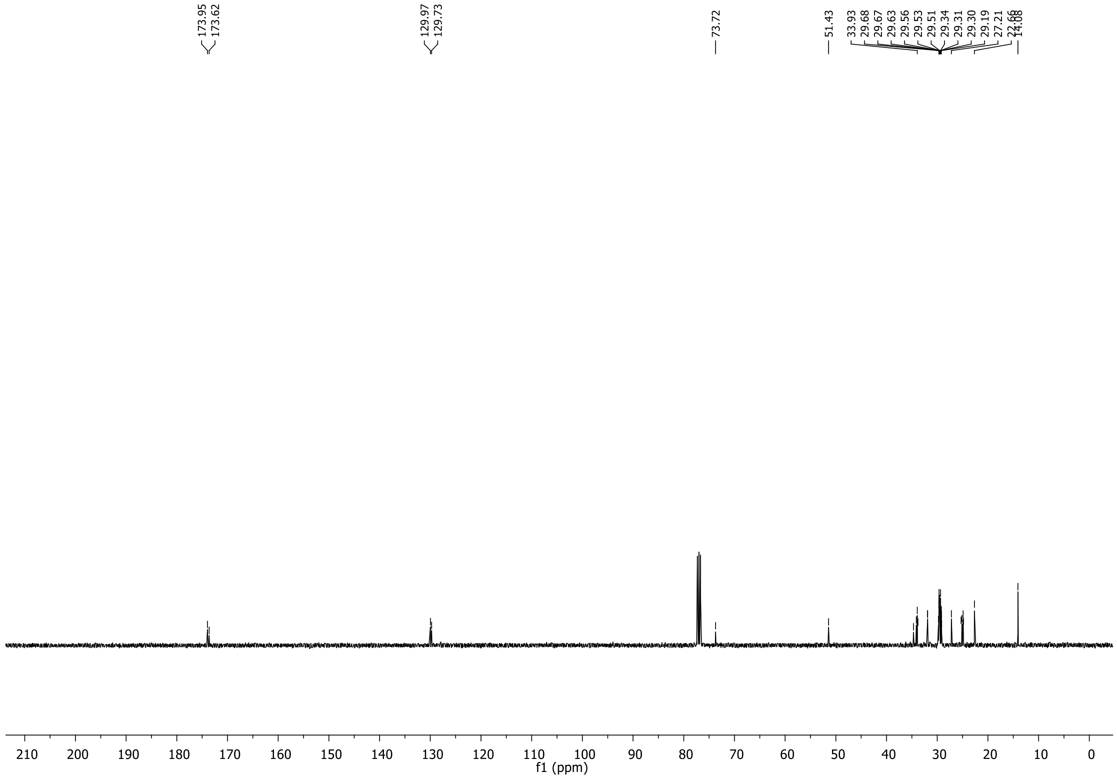
173.95
173.62
129.97
129.73
73.72
51.43
33.93
29.68
29.67
29.63
29.56
29.53
29.51
29.34
29.31
29.30
29.19
27.21
f1 (ppm)

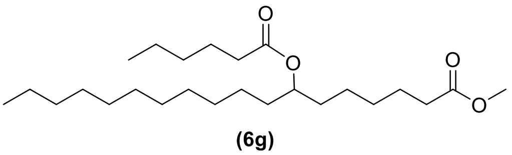

(6g)

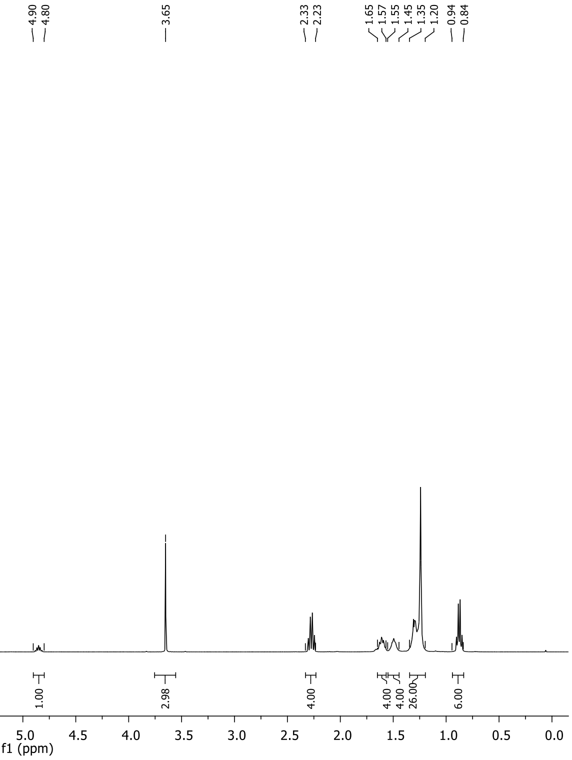

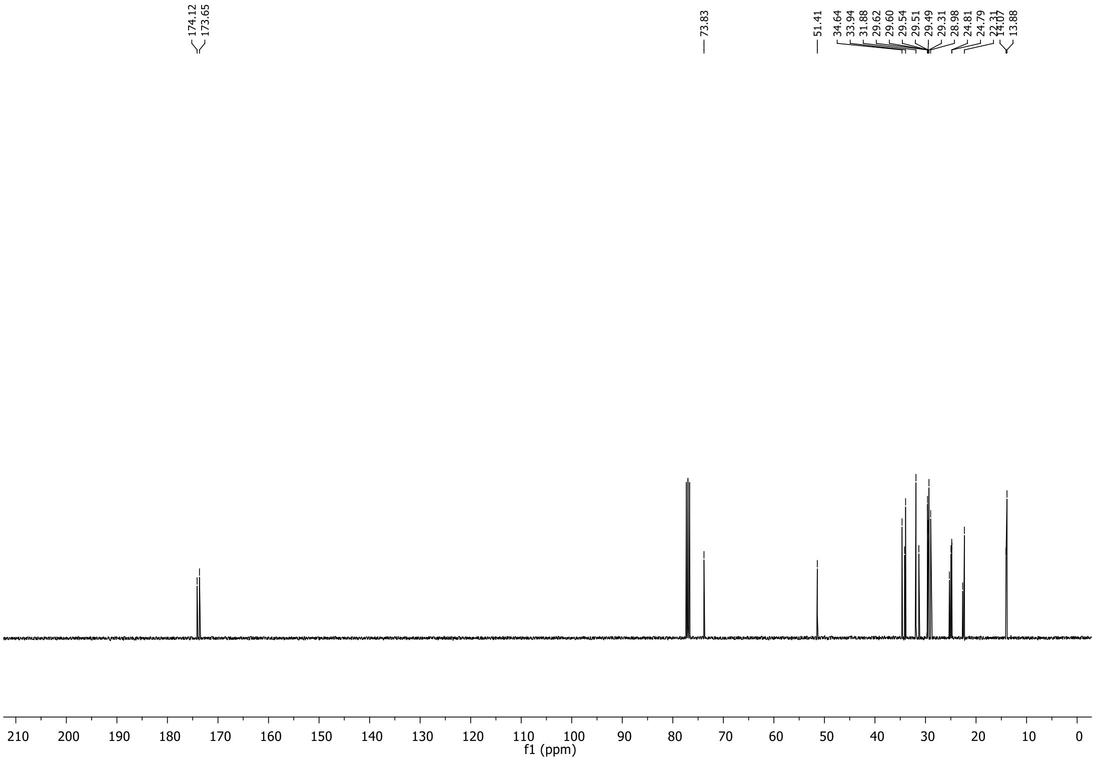

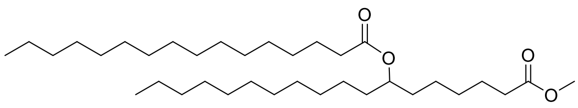

(6h)

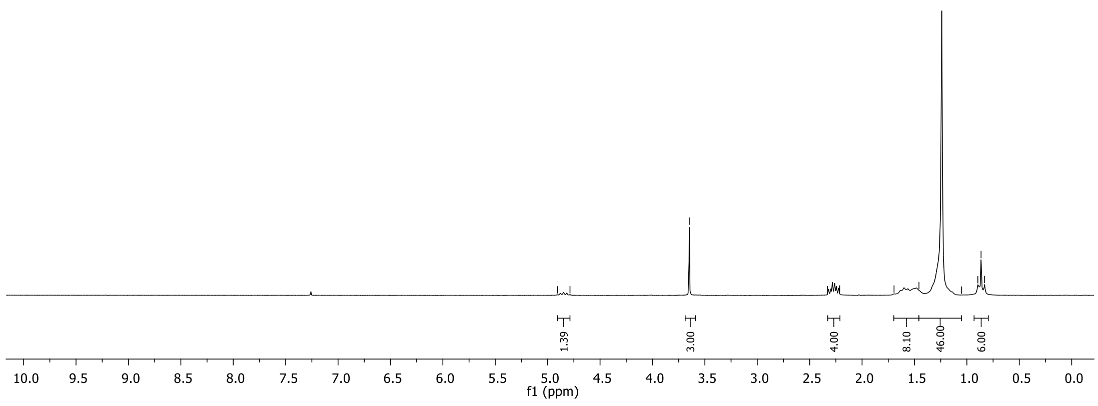
4.91
4.79
3.65
2.33
2.21
1.69
1.46
1.05
0.90
0.86
0.83
1.39
3.00
4.00
8.10
46.00
6.00
f1 (ppm)

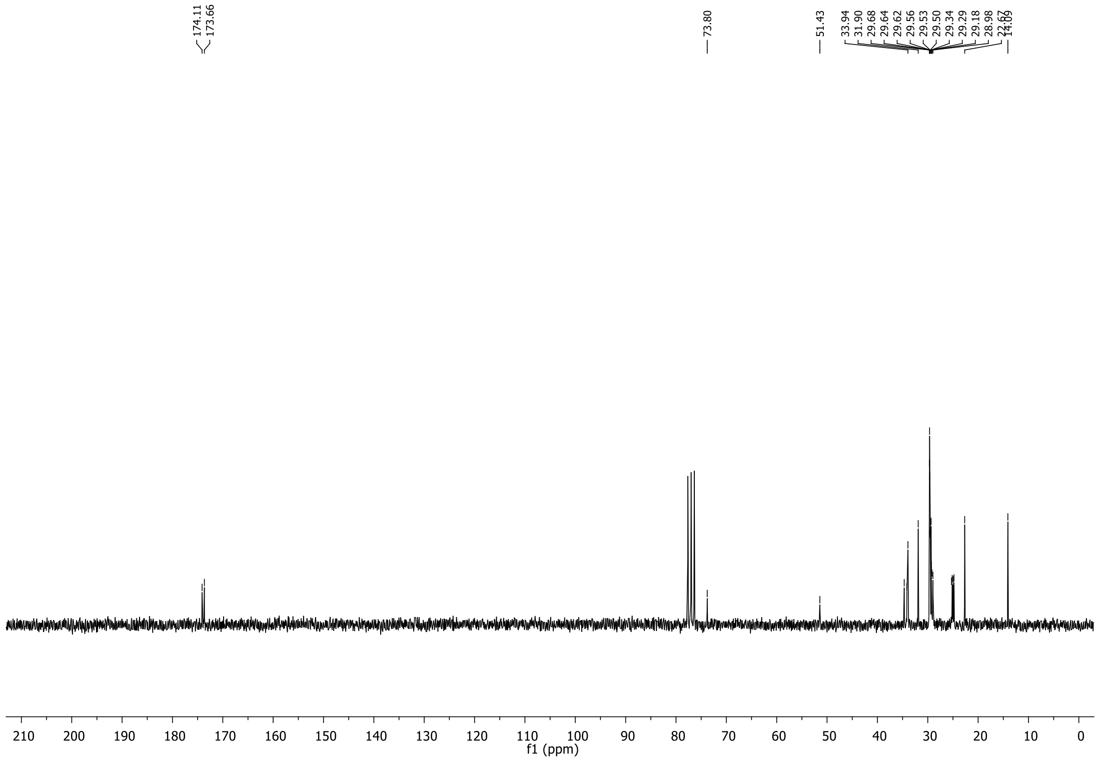
174.11
173.66
73.80
51.43
33.94
31.90
29.68
29.64
29.62
29.56
29.53
29.50
29.34
29.29
29.18
28.98
22.67
14.09
f1 (ppm)

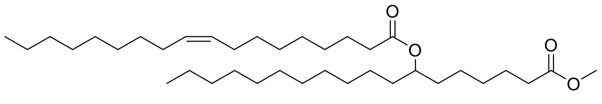
O
O
O
O

(6i)

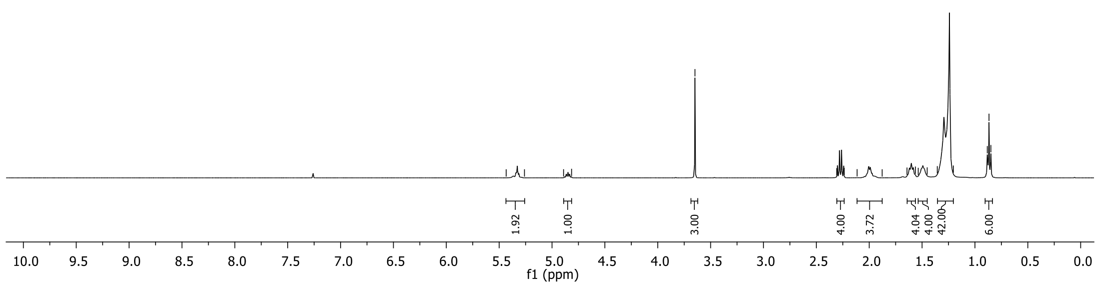
5.43
5.26
4.89
4.81
3.65
2.31
2.24
2.11
1.88
1.64
1.56
1.54
1.45
1.36
1.20
0.88
0.87
0.85
1.92
1.00
3.00
4.00
3.72
4.04
4.00
42.00
6.00
10.0 9.5 9.0 8.5 8.0 7.5 7.0 6.5 6.0 5.5 5.0 4.5 4.0 3.5 3.0 2.5 2.0 1.5 1.0 0.5 0.0
f1 (ppm)

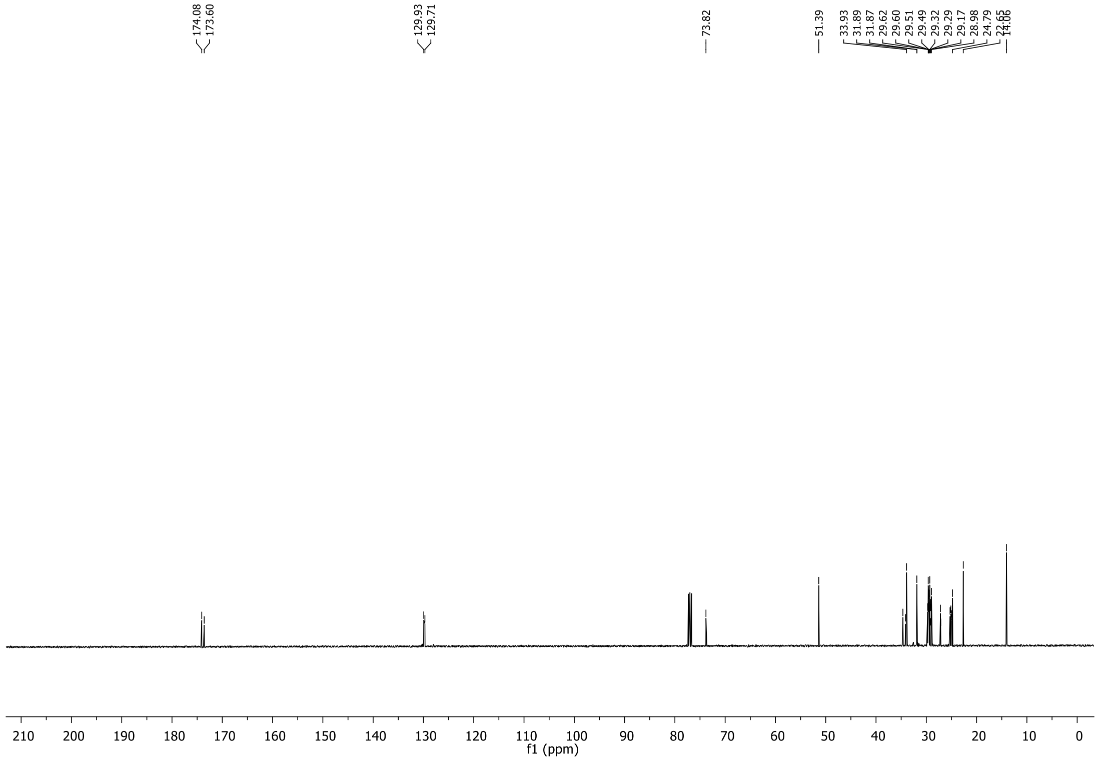
174.08
173.60
129.93
129.71
73.82
51.39
33.93
31.89
31.87
29.62
29.60
29.51
29.49
29.32
29.29
29.17
28.98
24.79
22.65
14.06
210 200 190 180 170 160 150 140 130 120 110 100 90 80 70 60 50 40 30 20 10 0
f1 (ppm)

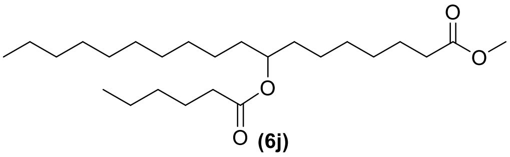

(6j)

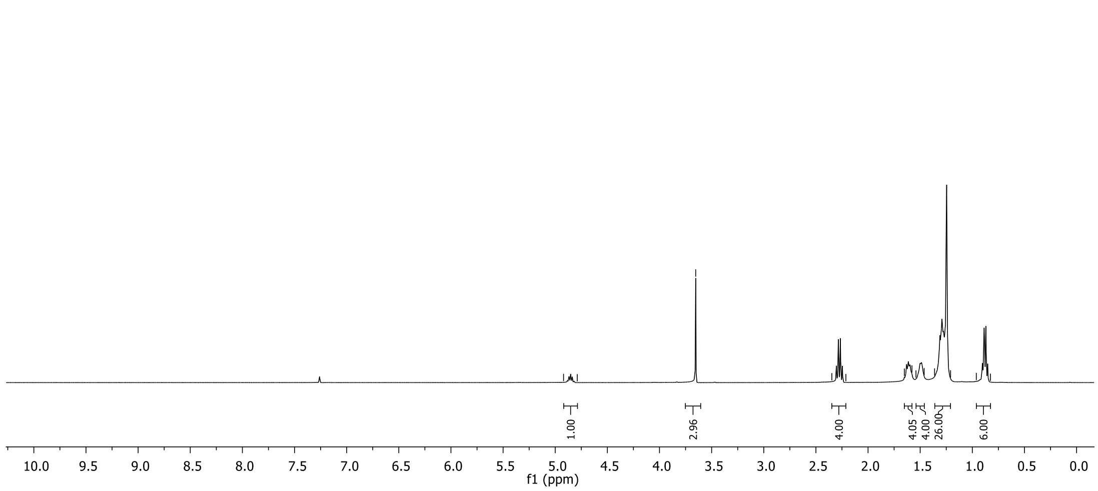
4.92
4.79
3.65
2.35
2.21
1.65
1.58
1.54
1.46
1.36
1.21
0.96
0.83
1.00
2.96
4.00
4.05
4.00
26.00
6.00
f1 (ppm)

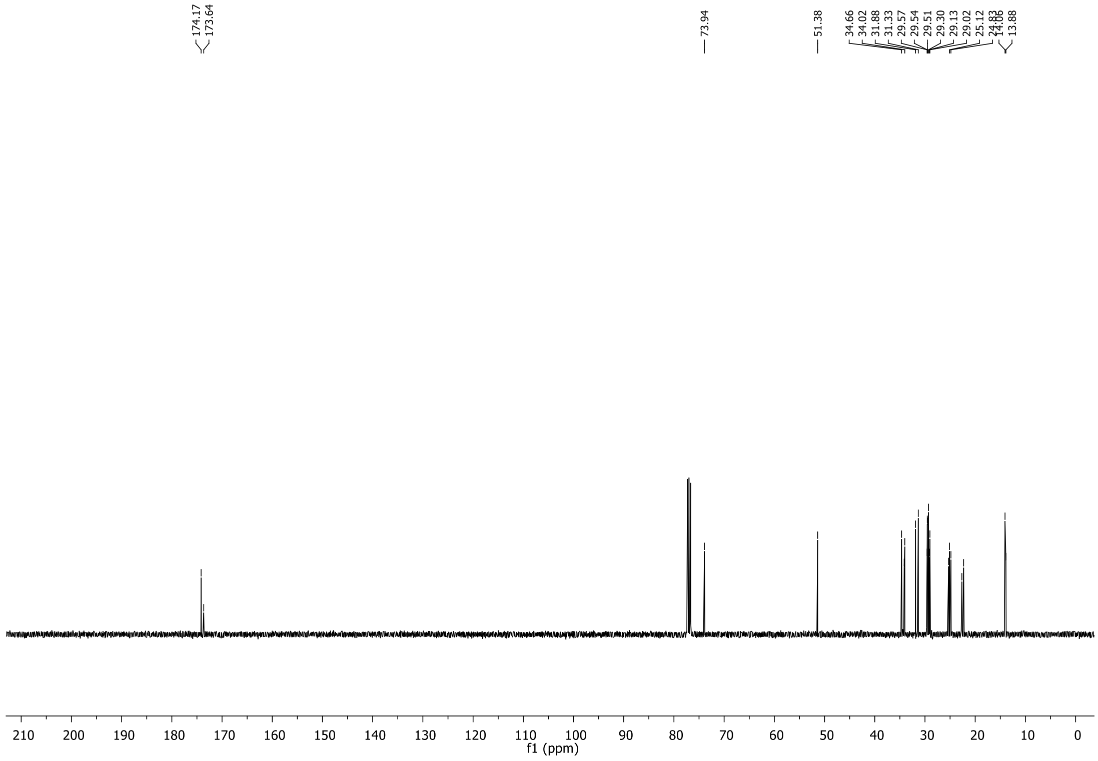
174.17
173.64
73.94
51.38
34.66
34.02
31.88
31.33
29.57
29.54
29.51
29.30
29.13
29.02
25.12
13.88
f1 (ppm)

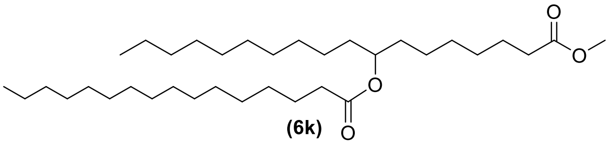

(6k)

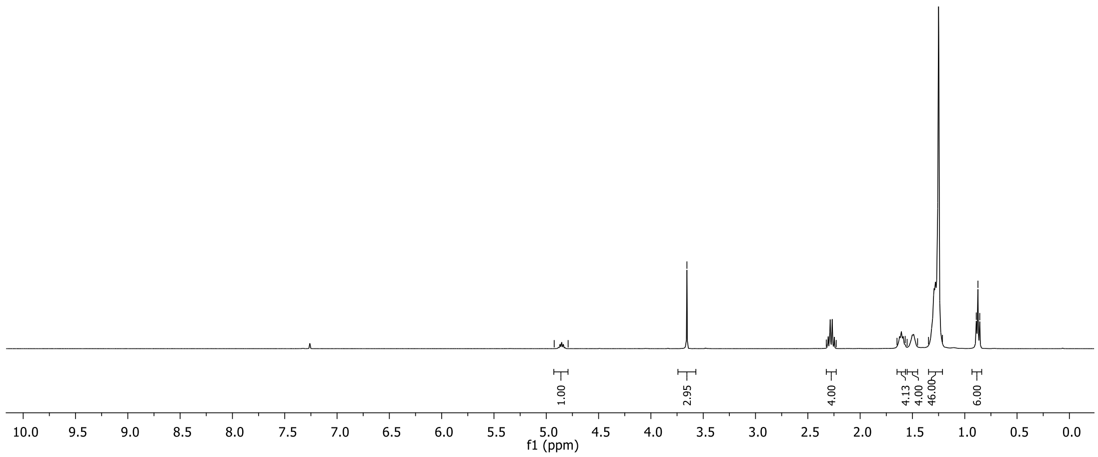

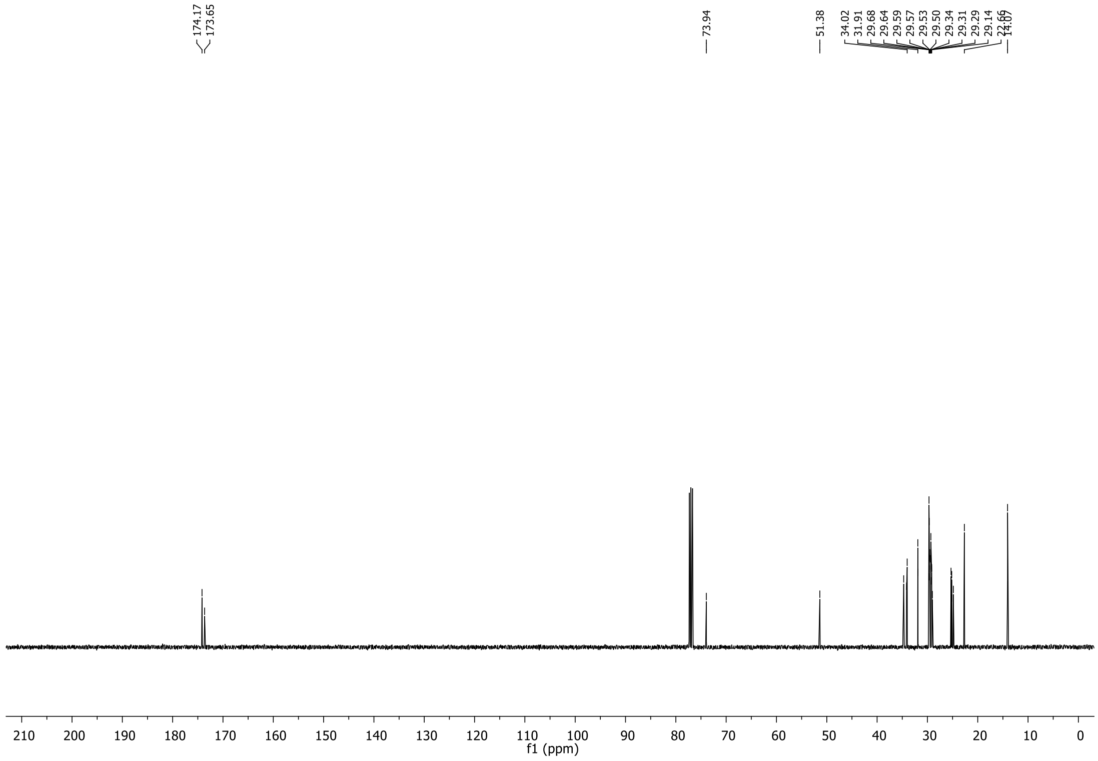

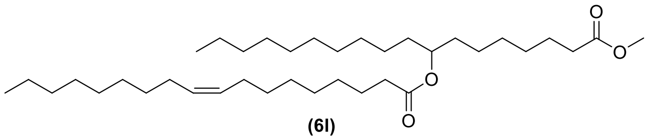
O
O
O
O

(6l)

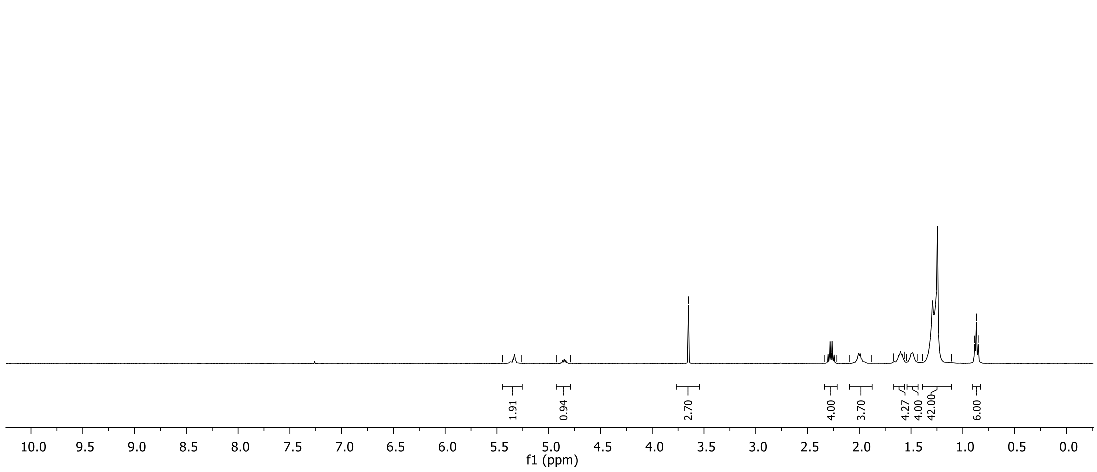
5.45
5.26
4.93
4.79
3.65
2.34
2.22
2.10
1.88
1.67
1.57
1.54
1.43
1.39
1.11
0.89
0.87
0.85
1.91
0.94
2.70
4.00
3.70
4.27
4.00
42.00
6.00
10.0 9.5 9.0 8.5 8.0 7.5 7.0 6.5 6.0 5.5 5.0 4.5 4.0 3.5 3.0 2.5 2.0 1.5 1.0 0.5 0.0
f1 (ppm)

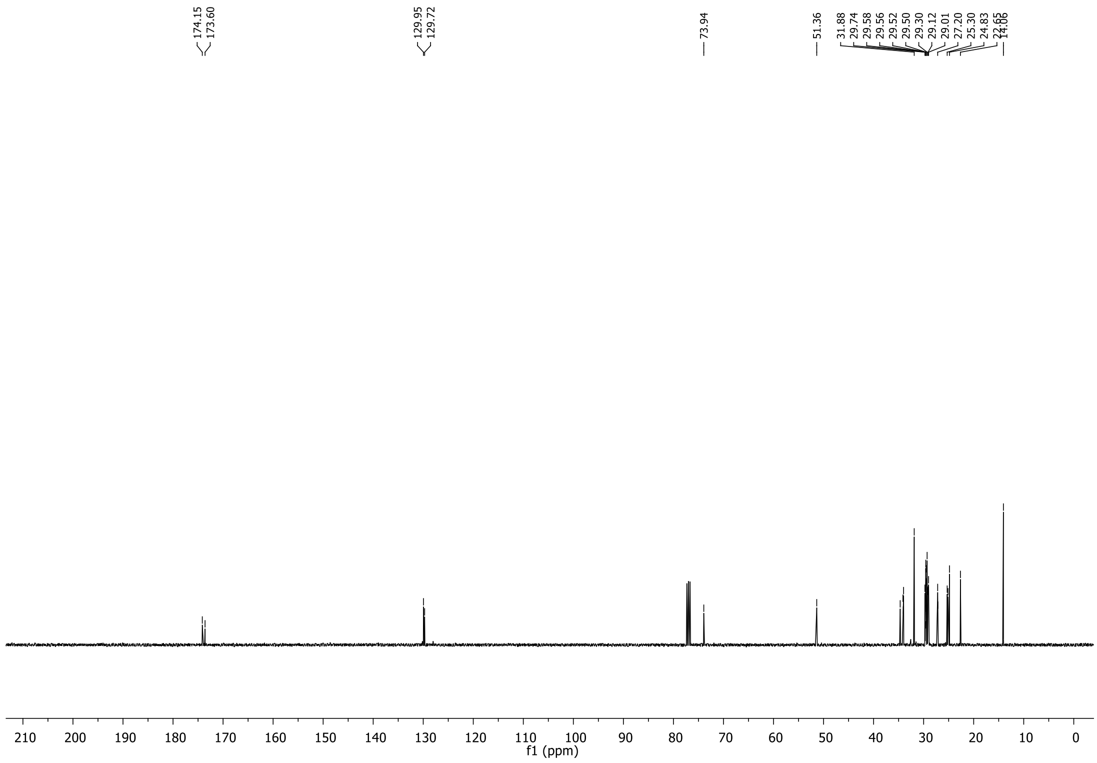
174.15
173.60
129.95
129.72
73.94
51.36
31.88
29.74
29.58
29.56
29.52
29.50
29.30
29.12
29.01
27.20
25.30
24.83
22.65
14.06
210 200 190 180 170 160 150 140 130 120 110 100 90 80 70 60 50 40 30 20 10 0
f1 (ppm)

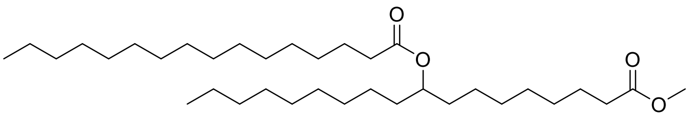

(6m)

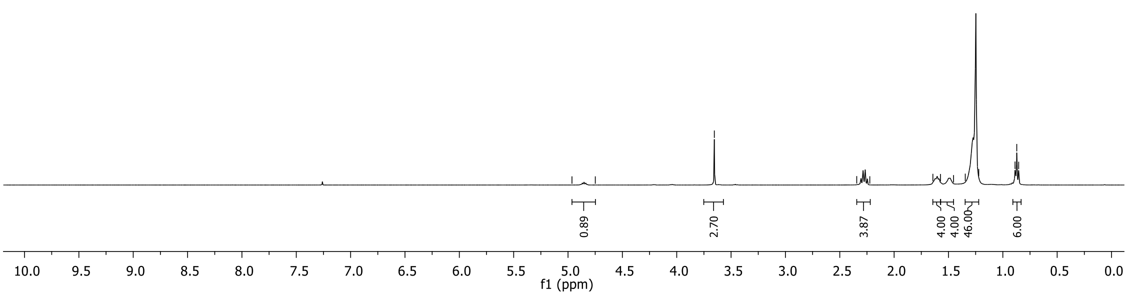
4.96
4.75
3.65
2.34
2.22
1.64
1.57
1.45
1.35
1.22
0.89
0.87
0.85
0.89
2.70
3.87
4.00
4.00
46.00
6.00
10.0 9.5 9.0 8.5 8.0 7.5 7.0 6.5 6.0 5.5 5.0 4.5 4.0 3.5 3.0 2.5 2.0 1.5 1.0 0.5 0.0
f1 (ppm)

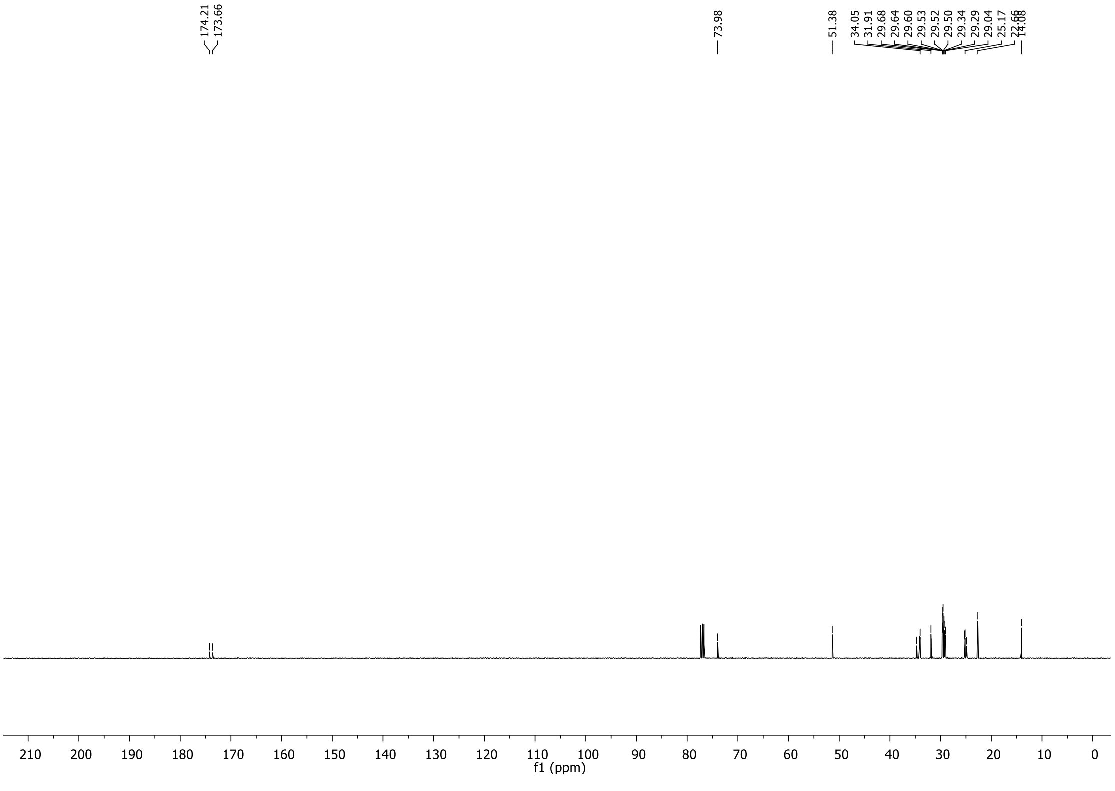
174.21
173.66
73.98
51.38
34.05
31.91
29.68
29.64
29.60
29.53
29.52
29.50
29.34
29.29
29.04
25.17
210 200 190 180 170 160 150 140 130 120 110 100 90 80 70 60 50 40 30 20 10 0
f1 (ppm)

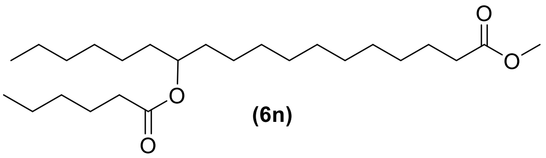

(6n)

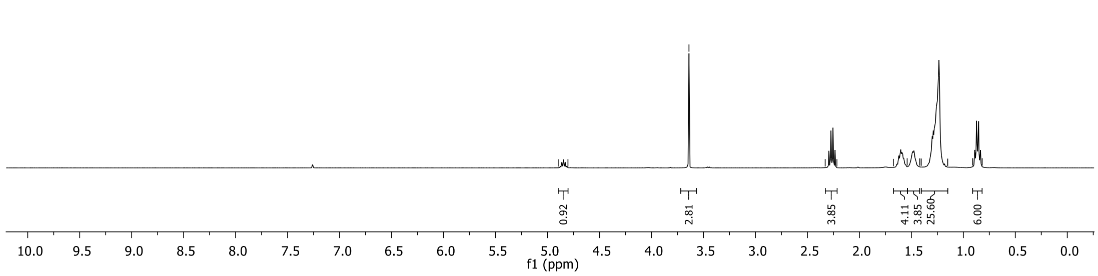
4.90
4.80
3.64
2.33
2.22
1.67
1.54
1.42
1.40
1.15
0.91
0.82
0.92
2.81
3.85
4.11
3.85
25.60
6.00
f1 (ppm)

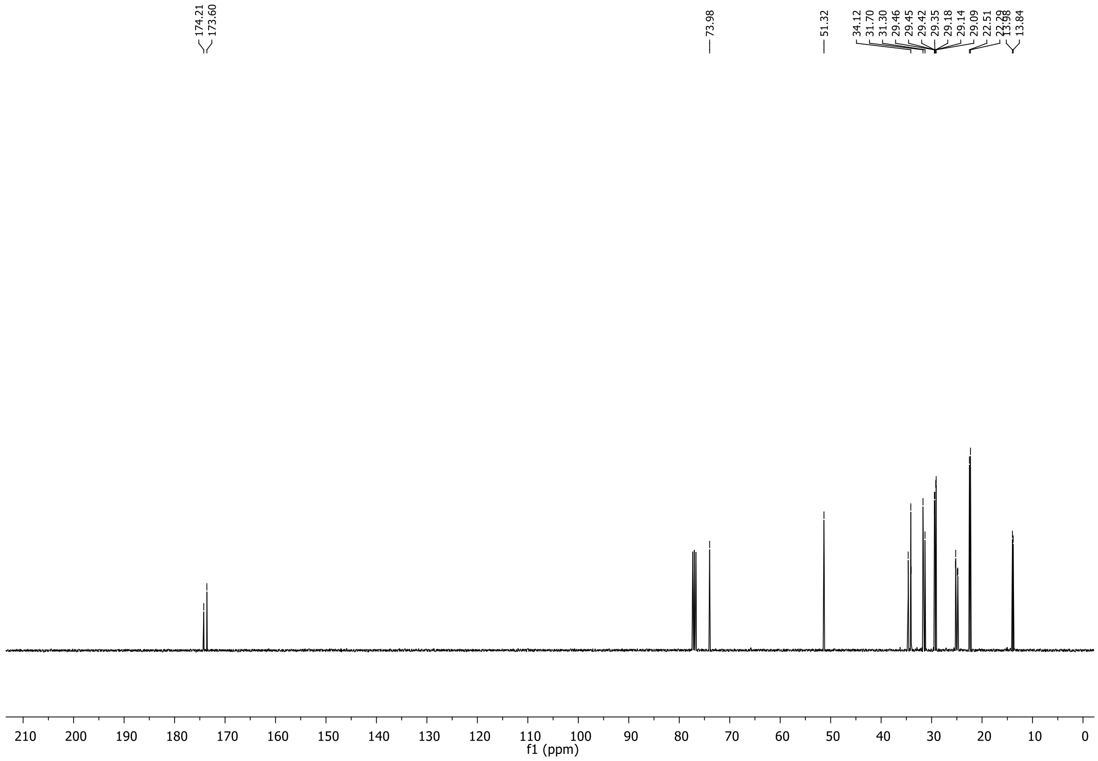
174.21
173.60
73.98
51.32
34.12
31.70
31.30
29.46
29.45
29.42
29.35
29.18
29.14
29.09
22.51
13.84
f1 (ppm)

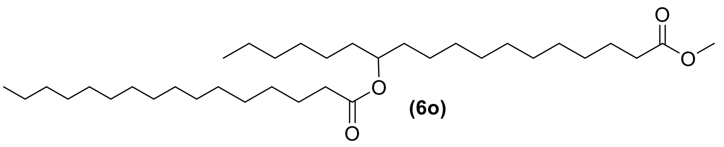

(6o)

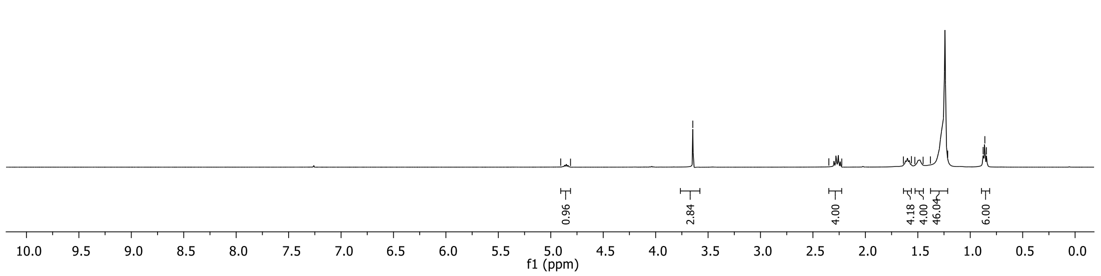
4.91
4.81
3.65
2.35
2.23
1.64
1.56
1.53
1.45
1.38
1.21
0.88
0.86
0.85
0.96
2.84
4.00
4.18
4.00
46.04
6.00
f1 (ppm)

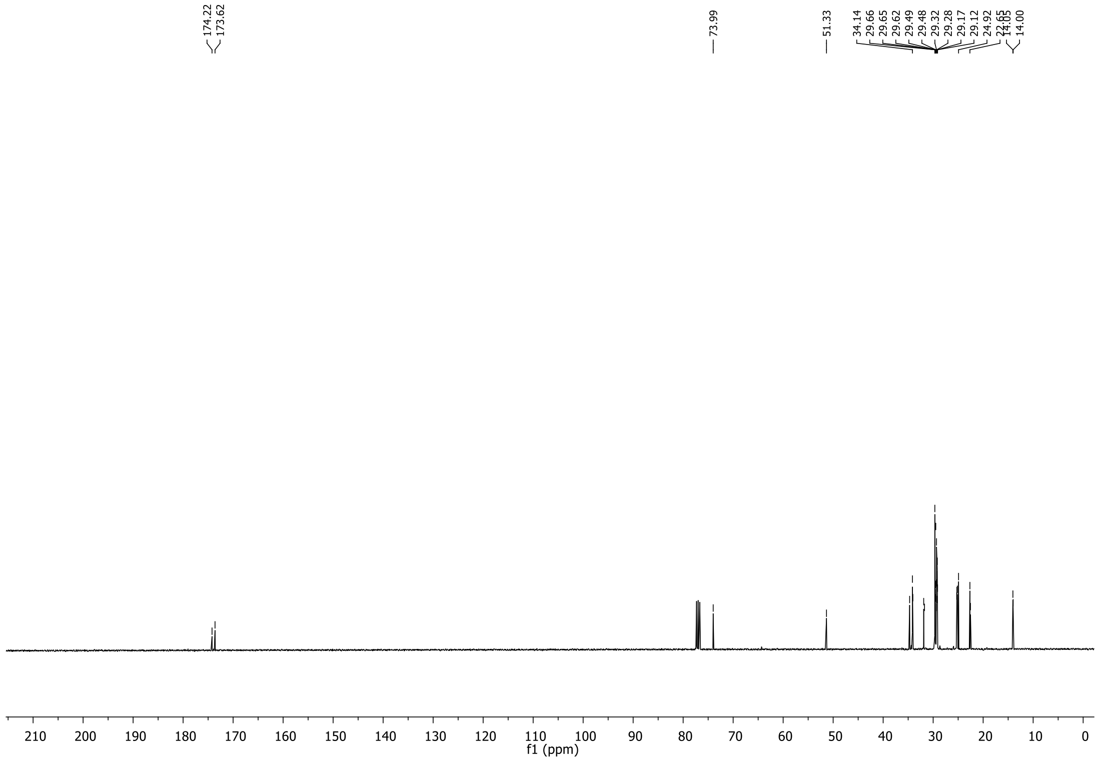
174.22
173.62
73.99
51.33
34.14
29.66
29.65
29.62
29.49
29.48
29.32
29.28
29.17
29.12
24.92
22.65
14.05
14.00
f1 (ppm)

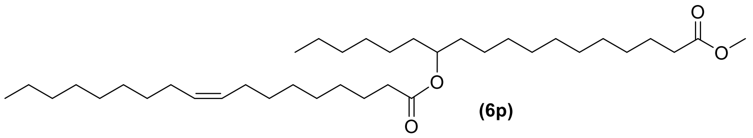

(6p)

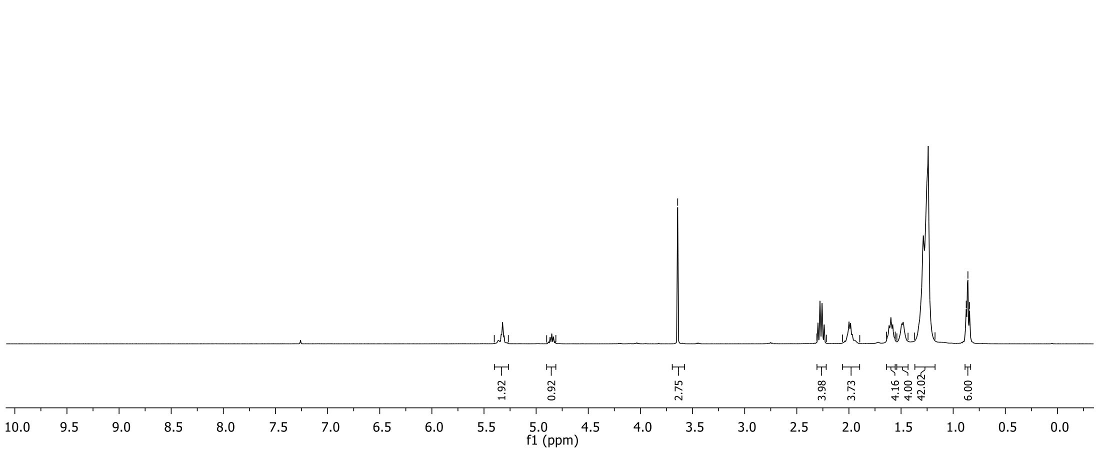
5.40
5.27
4.90
4.81
3.64
2.31
2.22
2.06
1.90
1.64
1.56
1.54
1.43
1.37
1.17
0.87
0.86
0.84
1.92
0.92
2.75
3.98
3.73
4.16
4.00
42.02
6.00
f1 (ppm)

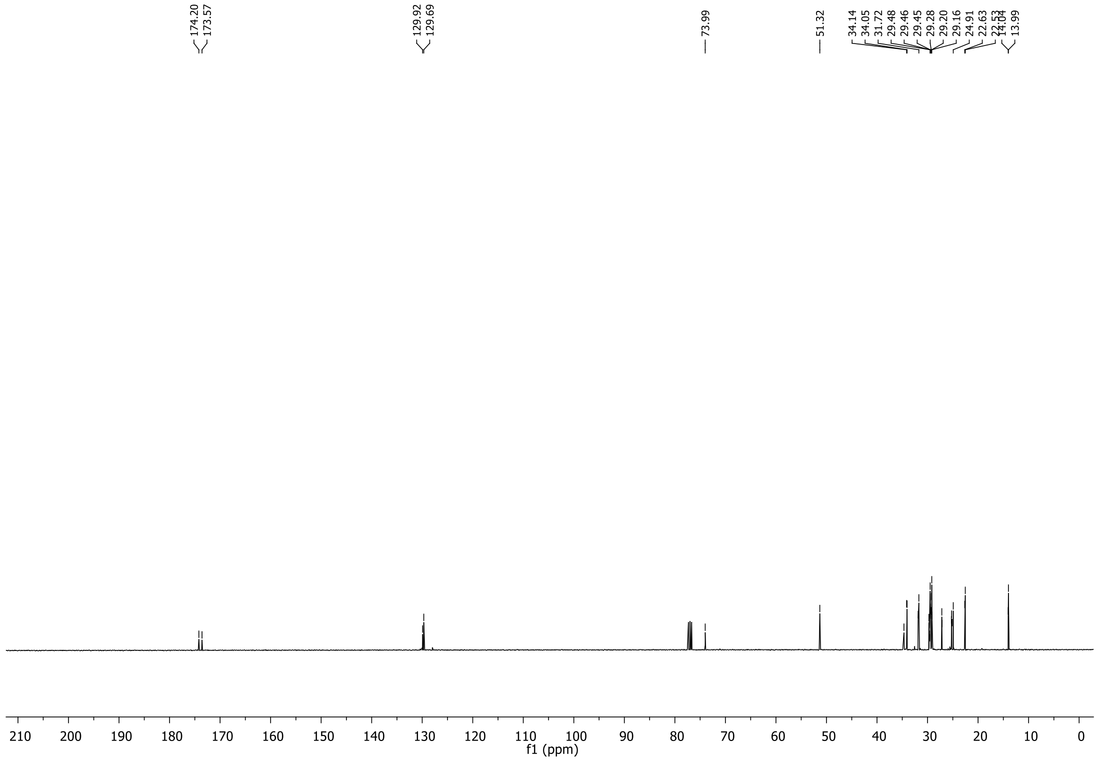
174.20
173.57
129.92
129.69
73.99
51.32
34.14
34.05
31.72
29.48
29.46
29.45
29.28
29.20
29.16
24.91
22.63
13.99
f1 (ppm)

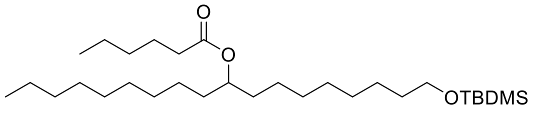
O
O
OTBDMS

(15a)

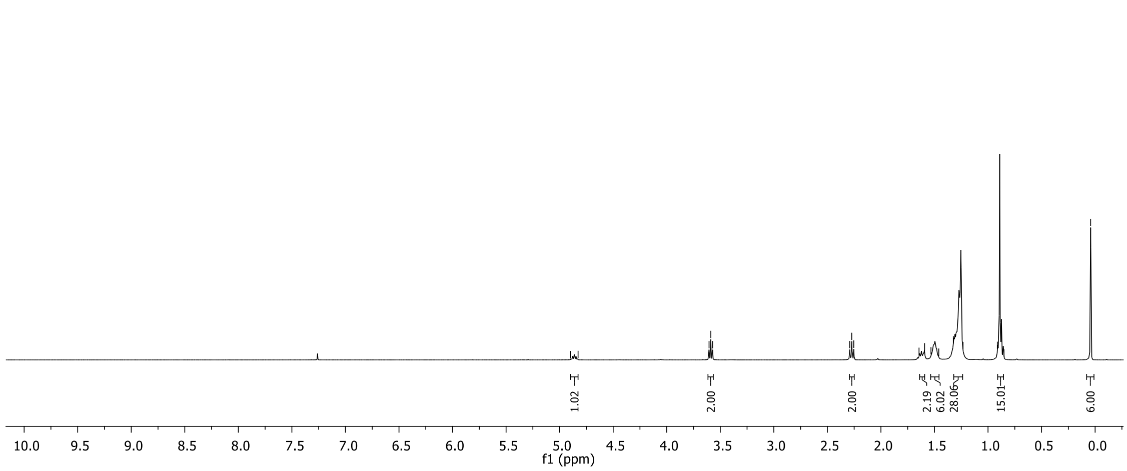
4.90
4.83
3.60
3.59
3.57
2.29
2.27
2.25
1.64
1.59
1.53
1.46
1.32
1.23
0.91
0.85
0.04
1.02
2.00
2.00
2.19
6.02
28.06
15.01
6.00
f1 (ppm)

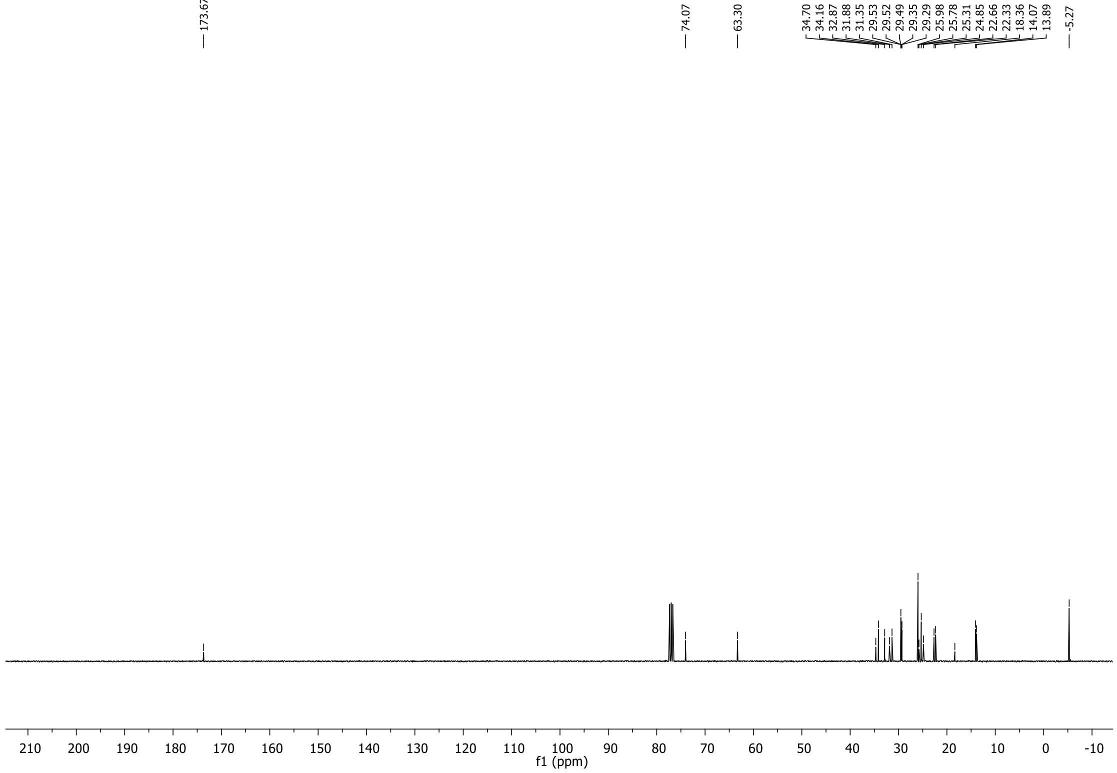
173.67
74.07
63.30
34.70
34.16
32.87
31.88
31.35
29.53
29.52
29.49
29.35
29.29
25.98
25.78
25.31
24.85
22.66
22.33
18.36
14.07
13.89
-5.27
f1 (ppm)

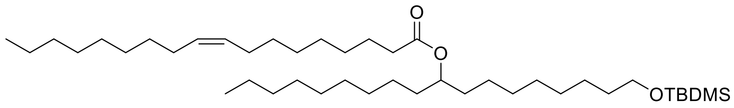
OTBDMS

(15b)

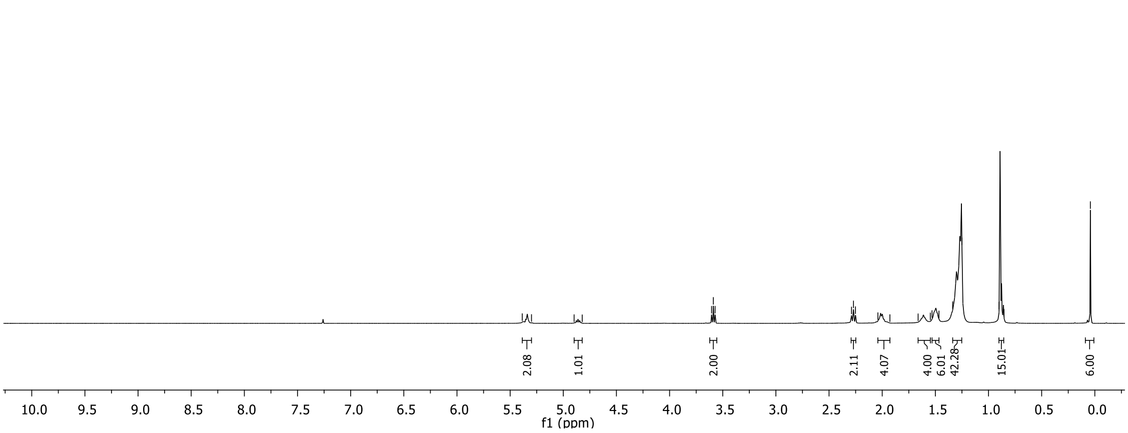
5.39
5.30
4.90
4.82
3.61
3.59
3.57
2.29
2.27
2.25
2.04
1.93
1.66
1.55
1.53
1.47
1.34
1.25
0.90
0.85
0.04
2.08
1.01
2.00
2.11
4.07
4.00
6.01
42.28
15.01
6.00
f1 (ppm)

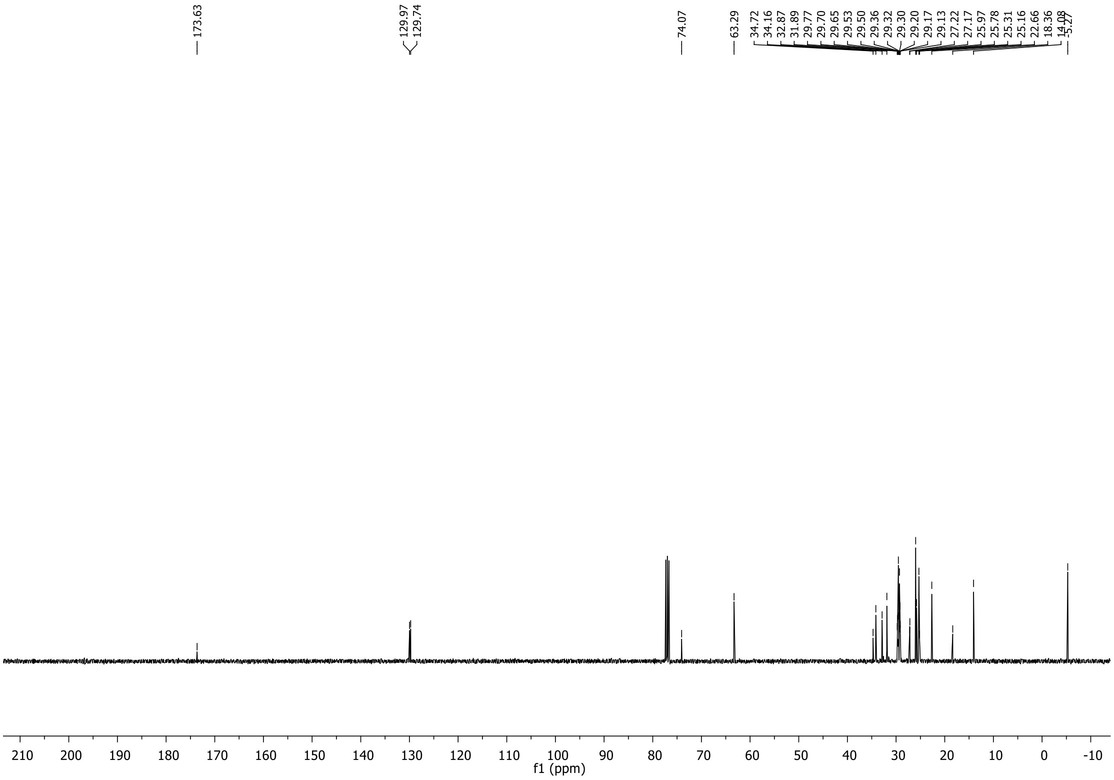
173.63
129.97
129.74
74.07
63.29
34.72
34.16
32.87
31.89
29.77
29.70
29.65
29.53
29.50
29.36
29.32
29.30
29.20
29.17
29.13
27.22
27.17
25.97
25.78
25.31
25.16
22.66
18.36
14.08
-5.27
f1 (ppm)

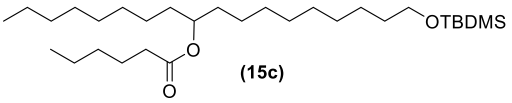
OTBDMS
O
O

(15c)

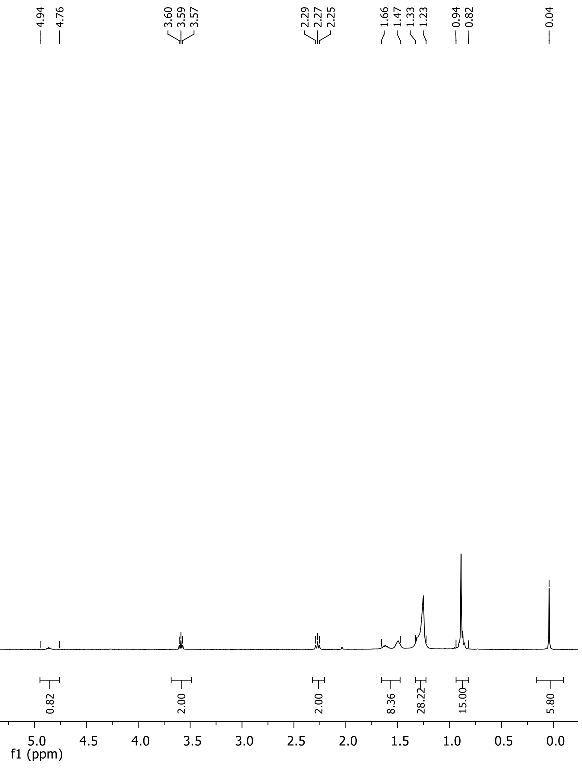
4.94
4.76
3.60
3.59
3.57
2.29
2.27
2.25
1.66
1.47
1.33
1.23
0.94
0.82
0.04
0.82
2.00
2.00
8.36
28.22
15.00
5.80
10.0 9.5 9.0 8.5 8.0 7.5 7.0 6.5 6.0 5.5 5.0 4.5 4.0 3.5 3.0 2.5 2.0 1.5 1.0 0.5 0.0
f1 (ppm)

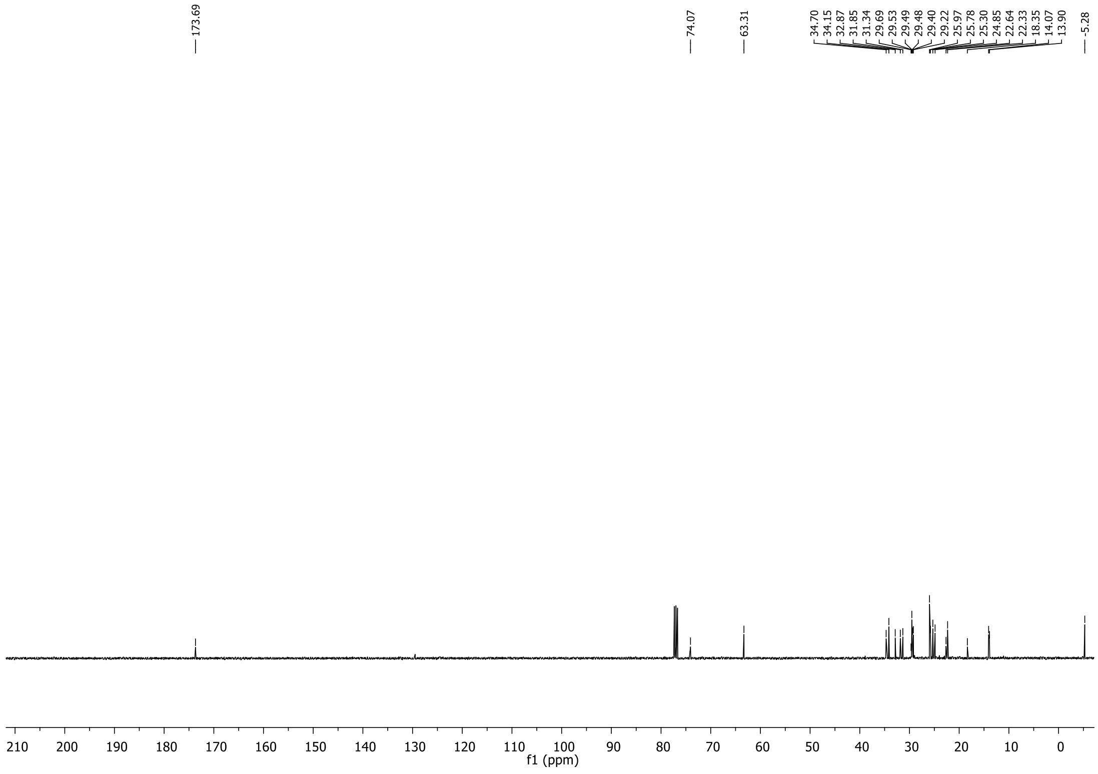
173.69
74.07
63.31
34.70
34.15
32.87
31.85
31.34
29.69
29.53
29.49
29.48
29.40
29.22
25.97
25.78
25.30
24.85
22.64
22.33
18.35
14.07
13.90
-5.28
210 200 190 180 170 160 150 140 130 120 110 100 90 80 70 60 50 40 30 20 10 0
f1 (ppm)

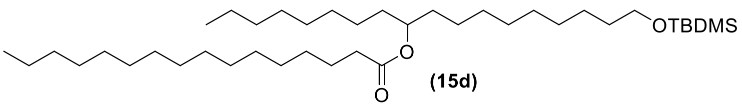
OTBDMS
O
O

(15d)

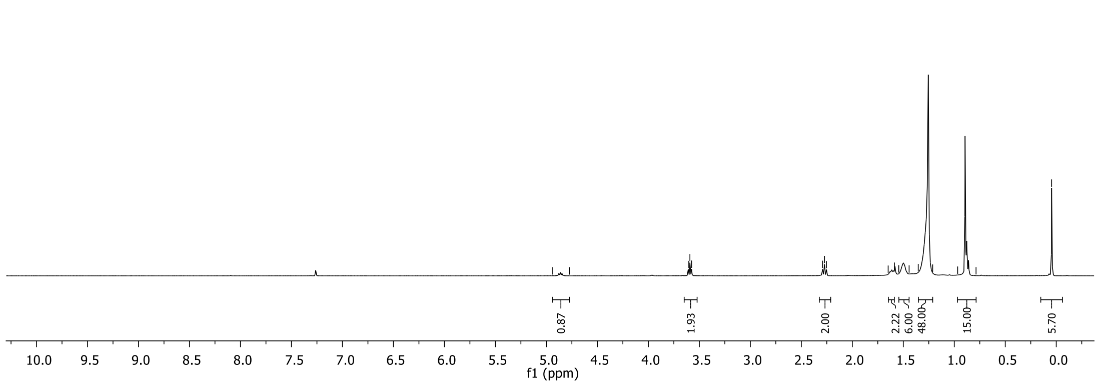
4.94
4.77
3.61
3.59
3.58
2.29
2.27
2.25
1.65
1.59
1.54
1.44
1.35
1.21
0.97
0.79
0.05
0.87
1.93
2.00
2.22
6.00
48.00
15.00
5.70
f1 (ppm)

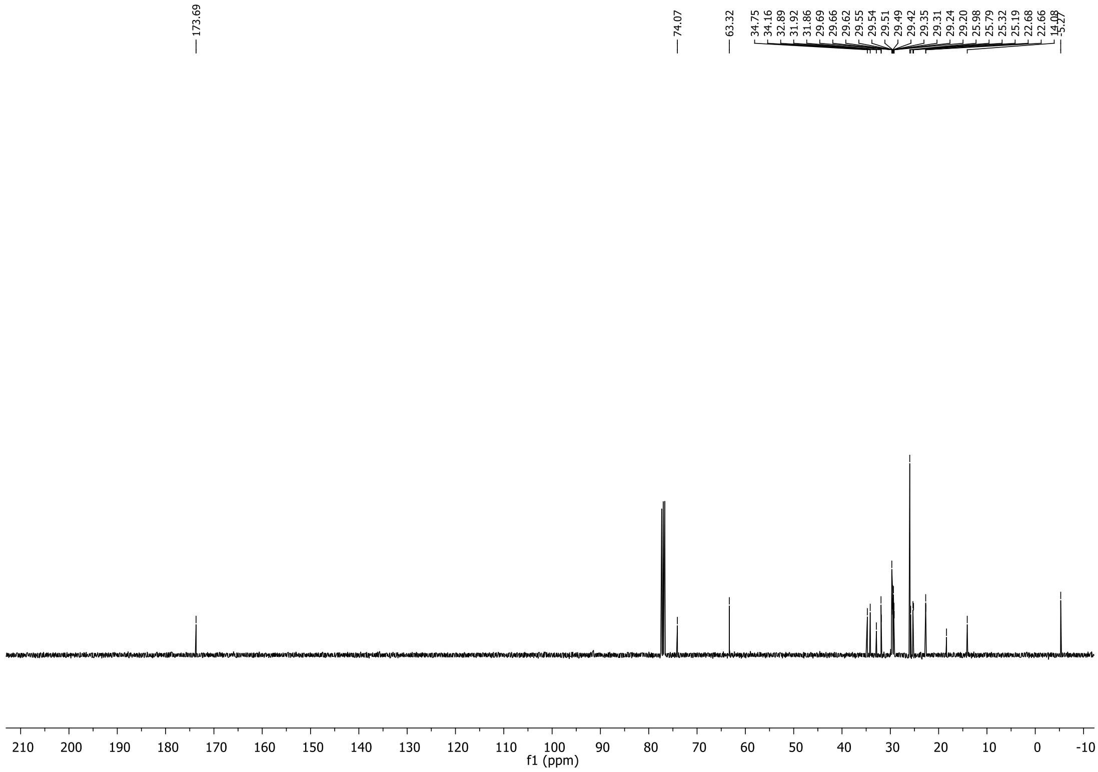
173.69
74.07
63.32
34.75
34.16
32.89
31.92
31.86
29.69
29.66
29.62
29.55
29.54
29.51
29.49
29.42
29.35
29.31
29.24
29.20
25.98
25.79
25.32
25.19
22.68
22.66
14.08
-5.27
f1 (ppm)

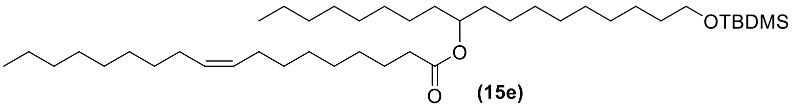
OTBDMS
O
O

(15e)

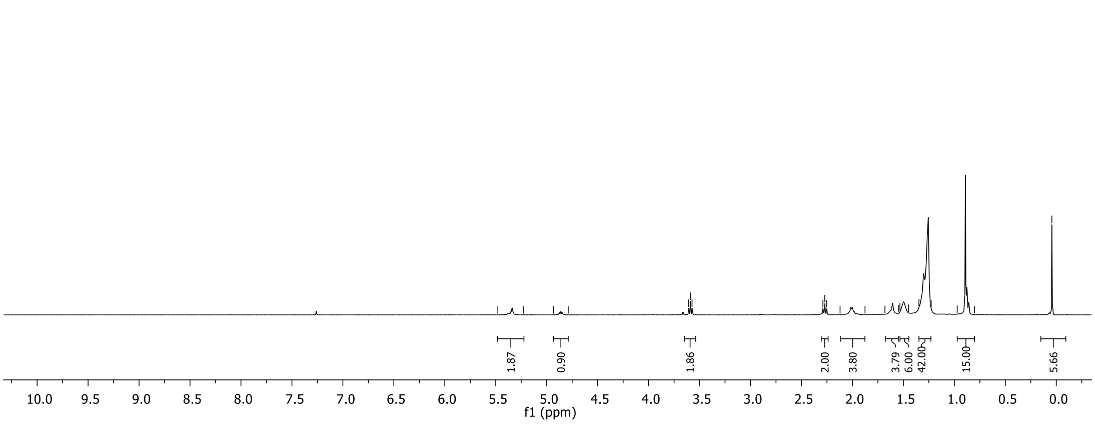
5.49
5.23
4.93
4.79
3.61
3.59
3.57
2.29
2.27
2.25
2.12
1.88
1.68
1.55
1.53
1.45
1.35
1.23
0.97
0.80
0.04
1.87
0.90
1.86
2.00
3.80
3.79
6.00
42.00
15.00
5.66
10.0 9.5 9.0 8.5 8.0 7.5 7.0 6.5 6.0 5.5 5.0 4.5 4.0 3.5 3.0 2.5 2.0 1.5 1.0 0.5 0.0
f1 (ppm)

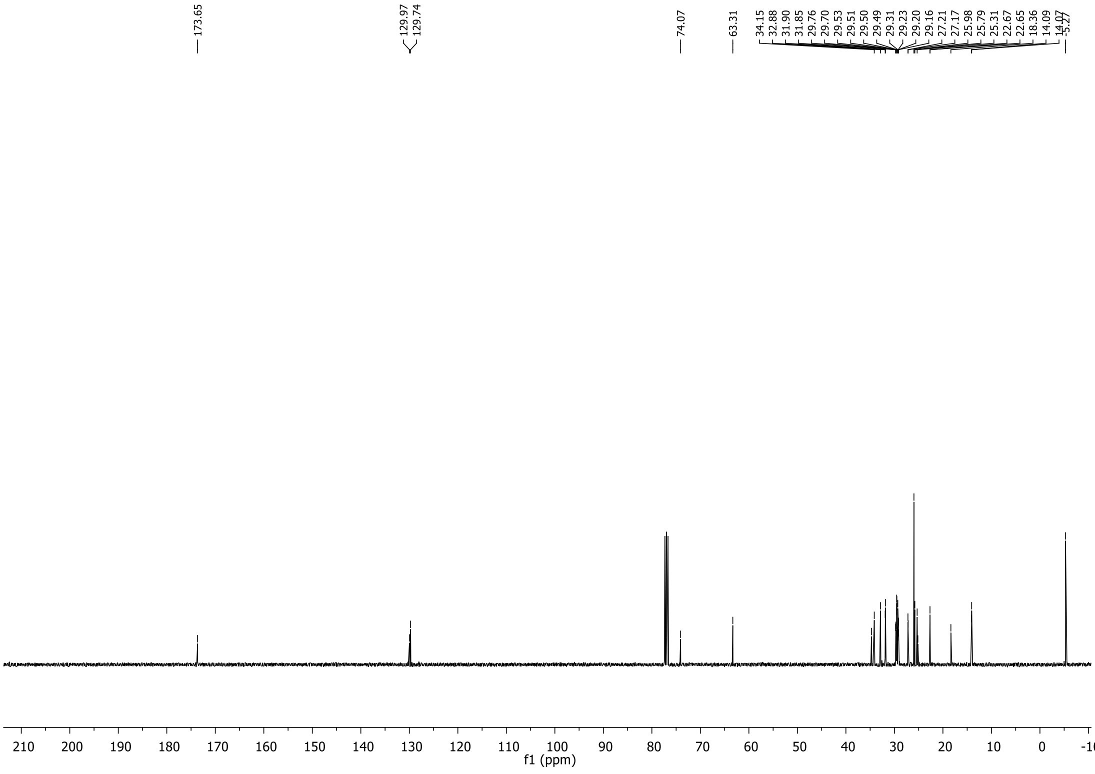
173.65
129.97
129.74
74.07
63.31
34.15
32.88
31.90
31.85
29.76
29.70
29.53
29.51
29.50
29.49
29.31
29.23
29.20
29.16
27.21
27.17
25.98
25.79
25.31
22.67
22.65
18.36
14.09
14.07
-5.27
210 200 190 180 170 160 150 140 130 120 110 100 90 80 70 60 50 40 30 20 10 0 -10
f1 (ppm)

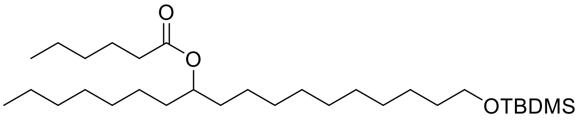
O
O
OTBDMS

(15f)

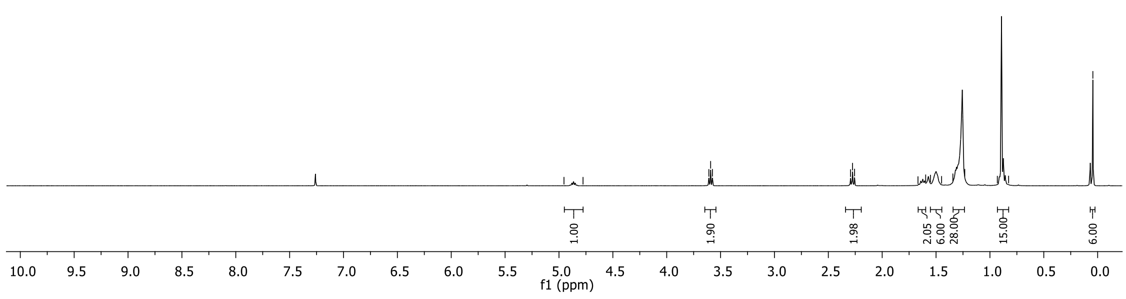
4.95
4.78
3.61
3.59
3.58
2.29
2.28
2.26
1.67
1.60
1.55
1.45
1.34
1.23
0.93
0.83
0.04
1.00
1.90
1.98
2.05
6.00
28.00
15.00
6.00
10.0 9.5 9.0 8.5 8.0 7.5 7.0 6.5 6.0 5.5 5.0 4.5 4.0 3.5 3.0 2.5 2.0 1.5 1.0 0.5 0.0
f1 (ppm)

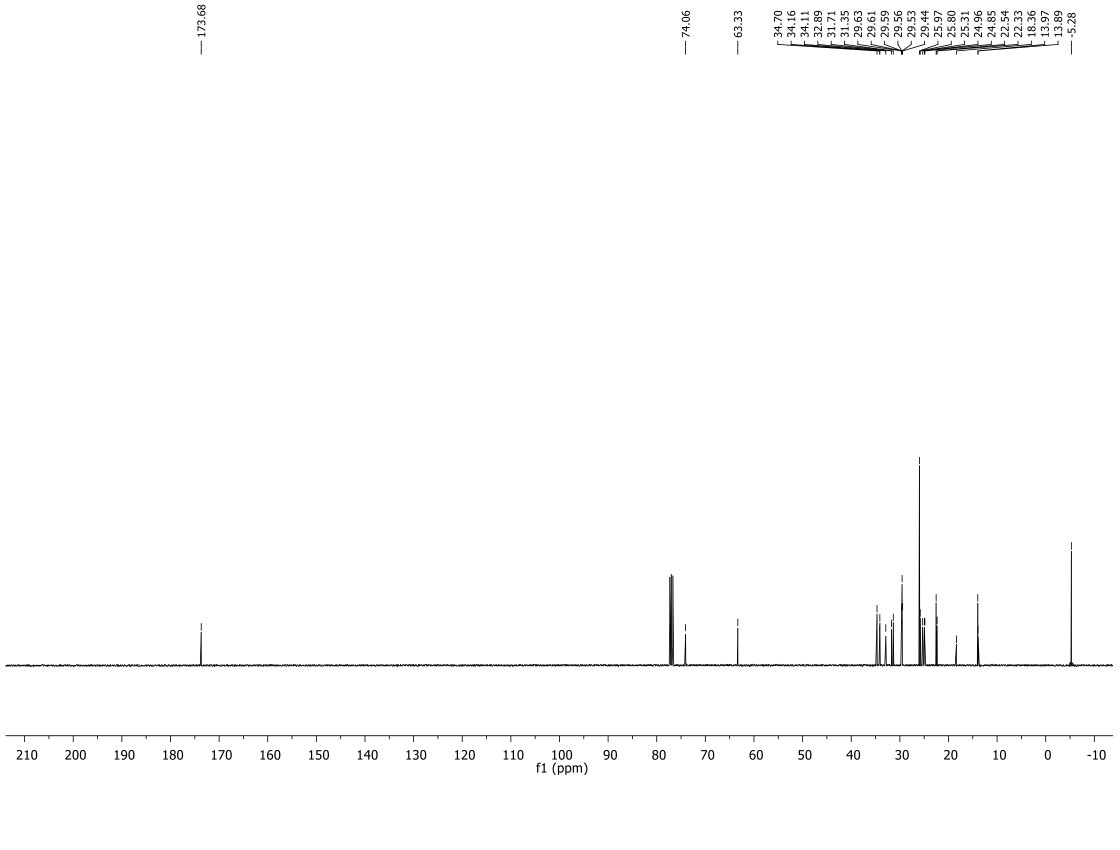
173.68
74.06
63.33
34.70
34.16
34.11
32.89
31.71
31.35
29.63
29.61
29.59
29.56
29.53
29.44
25.97
25.80
25.31
24.96
24.85
22.54
22.33
18.36
13.97
13.89
-5.28
210 200 190 180 170 160 150 140 130 120 110 100 90 80 70 60 50 40 30 20 10 0 -10
f1 (ppm)

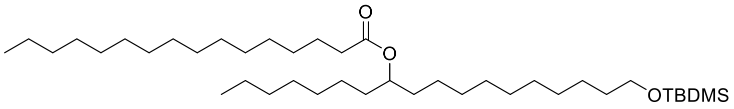
O
O
OTBDMS

(15g)

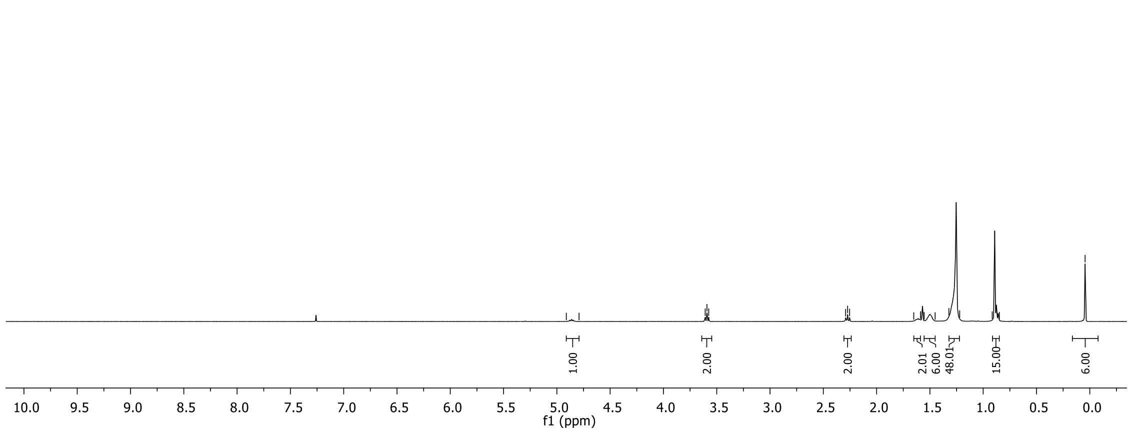
4.91
4.79
3.61
3.59
3.58
2.29
2.27
2.25
1.65
1.59
1.55
1.45
1.32
1.22
0.92
0.85
0.04
1.00
2.00
2.00
2.01
6.00
48.01
15.00
6.00
f1 (ppm)

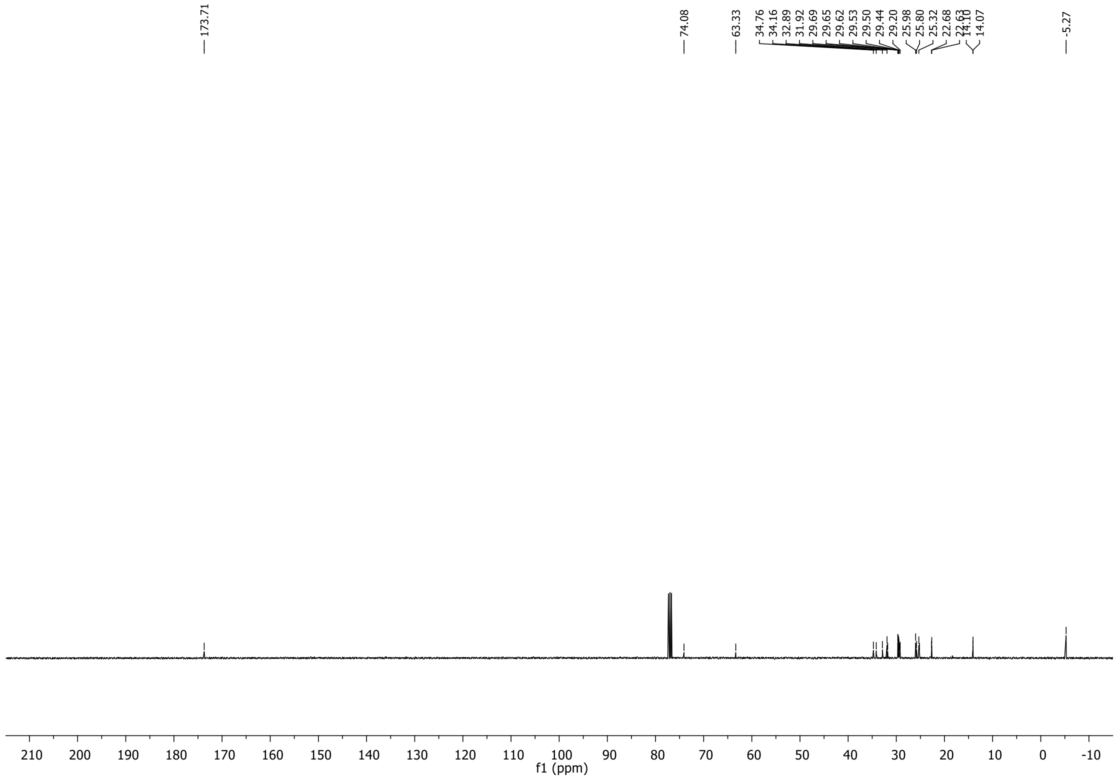
173.71
74.08
63.33
34.76
34.16
32.89
31.92
29.69
29.65
29.62
29.53
29.50
29.44
29.20
25.98
25.80
25.32
22.68
14.07
-5.27
f1 (ppm)

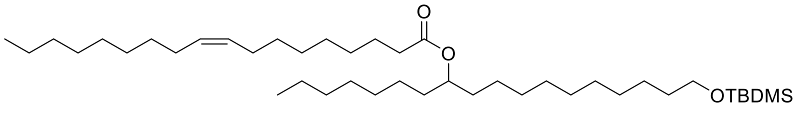
O
O
OTBDMS

(15h)

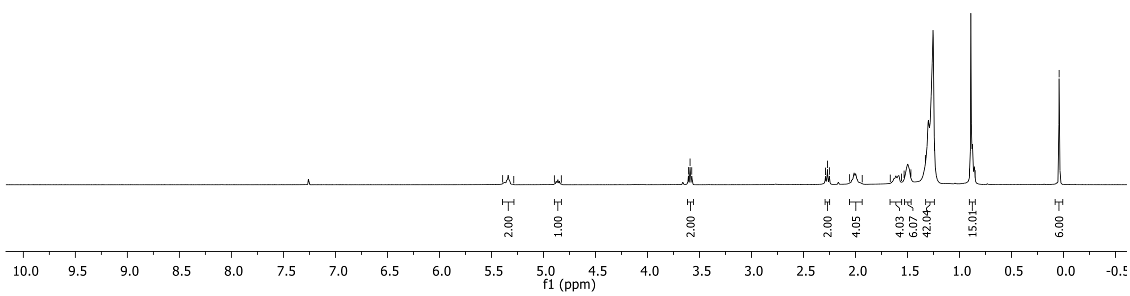
5.39
5.28
4.90
4.83
3.61
3.59
3.57
2.29
2.27
2.25
2.06
1.94
1.67
1.56
1.53
1.47
1.33
1.24
0.90
0.85
0.04
2.00
1.00
2.00
2.00
4.05
4.03
6.07
42.04
15.01
6.00
f1 (ppm)

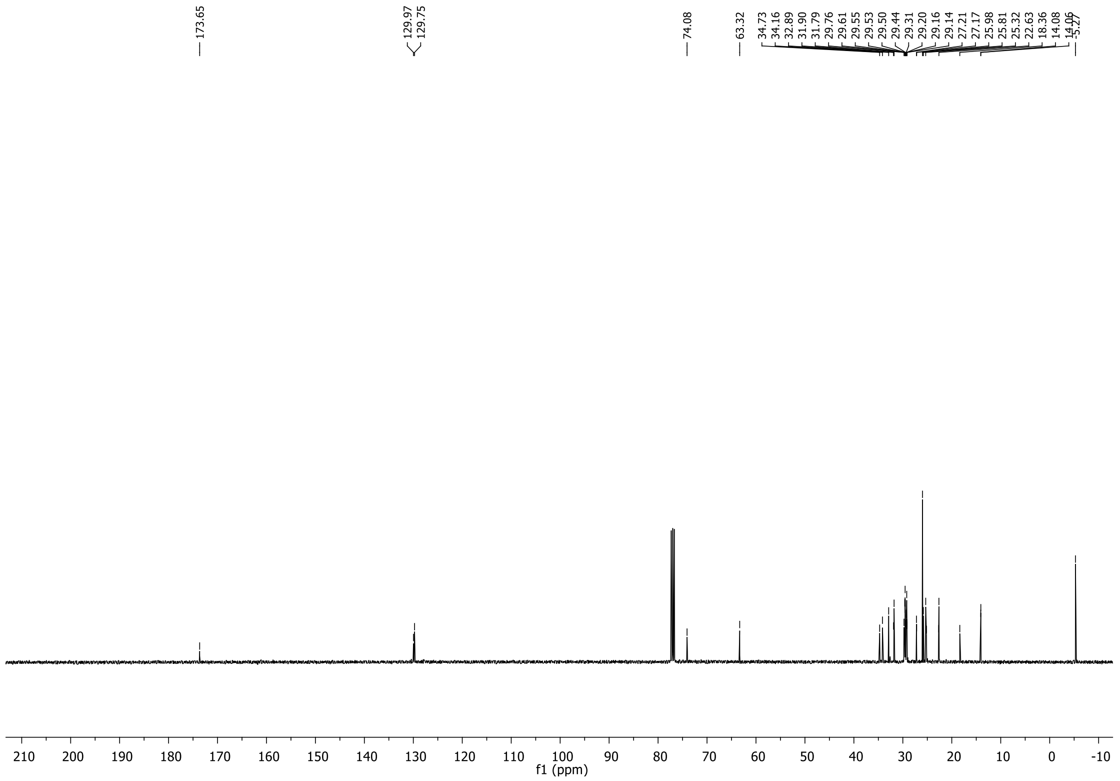
173.65
129.97
129.75
74.08
63.32
34.73
34.16
32.89
31.90
31.79
29.76
29.61
29.55
29.53
29.50
29.44
29.31
29.20
29.16
29.14
27.21
27.17
25.98
25.81
25.32
22.63
18.36
14.08
f1 (ppm)

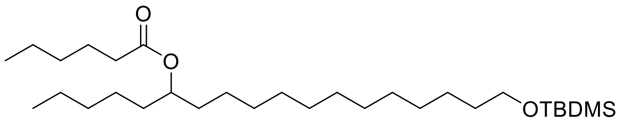
O
O
OTBDMS

(15i)

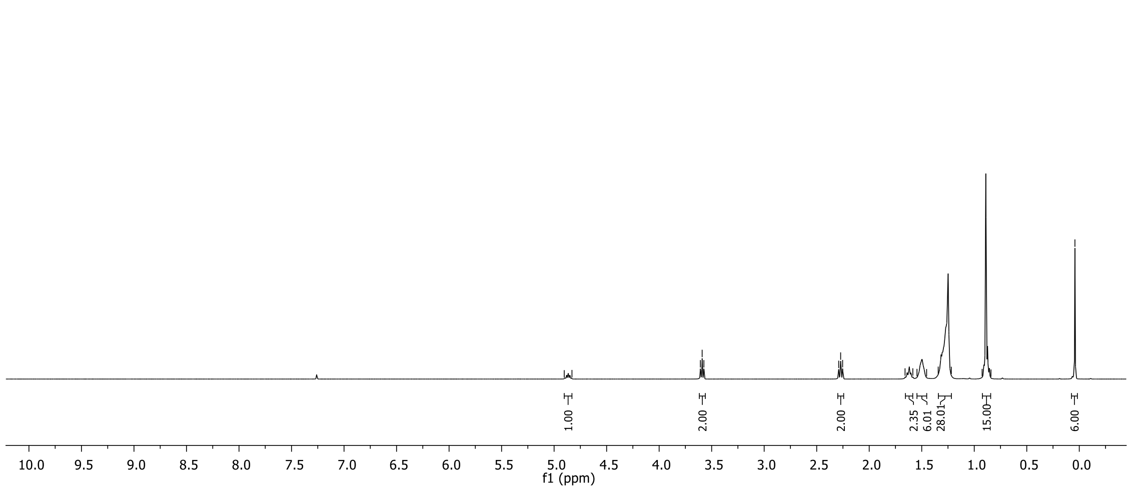
4.90
4.83
3.61
3.59
3.57
2.29
2.27
2.25
1.66
1.58
1.55
1.45
1.34
1.22
0.92
0.84
0.04
1.00
2.00
2.00
2.35
6.01
28.01
15.00
6.00
10.0 9.5 9.0 8.5 8.0 7.5 7.0 6.5 6.0 5.5 5.0 4.5 4.0 3.5 3.0 2.5 2.0 1.5 1.0 0.5 0.0
f1 (ppm)

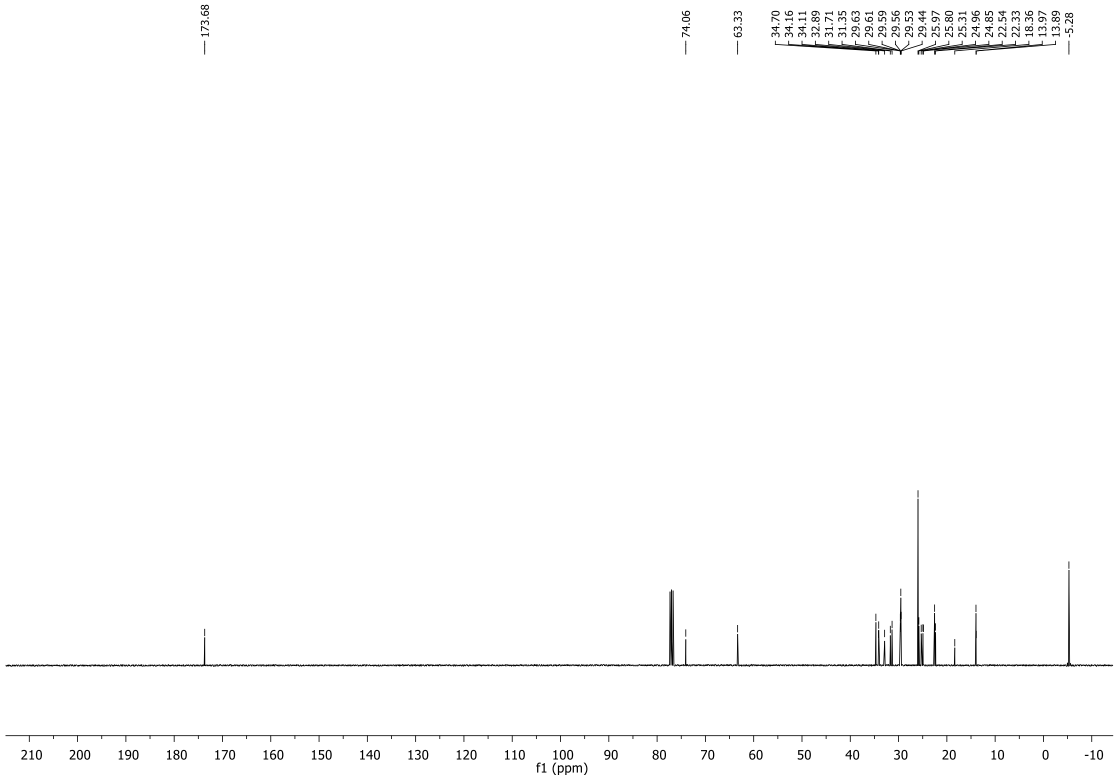
173.68
74.06
63.33
34.70
34.16
34.11
32.89
31.71
31.35
29.63
29.61
29.59
29.56
29.53
29.44
25.97
25.80
25.31
24.96
24.85
22.54
22.33
18.36
13.97
13.89
-5.28
210 200 190 180 170 160 150 140 130 120 110 100 90 80 70 60 50 40 30 20 10 0 -10
f1 (ppm)

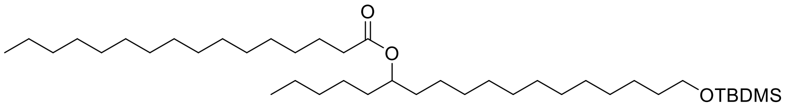
O
O
OTBDMS

(15j)

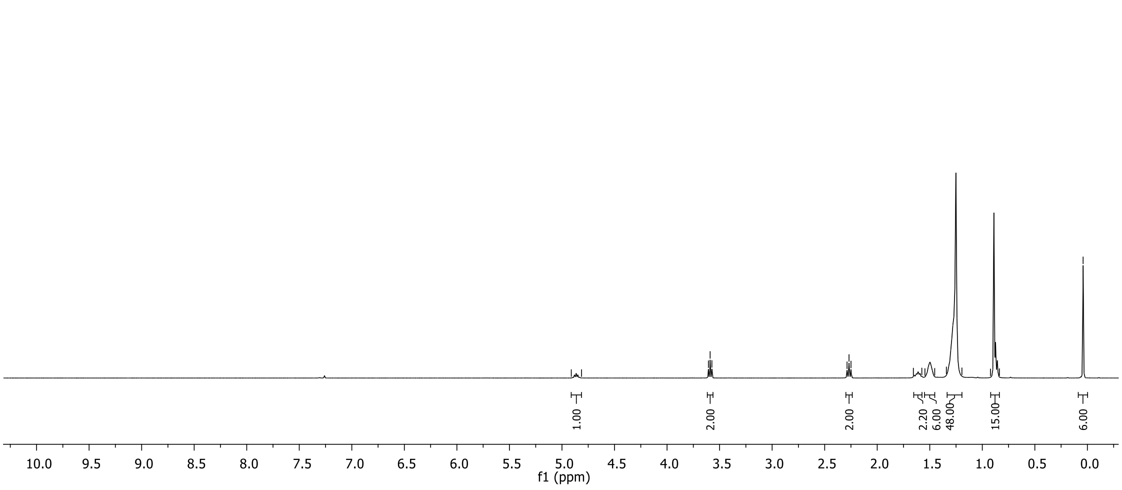
4.91
4.81
3.61
3.59
3.57
2.29
2.27
2.25
1.66
1.58
1.55
1.45
1.34
1.19
0.92
0.84
0.04
1.00
2.00
2.00
2.20
6.00
48.00
15.00
6.00
10.0 9.5 9.0 8.5 8.0 7.5 7.0 6.5 6.0 5.5 5.0 4.5 4.0 3.5 3.0 2.5 2.0 1.5 1.0 0.5 0.0
f1 (ppm)

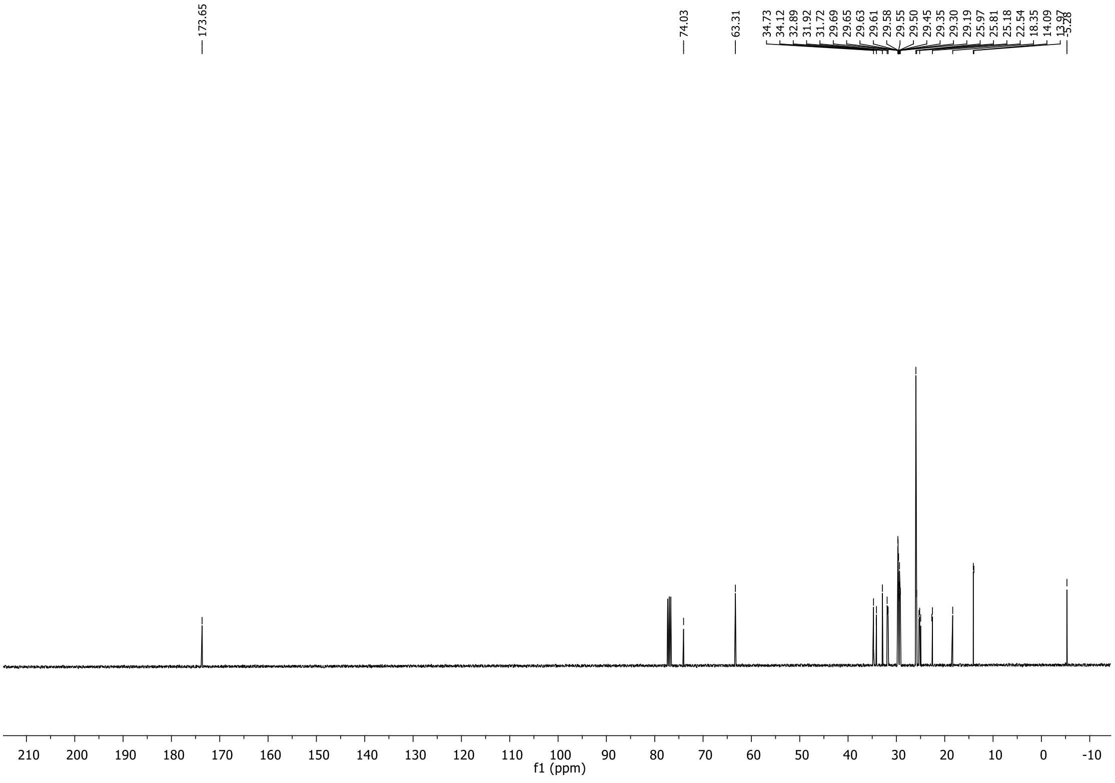
173.65
74.03
63.31
34.73
34.12
32.89
31.92
31.72
29.69
29.65
29.63
29.61
29.58
29.55
29.50
29.45
29.35
29.30
29.19
25.97
25.81
25.18
22.54
18.35
14.09
13.97
-5.28
210 200 190 180 170 160 150 140 130 120 110 100 90 80 70 60 50 40 30 20 10 0 -10
f1 (ppm)

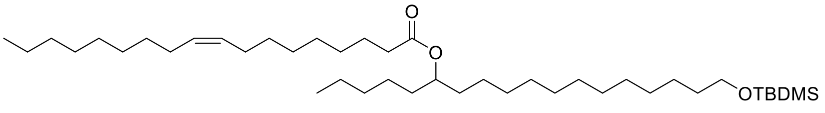
O
O
OTBDMS

(15k)

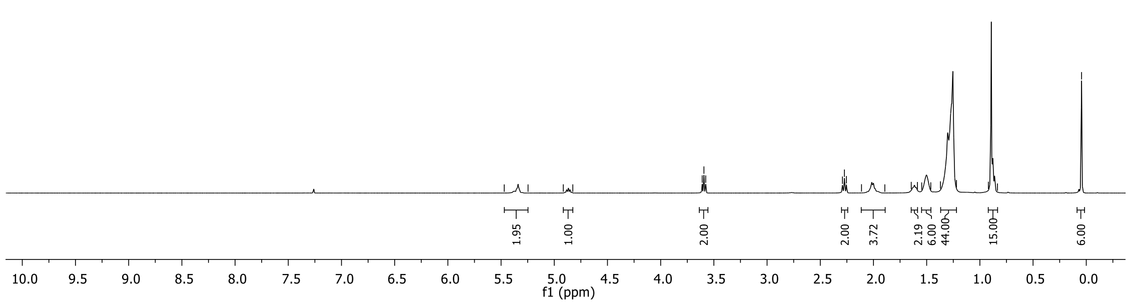
5.47
5.25
4.92
4.83
3.61
3.59
3.58
2.29
2.27
2.25
2.11
1.89
1.65
1.59
1.55
1.46
1.37
1.22
0.92
0.83
0.04
1.95
1.00
2.00
2.00
3.72
2.19
6.00
44.00
15.00
6.00
10.0 9.5 9.0 8.5 8.0 7.5 7.0 6.5 6.0 5.5 5.0 4.5 4.0 3.5 3.0 2.5 2.0 1.5 1.0 0.5 0.0
f1 (ppm)

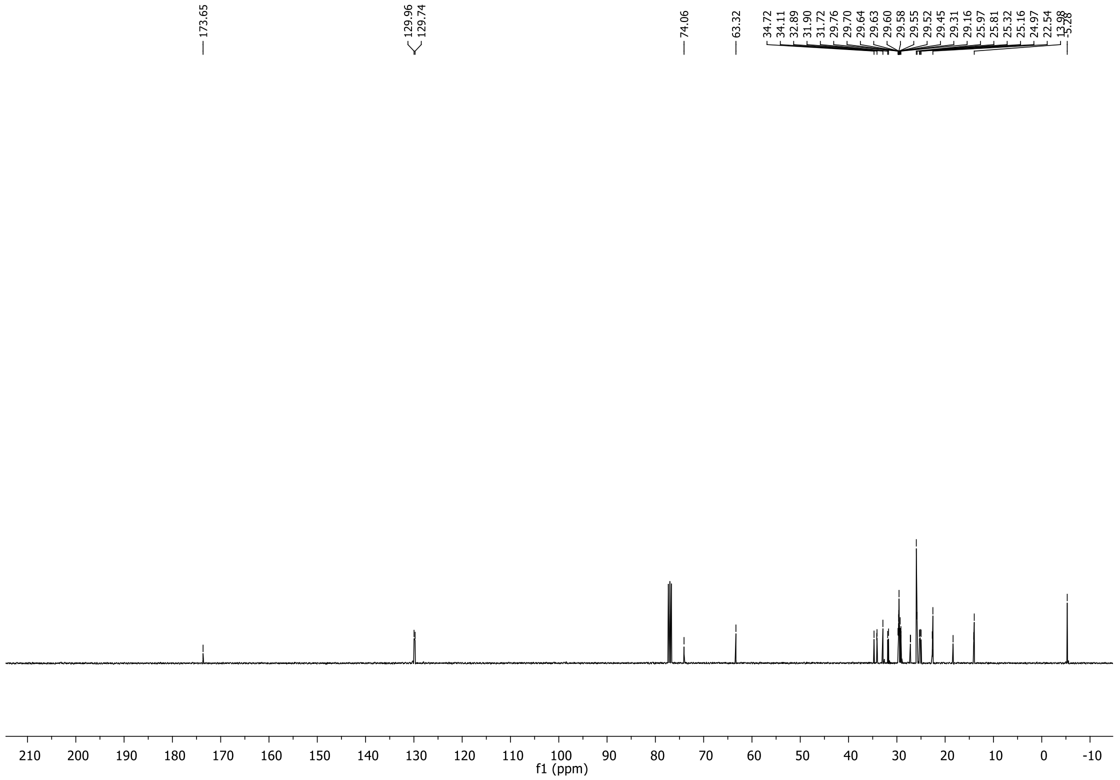
173.65
129.96
129.74
74.06
63.32
34.72
34.11
32.89
31.90
31.72
29.76
29.70
29.64
29.63
29.60
29.58
29.55
29.52
29.45
29.31
29.16
25.97
25.81
25.32
25.16
24.97
22.54
13.98
-5.28
210 200 190 180 170 160 150 140 130 120 110 100 90 80 70 60 50 40 30 20 10 0 -10
f1 (ppm)

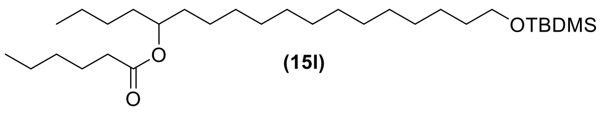
OTBDMS
O
O

(15l)

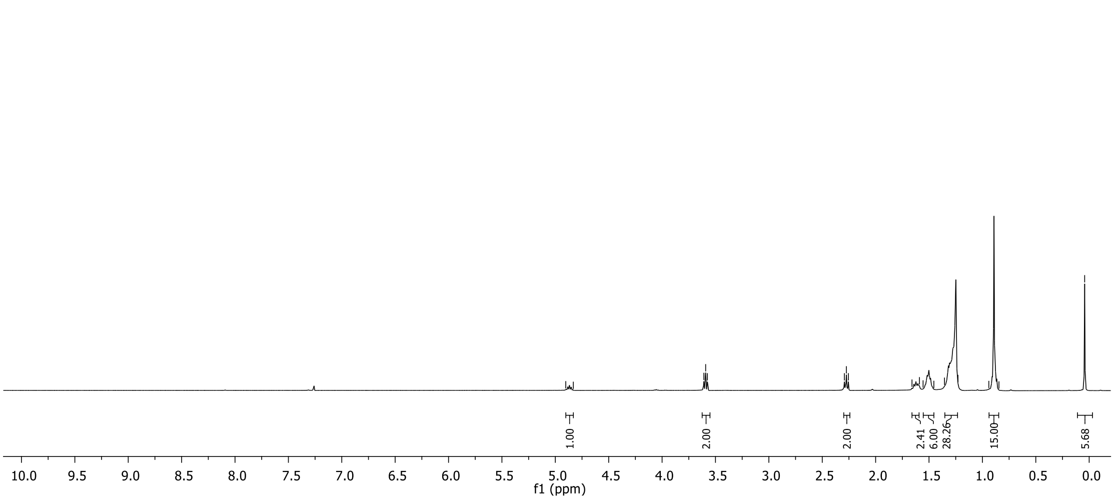
4.90
4.83
3.61
3.59
3.58
2.29
2.27
2.26
1.66
1.59
1.56
1.45
1.36
1.23
0.94
0.85
0.04
1.00
2.00
2.00
2.41
6.00
28.26
15.00
5.68
10.0 9.5 9.0 8.5 8.0 7.5 7.0 6.5 6.0 5.5 5.0 4.5 4.0 3.5 3.0 2.5 2.0 1.5 1.0 0.5 0.0
f1 (ppm)

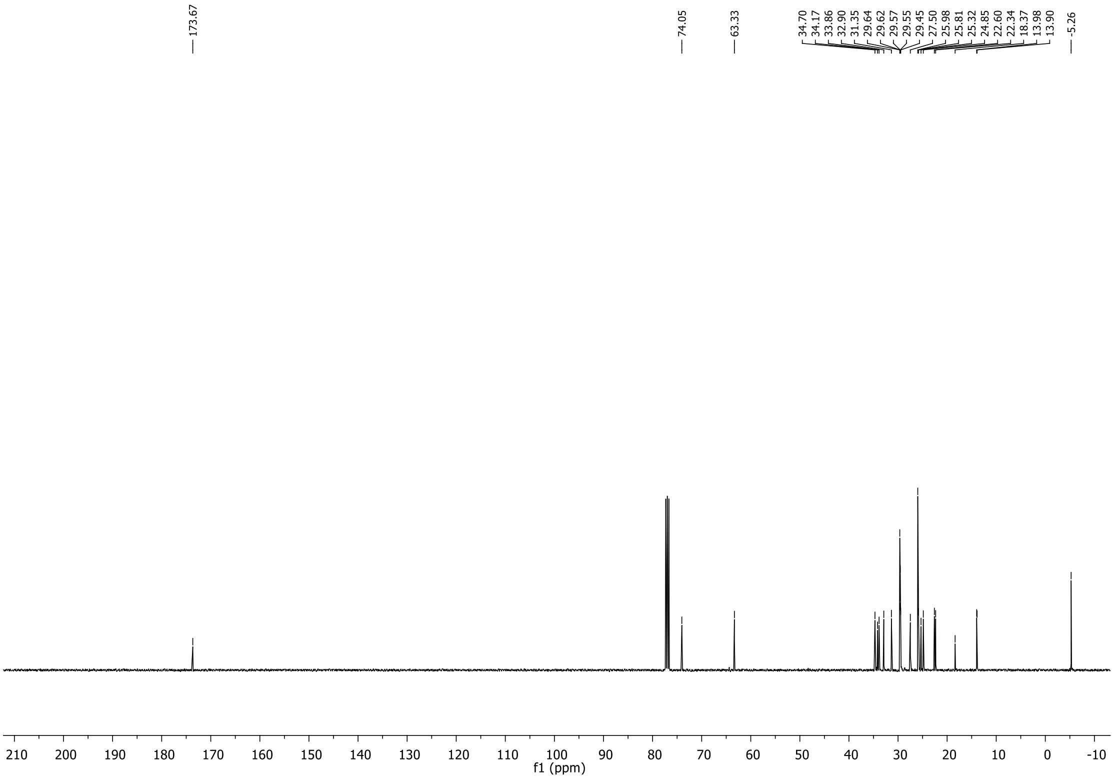
173.67
74.05
63.33
34.70
34.17
33.86
32.90
31.35
29.64
29.62
29.57
29.55
29.45
27.50
25.98
25.81
25.32
24.85
22.60
22.34
18.37
13.98
13.90
-5.26
210 200 190 180 170 160 150 140 130 120 110 100 90 80 70 60 50 40 30 20 10 0 -10
f1 (ppm)

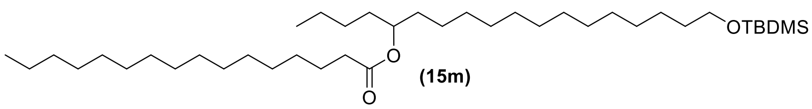
OTBDMS

(15m)

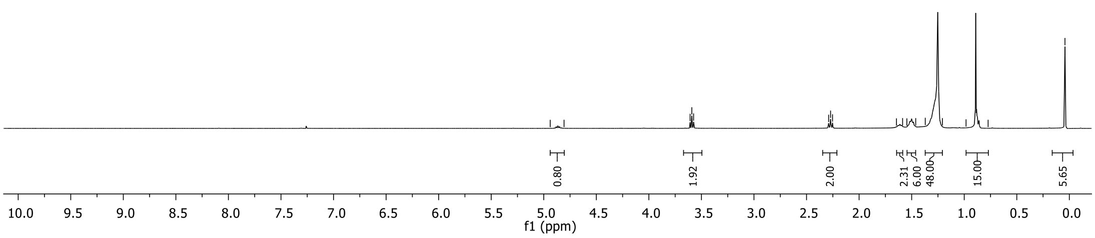
4.94
4.81
3.61
3.59
3.57
2.29
2.27
2.25
1.64
1.59
1.55
1.46
1.37
1.21
0.98
0.77
0.04
0.80
1.92
2.00
2.31
6.00
48.00
15.00
5.65
10.0
9.5
9.0
8.5
8.0
7.5
7.0
6.5
6.0
5.5
5.0
4.5
4.0
3.5
3.0
2.5
2.0
1.5
1.0
0.5
0.0
f1 (ppm)

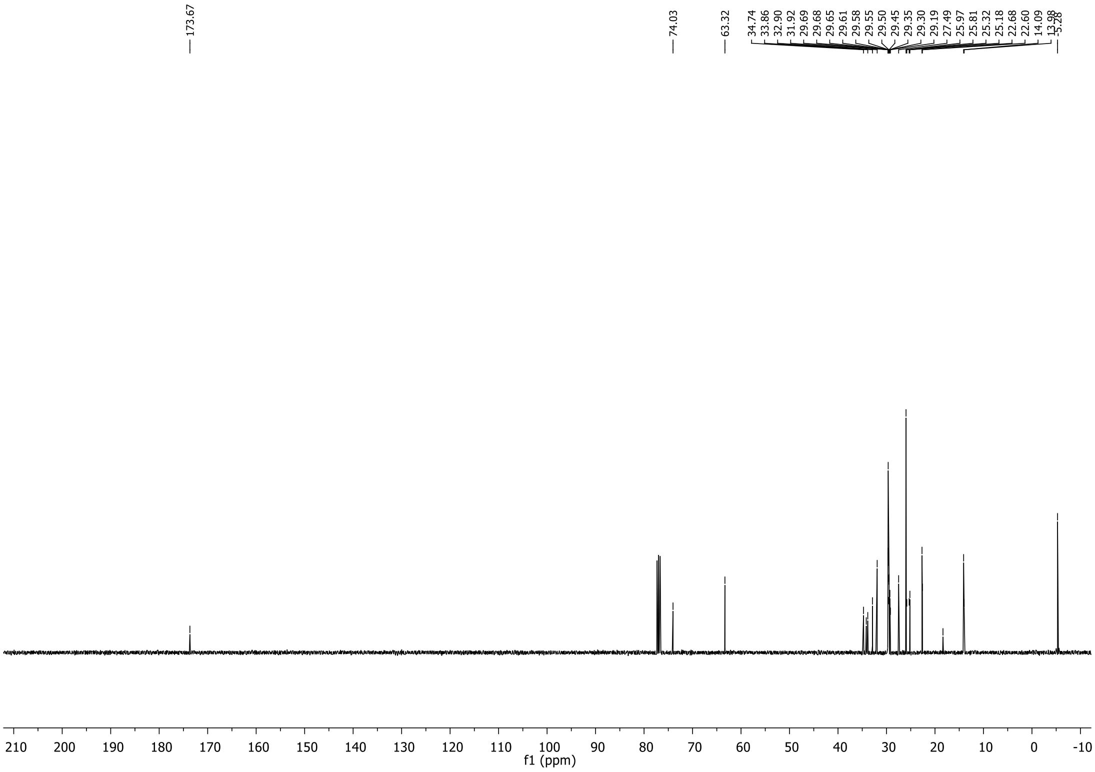
173.67
74.03
63.32
34.74
33.86
32.90
31.92
29.69
29.68
29.65
29.61
29.58
29.55
29.50
29.45
29.35
29.30
29.19
27.49
25.97
25.81
25.32
25.18
22.68
22.60
14.09
13.98
-5.28
210
200
190
180
170
160
150
140
130
120
110
100
90
80
70
60
50
40
30
20
10
0
-10
f1 (ppm)

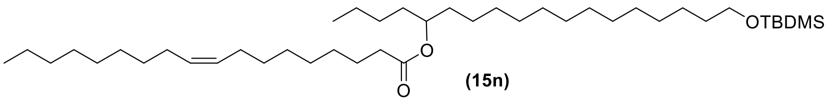
OTBDMS
O
O

(15n)

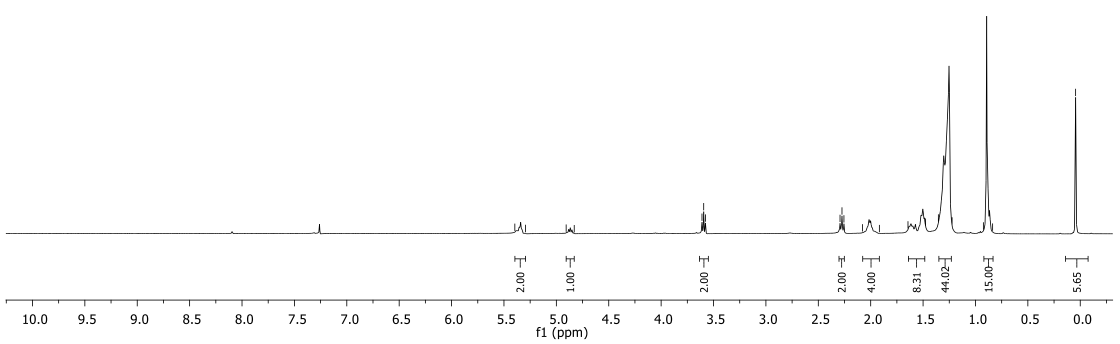
5.40
5.29
4.91
4.83
3.61
3.59
3.58
2.29
2.27
2.26
2.08
1.92
1.64
1.48
1.35
1.22
0.92
0.84
0.04
2.00
1.00
2.00
2.00
4.00
8.31
44.02
15.00
5.65
f1 (ppm)

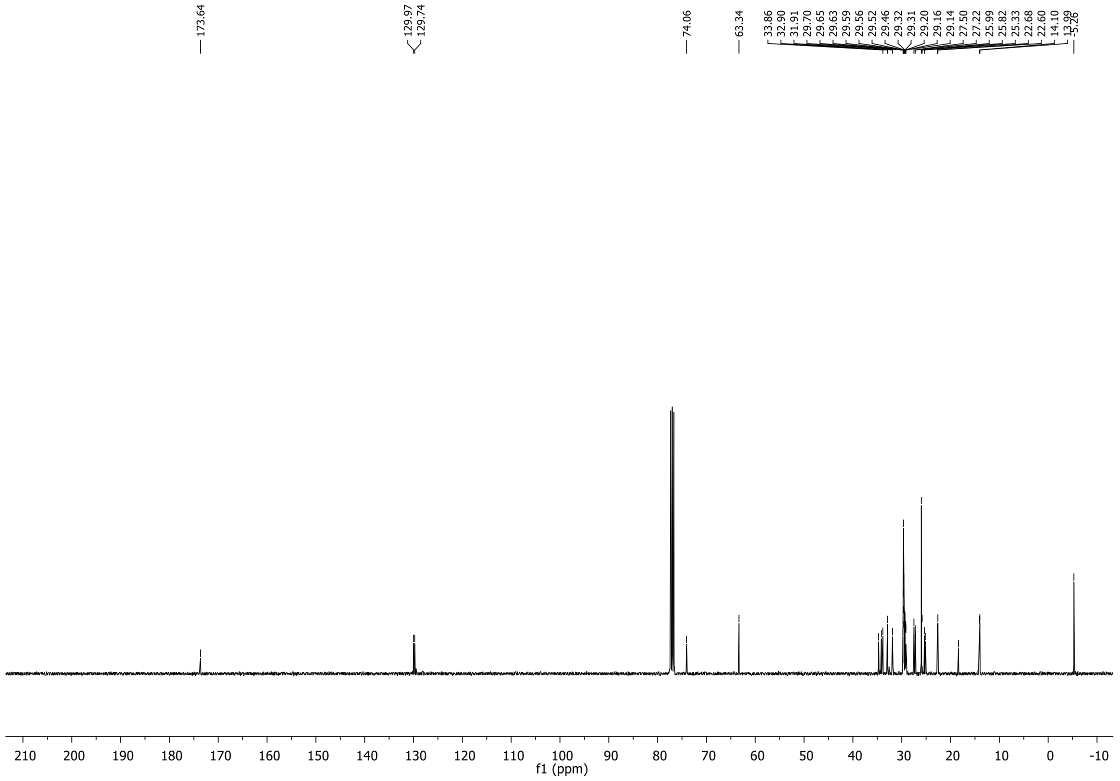
173.64
129.97
129.74
74.06
63.34
33.86
32.90
31.91
29.70
29.65
29.63
29.59
29.56
29.52
29.46
29.32
29.31
29.20
29.16
29.14
27.50
27.22
25.99
25.82
25.33
22.68
22.60
14.10
f1 (ppm)

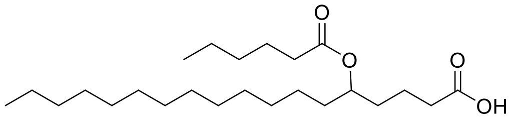

(7a)

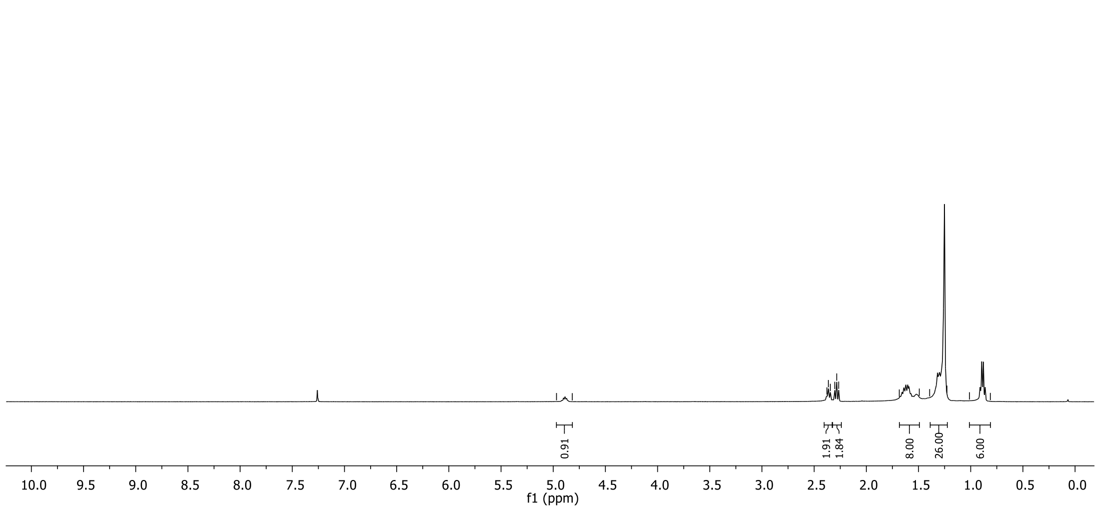
4.97
4.82
2.38
2.36
2.34
2.30
2.28
2.26
1.68
1.49
1.39
1.23
1.01
0.81
0.91
1.91
1.84
8.00
26.00
6.00
10.0 9.5 9.0 8.5 8.0 7.5 7.0 6.5 6.0 5.5 5.0 4.5 4.0 3.5 3.0 2.5 2.0 1.5 1.0 0.5 0.0
f1 (ppm)

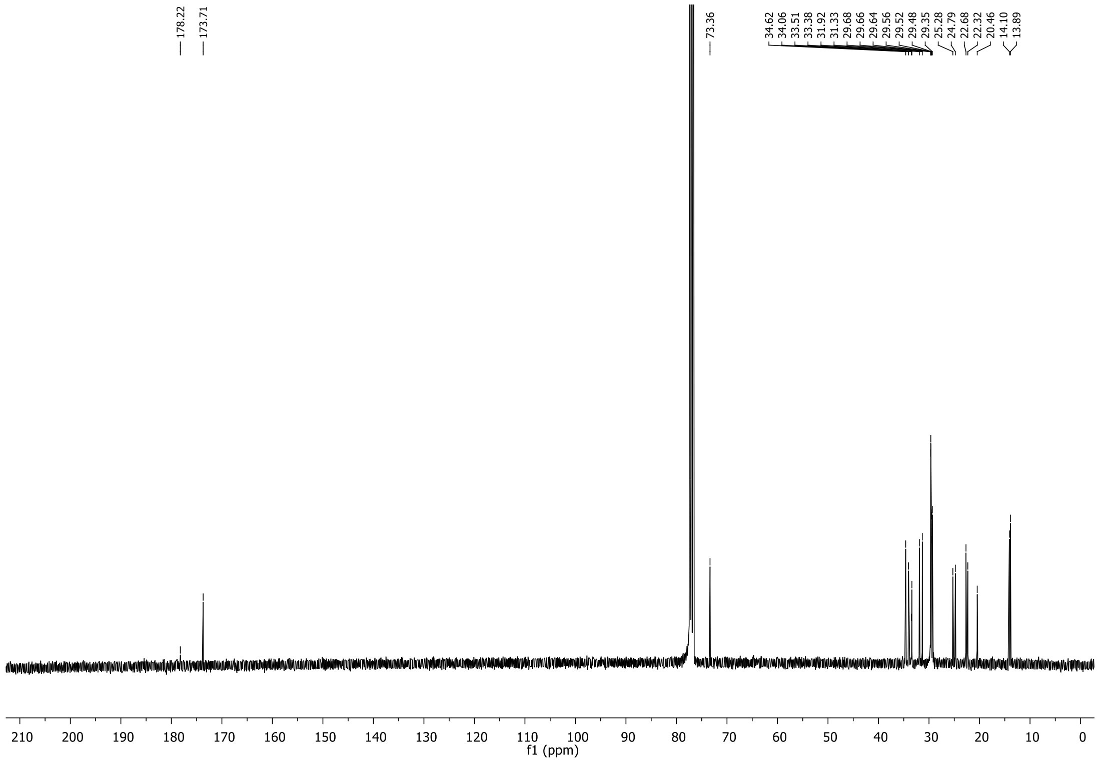
178.22
173.71
73.36
34.62
34.06
33.51
33.38
31.92
31.33
29.68
29.66
29.64
29.56
29.52
29.48
29.35
25.28
24.79
22.68
22.32
20.46
14.10
13.89
210 200 190 180 170 160 150 140 130 120 110 100 90 80 70 60 50 40 30 20 10 0
f1 (ppm)

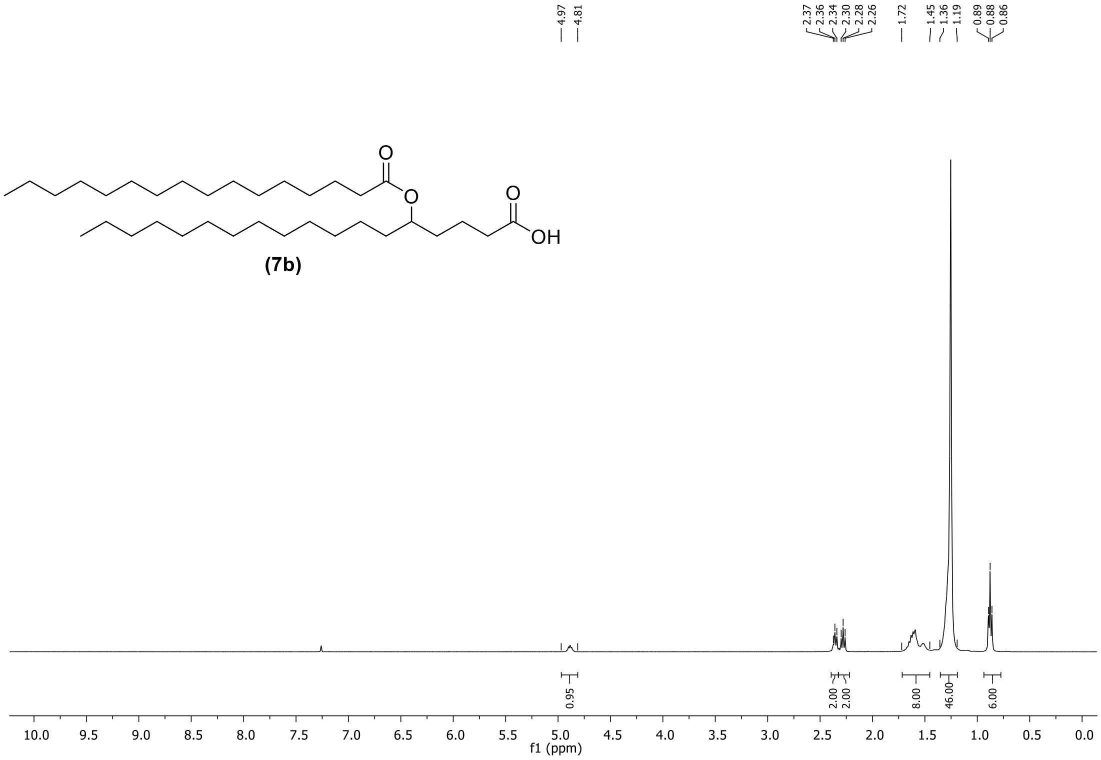
4.97
4.81
2.37
2.36
2.34
2.30
2.28
2.26
1.72
1.45
1.36
1.19
0.89
0.88
0.86
0.95
2.00
2.00
8.00
46.00
6.00
f1 (ppm)
O
O
O
OH
(7b)

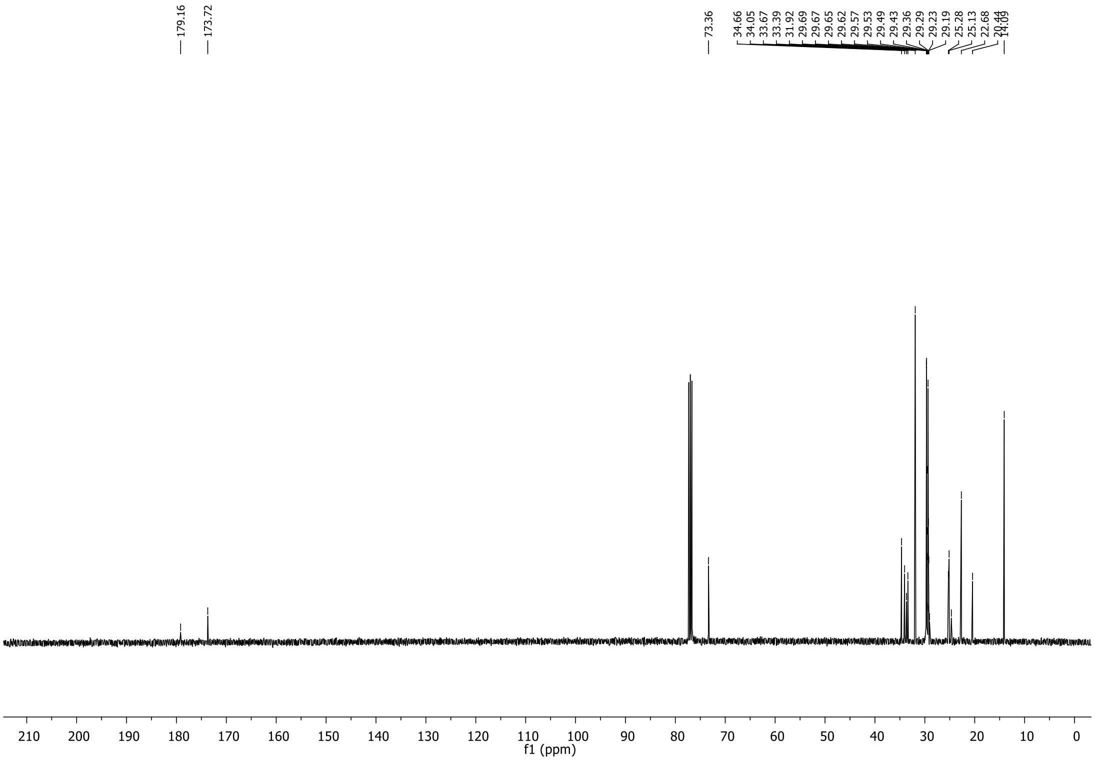
179.16
173.72
73.36
34.66
34.05
33.67
33.39
31.92
29.69
29.67
29.65
29.62
29.57
29.53
29.49
29.43
29.36
29.29
29.23
29.19
25.28
25.13
22.68
20.44
14.09
f1 (ppm)

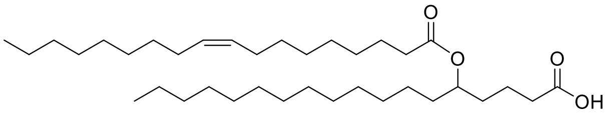
O
O
O
OH

(7c)

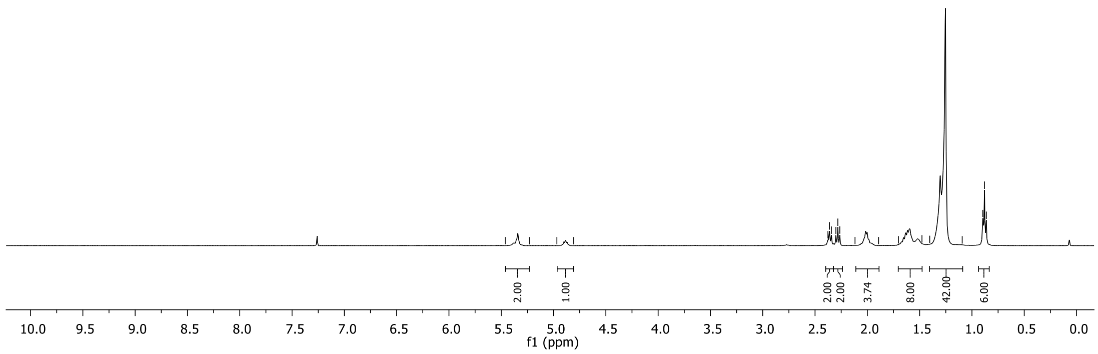
5.46
5.23
4.97
4.81
2.38
2.36
2.34
2.30
2.28
2.26
2.12
1.89
1.70
1.48
1.40
1.09
0.90
0.88
0.86
2.00
1.00
2.00
2.00
3.74
8.00
42.00
6.00
10.0 9.5 9.0 8.5 8.0 7.5 7.0 6.5 6.0 5.5 5.0 4.5 4.0 3.5 3.0 2.5 2.0 1.5 1.0 0.5 0.0
f1 (ppm)

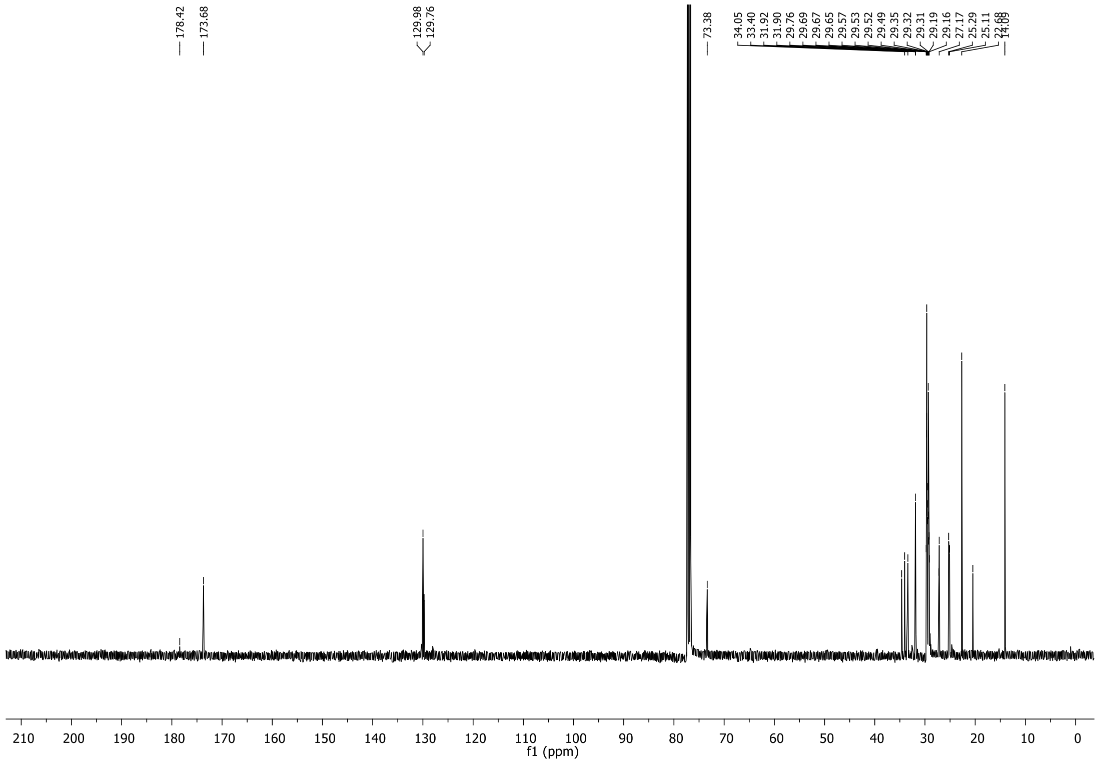
178.42
173.68
129.98
129.76
73.38
34.05
33.40
31.92
31.90
29.76
29.69
29.67
29.65
29.57
29.53
29.52
29.49
29.35
29.32
29.31
29.19
29.16
27.17
25.29
25.11
14.09
210 200 190 180 170 160 150 140 130 120 110 100 90 80 70 60 50 40 30 20 10 0
f1 (ppm)

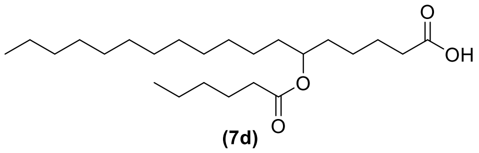
O
OH
O
O

(7d)

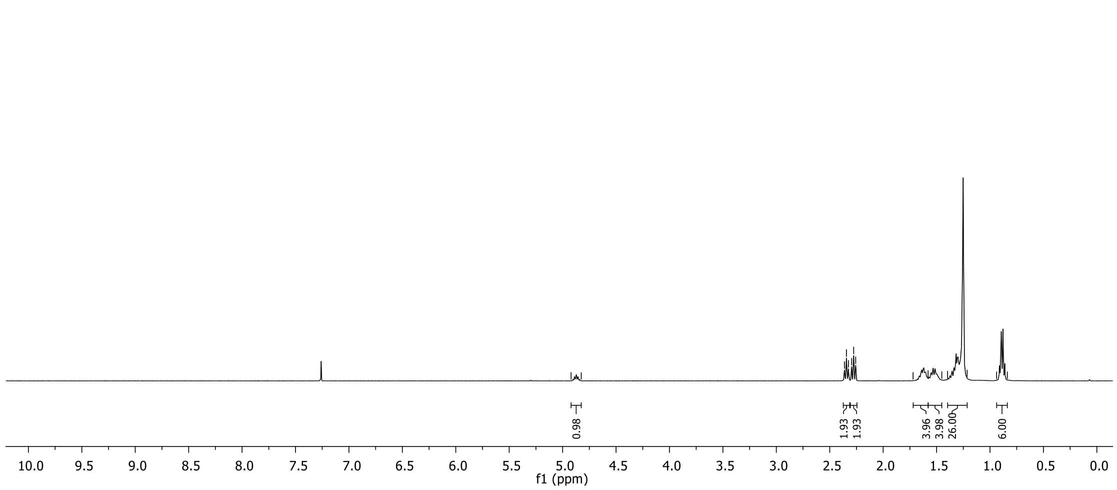
4.92
4.83
2.36
2.34
2.33
2.29
2.28
2.26
1.72
1.58
1.45
1.40
1.21
0.94
0.84
0.98
1.93
1.93
3.96
3.98
26.00
6.00
10.0 9.5 9.0 8.5 8.0 7.5 7.0 6.5 6.0 5.5 5.0 4.5 4.0 3.5 3.0 2.5 2.0 1.5 1.0 0.5 0.0
f1 (ppm)

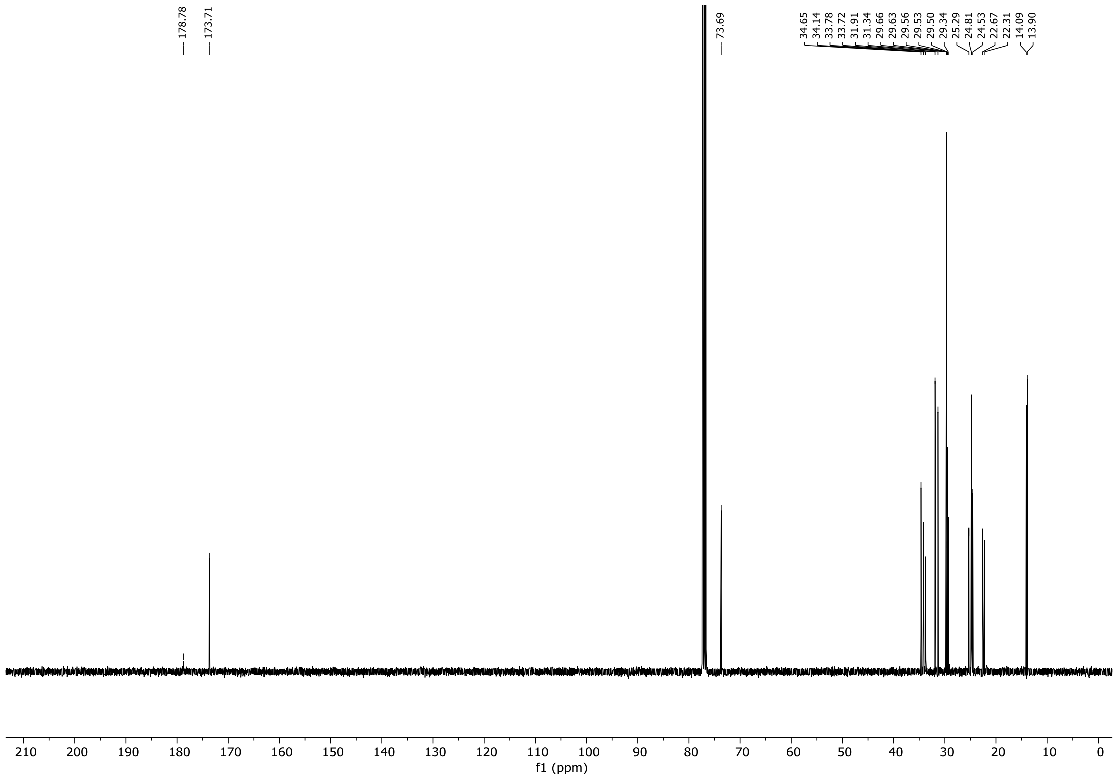
178.78
173.71
73.69
34.65
34.14
33.78
33.72
31.91
31.34
29.66
29.63
29.56
29.53
29.50
29.34
25.29
24.81
24.53
22.67
22.31
14.09
13.90
210 200 190 180 170 160 150 140 130 120 110 100 90 80 70 60 50 40 30 20 10 0
f1 (ppm)

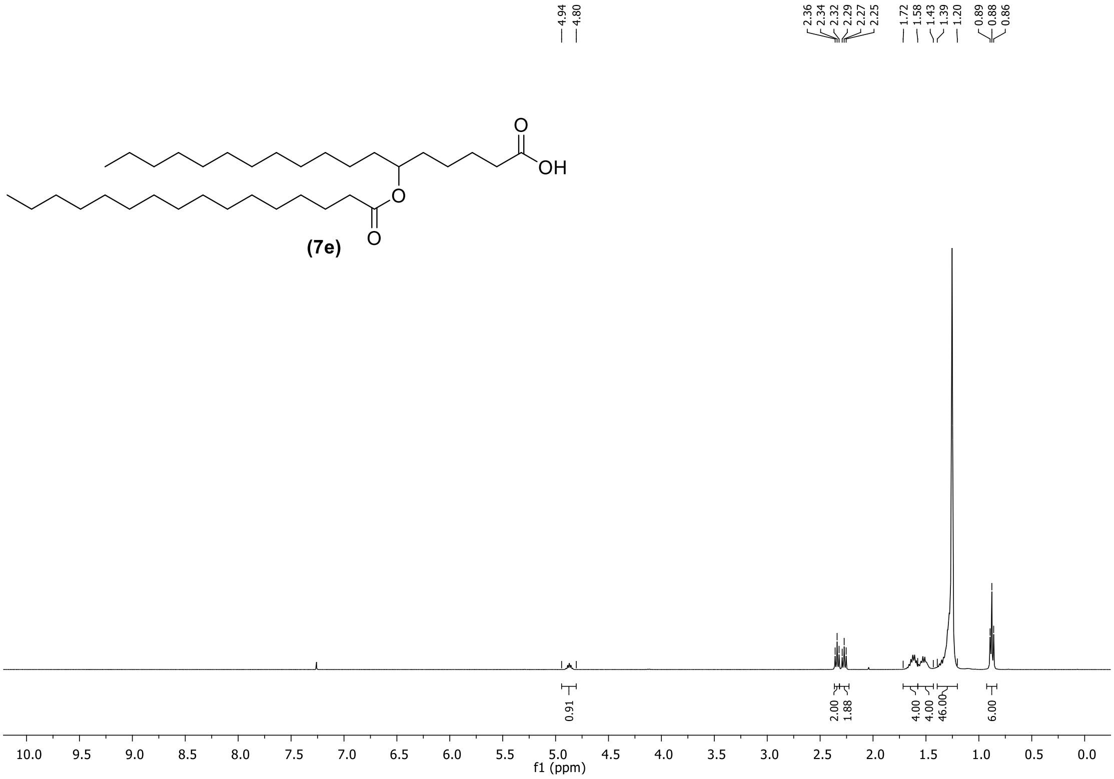
O
OH
O
O
(7e)
4.94
4.80
2.36
2.34
2.32
2.29
2.27
2.25
1.72
1.58
1.43
1.39
1.20
0.89
0.88
0.86
0.91
2.00
1.88
4.00
4.00
46.00
6.00
10.0 9.5 9.0 8.5 8.0 7.5 7.0 6.5 6.0 5.5 5.0 4.5 4.0 3.5 3.0 2.5 2.0 1.5 1.0 0.5 0.0
f1 (ppm)

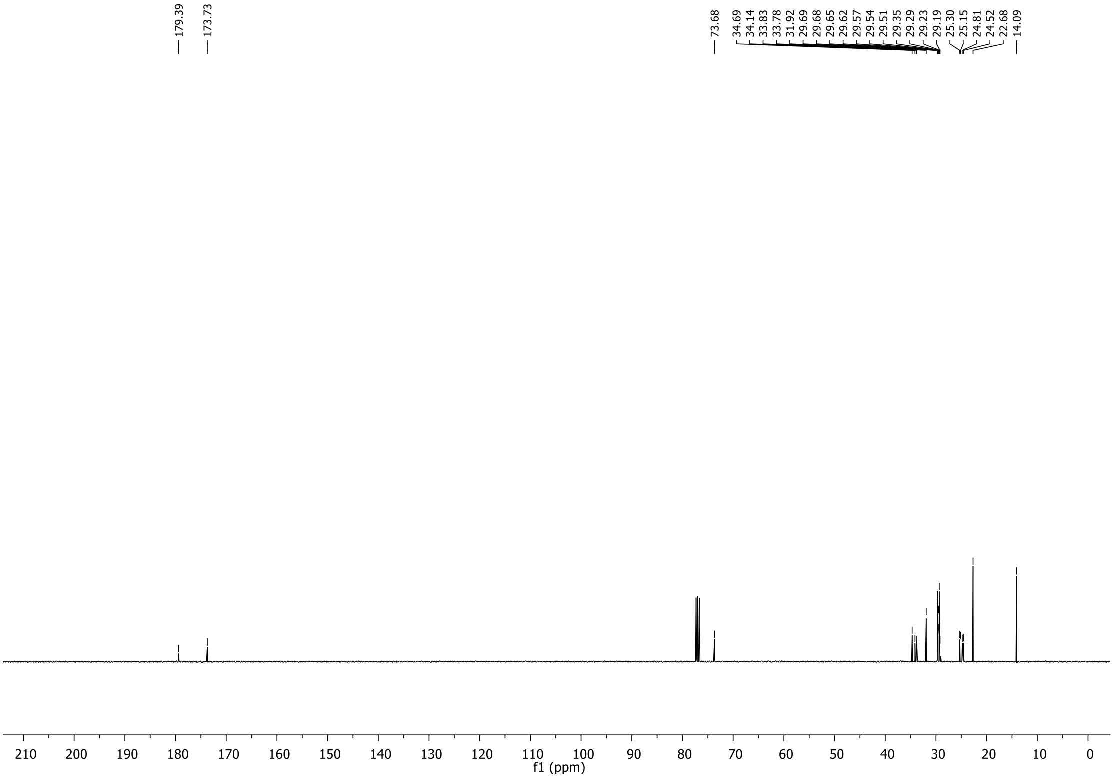
179.39
173.73
73.68
34.69
34.14
33.83
33.78
31.92
29.69
29.68
29.65
29.62
29.57
29.54
29.51
29.35
29.29
29.23
29.19
25.30
25.15
24.81
24.52
22.68
14.09
210 200 190 180 170 160 150 140 130 120 110 100 90 80 70 60 50 40 30 20 10 0
f1 (ppm)

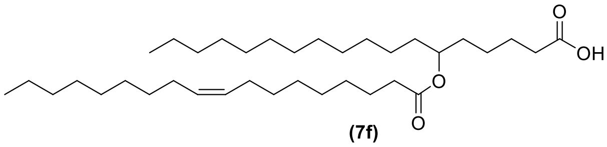

(7f)

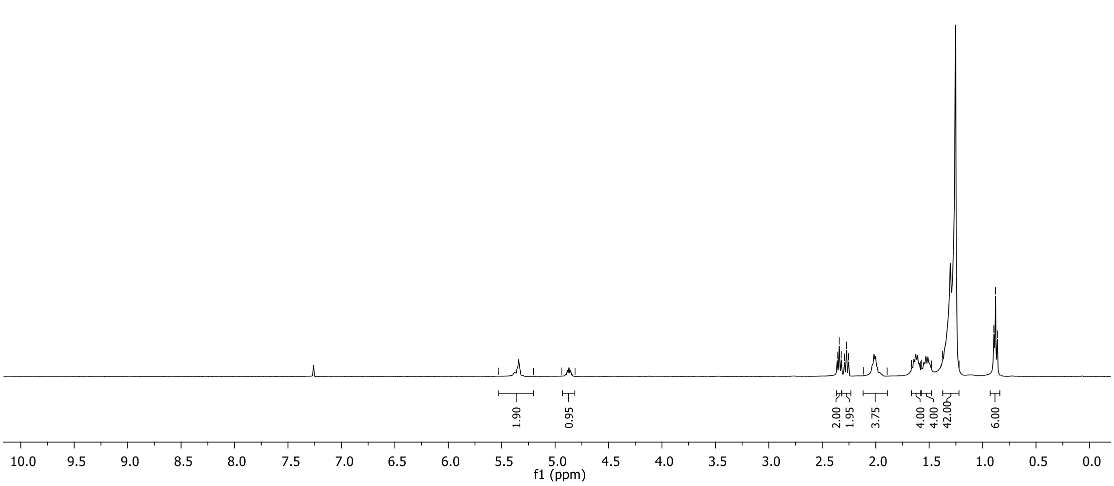

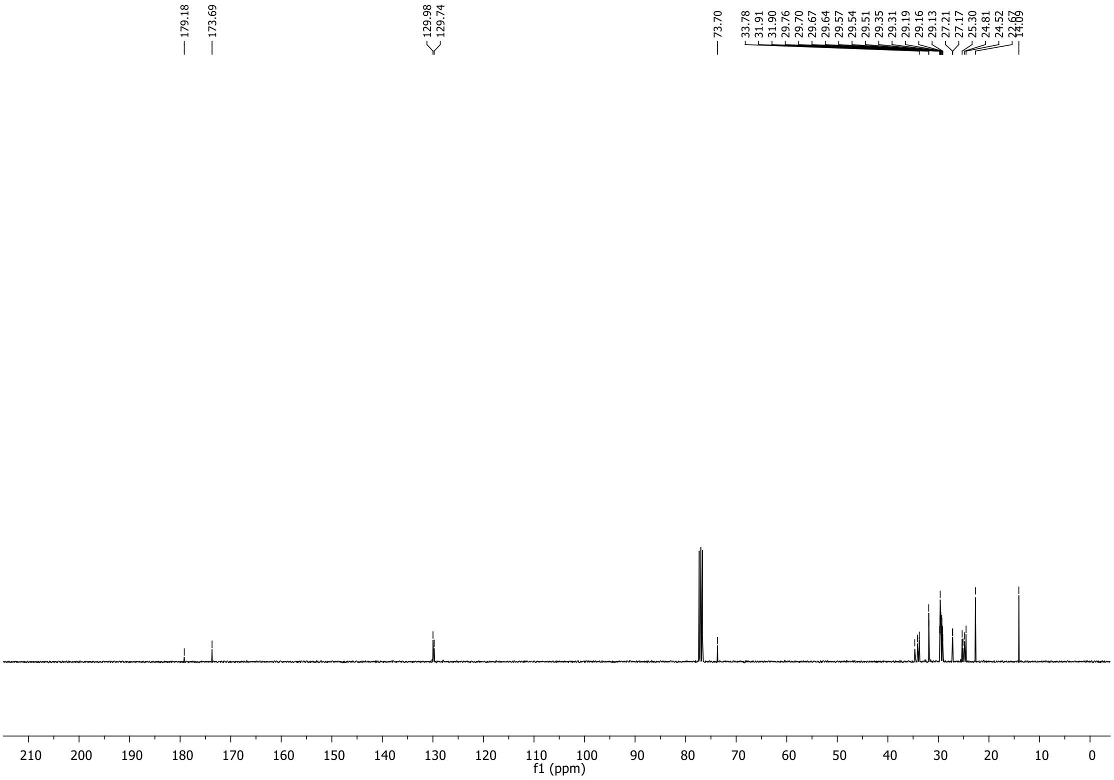

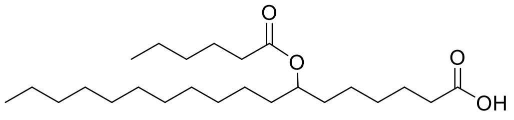

(7g)

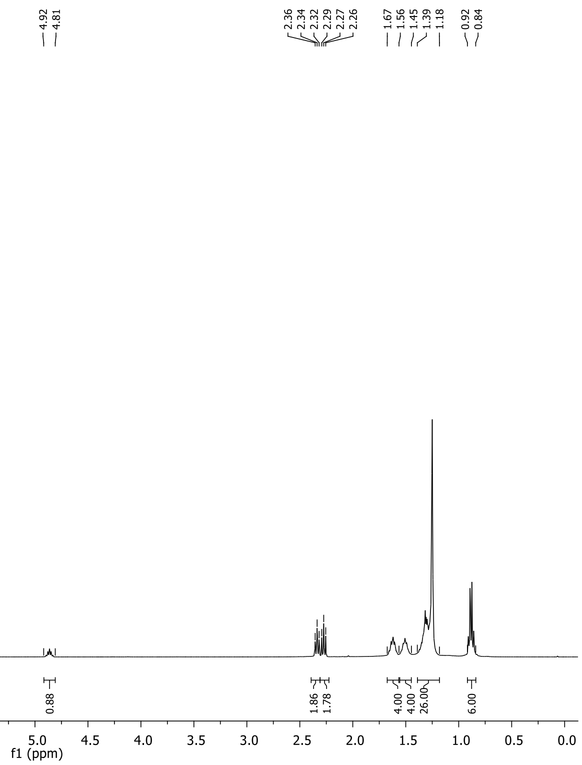

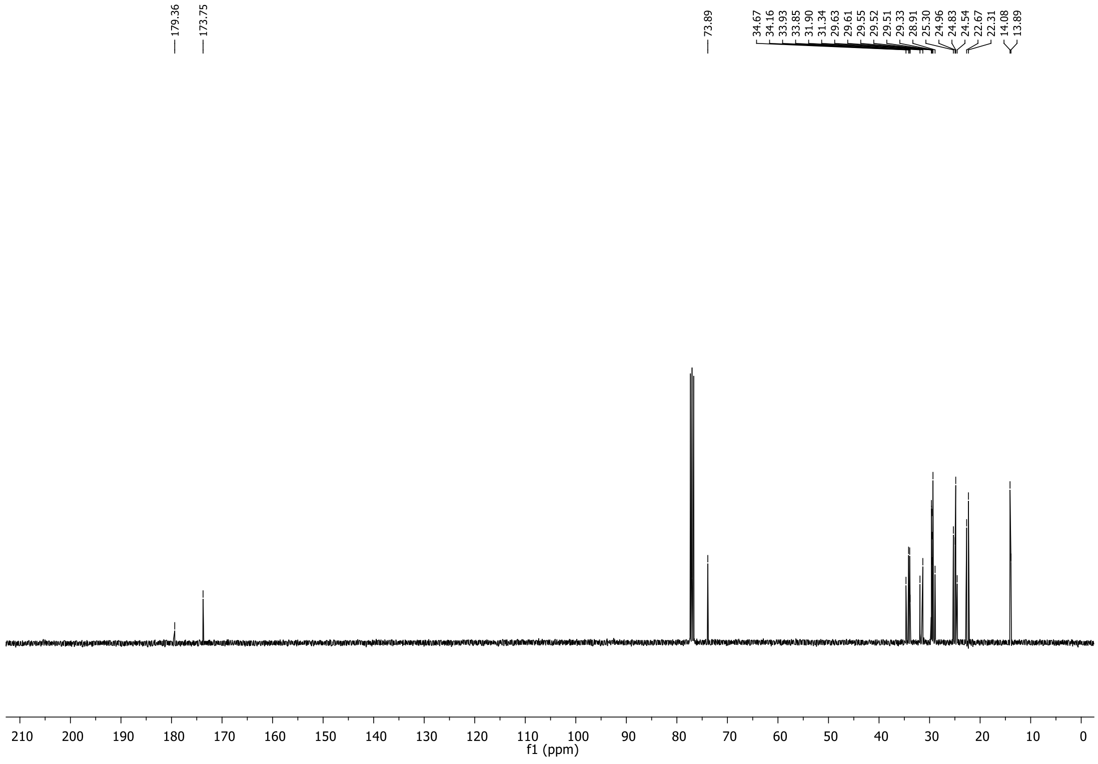

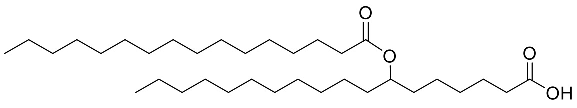
O
O
O
OH

(7h)

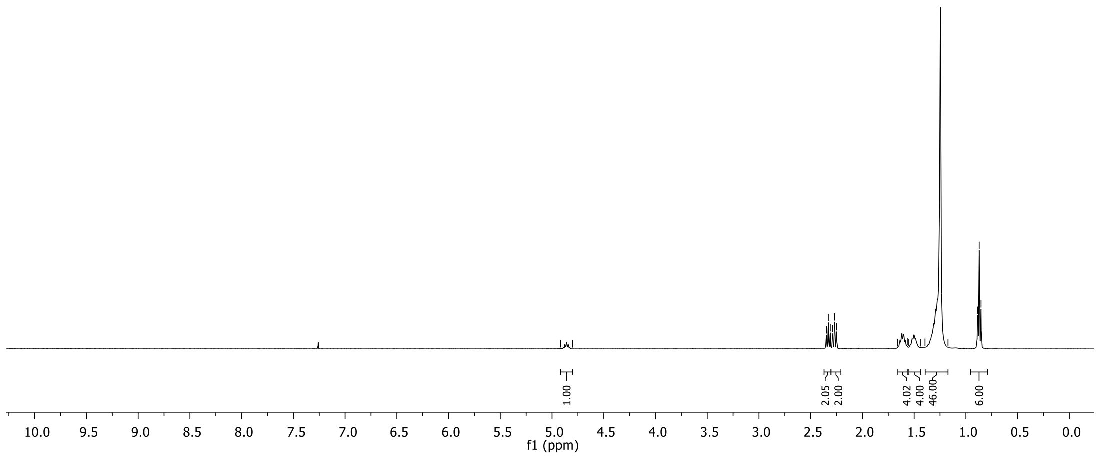
4.92
4.80
2.35
2.33
2.31
2.29
2.27
2.25
1.66
1.57
1.55
1.44
1.40
1.17
0.89
0.87
0.85
1.00
2.05
2.00
4.02
4.00
46.00
6.00
10.0 9.5 9.0 8.5 8.0 7.5 7.0 6.5 6.0 5.5 5.0 4.5 4.0 3.5 3.0 2.5 2.0 1.5 1.0 0.5 0.0
f1 (ppm)

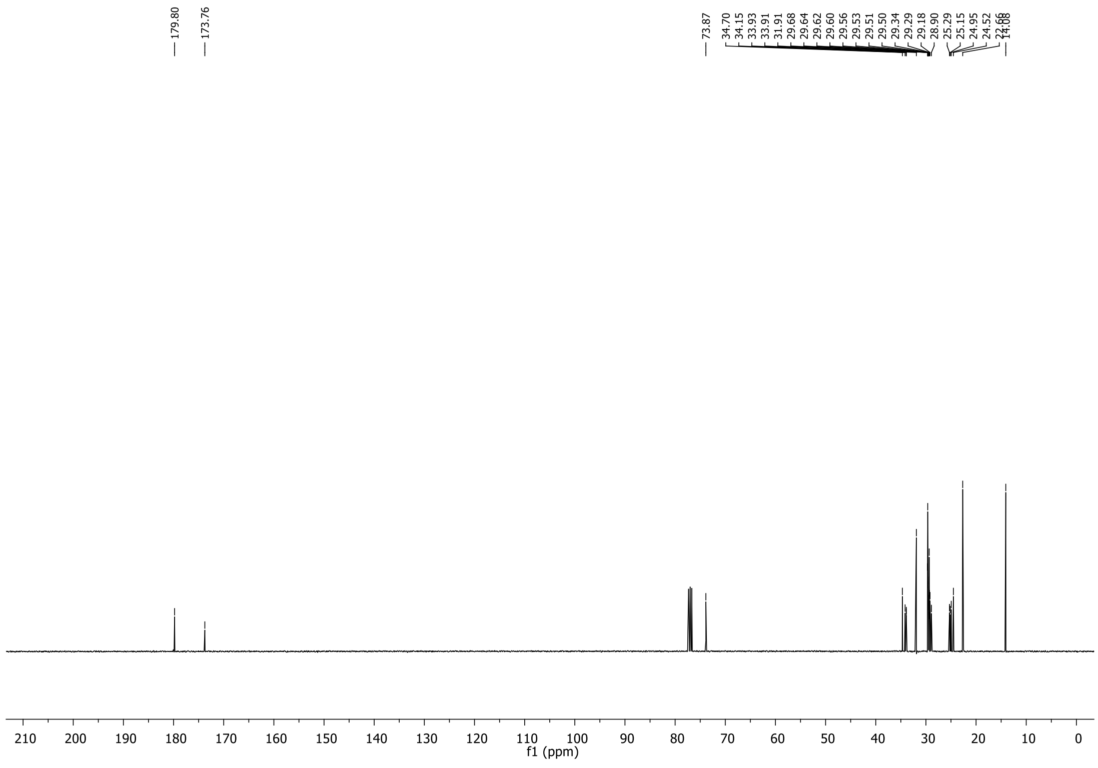
179.80
173.76
73.87
34.70
34.15
33.93
33.91
31.91
29.68
29.64
29.62
29.60
29.56
29.53
29.51
29.50
29.34
29.29
29.18
28.90
25.29
25.15
24.95
24.52
22.66
14.08
210 200 190 180 170 160 150 140 130 120 110 100 90 80 70 60 50 40 30 20 10 0
f1 (ppm)

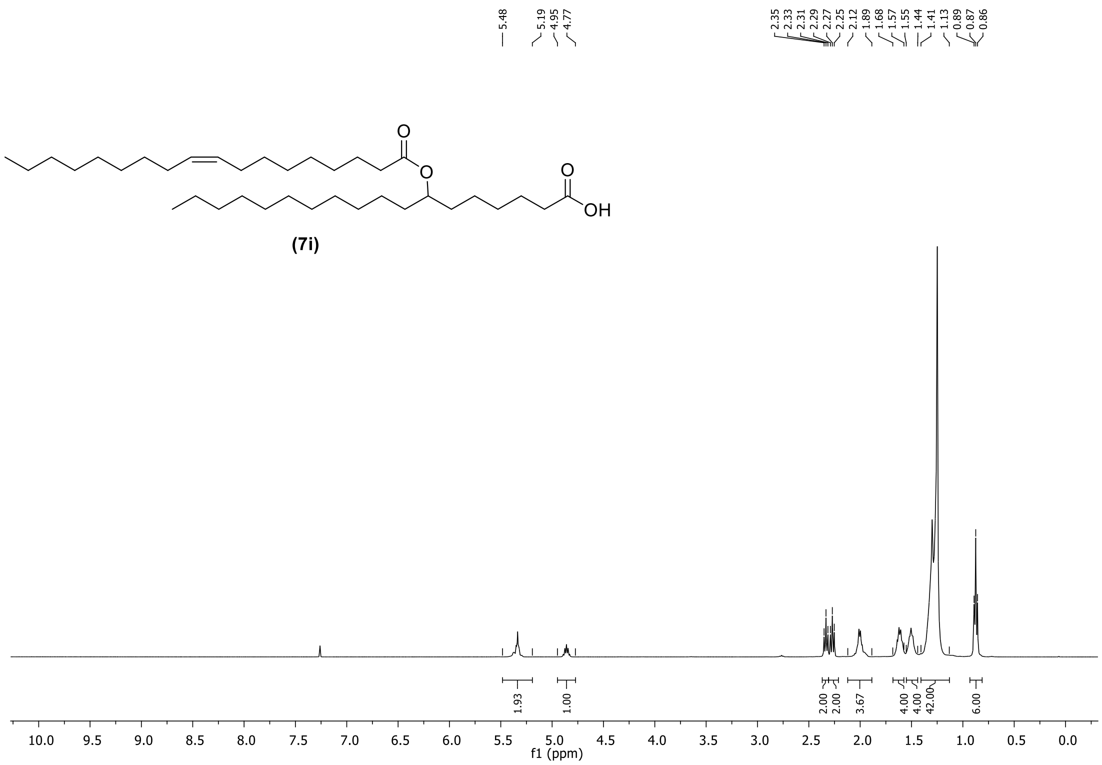
5.48
5.19
4.95
4.77
2.35
2.33
2.31
2.29
2.27
2.25
2.12
1.89
1.68
1.57
1.55
1.44
1.41
1.13
0.89
0.87
0.86
O
O
O
OH
(7i)
1.93
1.00
2.00
2.00
3.67
4.00
4.00
42.00
6.00
f1 (ppm)

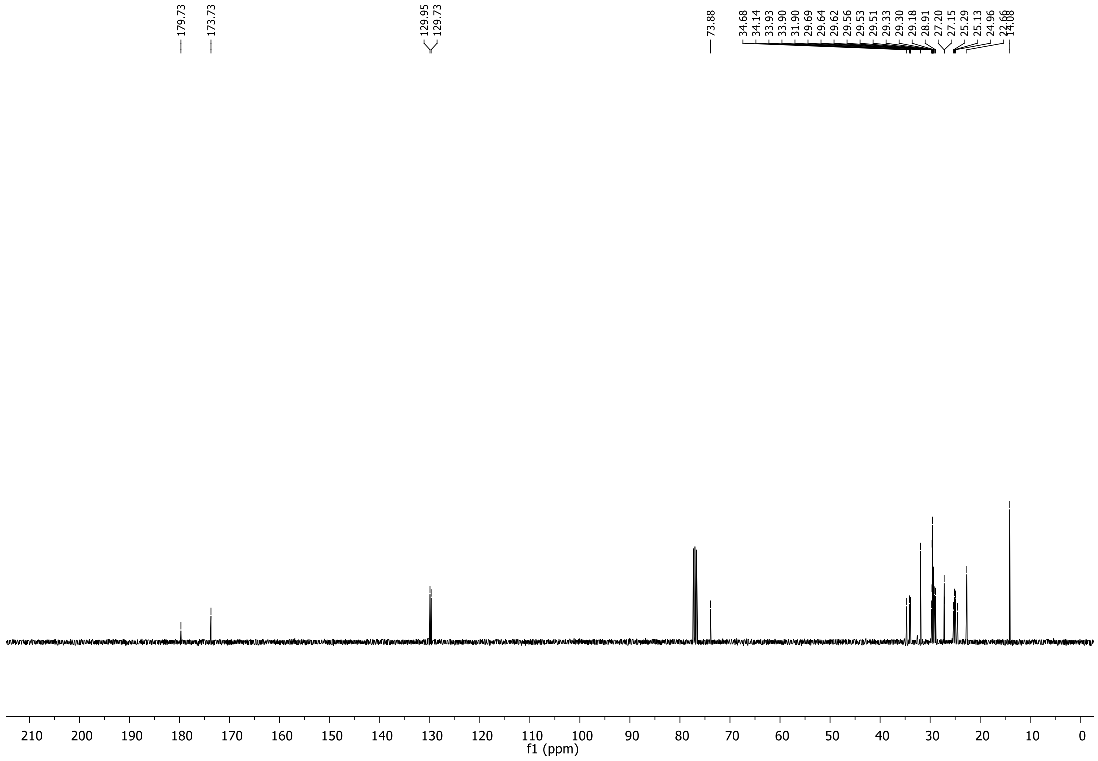
179.73
173.73
129.95
129.73
73.88
34.68
34.14
33.93
33.90
31.90
29.69
29.64
29.62
29.56
29.53
29.51
29.33
29.30
29.18
28.91
27.20
27.15
25.29
25.13
24.96
22.66
14.08
f1 (ppm)

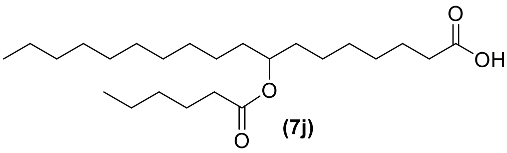

(7j)

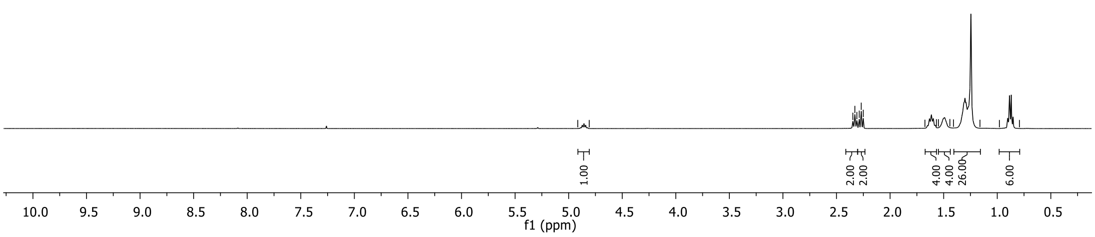

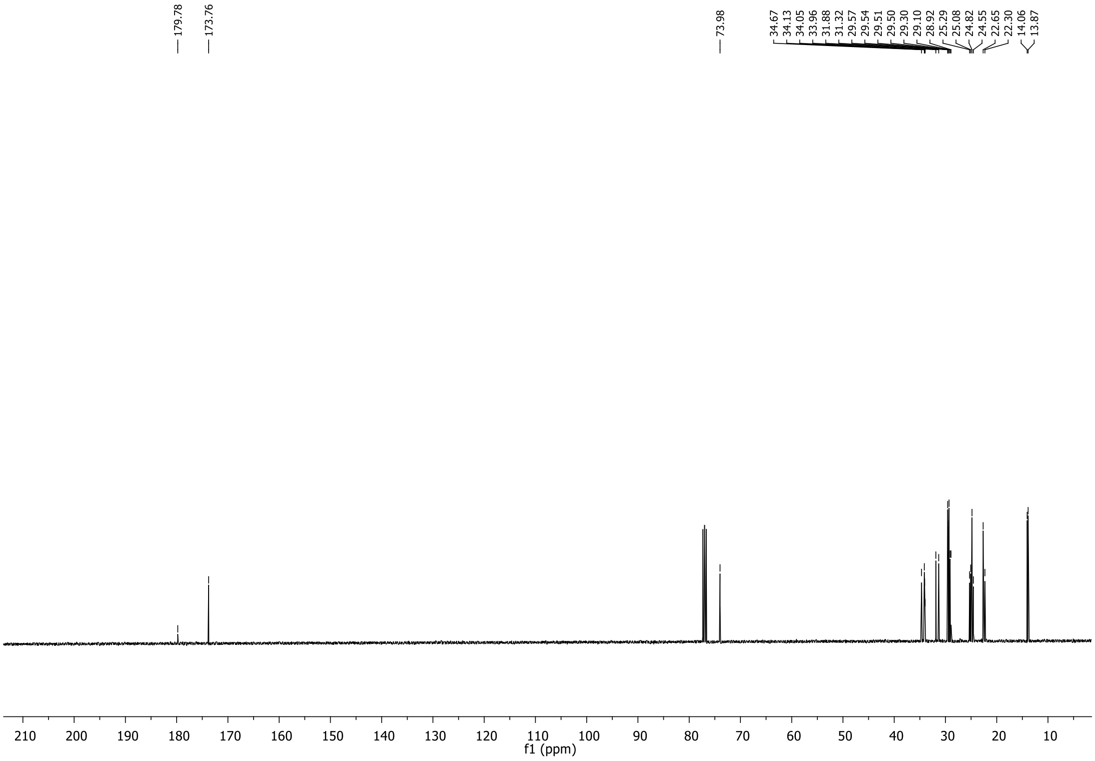

(7k)

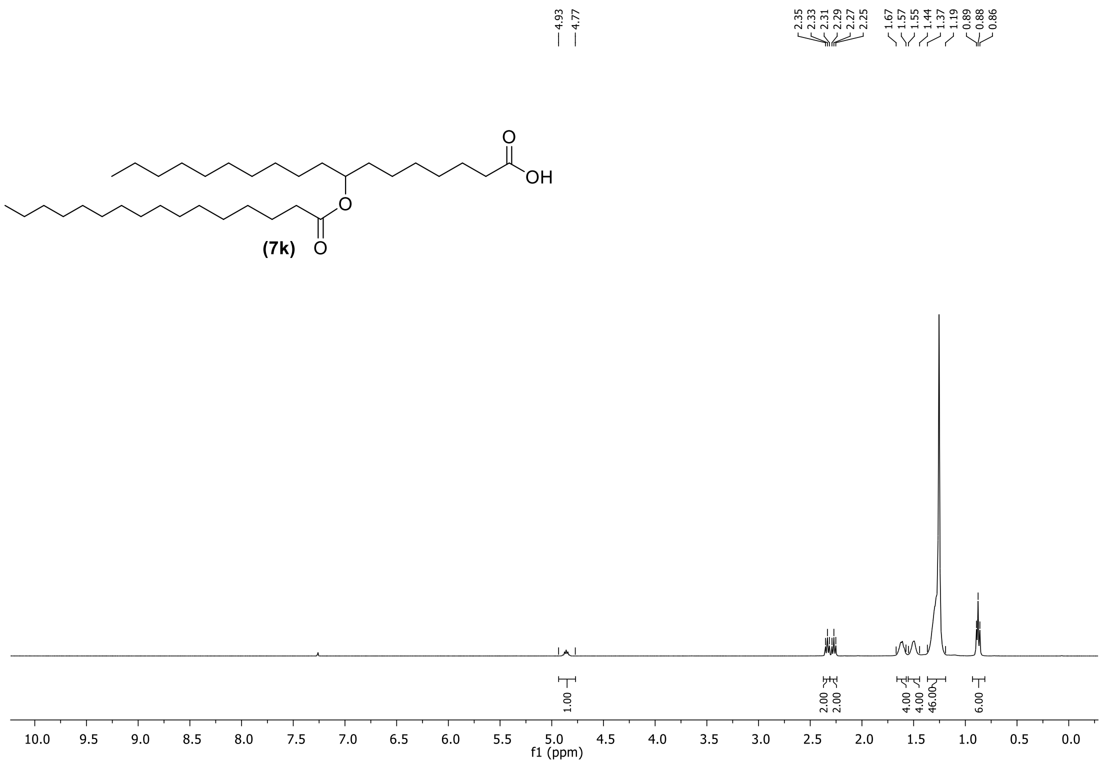
O
OH
O
O
4.93
4.77
2.35
2.33
2.31
2.29
2.27
2.25
1.67
1.57
1.55
1.44
1.37
1.19
0.89
0.88
0.86
1.00
2.00
2.00
4.00
4.00
46.00
6.00
10.0 9.5 9.0 8.5 8.0 7.5 7.0 6.5 6.0 5.5 5.0 4.5 4.0 3.5 3.0 2.5 2.0 1.5 1.0 0.5 0.0
f1 (ppm)

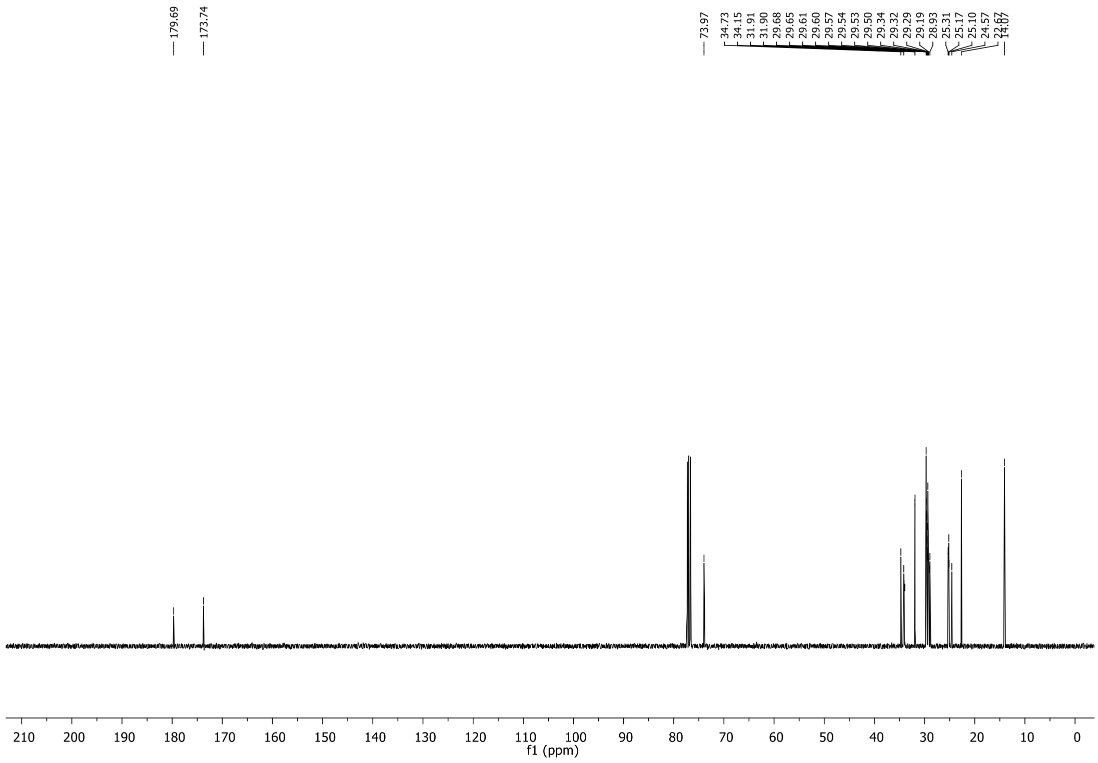
179.69
173.74
73.97
34.73
34.15
31.91
31.90
29.68
29.65
29.61
29.60
29.57
29.54
29.53
29.50
29.34
29.32
29.29
29.19
28.93
25.31
25.17
25.10
24.57
22.67
14.07
210 200 190 180 170 160 150 140 130 120 110 100 90 80 70 60 50 40 30 20 10 0
f1 (ppm)

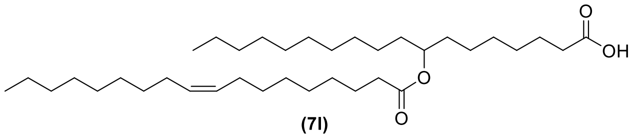

O
OH
O
O

(7l)

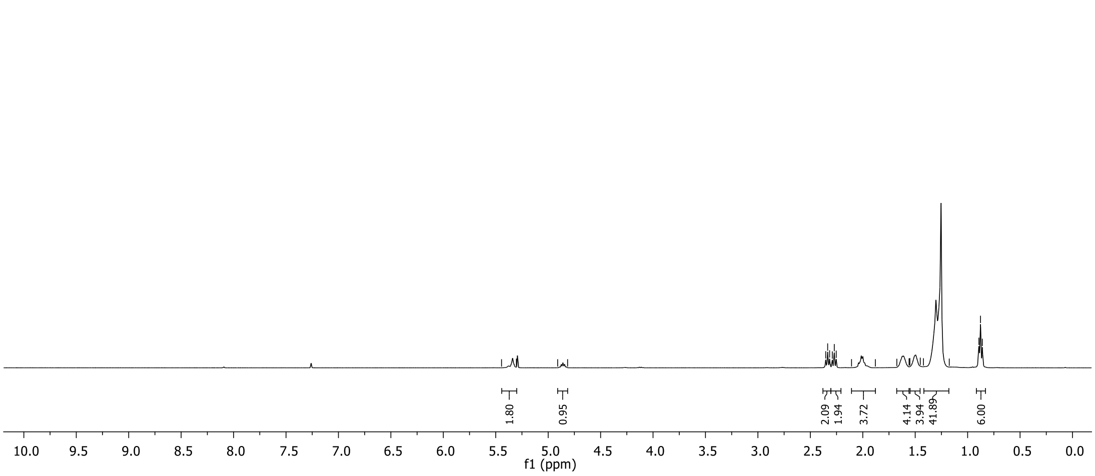

5.44
5.30
4.91
4.81
2.35
2.33
2.32
2.29
2.27
2.25
2.11
1.88
1.68
1.56
1.55
1.45
1.42
1.18
0.89
0.88
0.86
1.80
0.95
2.09
1.94
3.72
4.14
3.94
41.89
6.00
10.0
9.5
9.0
8.5
8.0
7.5
7.0
6.5
6.0
5.5
5.0
4.5
4.0
3.5
3.0
2.5
2.0
1.5
1.0
0.5
0.0
f1 (ppm)

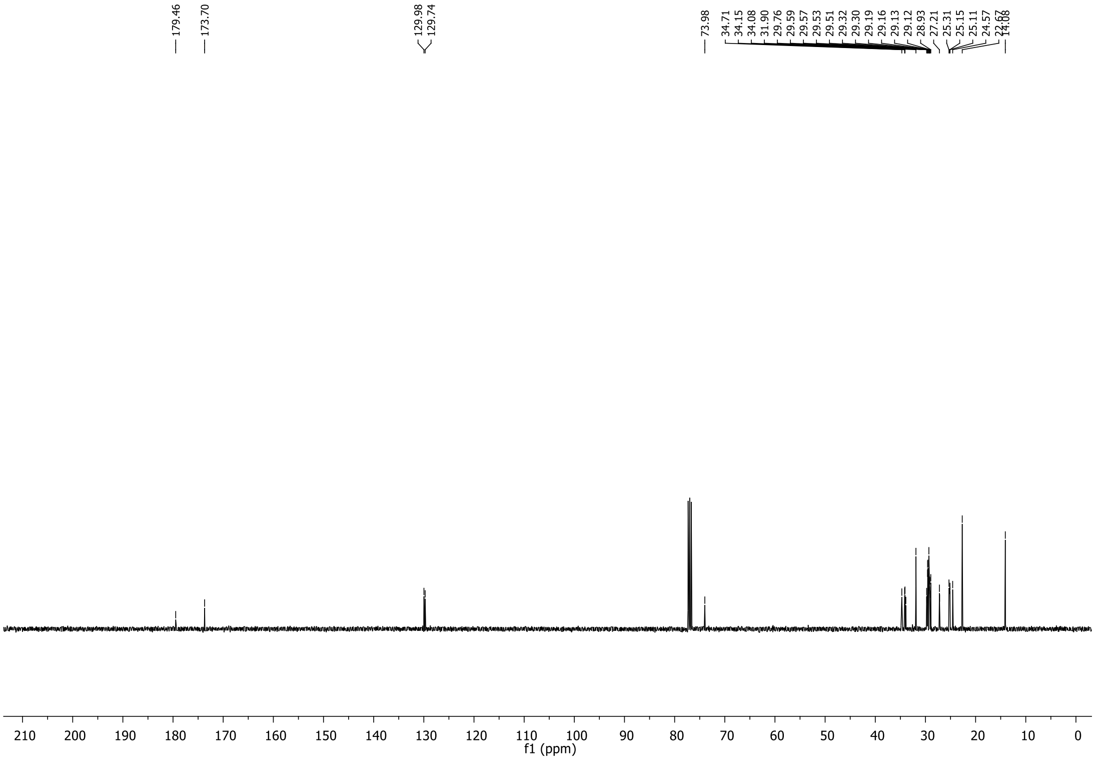

179.46
173.70
129.98
129.74
73.98
34.71
34.15
34.08
31.90
29.76
29.59
29.57
29.53
29.51
29.32
29.30
29.19
29.16
29.13
29.12
28.93
27.21
25.31
25.15
25.11
24.57
22.67
14.08
210
200
190
180
170
160
150
140
130
120
110
100
90
80
70
60
50
40
30
20
10
0
f1 (ppm)

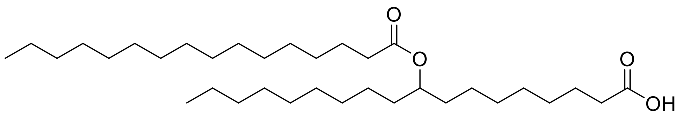
O
O
O
OH

(7m)

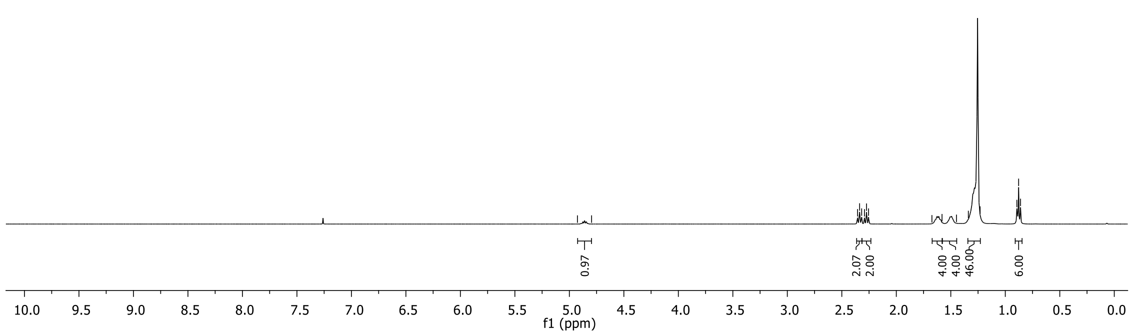
4.93
4.80
2.36
2.34
2.32
2.29
2.27
2.25
1.67
1.58
1.45
1.34
1.23
0.89
0.88
0.86
0.97
2.07
2.00
4.00
4.00
46.00
6.00
f1 (ppm)

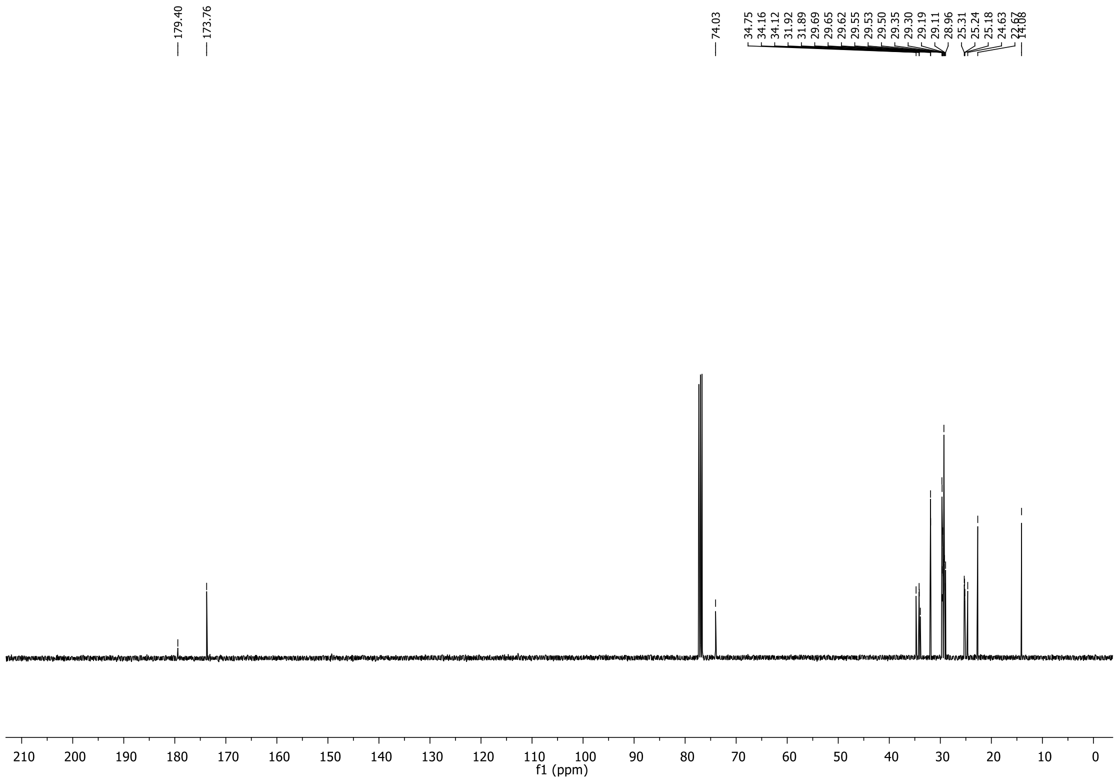
179.40
173.76
74.03
34.75
34.16
34.12
31.92
31.89
29.69
29.65
29.62
29.55
29.53
29.50
29.35
29.30
29.19
29.11
28.96
25.31
25.24
25.18
24.63
22.67
14.08
f1 (ppm)

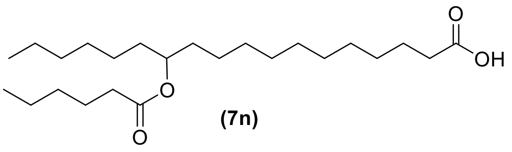

(7n)

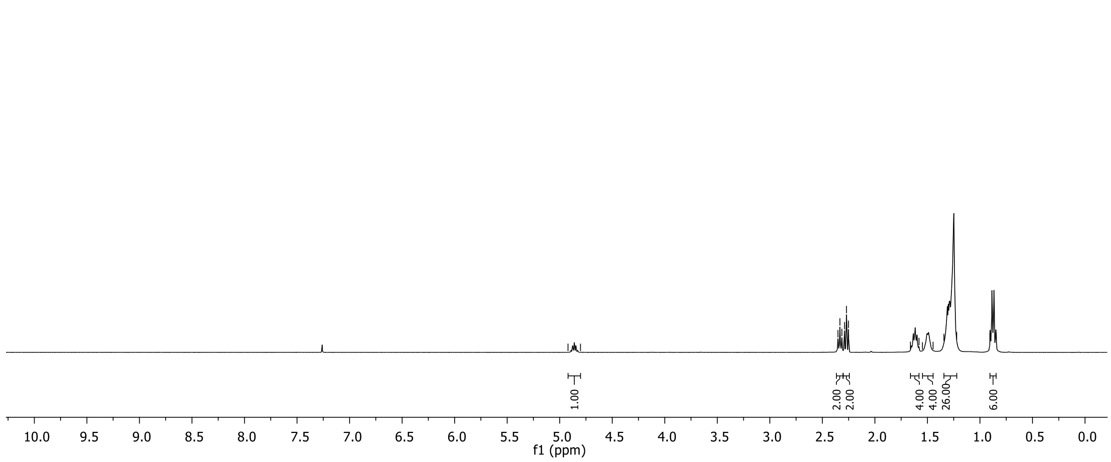
4.92
4.80
2.35
2.33
2.31
2.29
2.27
2.25
1.66
1.58
1.55
1.45
1.34
1.22
0.91
0.85
1.00
2.00
2.00
4.00
4.00
26.00
6.00
10.0 9.5 9.0 8.5 8.0 7.5 7.0 6.5 6.0 5.5 5.0 4.5 4.0 3.5 3.0 2.5 2.0 1.5 1.0 0.5 0.0
f1 (ppm)

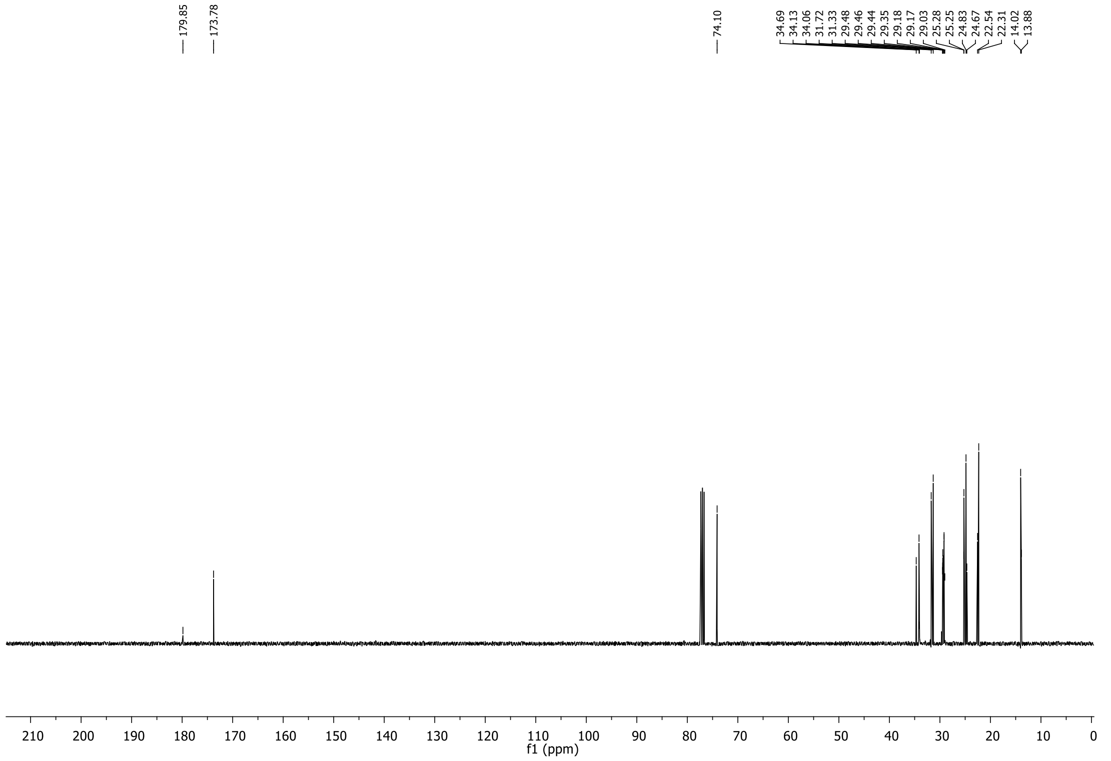
179.85
173.78
74.10
34.69
34.13
34.06
31.72
31.33
29.48
29.46
29.44
29.35
29.18
29.17
29.03
25.28
25.25
24.83
24.67
22.54
22.31
14.02
13.88
210 200 190 180 170 160 150 140 130 120 110 100 90 80 70 60 50 40 30 20 10 0
f1 (ppm)

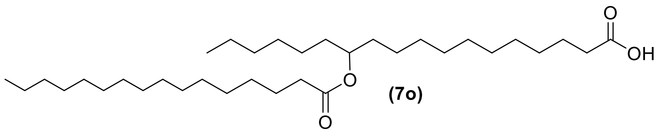
O
OH
O
O

(7o)

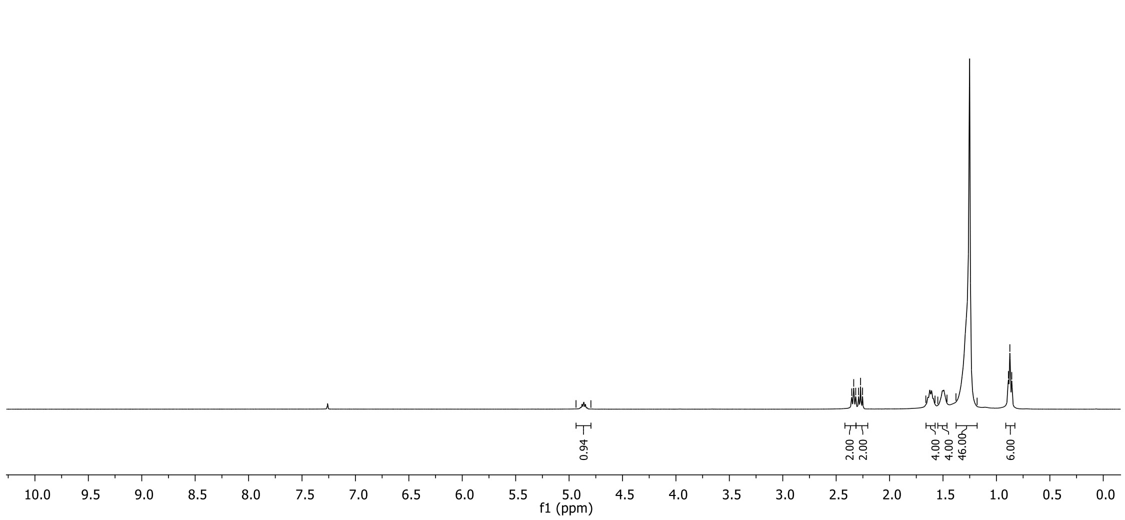
4.94
4.80
2.35
2.34
2.32
2.29
2.27
2.25
1.66
1.57
1.55
1.46
1.38
1.18
0.89
0.87
0.86
0.94
2.00
2.00
4.00
4.00
46.00
6.00
10.0 9.5 9.0 8.5 8.0 7.5 7.0 6.5 6.0 5.5 5.0 4.5 4.0 3.5 3.0 2.5 2.0 1.5 1.0 0.5 0.0
f1 (ppm)

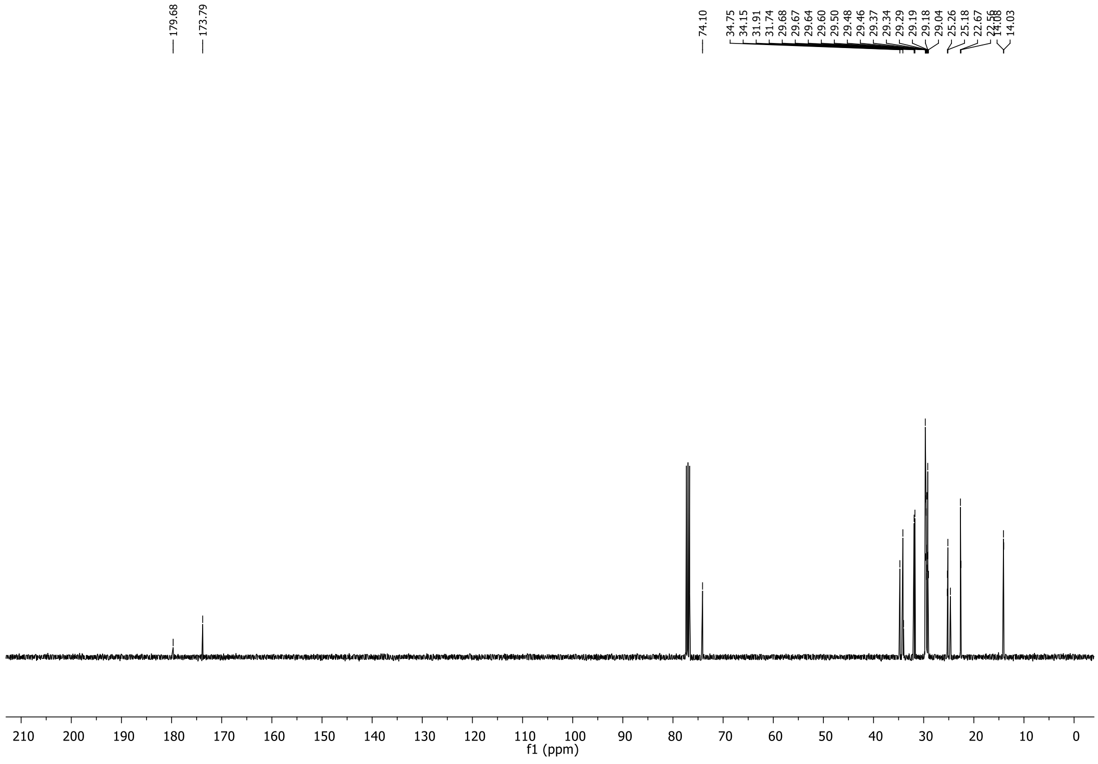
179.68
173.79
74.10
34.75
34.15
31.91
31.74
29.68
29.67
29.64
29.60
29.50
29.48
29.46
29.37
29.34
29.29
29.19
29.18
29.04
25.26
25.18
22.67
14.03
210 200 190 180 170 160 150 140 130 120 110 100 90 80 70 60 50 40 30 20 10 0
f1 (ppm)

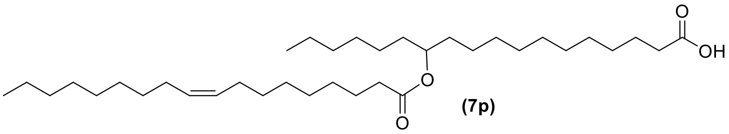
O
OH
O
O

(7p)

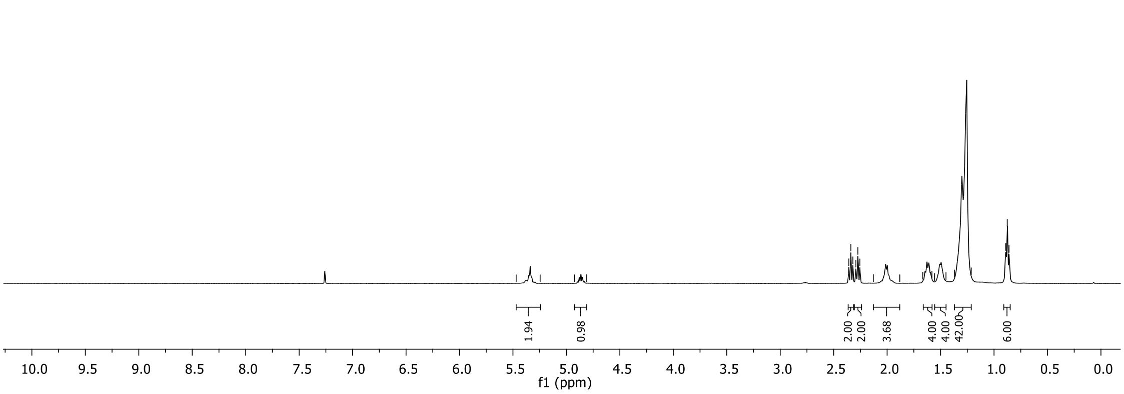
5.47
5.25
4.92
4.81
2.36
2.34
2.32
2.29
2.27
2.25
2.13
1.88
1.67
1.58
1.56
1.45
1.37
1.21
0.89
0.88
0.86
1.94
0.98
2.00
2.00
3.68
4.00
4.00
42.00
6.00
10.0 9.5 9.0 8.5 8.0 7.5 7.0 6.5 6.0 5.5 5.0 4.5 4.0 3.5 3.0 2.5 2.0 1.5 1.0 0.5 0.0
f1 (ppm)

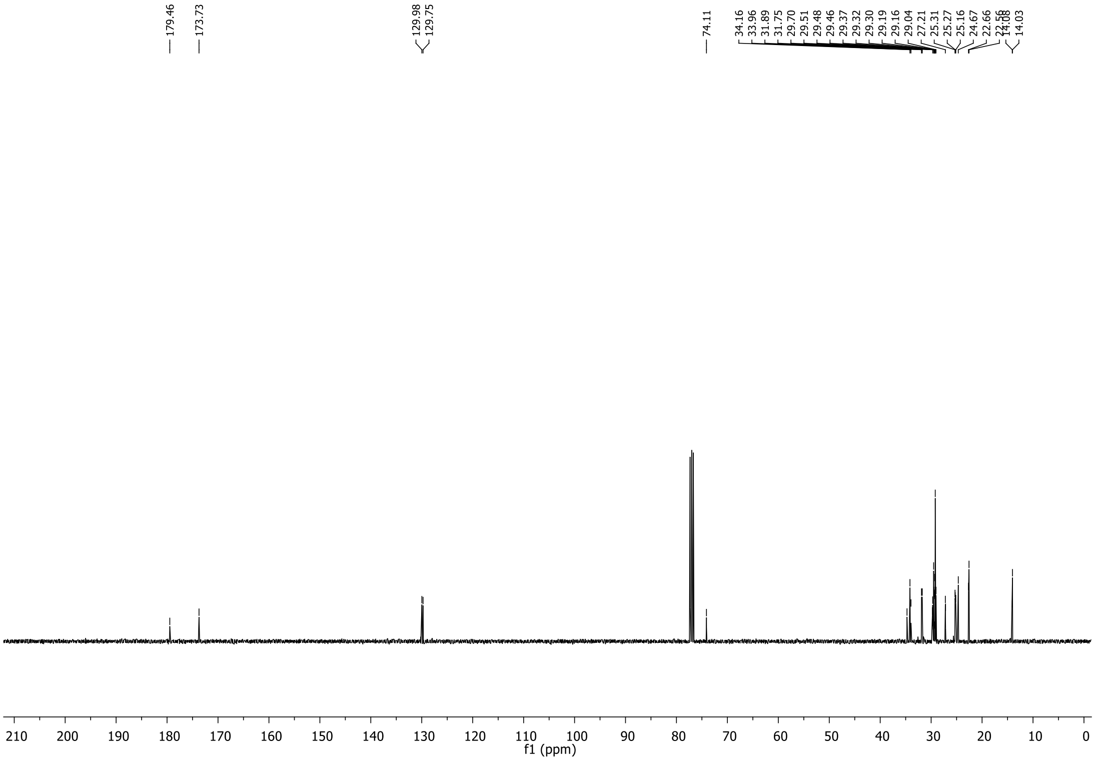
179.46
173.73
129.98
129.75
74.11
34.16
33.96
31.89
31.75
29.70
29.51
29.48
29.46
29.37
29.32
29.30
29.19
29.16
29.04
27.21
25.31
25.27
25.16
24.67
22.66
14.03
210 200 190 180 170 160 150 140 130 120 110 100 90 80 70 60 50 40 30 20 10 0
f1 (ppm)

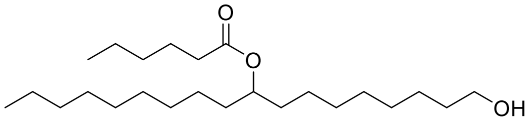
O
O
OH

(16a)

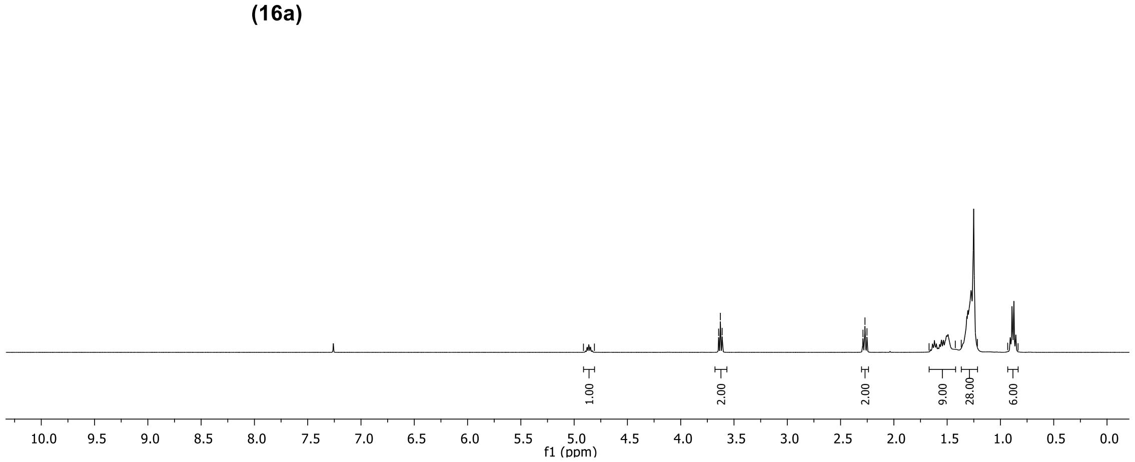
4.91
4.81
3.64
3.63
3.61
2.29
2.27
2.25
1.67
1.42
1.37
1.22
0.93
0.83
1.00
2.00
2.00
9.00
28.00
6.00
10.0 9.5 9.0 8.5 8.0 7.5 7.0 6.5 6.0 5.5 5.0 4.5 4.0 3.5 3.0 2.5 2.0 1.5 1.0 0.5 0.0
f1 (ppm)

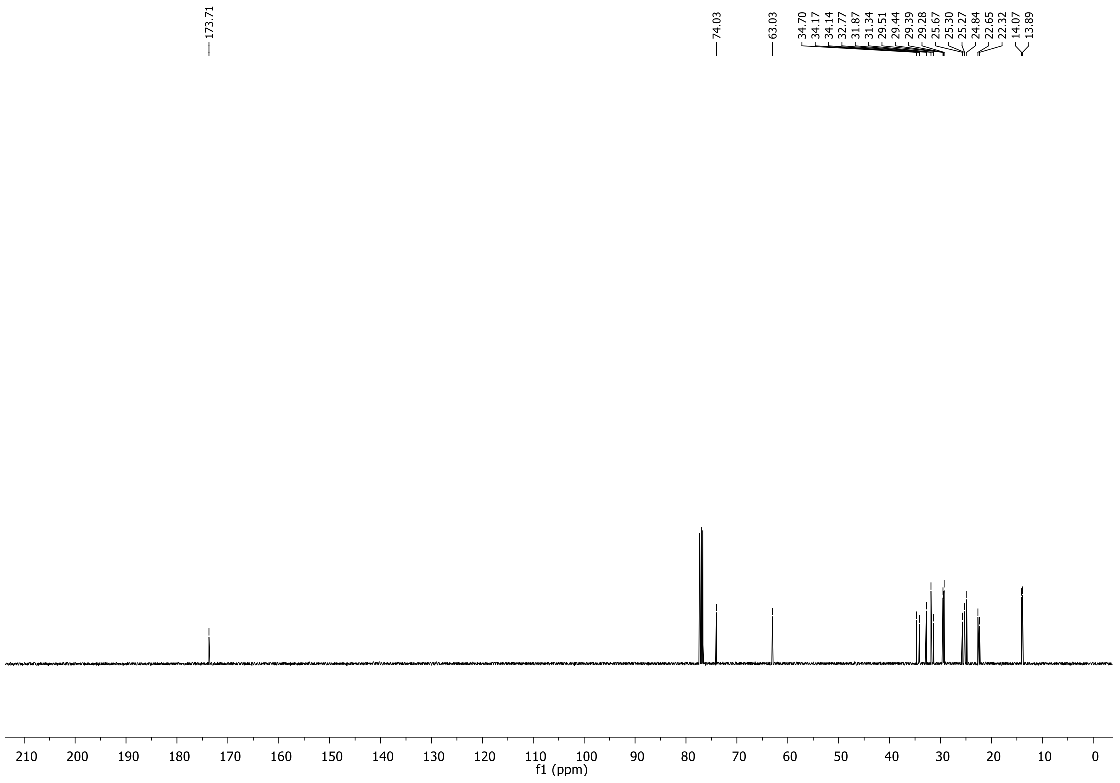
173.71
74.03
63.03
34.70
34.17
34.14
32.77
31.87
31.34
29.51
29.44
29.39
29.28
25.67
25.30
25.27
24.84
22.65
22.32
14.07
13.89
210 200 190 180 170 160 150 140 130 120 110 100 90 80 70 60 50 40 30 20 10 0
f1 (ppm)

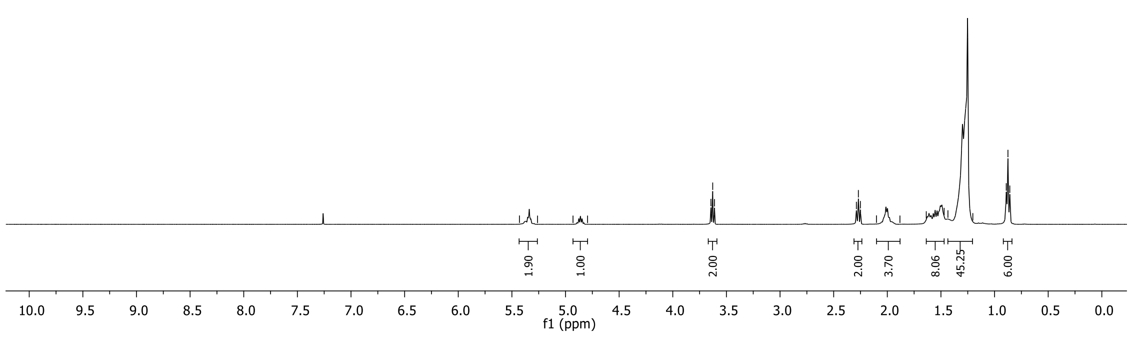

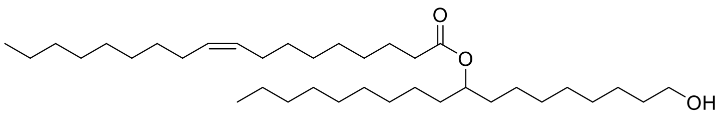
O
O
OH

(16b)

5.43
5.26
4.93
4.79
3.64
3.63
3.61
2.29
2.27
2.25
2.10
1.88
1.64
1.47
1.43
1.20
0.89
0.88
0.86
1.90
1.00
2.00
2.00
3.70
8.06
45.25
6.00
10.0 9.5 9.0 8.5 8.0 7.5 7.0 6.5 6.0 5.5 5.0 4.5 4.0 3.5 3.0 2.5 2.0 1.5 1.0 0.5 0.0
f1 (ppm)

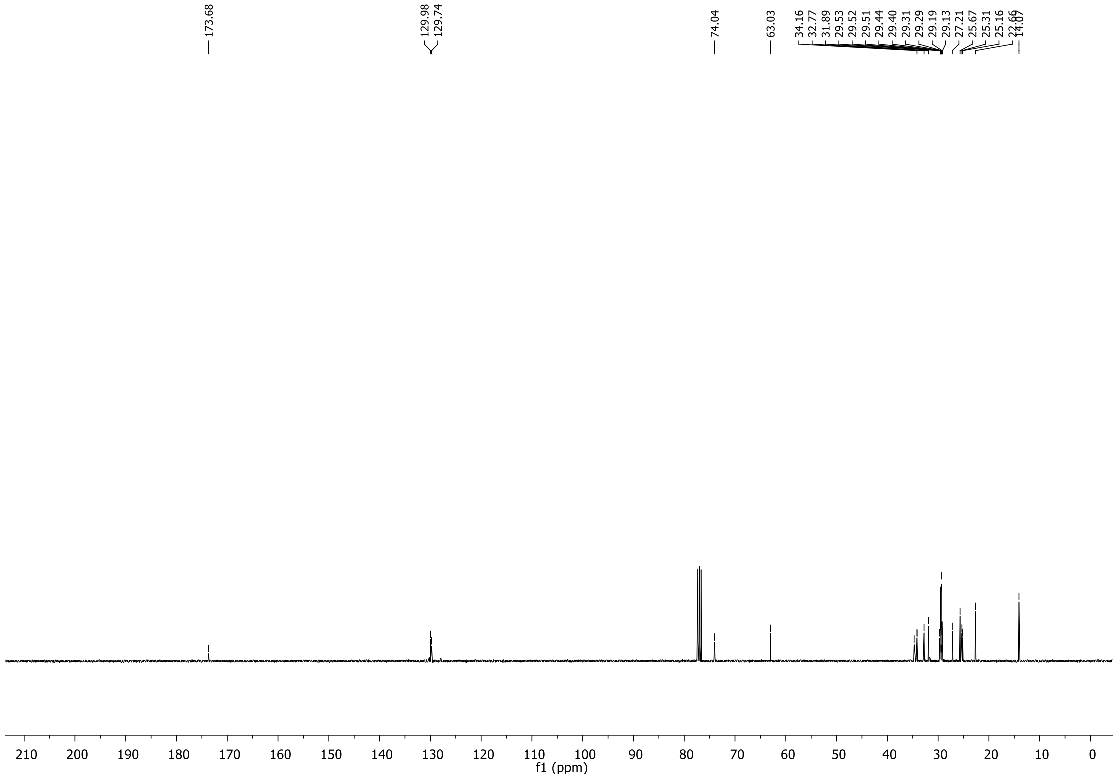
173.68
129.98
129.74
74.04
63.03
34.16
32.77
31.89
29.53
29.52
29.51
29.44
29.40
29.31
29.29
29.19
29.13
27.21
25.67
25.31
25.16
210 200 190 180 170 160 150 140 130 120 110 100 90 80 70 60 50 40 30 20 10 0
f1 (ppm)

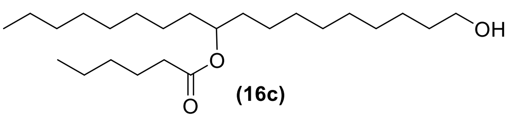
OH
O
O

(16c)

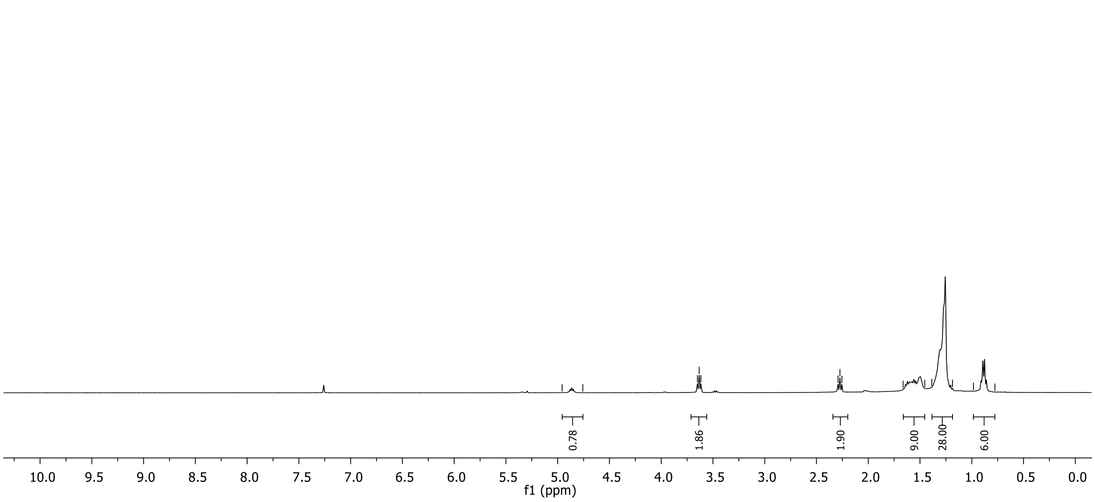
4.96
4.76
3.65
3.63
3.62
2.29
2.27
2.25
1.66
1.45
1.39
1.19
0.98
0.78
0.78
1.86
1.90
9.00
28.00
6.00
10.0 9.5 9.0 8.5 8.0 7.5 7.0 6.5 6.0 5.5 5.0 4.5 4.0 3.5 3.0 2.5 2.0 1.5 1.0 0.5 0.0
f1 (ppm)

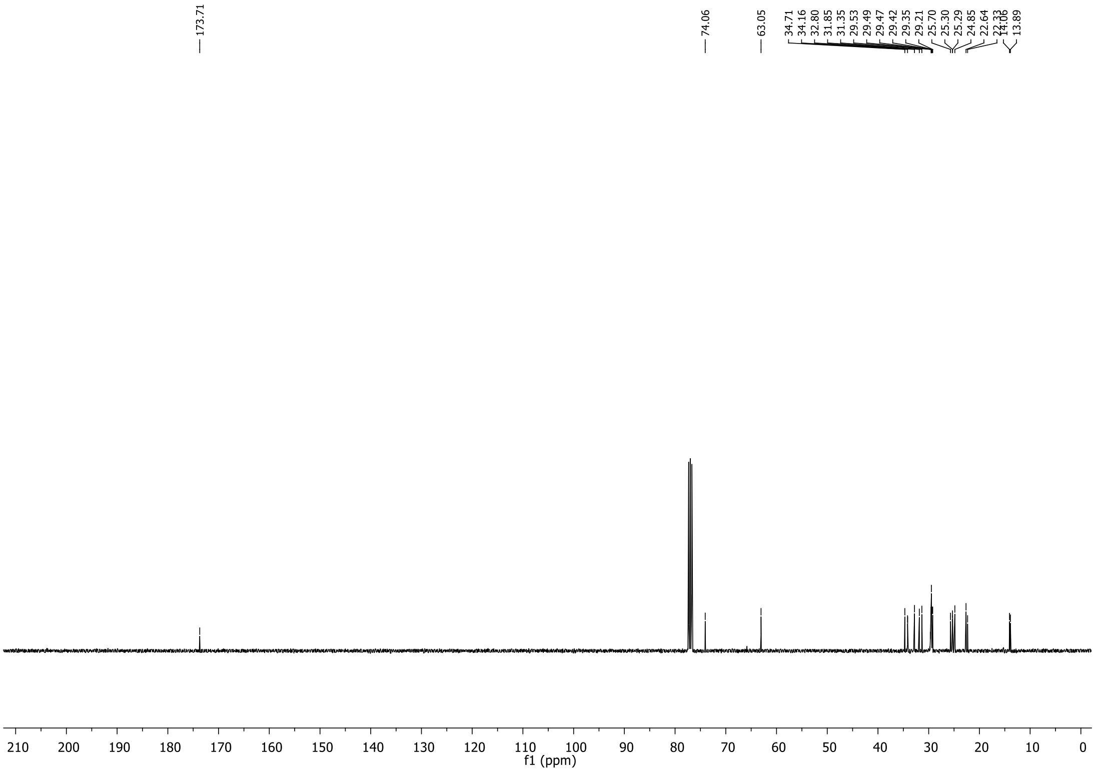
173.71
74.06
63.05
34.71
34.16
32.80
31.85
31.35
29.53
29.49
29.47
29.42
29.35
29.21
25.70
25.30
25.29
24.85
22.64
22.33
14.06
13.89
210 200 190 180 170 160 150 140 130 120 110 100 90 80 70 60 50 40 30 20 10 0
f1 (ppm)

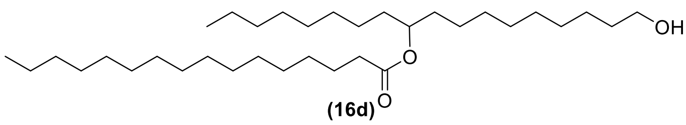
OH
O
O

(16d)

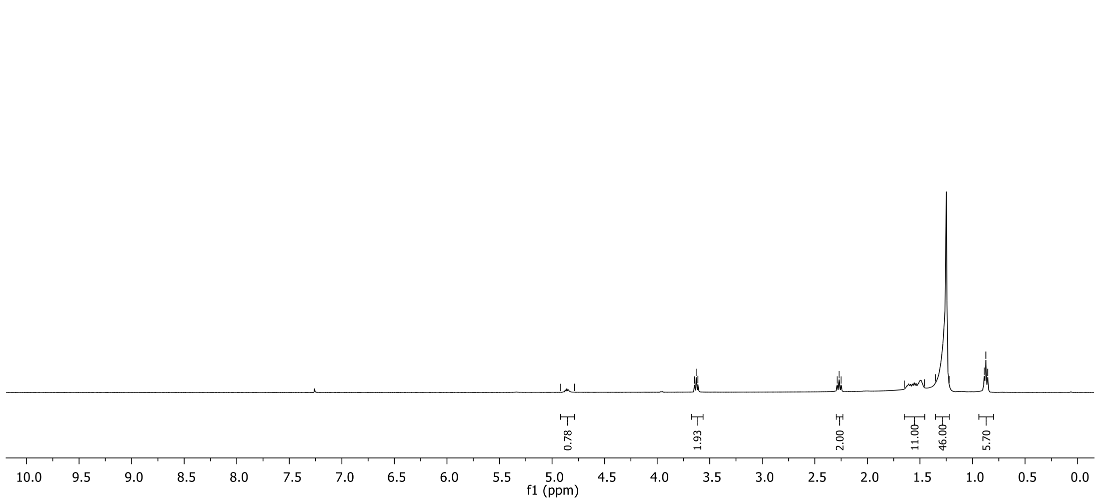
4.92
4.79
3.65
3.63
3.61
2.29
2.27
2.25
1.65
1.46
1.35
1.22
0.89
0.87
0.86
0.78
1.93
2.00
11.00
46.00
5.70
10.0 9.5 9.0 8.5 8.0 7.5 7.0 6.5 6.0 5.5 5.0 4.5 4.0 3.5 3.0 2.5 2.0 1.5 1.0 0.5 0.0
f1 (ppm)

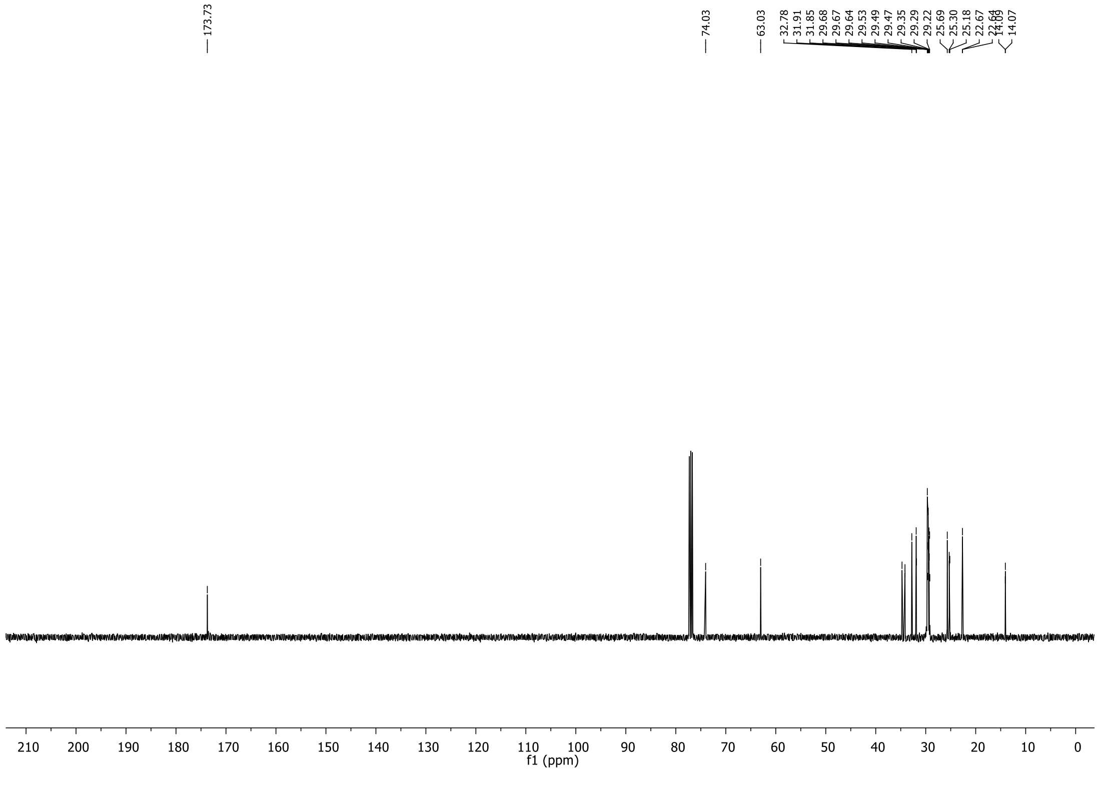
173.73
74.03
63.03
32.78
31.91
31.85
29.68
29.67
29.64
29.53
29.49
29.47
29.35
29.29
29.22
25.69
25.30
25.18
22.67
14.07
210 200 190 180 170 160 150 140 130 120 110 100 90 80 70 60 50 40 30 20 10 0
f1 (ppm)

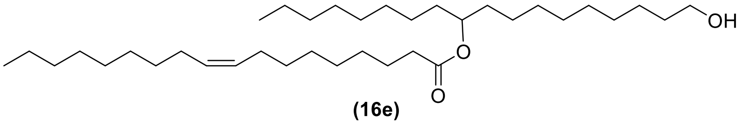

(16e)

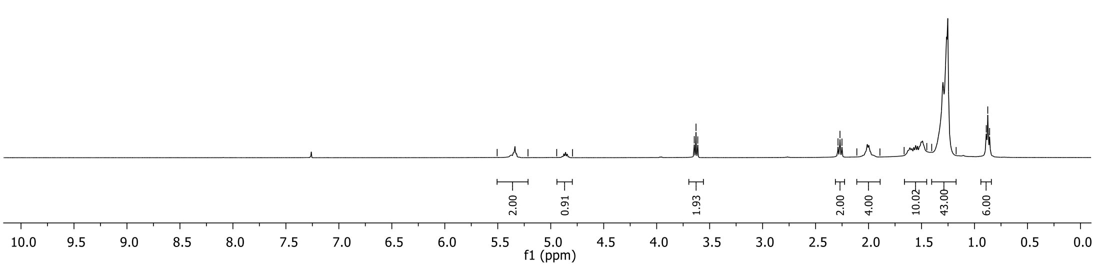

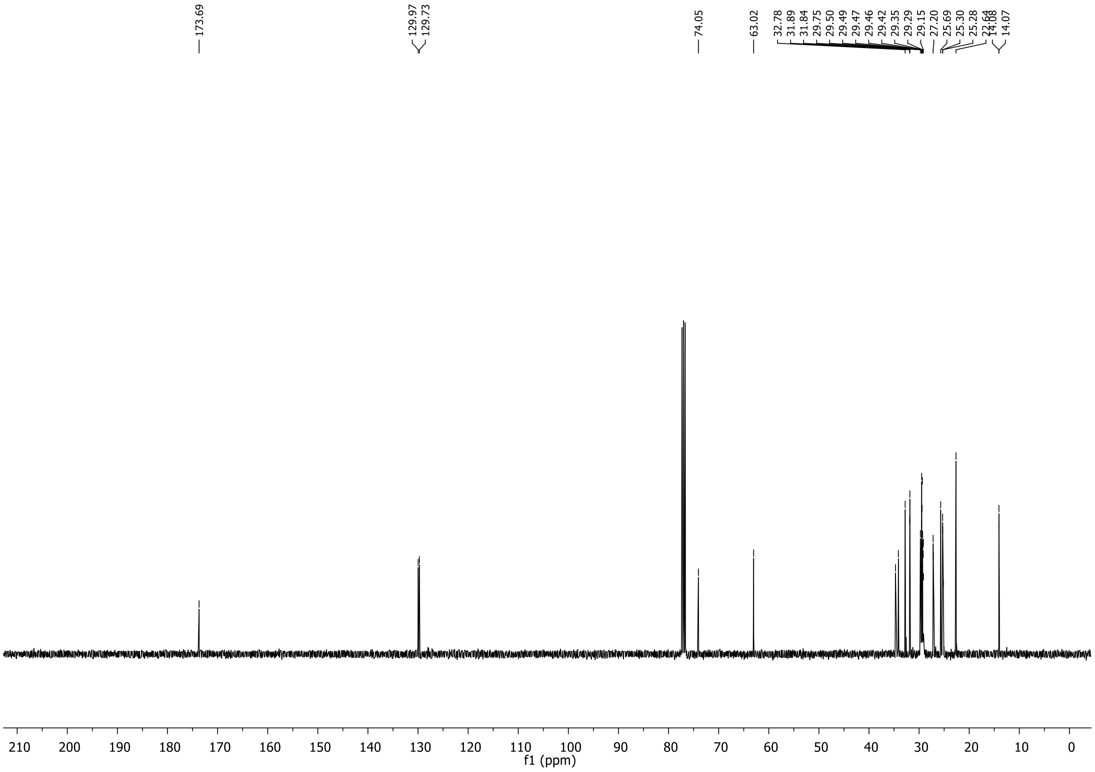

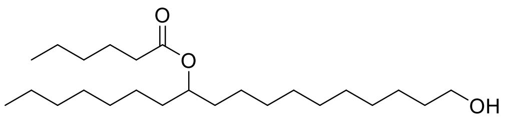
O
O
OH

(16f)

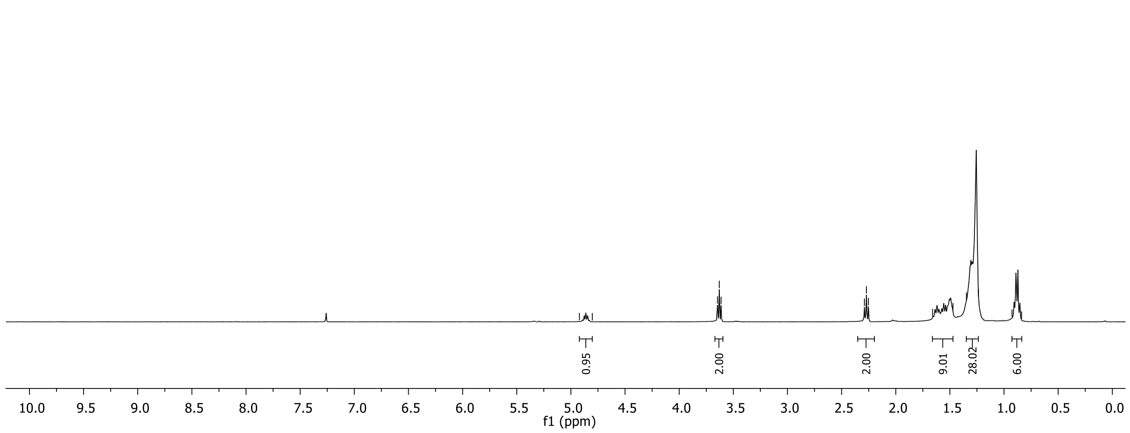
4.92
4.80
3.65
3.63
3.61
2.29
2.27
2.25
1.66
1.47
1.35
1.24
0.93
0.84
0.95
2.00
2.00
9.01
28.02
6.00
10.0 9.5 9.0 8.5 8.0 7.5 7.0 6.5 6.0 5.5 5.0 4.5 4.0 3.5 3.0 2.5 2.0 1.5 1.0 0.5 0.0
f1 (ppm)

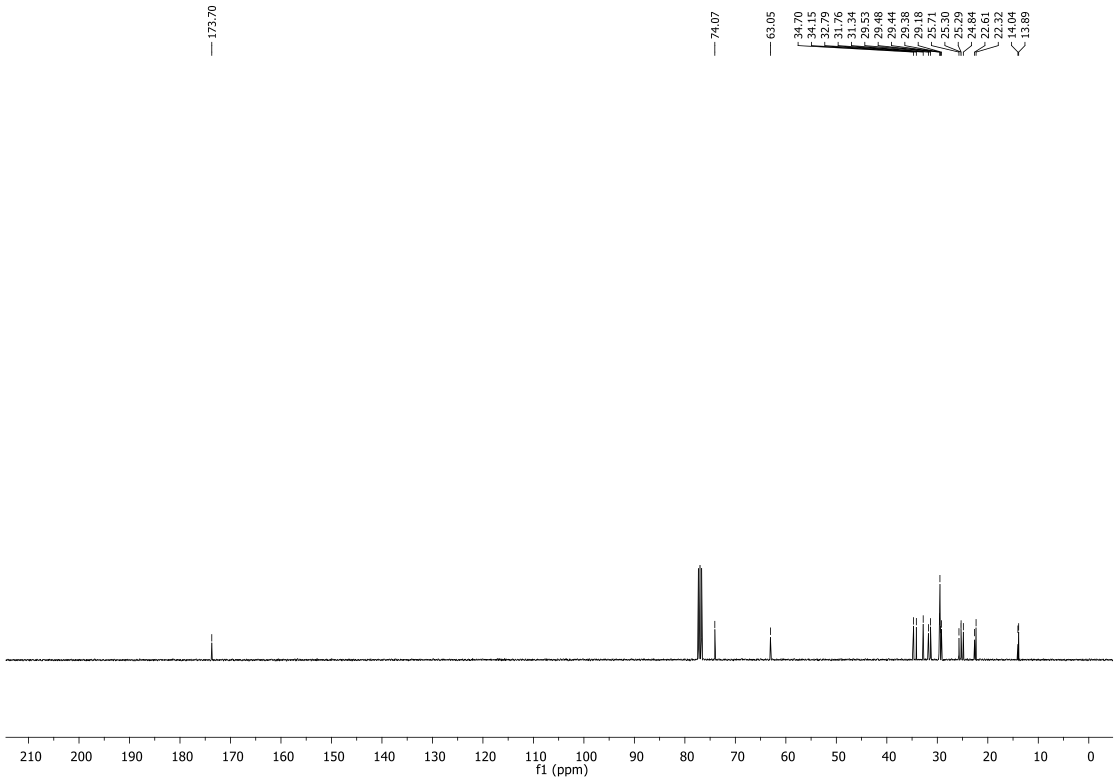
173.70
74.07
63.05
34.70
34.15
32.79
31.76
31.34
29.53
29.48
29.44
29.38
29.18
25.71
25.30
25.29
24.84
22.61
22.32
14.04
13.89
210 200 190 180 170 160 150 140 130 120 110 100 90 80 70 60 50 40 30 20 10 0
f1 (ppm)

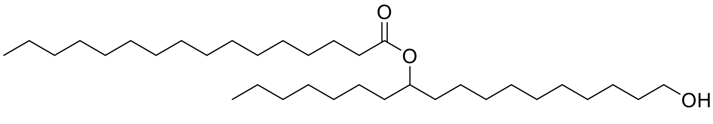
O
O
OH

(16g)

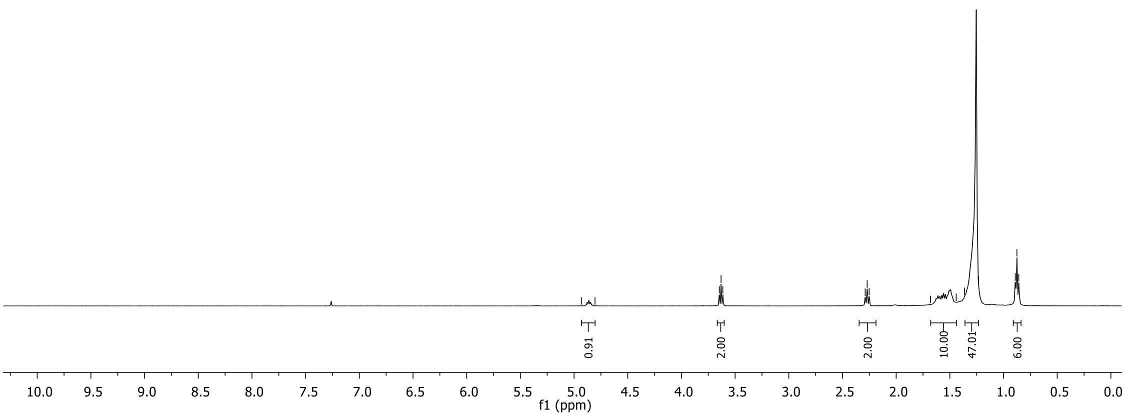
4.93
4.80
3.65
3.63
3.61
2.29
2.27
2.25
1.68
1.44
1.36
1.23
0.89
0.88
0.86
0.91
2.00
2.00
10.00
47.01
6.00
10.0 9.5 9.0 8.5 8.0 7.5 7.0 6.5 6.0 5.5 5.0 4.5 4.0 3.5 3.0 2.5 2.0 1.5 1.0 0.5 0.0
f1 (ppm)

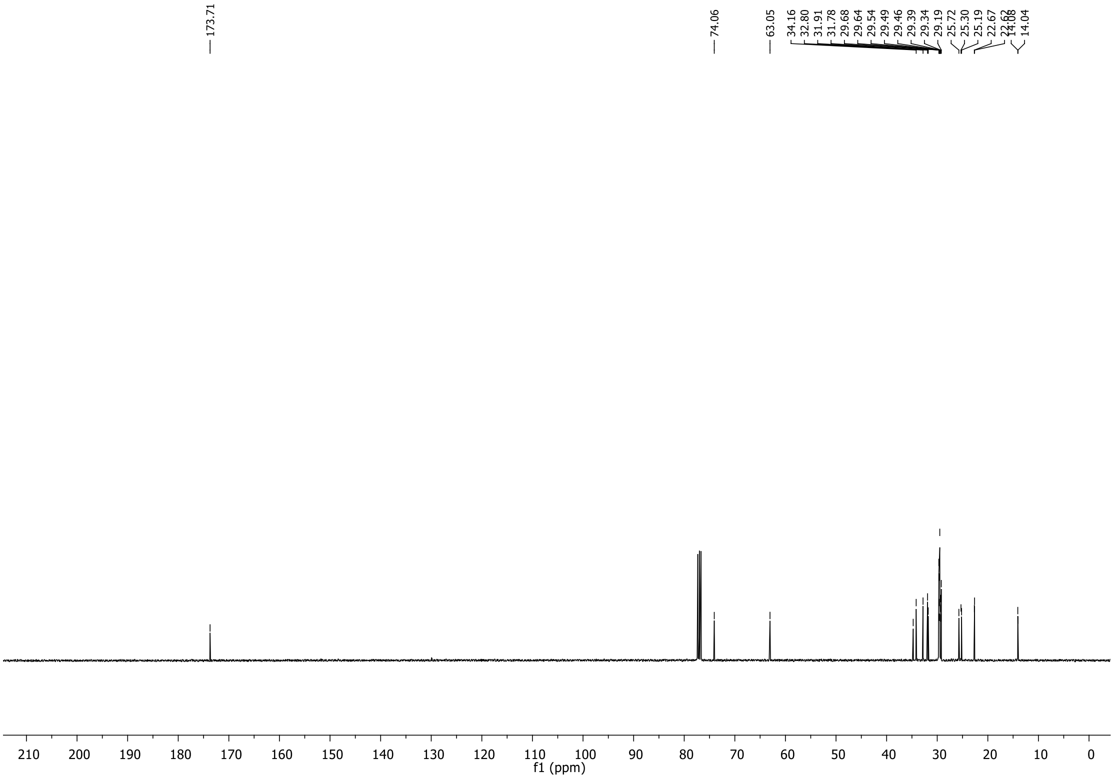
173.71
74.06
63.05
34.16
32.80
31.91
31.78
29.68
29.64
29.54
29.49
29.46
29.39
29.34
29.19
25.72
25.30
25.19
22.67
14.04
210 200 190 180 170 160 150 140 130 120 110 100 90 80 70 60 50 40 30 20 10 0
f1 (ppm)

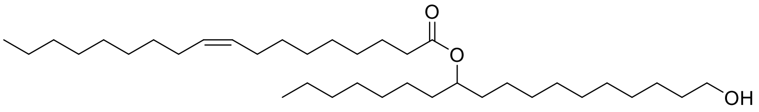
O
O
OH

(16h)

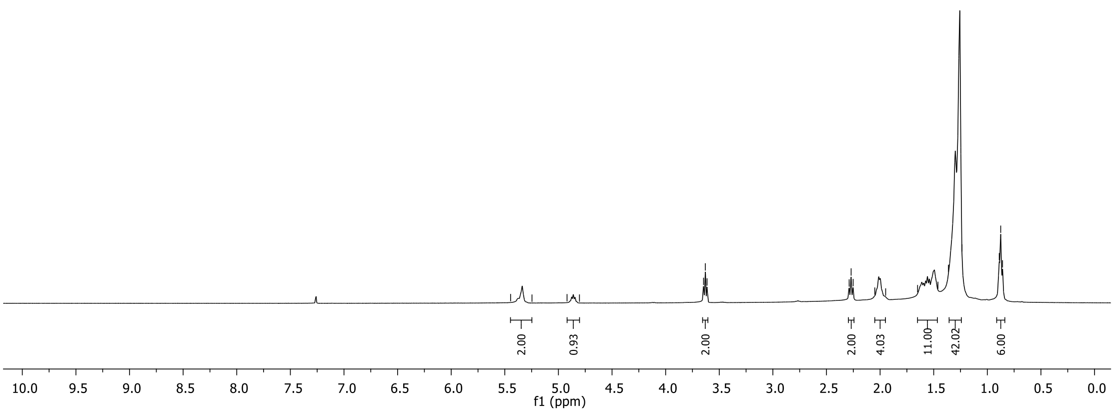
5.45
5.25
4.92
4.80
3.65
3.63
3.61
2.29
2.27
2.25
2.05
1.95
1.65
1.46
1.36
1.24
0.89
0.88
0.86
2.00
0.93
2.00
2.00
4.03
11.00
42.02
6.00
10.0 9.5 9.0 8.5 8.0 7.5 7.0 6.5 6.0 5.5 5.0 4.5 4.0 3.5 3.0 2.5 2.0 1.5 1.0 0.5 0.0
f1 (ppm)

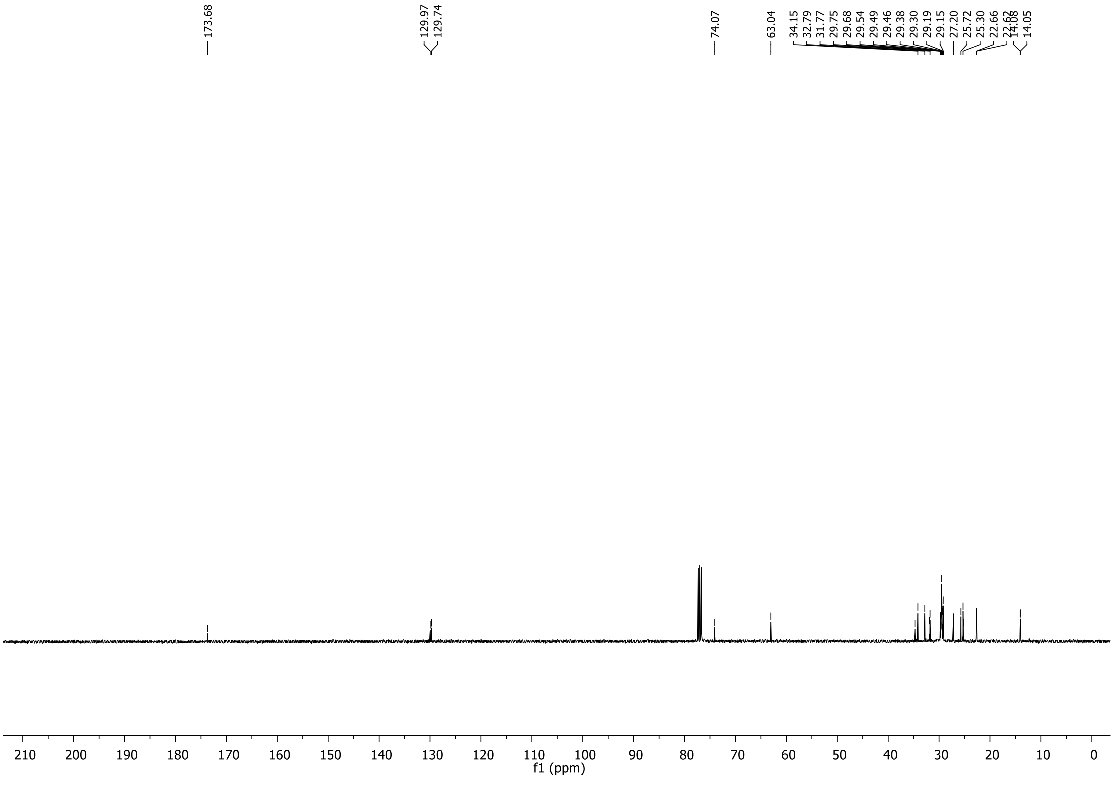
173.68
129.97
129.74
74.07
63.04
34.15
32.79
31.77
29.75
29.68
29.54
29.49
29.46
29.38
29.30
29.19
29.15
27.20
25.72
25.30
22.66
22.62
14.08
14.05
210 200 190 180 170 160 150 140 130 120 110 100 90 80 70 60 50 40 30 20 10 0
f1 (ppm)

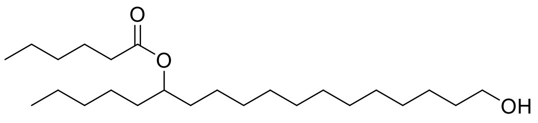
O
O
OH

(16i)

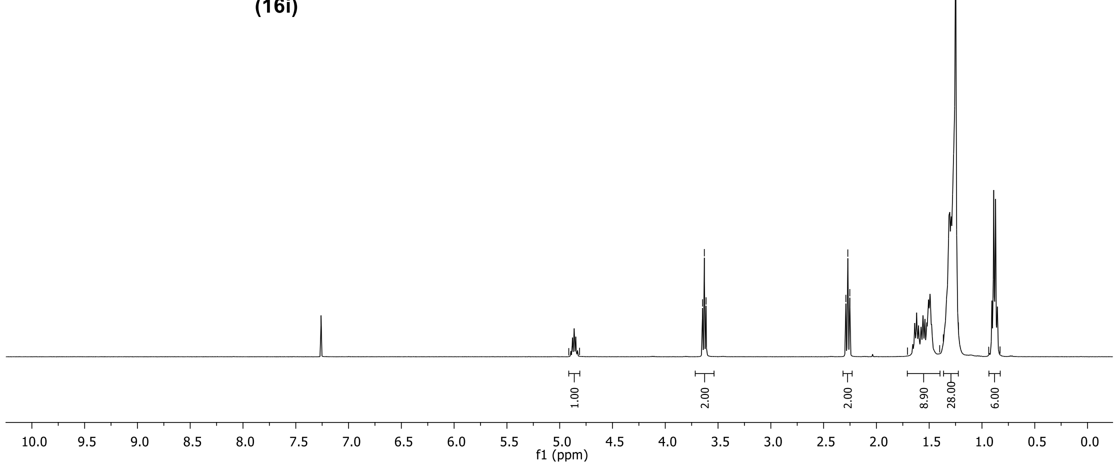
4.91
4.81
3.65
3.63
3.61
2.29
2.27
2.25
1.71
1.40
1.37
1.22
0.94
0.83
1.00
2.00
2.00
8.90
28.00
6.00
10.0 9.5 9.0 8.5 8.0 7.5 7.0 6.5 6.0 5.5 5.0 4.5 4.0 3.5 3.0 2.5 2.0 1.5 1.0 0.5 0.0
f1 (ppm)

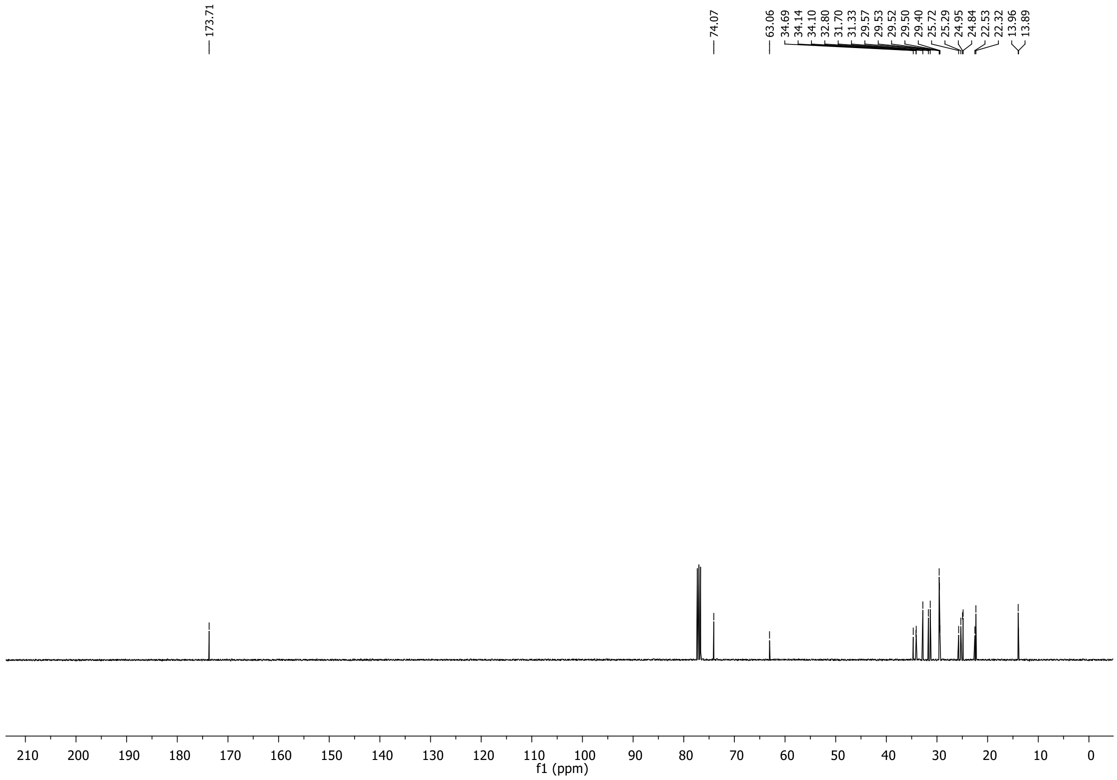
173.71
74.07
63.06
34.69
34.14
34.10
32.80
31.70
31.33
29.57
29.53
29.52
29.50
29.40
25.72
25.29
24.95
24.84
22.53
22.32
13.96
13.89
210 200 190 180 170 160 150 140 130 120 110 100 90 80 70 60 50 40 30 20 10 0
f1 (ppm)

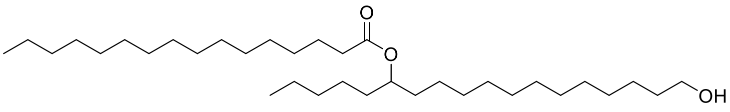

(16j)

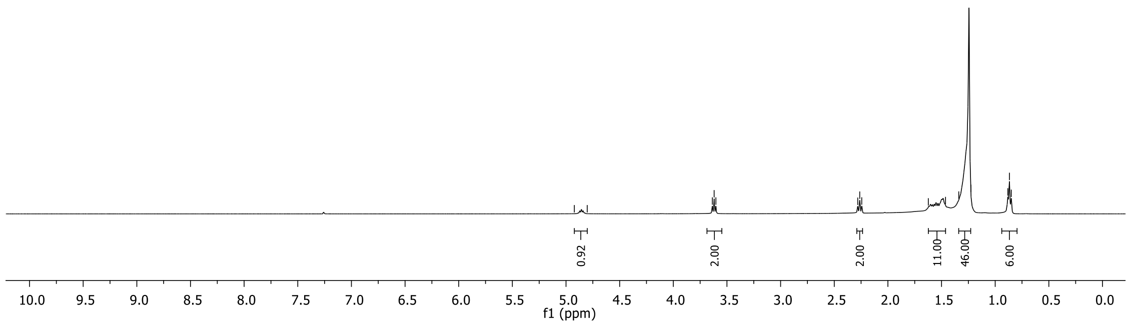

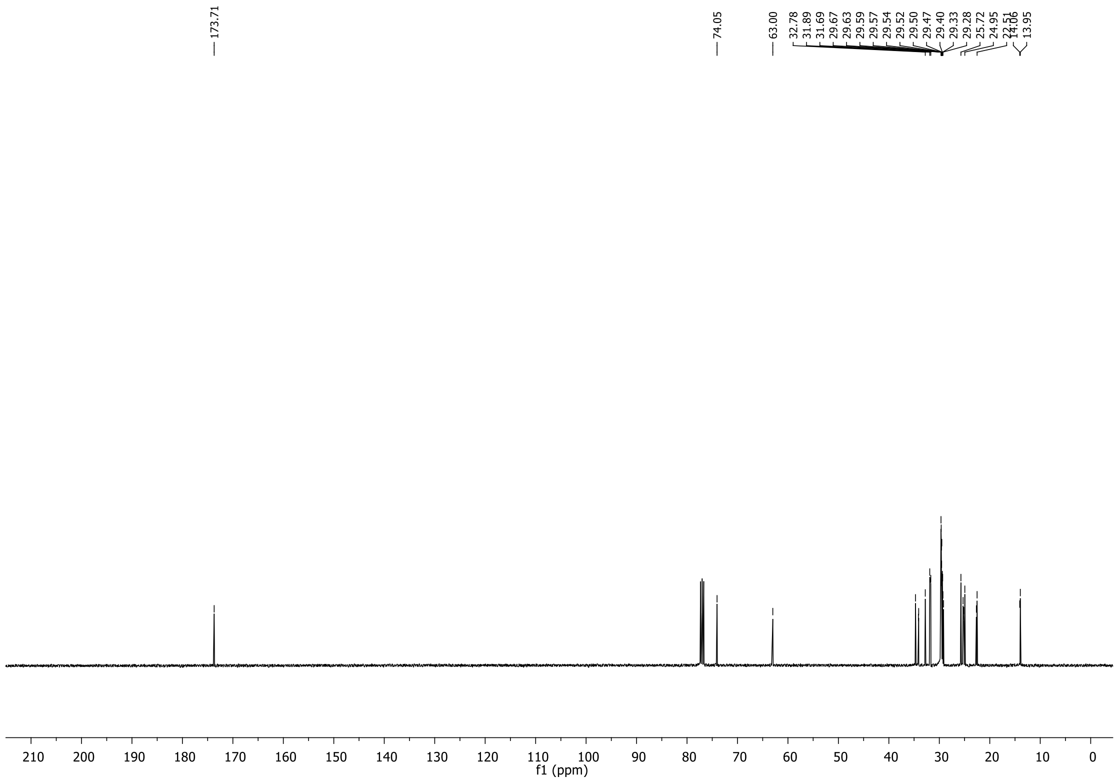

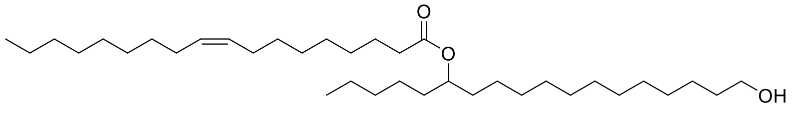
O
O
OH

(16k)

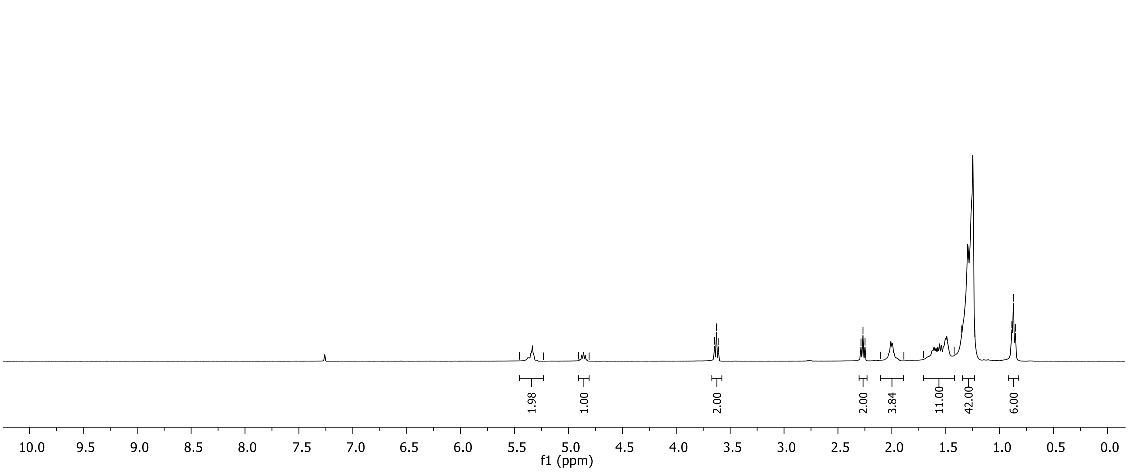
5.45
5.23
4.91
4.81
3.64
3.63
3.61
2.29
2.27
2.25
2.10
1.89
1.71
1.42
1.35
1.23
0.89
0.87
0.86
1.98
1.00
2.00
2.00
3.84
11.00
42.00
6.00
10.0 9.5 9.0 8.5 8.0 7.5 7.0 6.5 6.0 5.5 5.0 4.5 4.0 3.5 3.0 2.5 2.0 1.5 1.0 0.5 0.0
f1 (ppm)

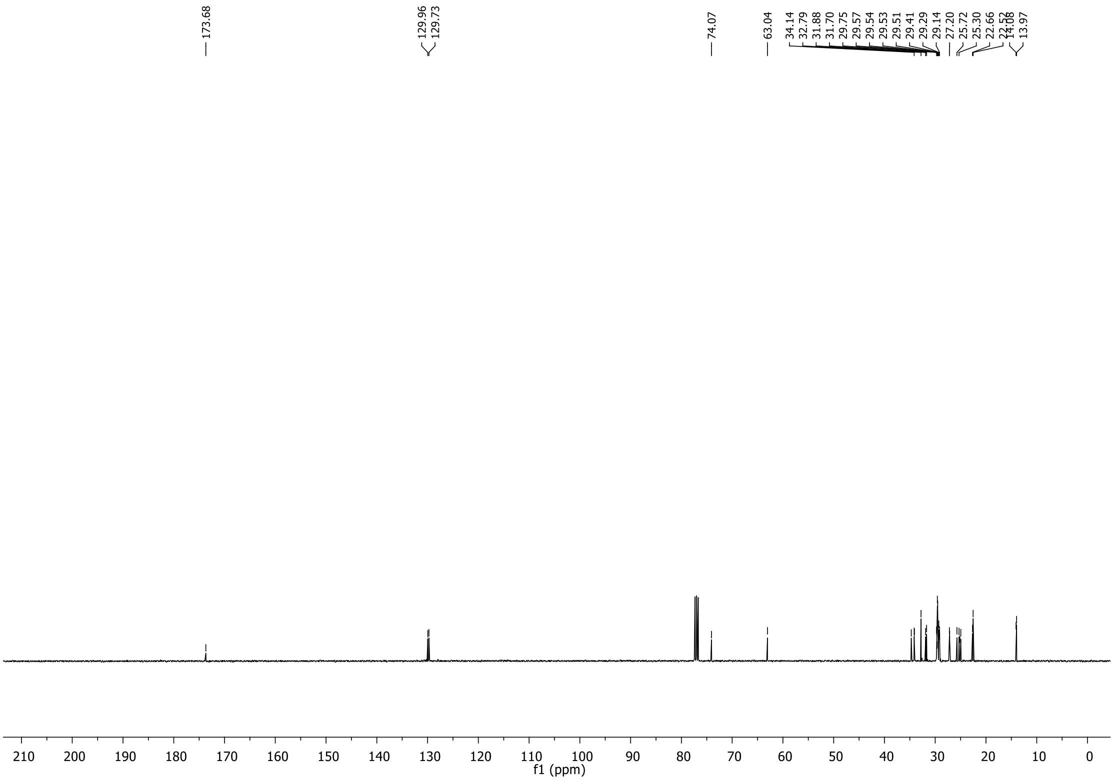
173.68
129.96
129.73
74.07
63.04
34.14
32.79
31.88
31.70
29.75
29.57
29.54
29.53
29.51
29.41
29.29
29.14
27.20
25.72
25.30
22.66
13.97
210 200 190 180 170 160 150 140 130 120 110 100 90 80 70 60 50 40 30 20 10 0
f1 (ppm)

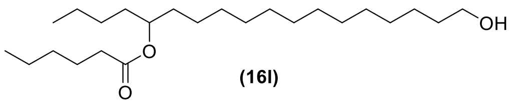
O
OH
O

(16l)

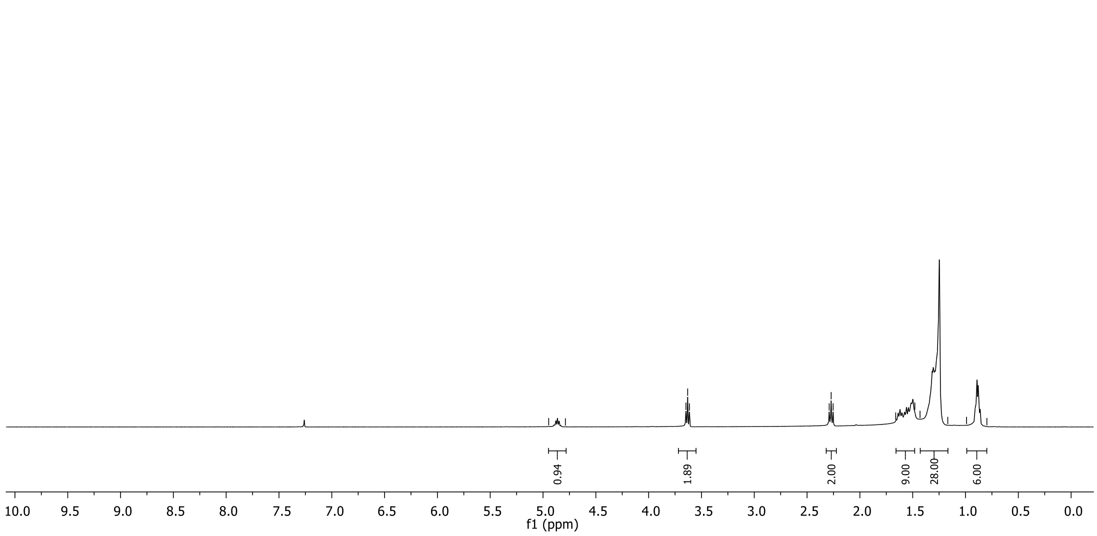
4.95
4.79
3.65
3.63
3.61
2.29
2.27
2.25
1.66
1.48
1.43
1.17
0.99
0.80
0.94
1.89
2.00
9.00
28.00
6.00
f1 (ppm)

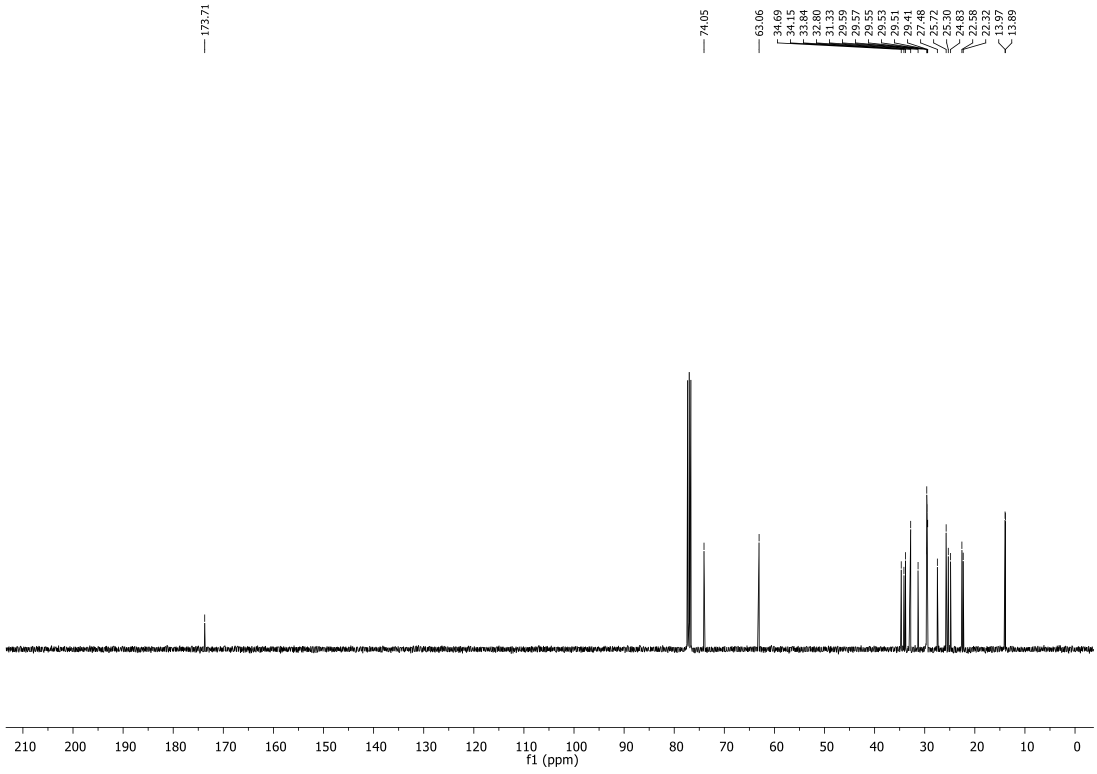
173.71
74.05
63.06
34.69
34.15
33.84
32.80
31.33
29.59
29.57
29.55
29.53
29.51
29.41
27.48
25.72
25.30
24.83
22.58
22.32
13.97
13.89
f1 (ppm)

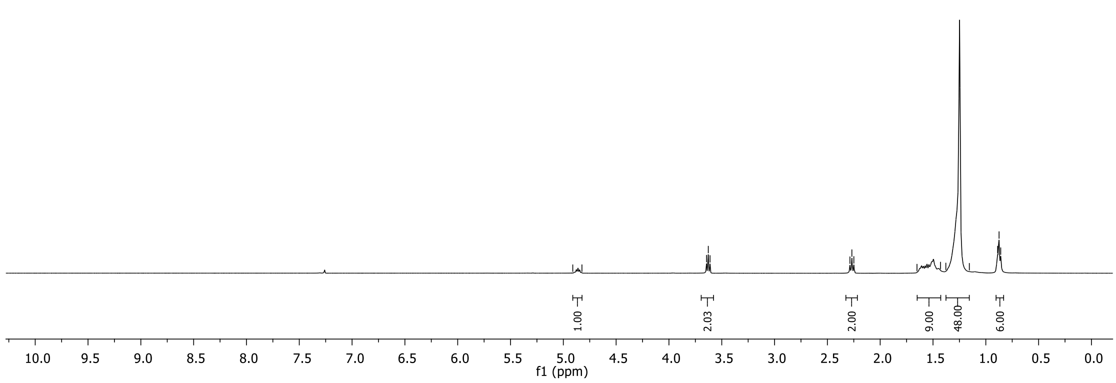
4.91
4.82
3.64
3.63
3.61
2.29
2.27
2.25
1.65
1.43
1.38
1.16
0.89
0.87
0.86
1.00
2.03
2.00
9.00
48.00
6.00
f1 (ppm)

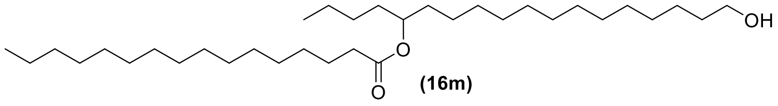
OH
O
O

(16m)

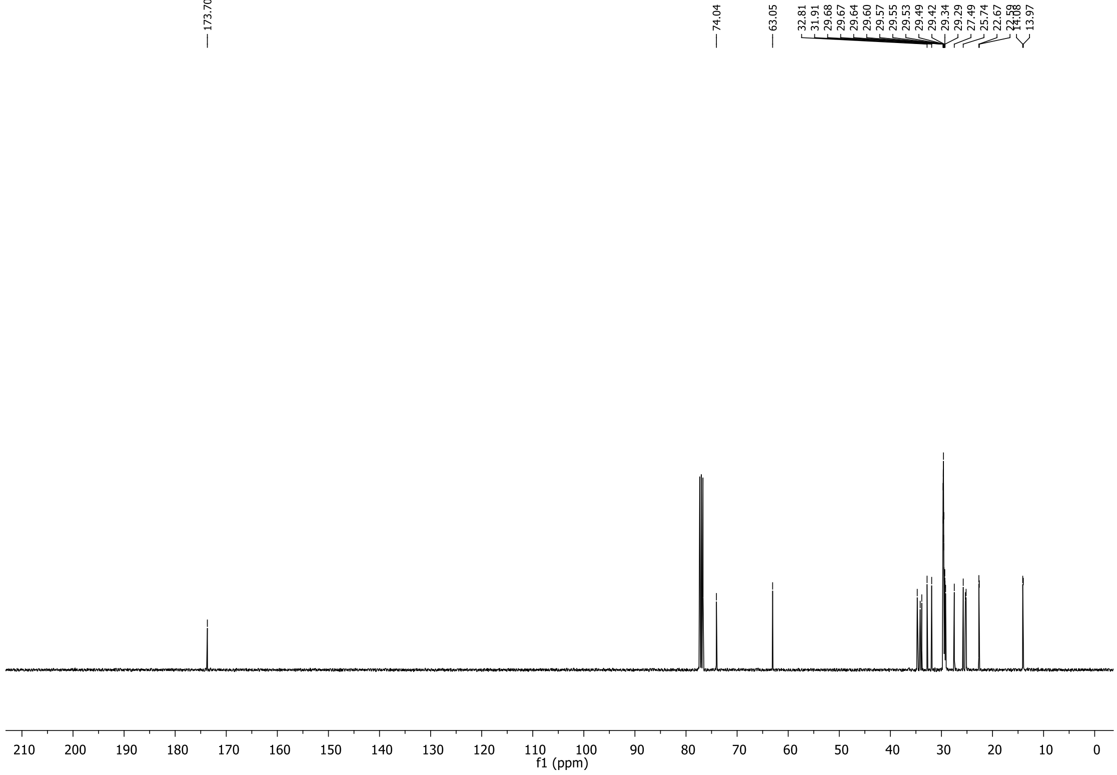
173.70
74.04
63.05
32.81
31.91
29.68
29.67
29.64
29.60
29.57
29.55
29.53
29.49
29.42
29.34
29.29
27.49
25.74
22.67
13.97
f1 (ppm)

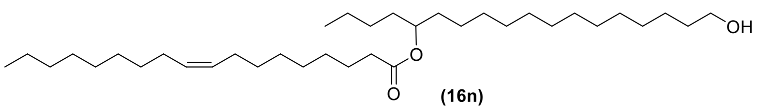
OH
O
O

(16n)

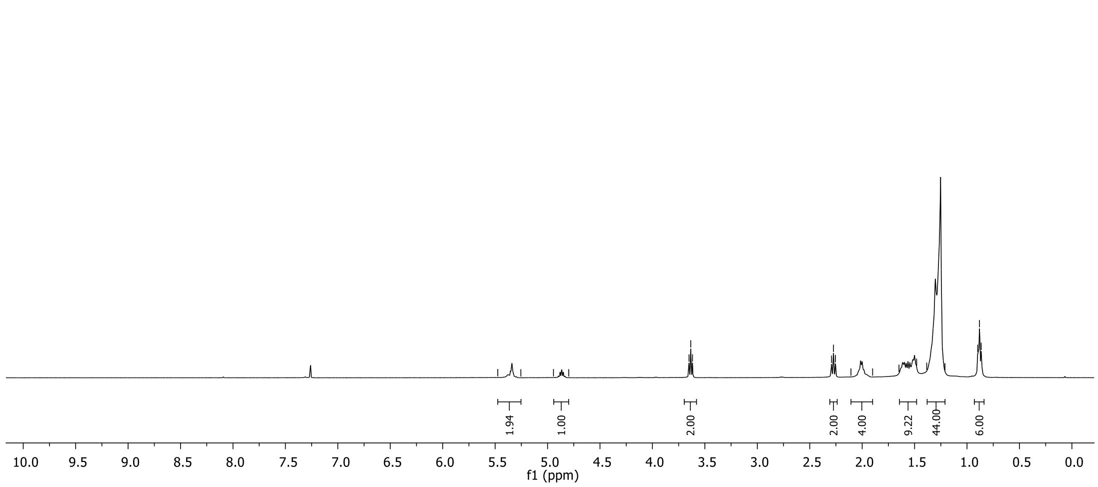
5.47
5.25
4.94
4.80
3.65
3.63
3.62
2.29
2.27
2.25
2.11
1.90
1.65
1.48
1.38
1.21
0.90
0.88
0.86
1.94
1.00
2.00
2.00
4.00
9.22
44.00
6.00
10.0 9.5 9.0 8.5 8.0 7.5 7.0 6.5 6.0 5.5 5.0 4.5 4.0 3.5 3.0 2.5 2.0 1.5 1.0 0.5 0.0
f1 (ppm)

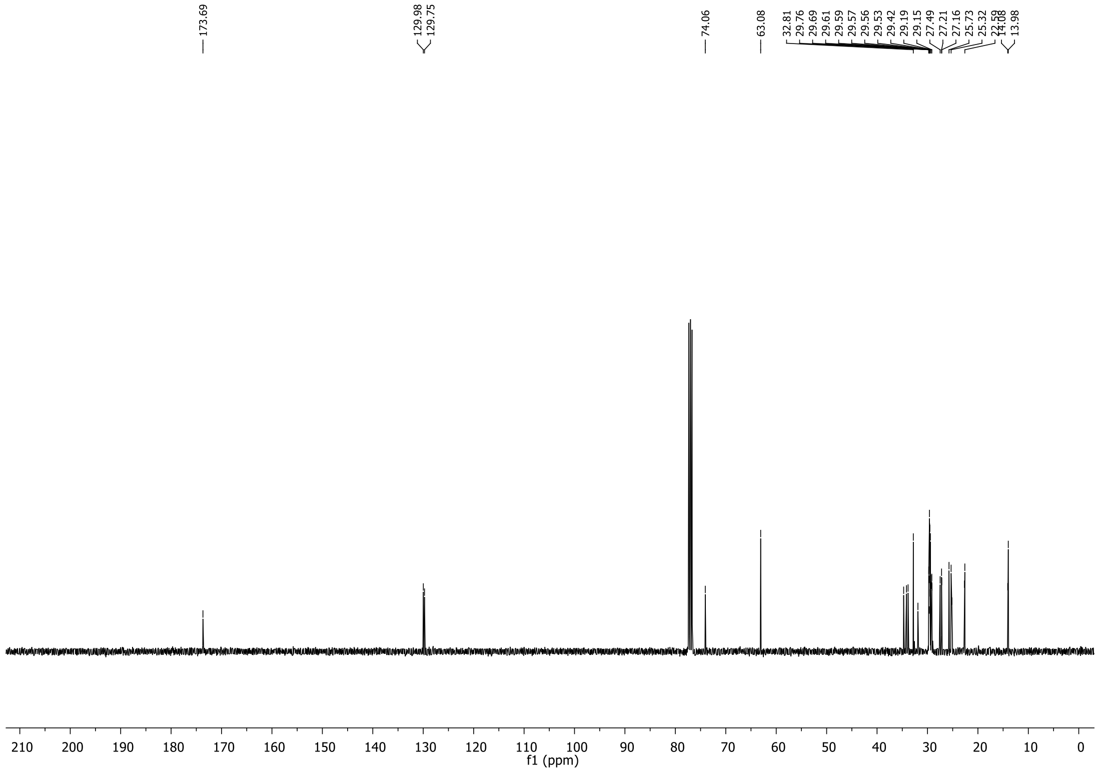
173.69
129.98
129.75
74.06
63.08
32.81
29.76
29.69
29.61
29.59
29.57
29.56
29.53
29.42
29.19
29.15
27.49
27.21
27.16
25.73
25.32
13.98
210 200 190 180 170 160 150 140 130 120 110 100 90 80 70 60 50 40 30 20 10 0
f1 (ppm)

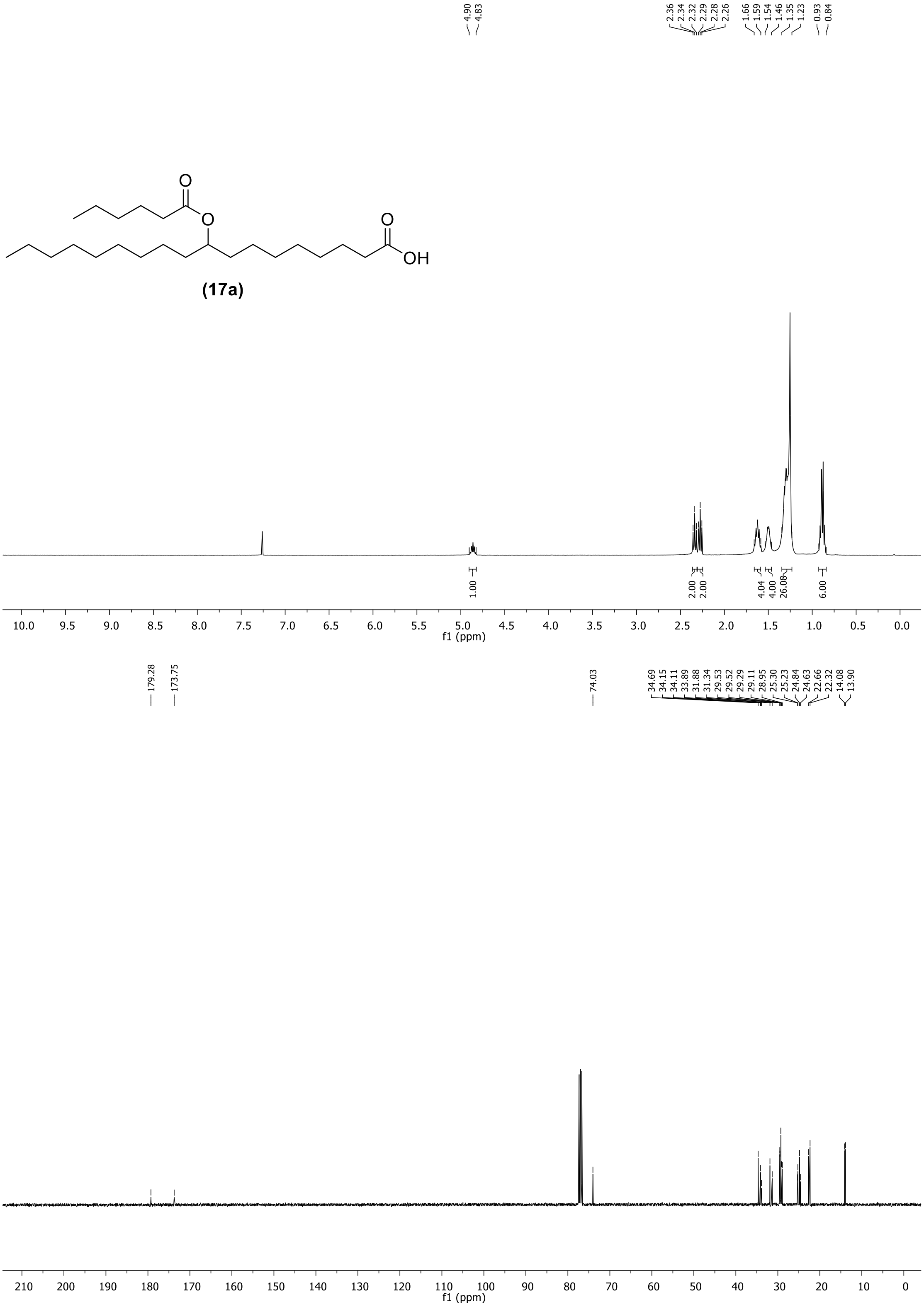
4.90
4.83
2.36
2.34
2.32
2.29
2.28
2.26
1.66
1.59
1.54
1.46
1.35
1.23
0.93
0.84
1.00
2.00
2.00
4.04
4.00
26.08
6.00
10.0 9.5 9.0 8.5 8.0 7.5 7.0 6.5 6.0 5.5 5.0 4.5 4.0 3.5 3.0 2.5 2.0 1.5 1.0 0.5 0.0
f1 (ppm)
179.28
173.75
74.03
34.69
34.15
34.11
33.89
31.88
31.34
29.53
29.52
29.29
29.11
28.95
25.30
25.23
24.84
24.63
22.66
22.32
14.08
13.90
210 200 190 180 170 160 150 140 130 120 110 100 90 80 70 60 50 40 30 20 10 0
f1 (ppm)

(17a)

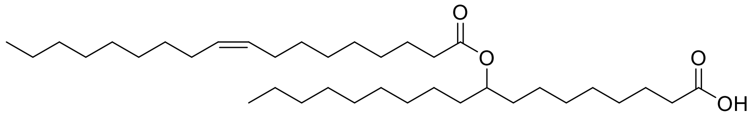
O
O
O
OH

(17b)

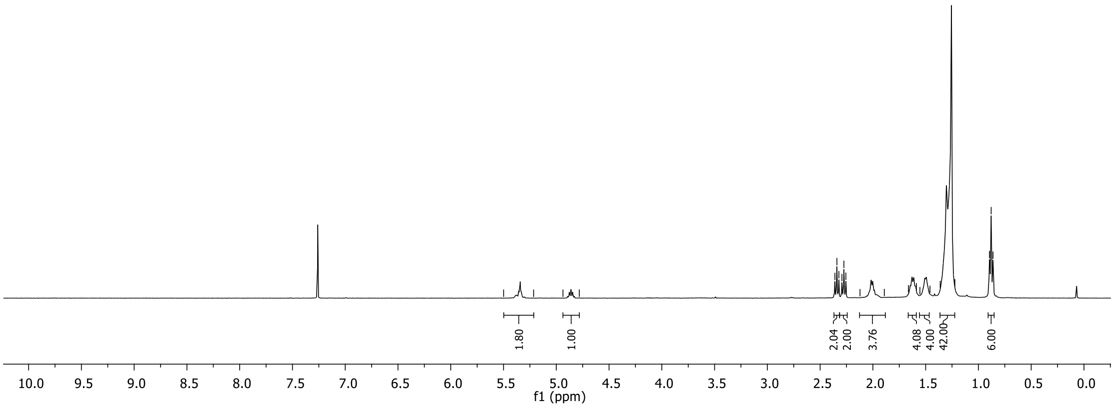
5.50
5.22
4.94
4.78
2.36
2.34
2.32
2.29
2.27
2.26
2.12
1.89
1.66
1.59
1.55
1.46
1.36
1.22
0.90
0.88
0.86
1.80
1.00
2.04
2.00
3.76
4.08
4.00
42.00
6.00
10.0 9.5 9.0 8.5 8.0 7.5 7.0 6.5 6.0 5.5 5.0 4.5 4.0 3.5 3.0 2.5 2.0 1.5 1.0 0.5 0.0
f1 (ppm)

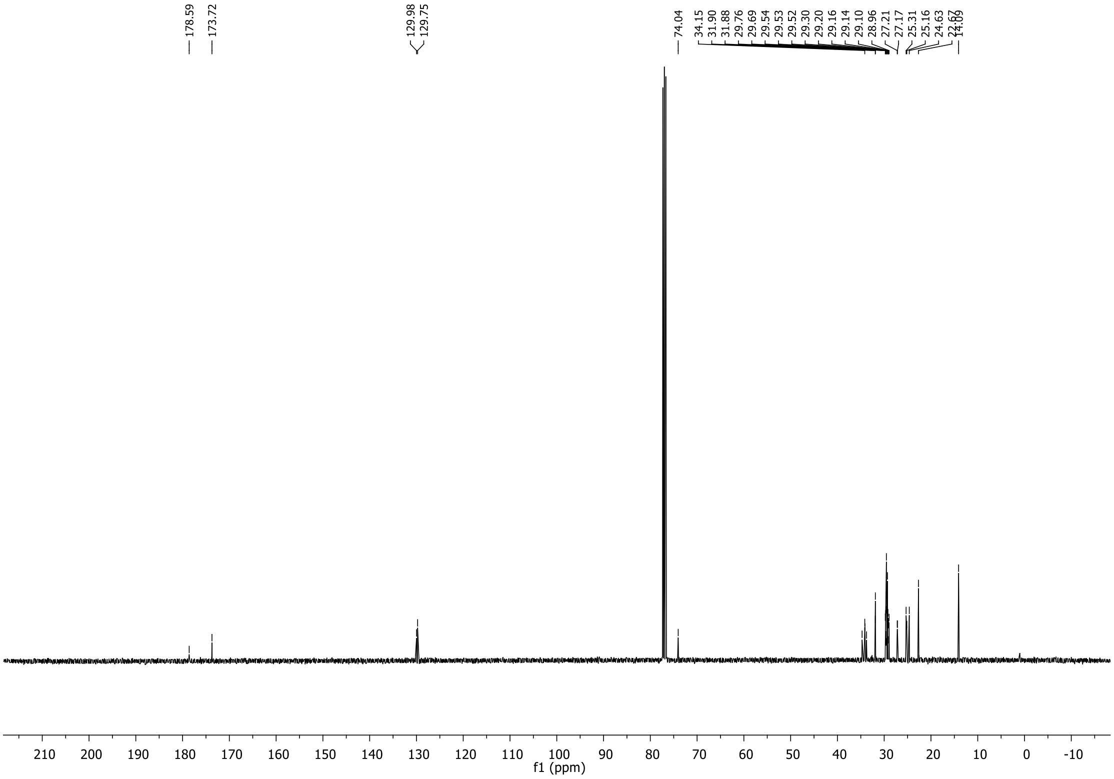
178.59
173.72
129.98
129.75
74.04
34.15
31.90
31.88
29.76
29.69
29.54
29.53
29.52
29.30
29.20
29.16
29.14
29.10
28.96
27.21
27.17
25.31
25.16
24.63
22.67
14.09
210 200 190 180 170 160 150 140 130 120 110 100 90 80 70 60 50 40 30 20 10 0 -10
f1 (ppm)

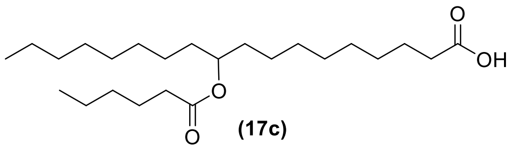
O
OH
O
O

(17c)

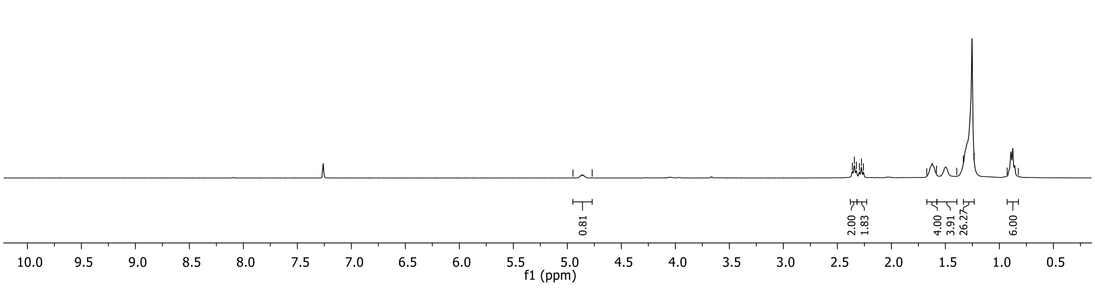
4.95
4.77
2.36
2.34
2.32
2.30
2.28
2.26
1.67
1.58
1.39
1.33
1.23
0.93
0.82
0.81
2.00
1.83
4.00
3.91
26.27
6.00
10.0 9.5 9.0 8.5 8.0 7.5 7.0 6.5 6.0 5.5 5.0 4.5 4.0 3.5 3.0 2.5 2.0 1.5 1.0 0.5
f1 (ppm)

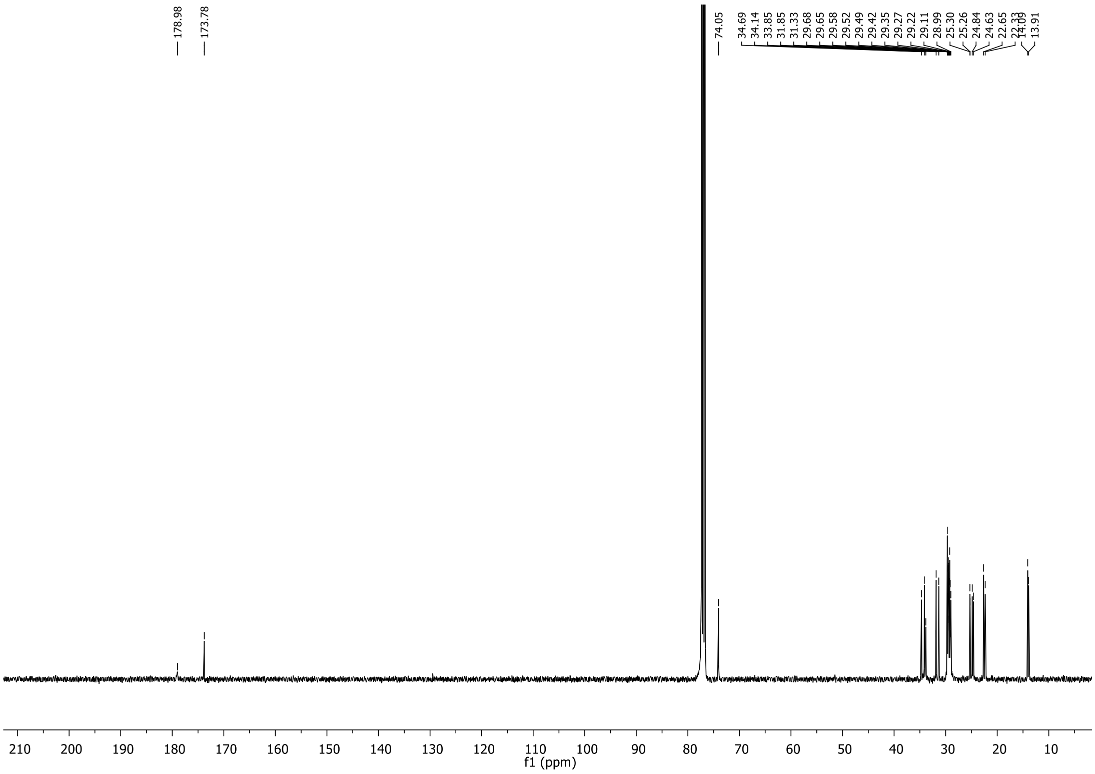
178.98
173.78
74.05
34.69
34.14
33.85
31.85
31.33
29.68
29.65
29.58
29.52
29.49
29.42
29.35
29.27
29.22
29.11
28.99
25.30
25.26
24.84
24.63
22.65
13.91
210 200 190 180 170 160 150 140 130 120 110 100 90 80 70 60 50 40 30 20 10
f1 (ppm)

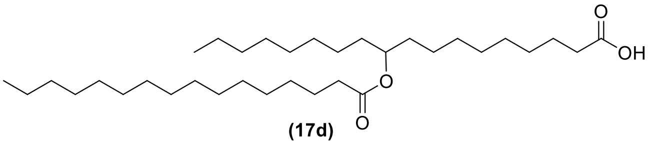
O
OH
O
O

(17d)

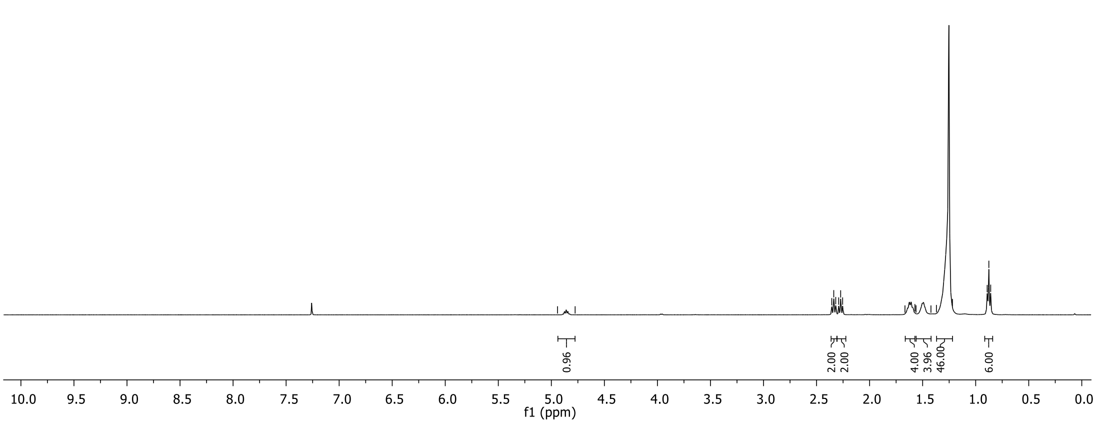
4.94
4.78
2.36
2.34
2.32
2.29
2.27
2.25
1.67
1.57
1.56
1.42
1.37
1.22
0.89
0.88
0.86
0.96
2.00
2.00
4.00
3.96
46.00
6.00
f1 (ppm)

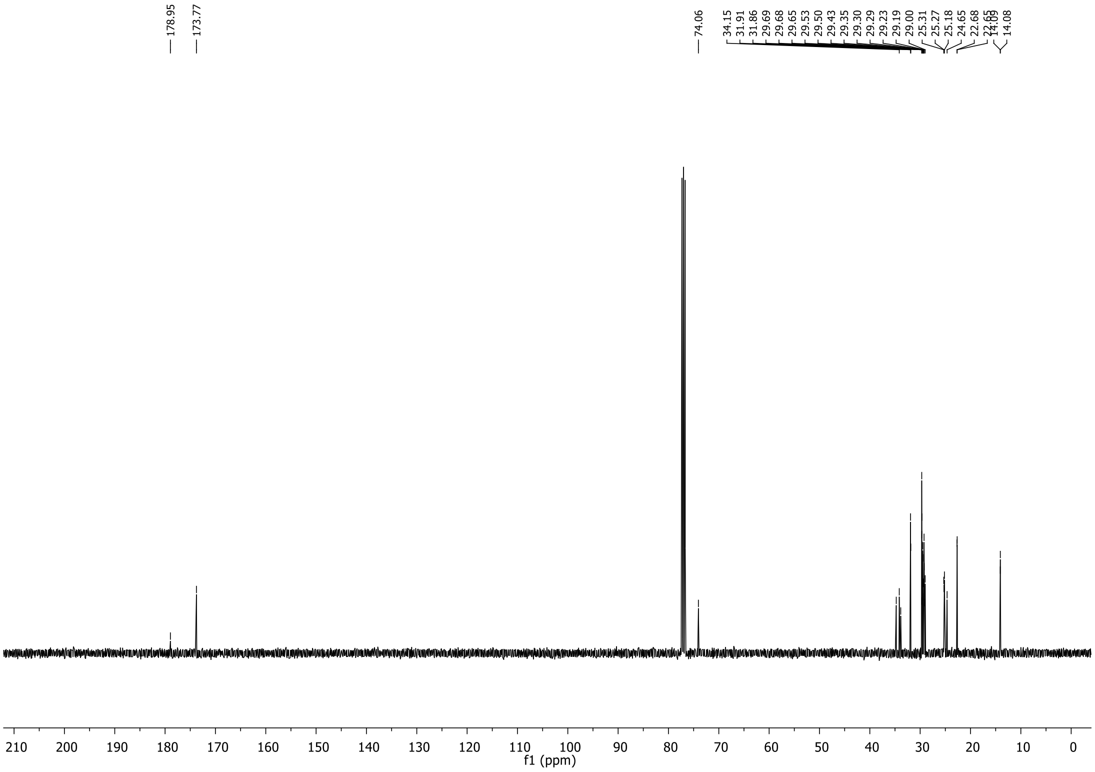
178.95
173.77
74.06
34.15
31.91
31.86
29.69
29.68
29.65
29.53
29.50
29.43
29.35
29.30
29.29
29.23
29.19
29.00
25.31
25.27
25.18
24.65
22.68
14.08
f1 (ppm)

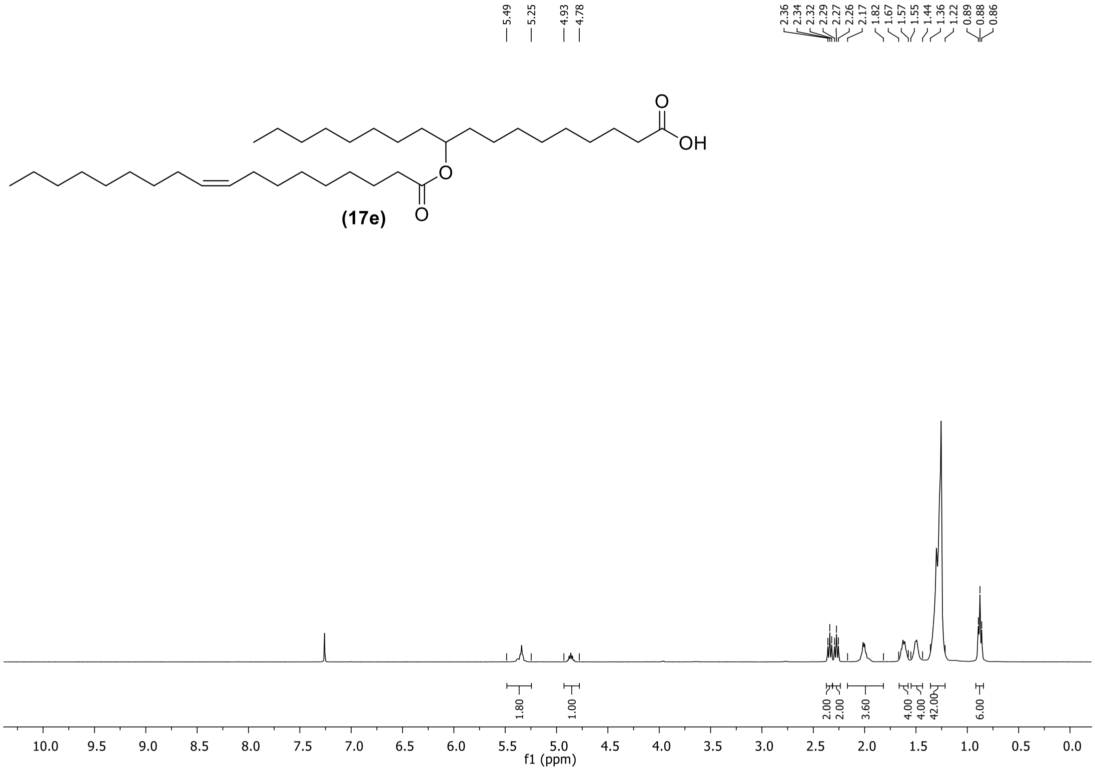
5.49
5.25
4.93
4.78
2.36
2.34
2.32
2.29
2.27
2.26
2.17
1.82
1.67
1.57
1.55
1.44
1.36
1.22
0.89
0.88
0.86
O
OH
O
O
(17e)
1.80
1.00
2.00
2.00
3.60
4.00
4.00
42.00
6.00
f1 (ppm)

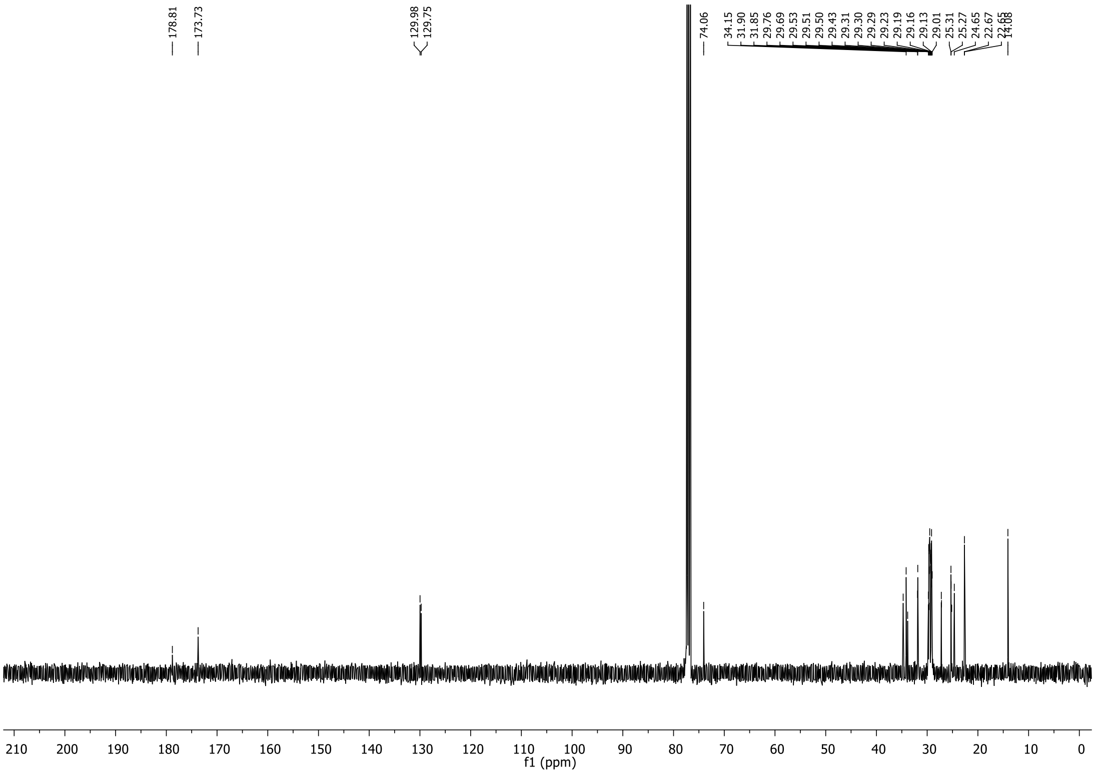
178.81
173.73
129.98
129.75
74.06
34.15
31.90
31.85
29.76
29.69
29.53
29.51
29.43
29.31
29.30
29.29
29.23
29.19
29.16
29.13
29.01
25.31
25.27
24.65
22.67
f1 (ppm)

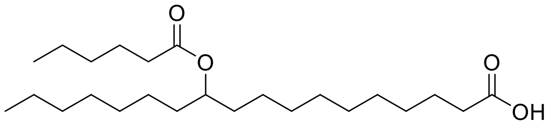

(17f)

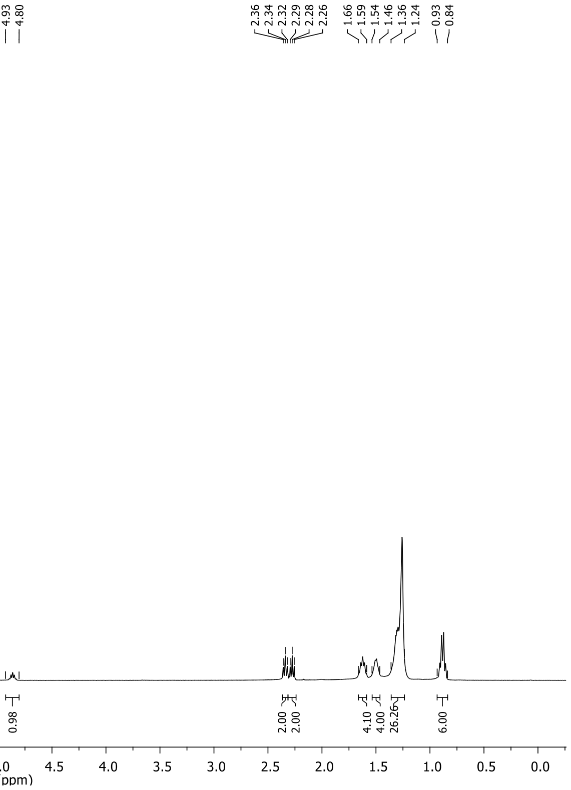
4.93
4.80
2.36
2.34
2.32
2.29
2.28
2.26
1.66
1.59
1.54
1.46
1.36
1.24
0.93
0.84
0.98
2.00
2.00
4.10
4.00
26.26
6.00
f1 (ppm)

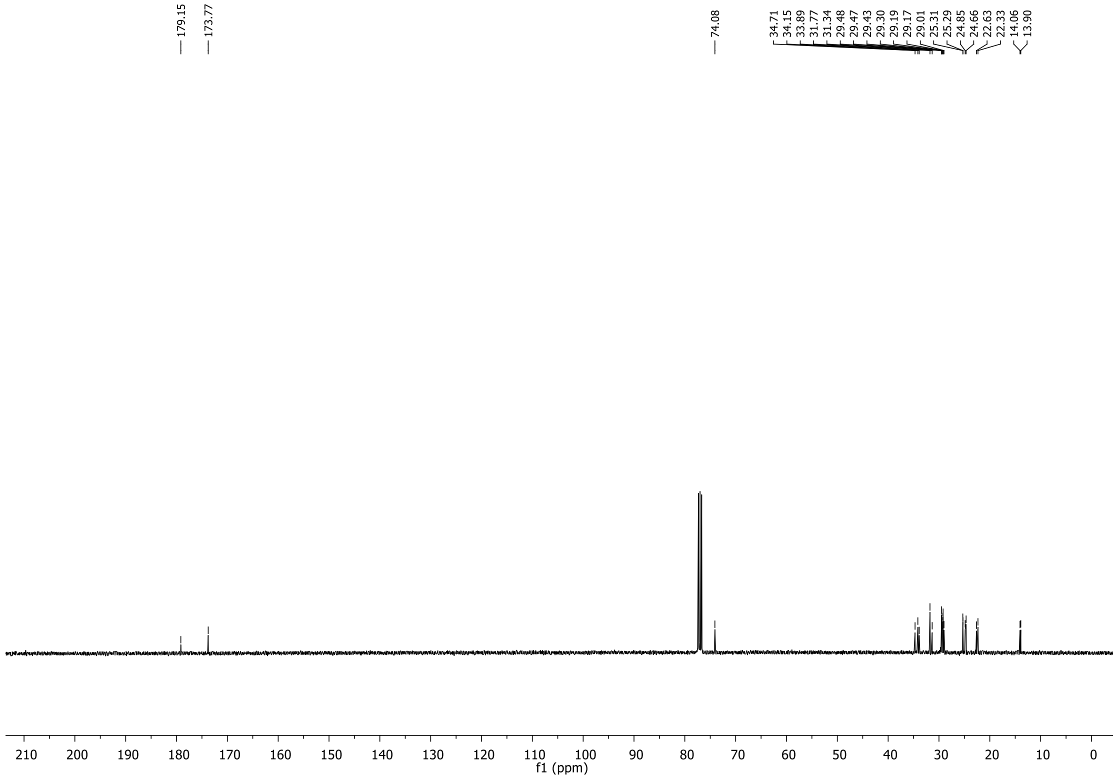
179.15
173.77
74.08
34.71
34.15
33.89
31.77
31.34
29.48
29.47
29.43
29.30
29.19
29.17
29.01
25.31
25.29
24.85
24.66
22.63
22.33
14.06
13.90
f1 (ppm)

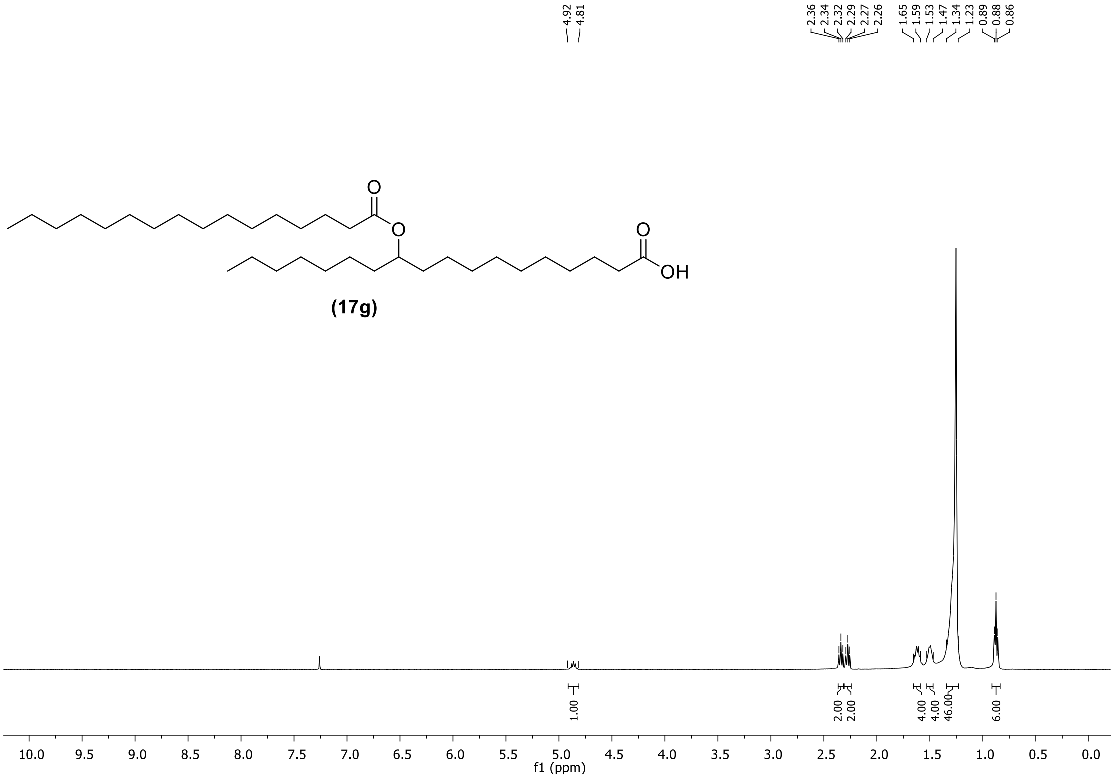

(17g)

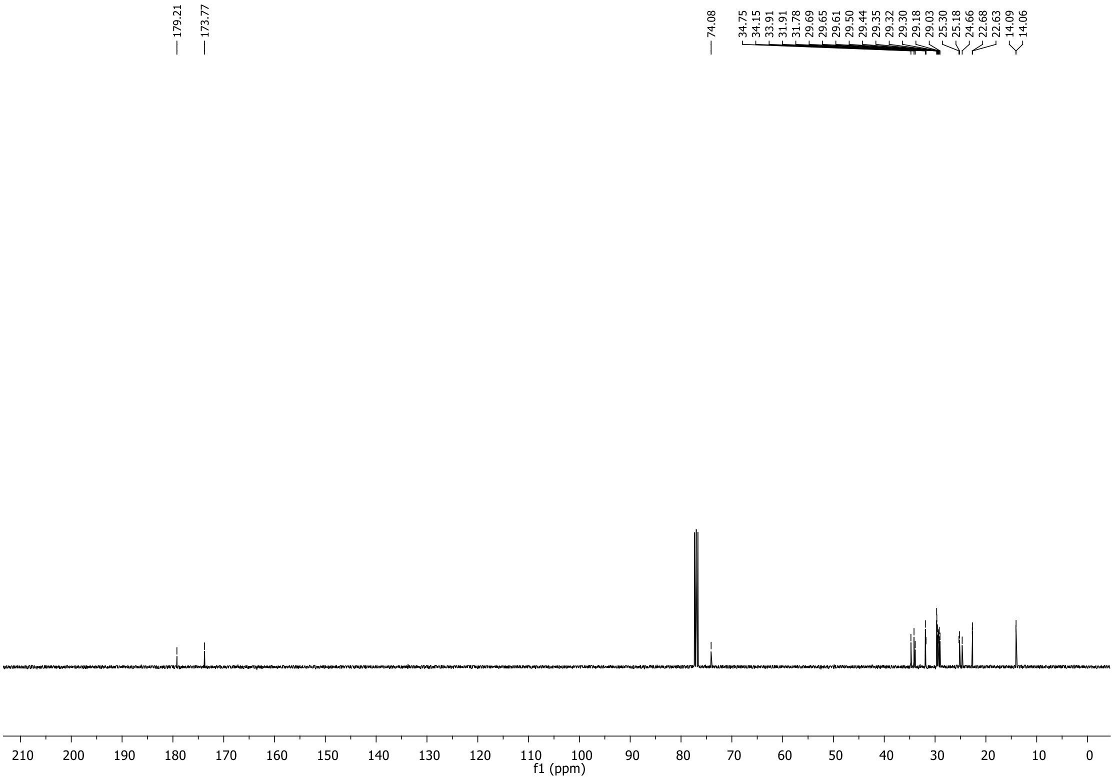

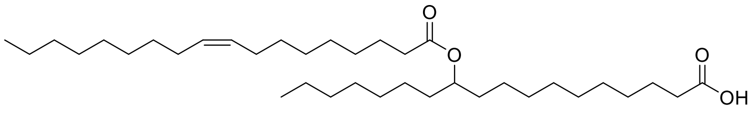

(17h)

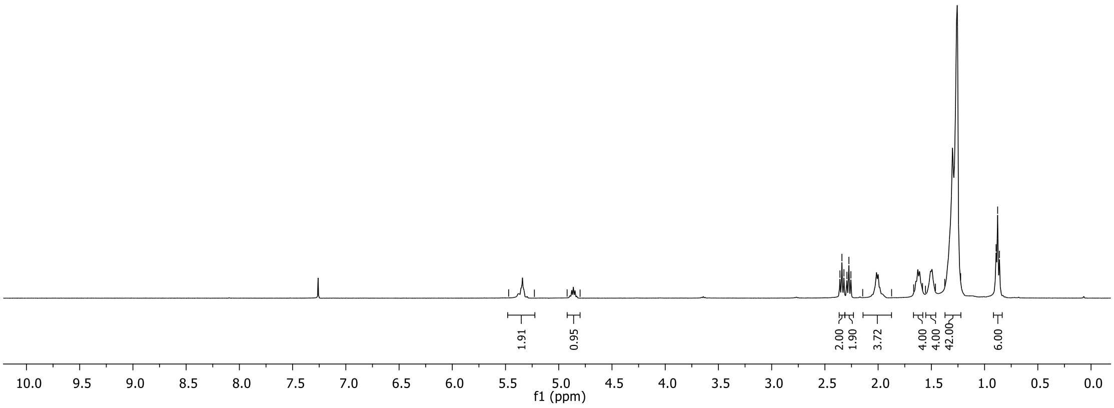

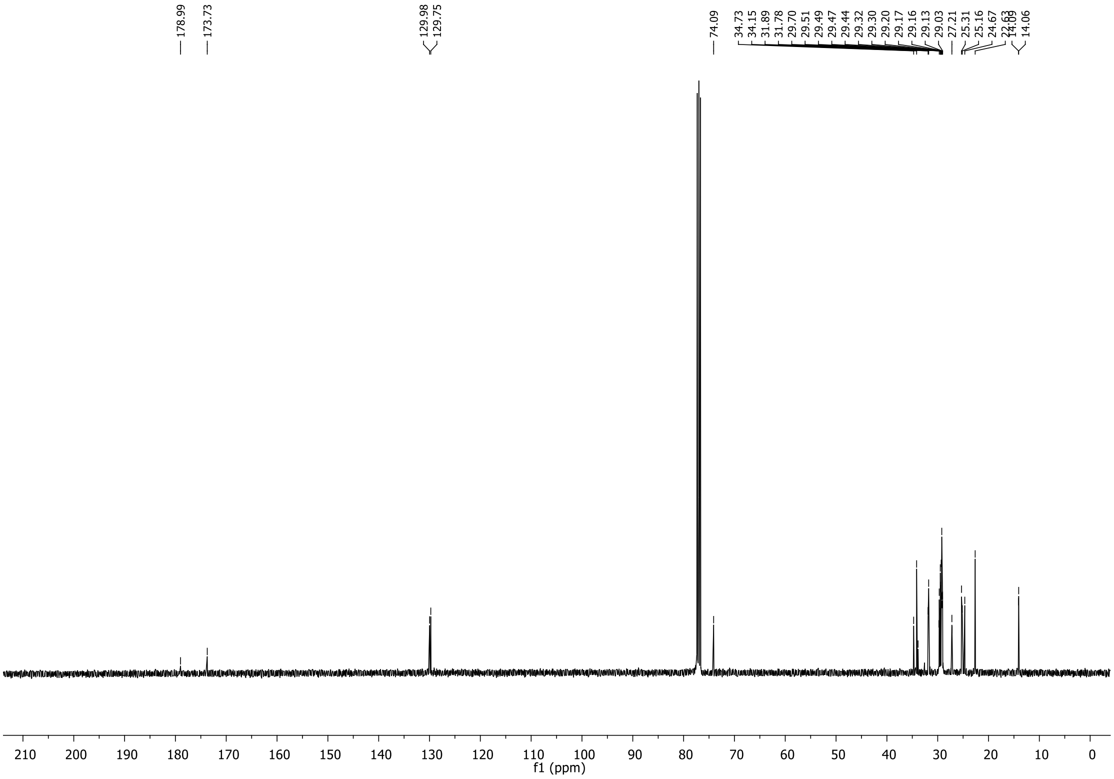

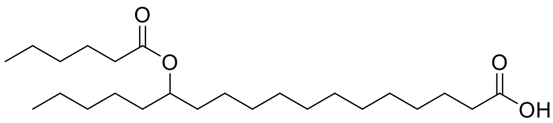

(17i)

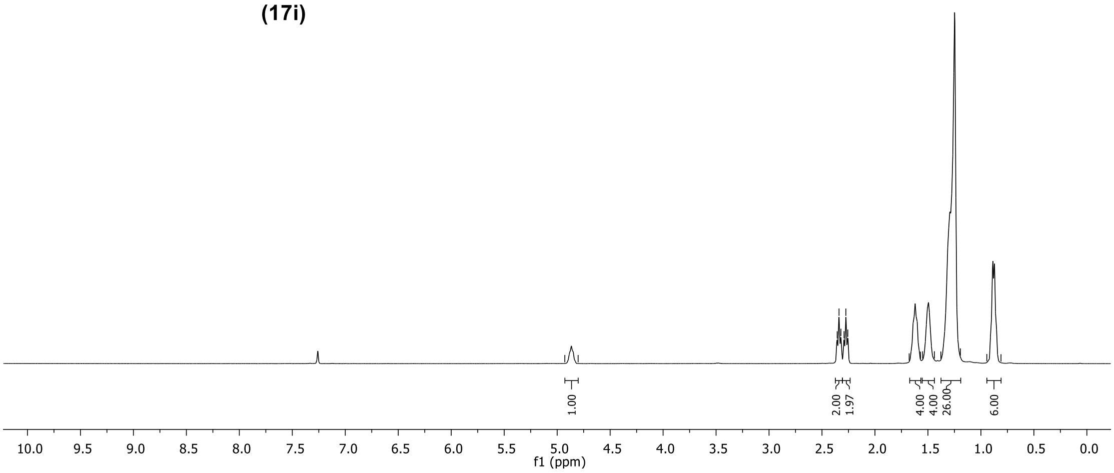
4.93
4.80
2.36
2.34
2.32
2.29
2.27
2.26
1.68
1.57
1.55
1.44
1.38
1.19
0.94
0.81
1.00
2.00
1.97
4.00
4.00
26.00
6.00
10.0 9.5 9.0 8.5 8.0 7.5 7.0 6.5 6.0 5.5 5.0 4.5 4.0 3.5 3.0 2.5 2.0 1.5 1.0 0.5 0.0
f1 (ppm)

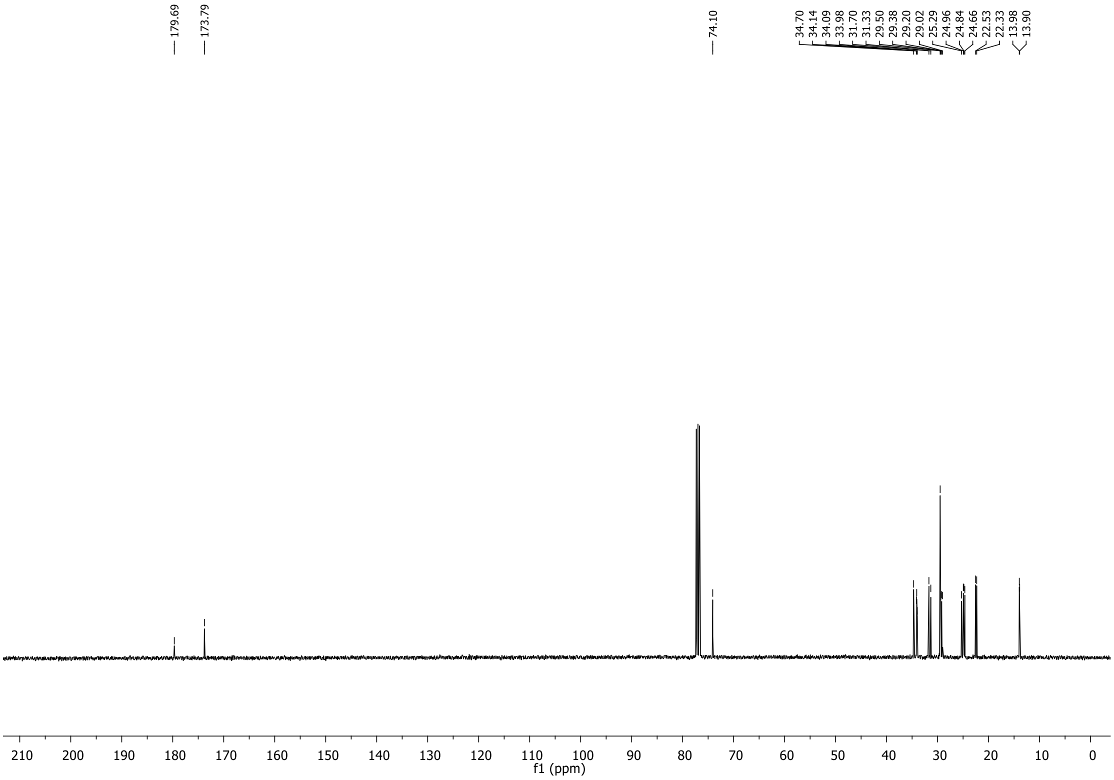
179.69
173.79
74.10
34.70
34.14
34.09
33.98
31.70
31.33
29.50
29.38
29.20
29.02
25.29
24.96
24.84
24.66
22.53
22.33
13.98
13.90
210 200 190 180 170 160 150 140 130 120 110 100 90 80 70 60 50 40 30 20 10 0
f1 (ppm)

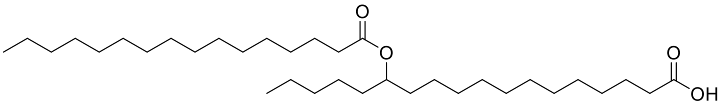
O
O
O
OH

(17j)

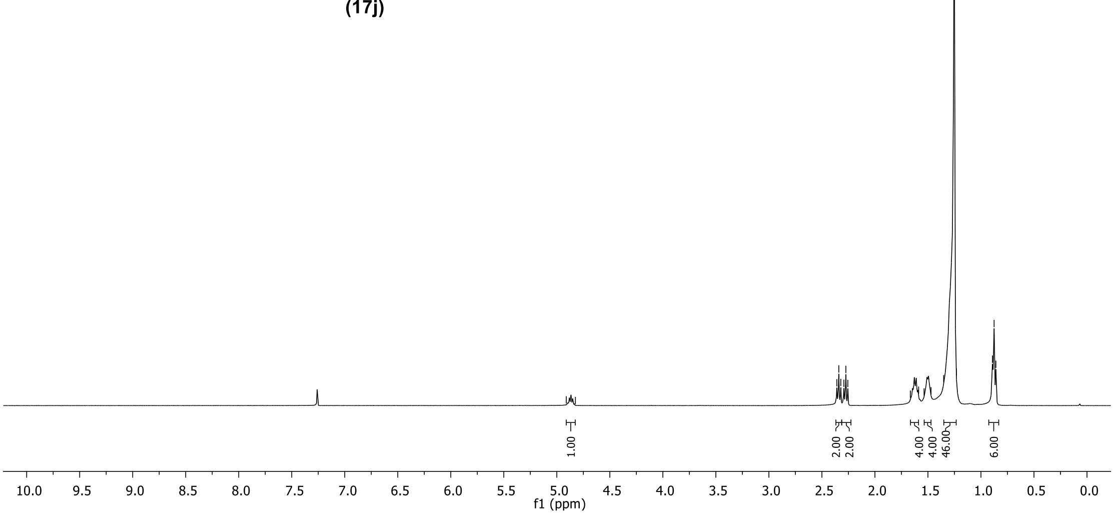
4.91
4.82
2.36
2.34
2.32
2.29
2.27
2.26
1.67
1.59
1.54
1.47
1.35
1.23
0.89
0.88
0.86
1.00
2.00
2.00
4.00
4.00
46.00
6.00
10.0 9.5 9.0 8.5 8.0 7.5 7.0 6.5 6.0 5.5 5.0 4.5 4.0 3.5 3.0 2.5 2.0 1.5 1.0 0.5 0.0
f1 (ppm)

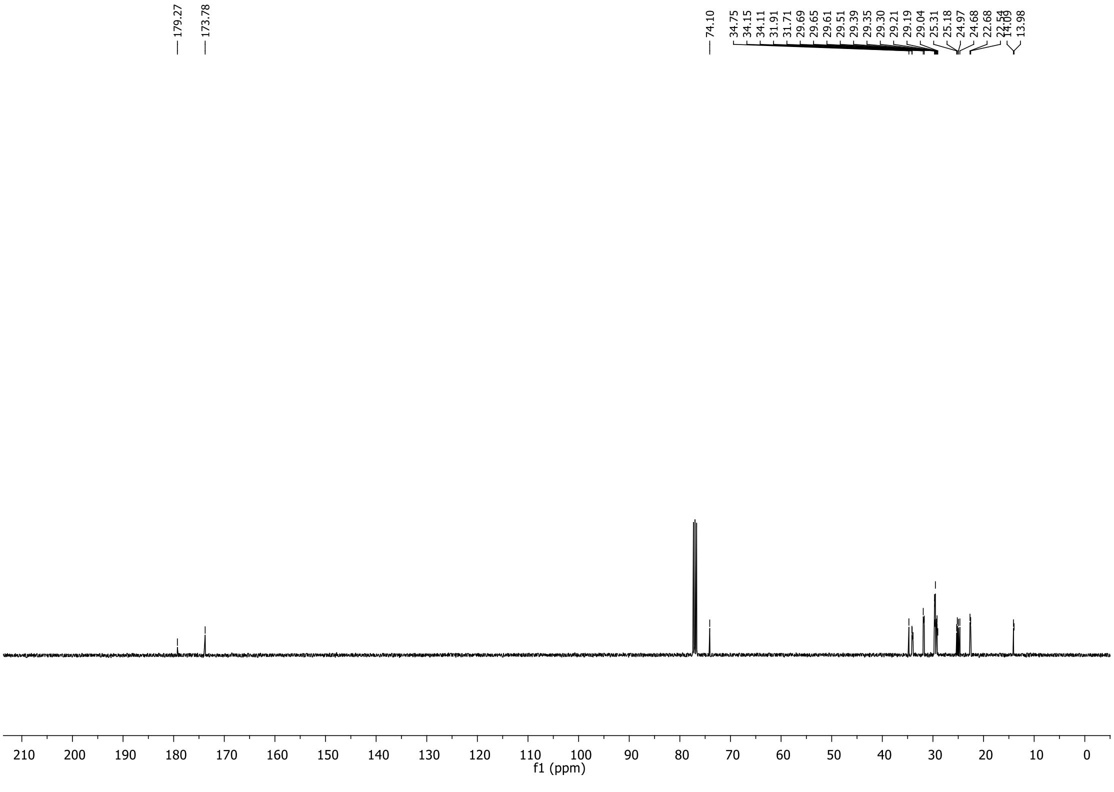
179.27
173.78
74.10
34.75
34.15
34.11
31.91
31.71
29.69
29.65
29.61
29.51
29.39
29.35
29.30
29.21
29.19
29.04
25.31
25.18
24.97
24.68
22.68
13.98
210 200 190 180 170 160 150 140 130 120 110 100 90 80 70 60 50 40 30 20 10 0
f1 (ppm)

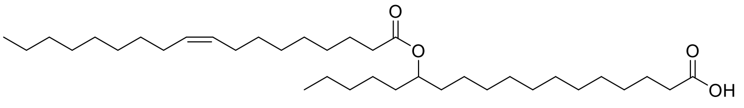

(17k)

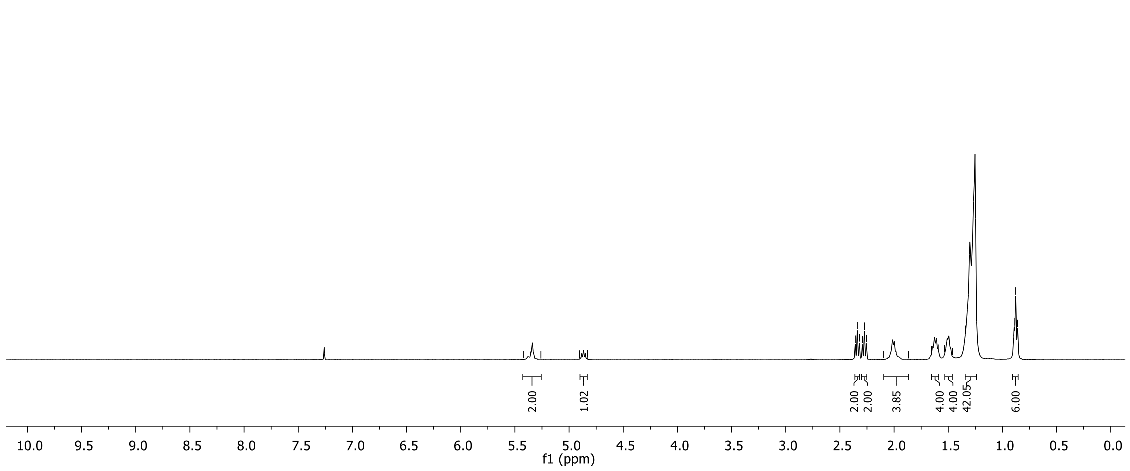

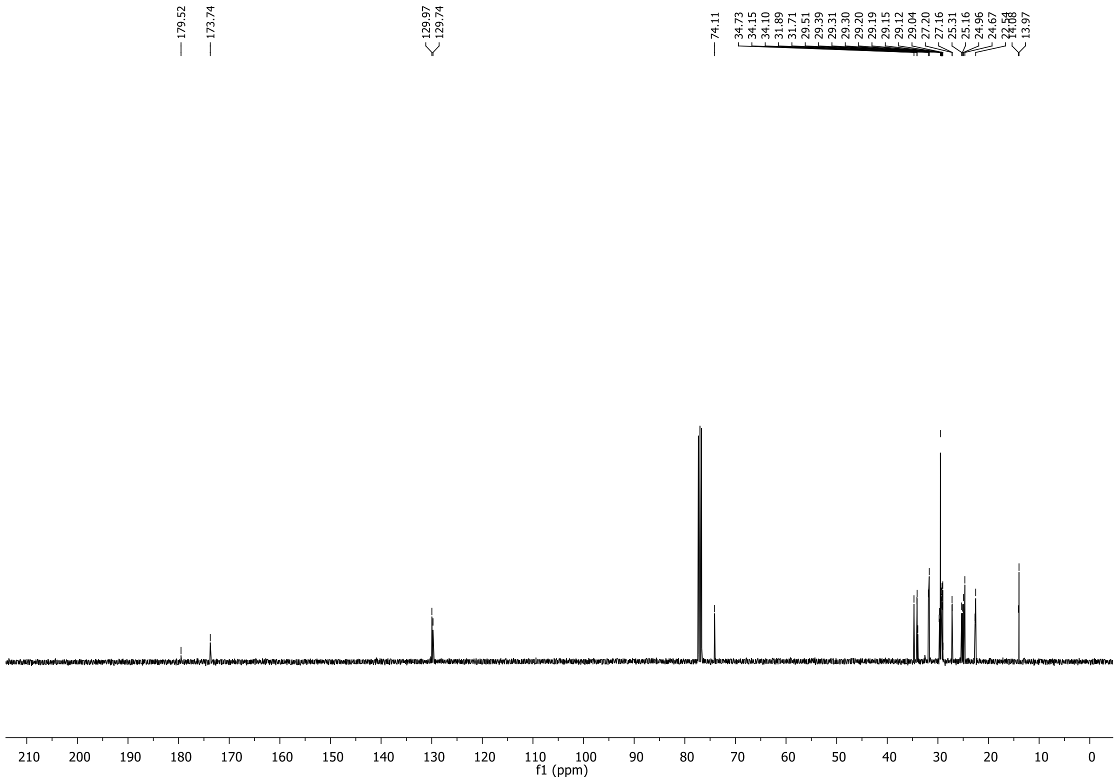

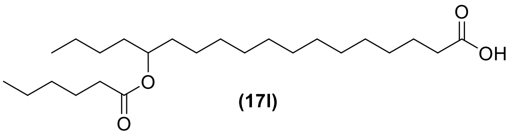
O
OH
O
O

(17l)

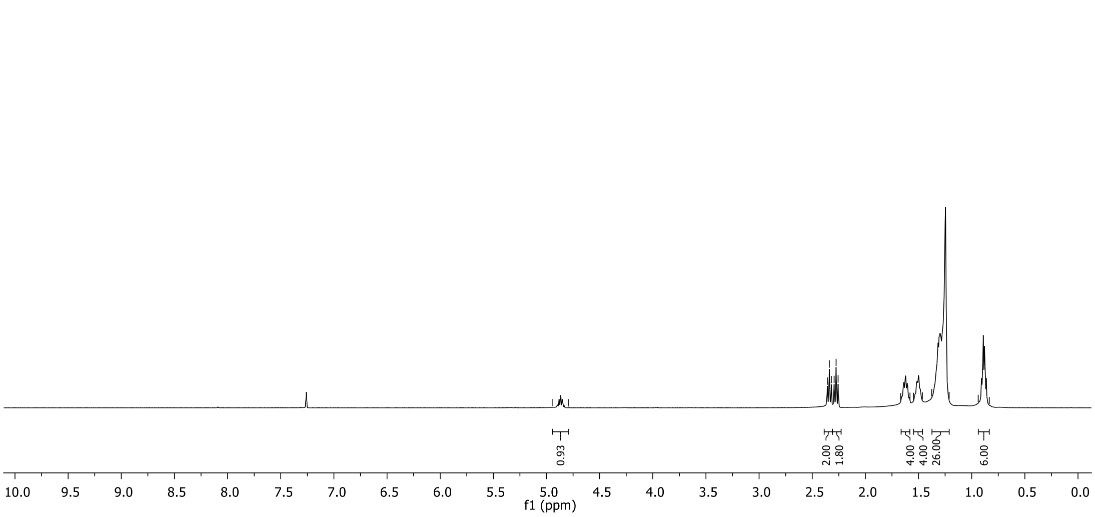
4.95
4.80
2.36
2.34
2.32
2.29
2.28
2.26
1.67
1.58
1.55
1.46
1.38
1.21
0.94
0.83
0.93
2.00
1.80
4.00
4.00
26.00
6.00
10.0 9.5 9.0 8.5 8.0 7.5 7.0 6.5 6.0 5.5 5.0 4.5 4.0 3.5 3.0 2.5 2.0 1.5 1.0 0.5 0.0
f1 (ppm)

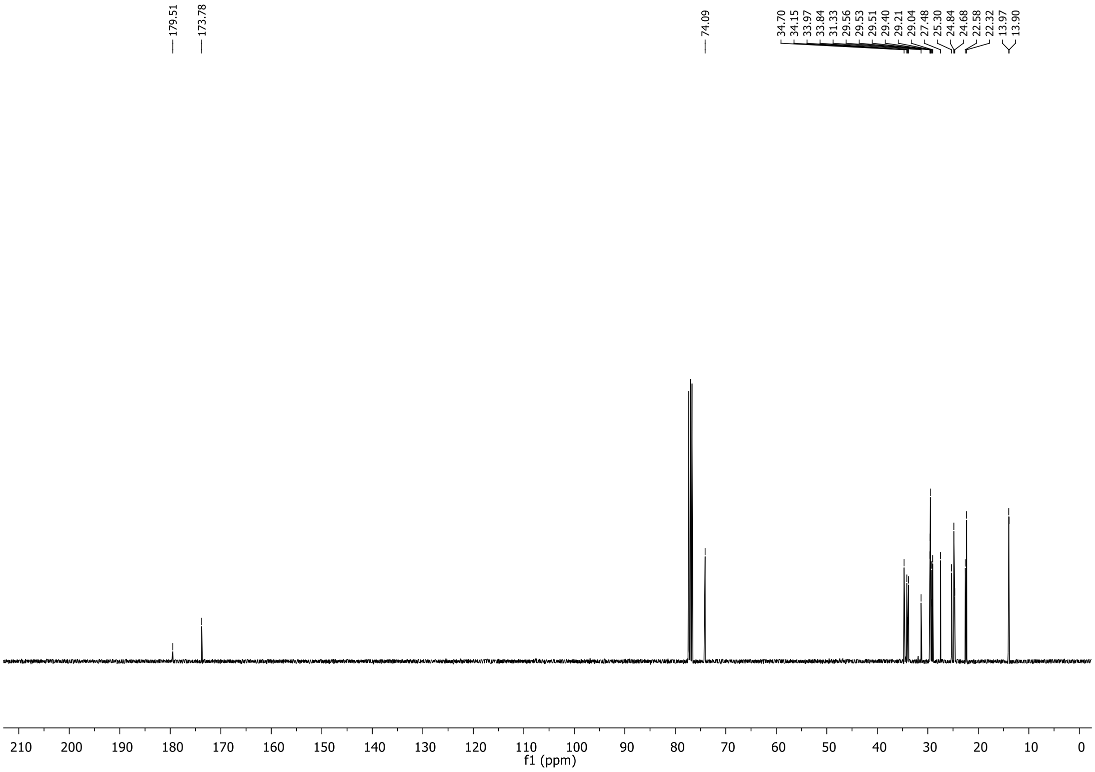
179.51
173.78
74.09
34.70
34.15
33.97
33.84
31.33
29.56
29.53
29.51
29.40
29.21
29.04
27.48
25.30
24.84
24.68
22.58
22.32
13.97
13.90
210 200 190 180 170 160 150 140 130 120 110 100 90 80 70 60 50 40 30 20 10 0
f1 (ppm)

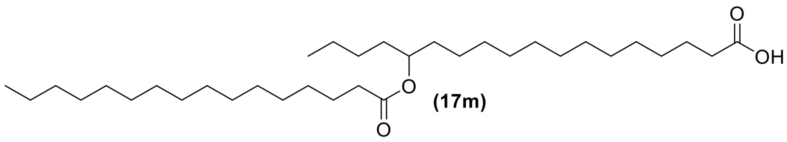
O
OH
O
O

(17m)

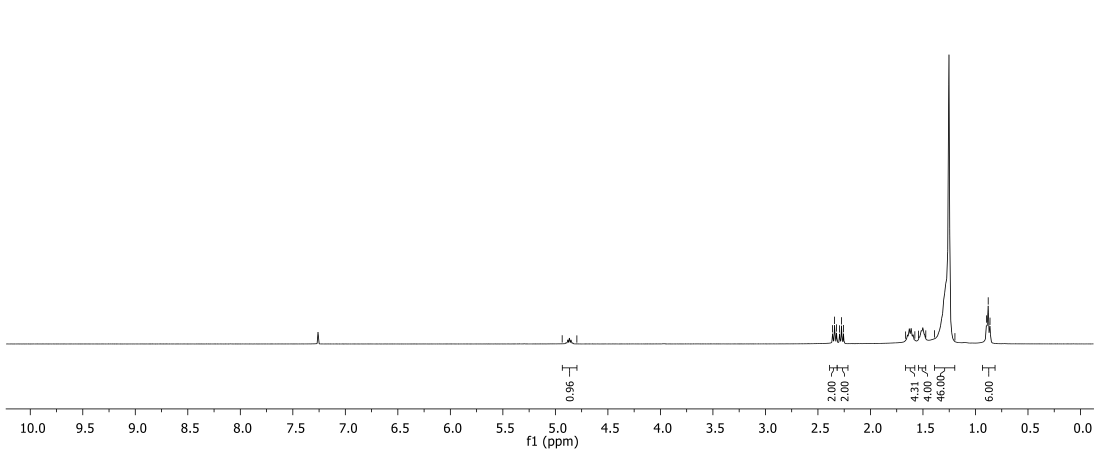
4.94
4.80
2.36
2.34
2.32
2.29
2.28
2.26
1.67
1.58
1.54
1.47
1.39
1.20
0.89
0.88
0.86
0.96
2.00
2.00
4.31
4.00
46.00
6.00
10.0
9.5
9.0
8.5
8.0
7.5
7.0
6.5
6.0
5.5
5.0
4.5
4.0
3.5
3.0
2.5
2.0
1.5
1.0
0.5
0.0
f1 (ppm)

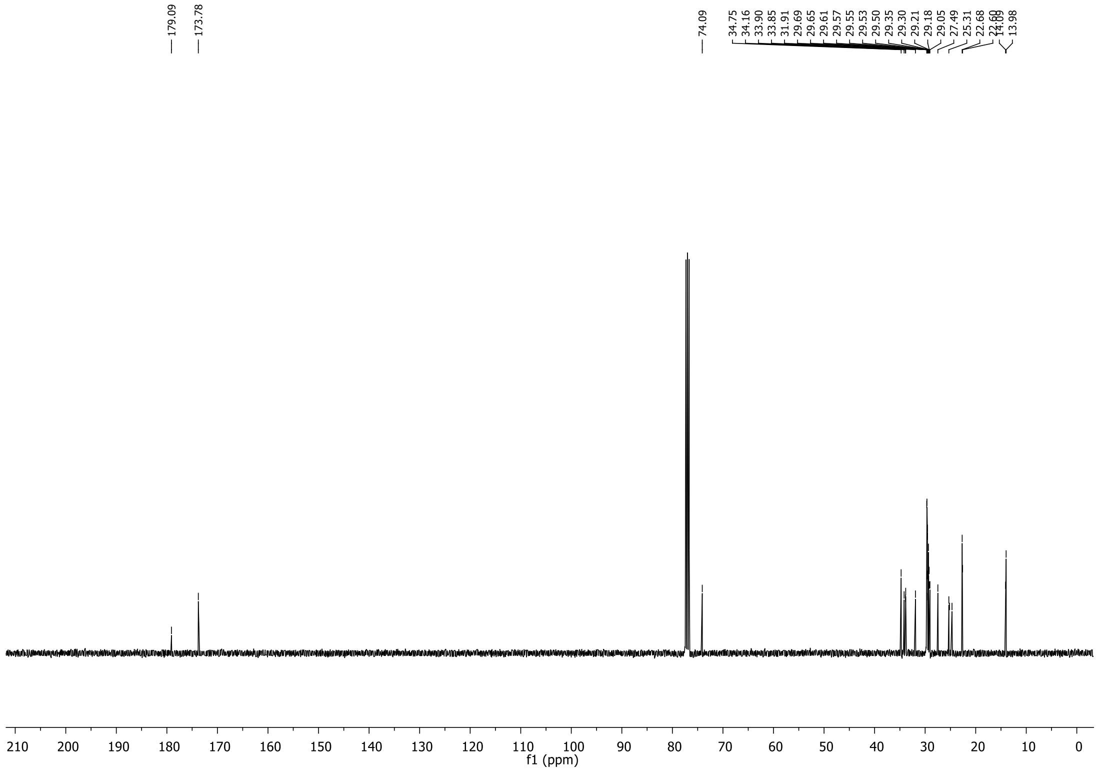
179.09
173.78
74.09
34.75
34.16
33.90
33.85
31.91
29.69
29.65
29.61
29.57
29.55
29.53
29.50
29.35
29.30
29.21
29.18
29.05
27.49
25.31
22.68
13.98
210
200
190
180
170
160
150
140
130
120
110
100
90
80
70
60
50
40
30
20
10
0
f1 (ppm)

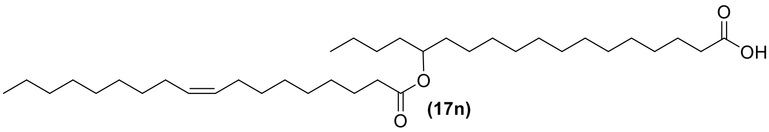
O
OH
O
O

(17n)

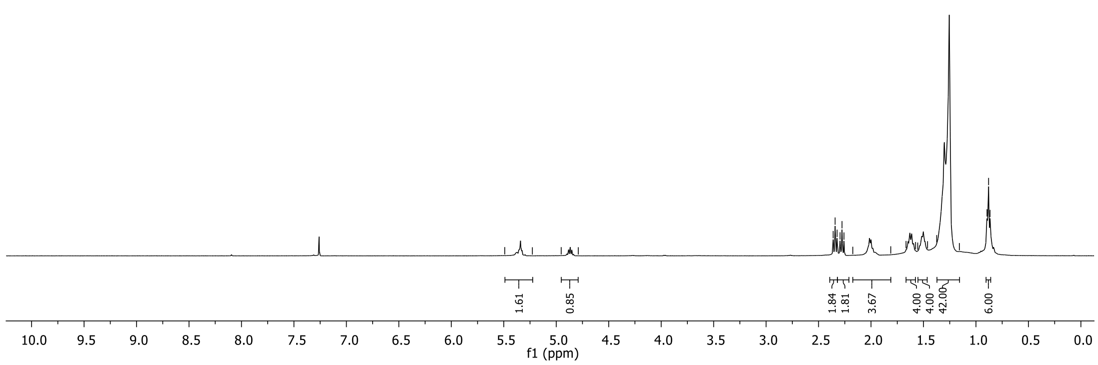
5.49
5.23
4.95
4.79
2.36
2.34
2.32
2.29
2.28
2.26
2.17
1.81
1.67
1.58
1.56
1.46
1.37
1.16
0.89
0.88
0.87
1.61
0.85
1.84
1.81
3.67
4.00
4.00
42.00
6.00
f1 (ppm)

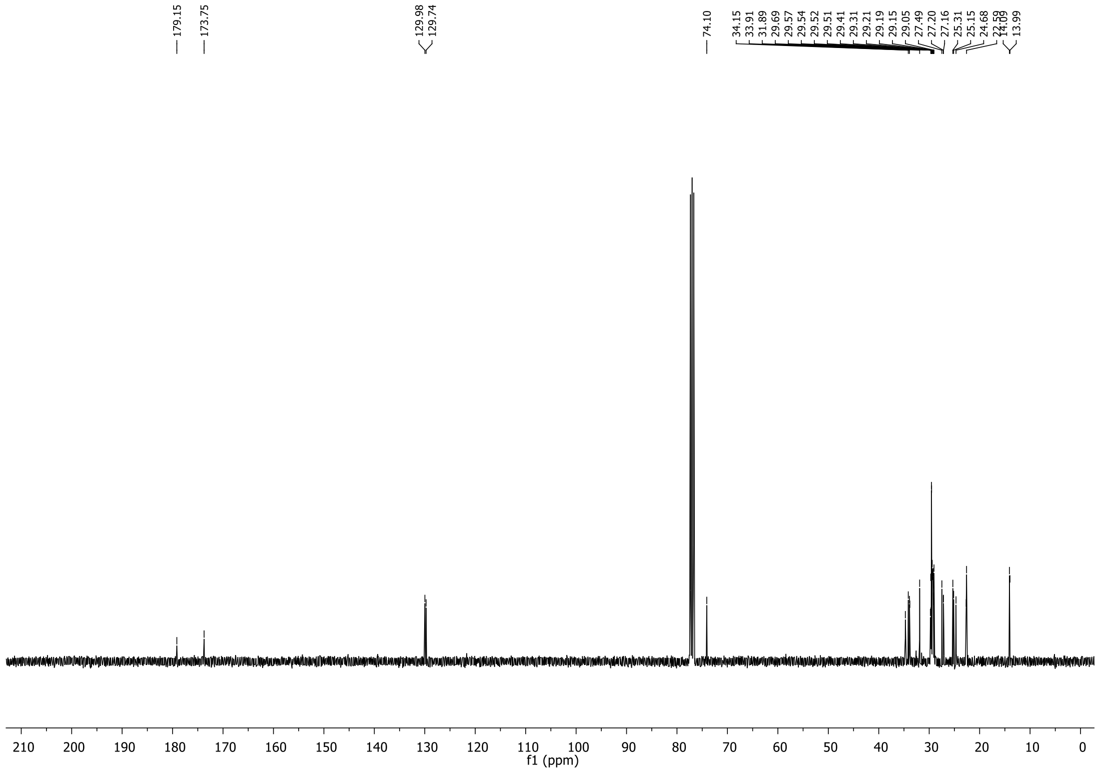
179.15
173.75
129.98
129.74
74.10
34.15
33.91
31.89
29.69
29.57
29.54
29.52
29.51
29.41
29.31
29.21
29.19
29.15
29.05
27.49
27.20
27.16
25.31
25.15
24.68
13.99
f1 (ppm)
